# 7-[^18^F]Fluoro-8-azaisatoic Anhydrides: Versatile Prosthetic Groups for the Preparation of PET Tracers

**DOI:** 10.1021/acs.jmedchem.3c01310

**Published:** 2023-08-25

**Authors:** Benedikt Gröner, Michael Willmann, Lisa Donnerstag, Elizaveta A. Urusova, Felix Neumaier, Swen Humpert, Heike Endepols, Bernd Neumaier, Boris D. Zlatopolskiy

**Affiliations:** † Forschungszentrum Jülich GmbH, Institute of Neuroscience and Medicine, Nuclear Chemistry (INM-5), Wilhelm-Johnen-Straße, 52428 Jülich, Germany; ‡ Faculty of Medicine and University Hospital Cologne, Institute of Radiochemistry and Experimental Molecular Imaging, University of Cologne, Kerpener Straße 62, 50937 Cologne, Germany; § Faculty of Medicine and University Hospital Cologne, Department of Nuclear Medicine, University of Cologne, Kerpener Straße 62, 50937 Cologne, Germany; ∥ Max Planck Institute for Metabolism Research, Gleueler Straße 50, 50931 Cologne, Germany

## Abstract

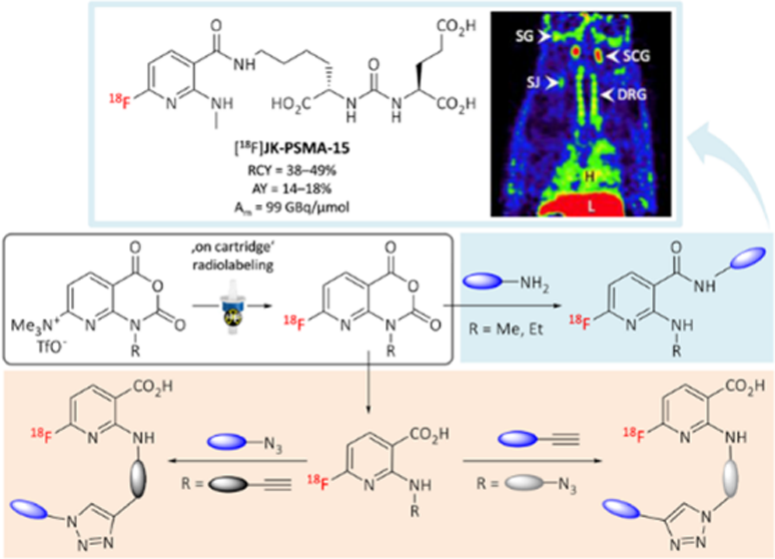

^18^F-Fluorination of sensitive molecules is often challenging, but can be accomplished under suitably mild conditions using radiofluorinated prosthetic groups (PGs). Herein, 1-alkylamino-7-[^18^F]fluoro-8-azaisatoic anhydrides ([^18^F]AFAs) are introduced as versatile ^18^F-labeled building blocks that can be used as amine-reactive or “click chemistry” PGs. [^18^F]AFAs were efficiently prepared within 15 min by “on cartridge” radiolabeling of readily accessible trimethylammonium precursors. Conjugation with a range of amines afforded the corresponding 2-alkylamino-6-[^18^F]fluoronicotinamides in radiochemical conversions (RCCs) of 15–98%. In addition, radiolabeling of alkyne- or azide-functionalized precursors with azidopropyl- or propargyl-substituted [^18^F]AFAs using Cu-catalyzed click cycloaddition afforded the corresponding conjugates in RCCs of 44–88%. The practical utility of the PGs was confirmed by the preparation of three ^18^F-labeled PSMA ligands in radiochemical yields of 28–42%. Biological evaluation in rats demonstrated excellent *in vivo* stability of all three conjugates. In addition, one conjugate ([^18^F]JK-PSMA-15) showed favorable imaging properties for high-contrast visualization of small PSMA-positive lesions.

## Introduction

Positron emission tomography (PET) is a noninvasive imaging technique that enables *in vivo* visualization and quantification of physiological processes by tracking the biodistribution of pharmaceuticals labeled with positron-emitting radionuclides. Unlike conventional imaging techniques such as computed tomography or magnetic resonance imaging, which primarily provide anatomical information, PET can be used for functional imaging on the cellular or molecular level.^1^ For example, PET imaging with ^68^Ga-labeled probes targeting prostate-specific membrane antigen (PSMA), a transmembrane glycoprotein that is highly overexpressed on malignant prostate cells,^2−7^ has proven instrumental for the detection and staging of prostate cancer (PCa).^2,3,7,8^ However, ^68^Ga-labeled PSMA radioligands are increasingly replaced by ligands labeled with fluorine-18 (like [^18^F]DCFPyL,^9^ [^18^F]JK-PSMA-7,^10^ or [^18^F]PSMA-1007^11^), which is the most frequently used radionuclide for PET imaging. Important advantages of fluorine-18 over gallium-68 include the accessibility of [^18^F]fluoride ([^18^F]F^–^) in >100 GBq quantities via the ^18^O(p,n)^18^F nuclear reaction and a longer half-life (*t*_1/2_ = 110 vs 68 min), which enable large-scale, centralized production and distribution of ^18^F-labeled radioligands.^1,12^ In addition, due to the lower positron energy of fluorine-18 compared to gallium-68 (*E*_max_ = 0.63 vs 1.9 MeV), imaging with ^18^F-labeled probes offers an improved spatial resolution. While a few direct ^18^F-labeling methods described in the literature are sufficiently mild to be applied to sensitive substrates (e.g., SiFA, boron-^18^F, or [^18^F]AlF-based approaches), most of them require harsh and/or strictly anhydrous reaction conditions that are incompatible with sensitive compounds or biomolecules.^1,12−14^ As a consequence, such substrates are often labeled by indirect radiofluorination using ^18^F-labeled prosthetic groups (PGs), which can be conjugated to the target molecule under mild conditions.^12,15^ Among the most common traditional PGs are radiolabeled active esters like *N*-succinimidyl 4-[^18^F]fluorobenzoate ([^18^F]SFB)^13,16−19^ or 2,3,5,6-tetrafluorophenyl 6-[^18^F]fluoronicotinate ([^18^F]FPy-TFP),^6,20^ which readily react with primary amine groups present in peptides or proteins. However, their preparation often involves time-consuming multistep procedures^21−23^ or is complicated by the formation of reactive side products that cannot be easily separated from the radiolabeled active ester.^6,20,24^ In addition, biomolecules like peptides or proteins typically contain more than one reactive amino group, which can lead to the formation of isomeric side products during conjugation reactions. An alternative approach for indirect radiolabeling is based on ^18^F-labeled “click chemistry” PGs with an alkyne or azide moiety, which can be conjugated to suitably functionalized precursors using the copper-catalyzed alkyne-azide cycloaddition (CuAAC).

Herein, we describe 1-alkylamino-7-[^18^F]fluoro-8-azaisatoic anhydrides ([^18^F]AFAs) as easily accessible ^18^F-labeled building blocks that can be utilized for radiolabeling via acylation or “click chemistry”. We also show that amine-reactive [^18^F]AFAs can preferentially acylate the more reactive or sterically accessible amine present in a mixture of amines. Finally, we exemplify the application of [^18^F]AFAs for the radiofluorination of different PSMA ligands and provide preliminary results of *in vivo* evaluation of the resulting radiotracers by small animal PET imaging.

## Results and Discussion

### Synthesis of Radiolabeling Precursors **4a–d** and Reference Compounds **3e–h**

**Scheme 1:**
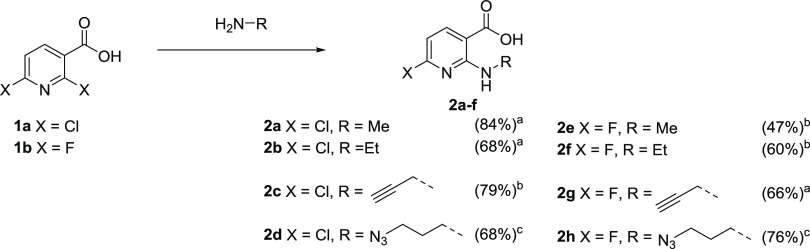
Preparation of 2-Alkylamino-6-halonicotinic Acids Reaction conditions: (a) THF, 80 °C, 3 d; (b) THF, 55 °C, 4 d; (c) THF, 45 °C, 7 d.

**Scheme 2:**
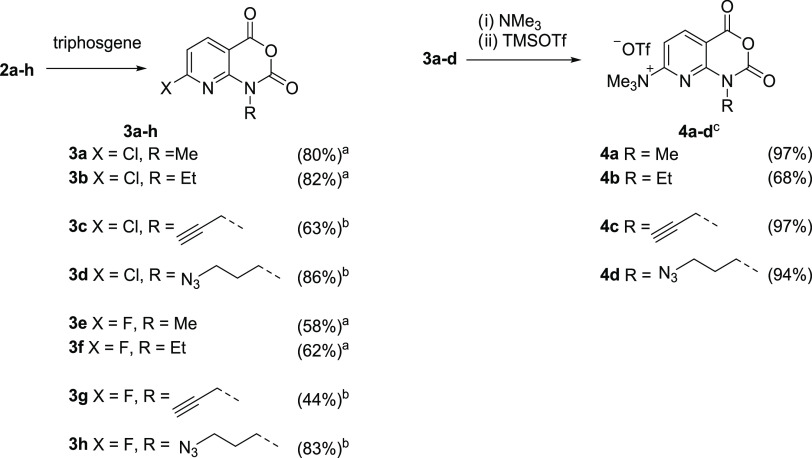
Synthesis of Radiolabeling Precursors and Reference Compounds Reaction conditions: (a) 1,4-dioxane, reflux, 3 d; (b) 1,4-dioxane, 60 °C, 3 d; (c) (i) THF, r.t., 1 h; (ii) CH_2_Cl_2_, r.t., 40 min.

*N,N,N*-Trimethylammonium triflate radiolabeling precursors^25^**4a**–**d** and reference compounds **3e**–**h** were prepared starting from 2,6-dichloronicotinic (**1a**) or 2,6-difluoronicotinic acid (**1b**), respectively (Schemes 1 and 2).

To this end, 2,6-dihalonicotinic acids were regioselectively aminated at the second position by heating them with a 3- to 20-fold molar excess of the respective amine over 2–7 days, which afforded the corresponding 2-alkylamino-6-halo-nicotinic acid intermediates (**2a**–**h**) in 47–84% yields (Scheme 1).^26^ Subsequent cyclization of **2a**–**h** with triphosgene in boiling 1,4-dioxane gave the 1-alkylamino-7-halo-8-azaisatoic anhydrides **3a**–**h** in 44–83% yields (Scheme 2).

**Figure 1:**
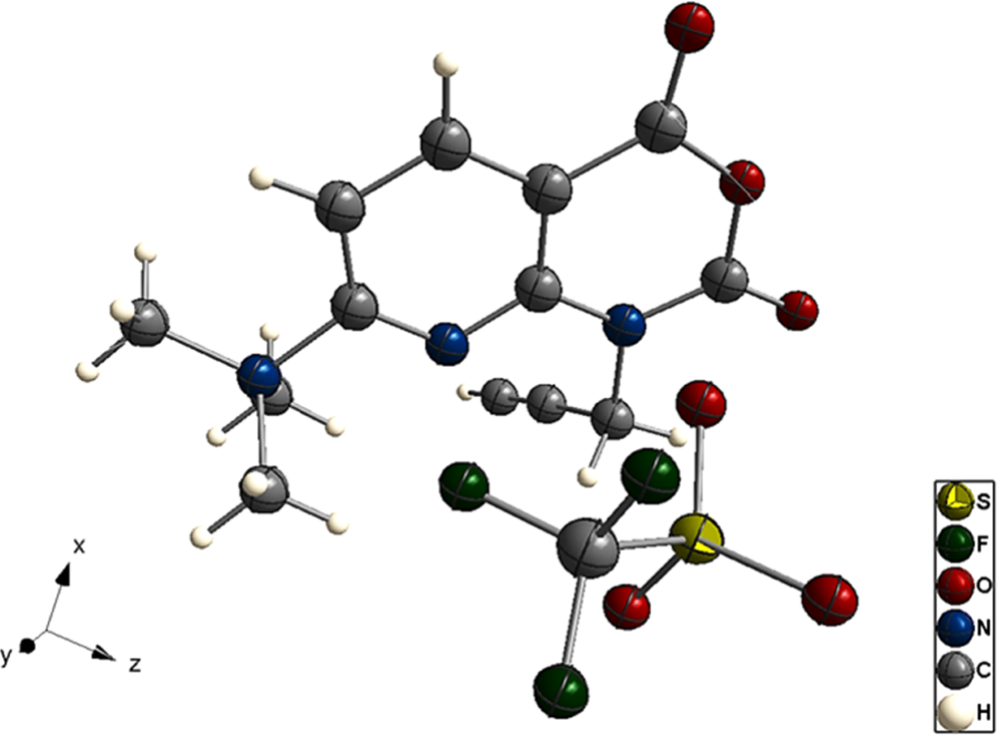
X-ray crystal structure of alkyne-substituted azaisatoic anhydride **4c**. View along *y*-axis.

*N,N,N*-Trimethylammonium triflate precursors **4a**–**d** were prepared by treatment of **3a**–**d** with an excess of trimethylamine in THF followed by anion metathesis with trimethylsilyl triflate. All four precursors were stable at −20 °C under argon for at least 6 months. Single crystals of **4c** for X-ray crystallography were obtained by slow evaporation of a solution of the compound in MeCN/*t*BuOH (1:4) at ambient temperature. Crystallographic data are provided in Figure 1.

### Radiosynthesis of ^18^F-Labeled AFAs [^18^F]**3e–f** and Model Compounds [^18^F]**5a–m**

We first attempted to radiolabel the azaisatoic anhydrides **4a** and **4b** according to the “minimalist protocol”^27^ as follows. [^18^F]F^–^ was loaded onto an anion exchange resin and eluted with a solution of the corresponding radiolabeling precursor in MeOH. Following the evaporation of MeOH, DMSO or MeCN were added and the resulting solution was briefly heated. This approach afforded the desired radiolabeled building blocks, albeit in highly variable radiochemical conversions (RCCs) of 10–60%, as determined by radio-HPLC analysis with post-column injection (for details, see ref (28) and Experimental Section). Based on significant contamination of the products by the corresponding acids and methyl esters, we reasoned that these results could be explained by the insufficient thermal/solvolytic stability of 8-azaisatoic anhydrides. Therefore, we next tested a protocol for “on cartridge” radiofluorination of highly activated precursors, which enabled ^18^F-labeling at ambient temperature and obviated the need for evaporation steps.^29^ To this end, [^18^F]F^–^ trapped on an anion exchange cartridge (PS-HCO_3_^–^) was slowly eluted with a solution of the respective precursor (**4a** or **4b**) in MeCN/*t*BuOH (1:4), furnishing the labeled products [^18^F]**3e** or [^18^F]**3f** in RCCs of 70–80% and radiochemical purities (RCPs) of >99% (*n* > 50).

**Scheme 3:**
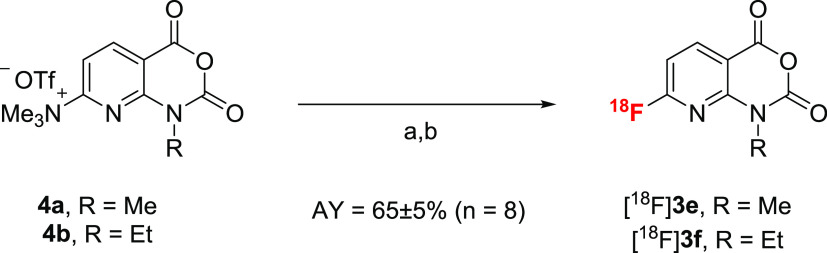
“On Cartridge” Radiolabeling of Azaisatoic Anhydrides **4a** and **4b** Reaction conditions: (a) [^18^F]F^–^-elution (from PS-HCO_3_^–^ cartridge) with a solution of the respective precursor in MeCN/*t*BuOH (1:4) over 2 min; (b) removal of unreacted precursor and [^18^F]F^–^ by solid-phase extraction (SPE). AY—activity yield.

Unreacted *N,N,N*-trimethylammonium triflate precursors were effectively removed by solid-phase extraction (SPE) using polymer reversed-phase cartridges (like Oasis HLB Plus Short), affording analytically pure [^18^F]**3e** and [^18^F]**3f** in activity yields (AYs) of 65 ± 5% (*n* = 8 for each) within only 15 min (Scheme 3).

**Figure 2:**
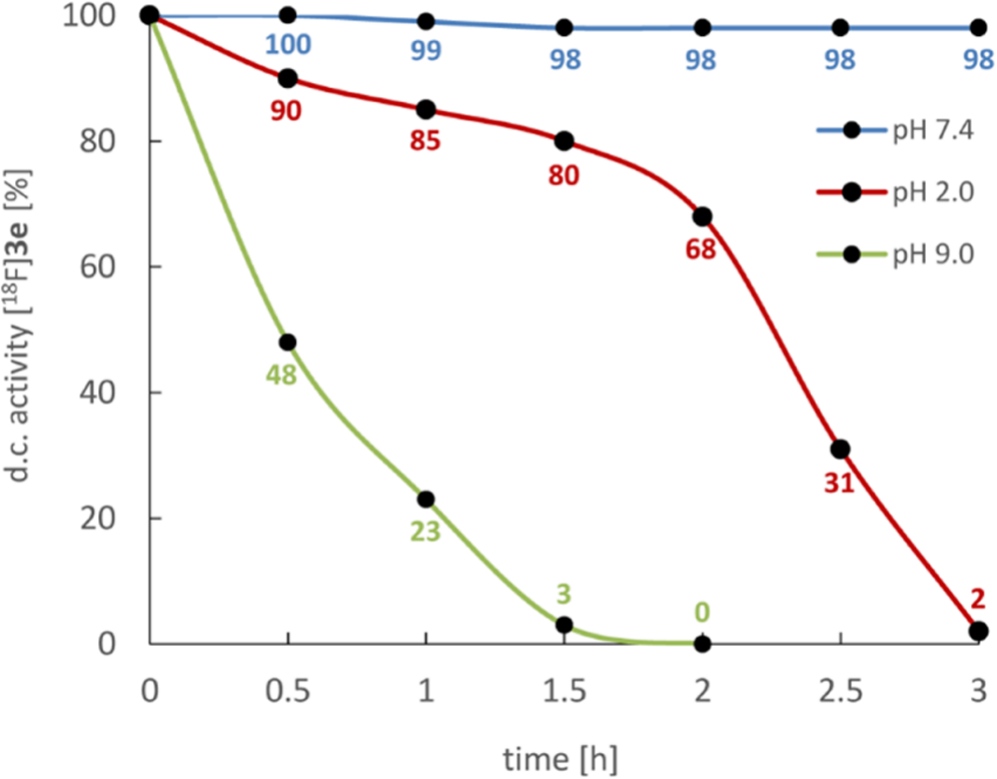
Stability of [^18^F]**3e** at pH 2.0 (30 mM citric acid, 8 mM HCl, 61 mM NaCl; red), pH 7.4 (50 mM TRIS·HCl; blue), and pH 9.0 (50 mM Na_2_CO_3_/Na_2_HCO_3_; green) at 20 °C. The percentage of intact [^18^F]**3e** at the different time points was determined by radio-HPLC and corrected for decay.

The hydrolytic stability of [^18^F]**3e** was investigated at different pH values (Figure 2). [^18^F]**3e** proved to be stable for several hours at a physiological pH value (98% intact after 3 h at pH 7.4), while it rapidly decomposed in basic solution (half-life <30 min at pH 9.0) and showed moderate stability in acidic medium (half-life <2.5 h at pH 2.0).

**Scheme 4:**
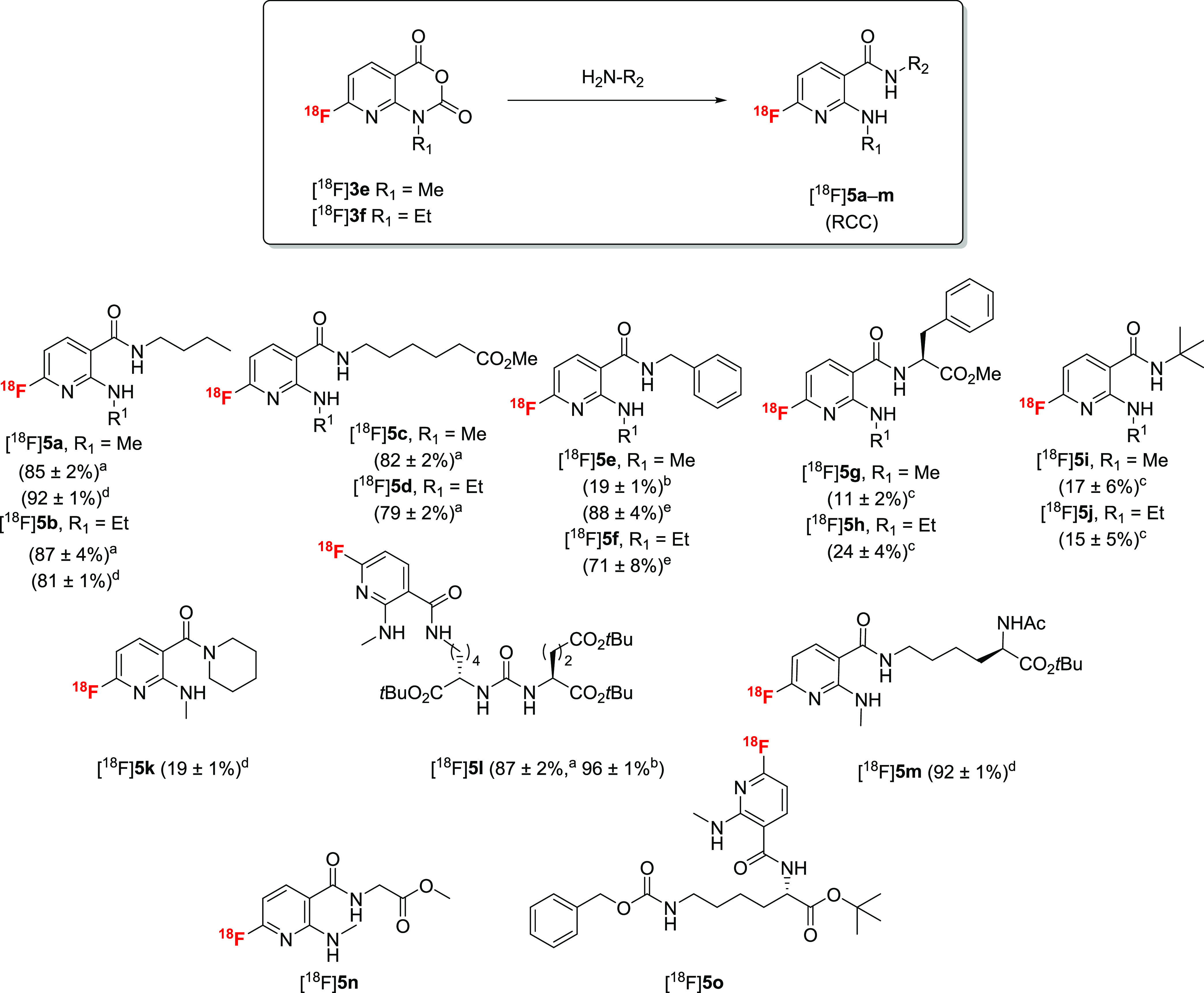
Preparation of [^18^F]**5a**–**m** by Conjugation of [^18^F]**3e** and [^18^F]**3f** with Various Amines (10 μmol) Reaction conditions: (a) 40 °C, 10 min, MeCN, (b) 60 °C, 15 min, MeCN, (c) 110 °C, 20 min, DMF, (d) 35 °C, 10 min, 20% MeCN in 0.2 M borate buffer (pH 8.7), (e) 80 °C, 10 min, 20% MeCN in 0.2 M borate buffer (pH 8.7). Following radiolabeling, H_2_O (1 mL) was added to the reaction mixture and radiochemical conversions (RCCs) were determined by radio-HPLC with post-column injection. Compounds [^18^F]**5n** and [^18^F]**5o** were obtained as side products in competition experiments with different amino acid derivatives (see text and Tables 1 and 2).

The suitability of [^18^F]**3e** and [^18^F]**3f** as amine-reactive PGs was first assessed using *n*-butylamine and several other primary and secondary amines as model substrates (Scheme 4). Given the structural similarity and comparable base strength, *n*-butylamine (*p*K_a_ = 10.6^30,31^) can be considered as a surrogate for the lysine side chain (*p*K_a_ around 10.5^30,31^) present in typical PSMA-binding motifs (like Glu-ureido-Lys) and other biomolecules. The reaction between [^18^F]**3e** or [^18^F]**3f** and *n*-butylamine (10 μmol) in MeCN proceeded within 10 min at 40 °C and afforded [^18^F]**5a** or [^18^F]**5b** in RCCs of >85%. Various other amines could also be acylated with [^18^F]**3e** or [^18^F]**3f** at 40–110 °C for 10–20 min in MeCN or DMF, affording the radiofluorinated 2-(alkylamino)nicotinamides [^18^F]**5c**–**m** in RCCs of 11–96% (Scheme 4).

Based on the results obtained for the different model compounds, conjugation with long-chained terminal amines gave good to excellent RCCs ([^18^F]**5a**–**d**, [^18^F]**5l**,**m**), whereas the RCCs for α-branched amines were lower ([^18^F]**5g**–**j**). Conjugation with piperidine as a model secondary amine yielded the corresponding radiolabeled amide [^18^F]**5k** in moderate RCCs of 19 ± 1%.

Biomolecules like peptides and proteins are usually insoluble in pure organic solvents. To determine the suitability of [^18^F]AFAs for bioconjugation in aqueous medium, we also acylated *n*-butylamine with [^18^F]**3e** using 20% MeCN in borate buffer (pH 7.4), which afforded the desired product [^18^F]**5a** in 65% RCC. RCCs of >90% were obtained with 20% MeCN in borate buffer (pH 8.7) as reaction solvent (Scheme 4).

In general, [^18^F]AFAs [^18^F]**3e** and [^18^F]**3f** appeared to be less reactive acylating agents than the radiofluorinated OSu or OTfp active esters conventionally applied for indirect radiolabeling. Accordingly, their application could enable regioselective conjugation to the less sterically hindered/more nucleophilic amino group like the ε-amino group in lysine-containing biomolecules.

In order to test this assumption, we performed competition experiments with *n*-butylamine and different amino acid esters in 0.2 M sodium borate buffer (pH 8.7) at 35 °C for 10 min using the *N*-methyl-substituted AFA [^18^F]**3e** as acylating agent (Table 1). Under these conditions, 1:1 mixtures of *n*-butylamine and amino acid esters afforded *n*-butyl amide [^18^F]**5a** as a single product. The only exception was H-Gly-OMe, the addition of which led to the concurrent formation of 2–3% of the radiolabeled Gly derivative [^18^F]**5n** (for structure, see Scheme 4).

**Table 1:** Competition between *n*BuNH_2_ and Different Amino Acid Esters for Conjugation with [^18^F]**3e**^a^

	RCC [%, *n* = 3]	
added amino acid derivative	[^18^F]**5a**	other products
none	92 ± 1	
H-Phe-OH	35 ± 1	0
H-Gly-OMe	81 ± 2	3 ± 1^b^
H-Phe-OMe	88 ± 1	0
H-Pro-OMe	81 ± 1	0
H-Lys(Z)-O*t*Bu	84 ± 1	0
H-Ala-OMe	89 ± 1	0
Ac-Lys(H)-O*t*Bu	83 ± 1	0

a Experiments were performed with 10 μmol of *n*BuNH_2_ and equimolar amounts of the indicated amino acid esters. Reaction conditions: 10 min at 35 °C with 20% MeCN in 0.2 M borate buffer (pH 8.7, 0.5 mL) as the reaction solvent.

b Acylated Gly-OMe ([^18^F]**5n**).

Similarly, acylation of 1:1 mixtures of the *N*-ε- or *N*-α-protected lysine esters H-Lys(Z)-O*t*Bu and Ac-Lys(H)-O*t*Bu with [^18^F]**3e** furnished side-chain-radiolabeled [^18^F]**5m** (for structure, see Scheme 4) as a single product (Table 2).

**Table 2:** Competition between H-Lys(Z)-O*t*Bu and Ac-Lys(H)-O*t*Bu for the Conjugation with [^18^F]**3e**^a^

	RCC [%; *n* = 3]	
ratio [H-Lys(Z)-O*t*Bu : Ac-Lys(H)-O*t*Bu]	[^18^F]**5m**	[^18^F]**5o**
1:1	85 ± 1	0 ± 0
2:1	79 ± 2	2 ± 2
4:1	71 ± 1	11 ± 1
8:1	28 ± 3	24 ± 2
10:1	22 ± 2	25 ± 1

a Experiments were performed with 10 μmol of Ac-Lys(H)-O*t*Bu and variable amounts of H-Lys(Z)-O*t*Bu as indicated. Reaction conditions: 10 min at 35 °C with 20% MeCN in 0.2 M borate buffer (pH 8.7, 0.5 mL) as the reaction solvent.

Additionally, the robustness of the observed selectivity was evaluated using different ratios of H-Lys(Z)-O*t*Bu to Ac-Lys(H)-O*t*Bu (Table 2). At ratios below 4:1, acylation of H-Lys(Z)-O*t*Bu was insignificant and the side-chain-acylated lysine derivative [^18^F]**5m** was obtained in >79% RCCs. At a ratio of 4:1, acylation of the α-amino group in H-Lys(Z)-O*t*Bu became more prominent and 11% of the lysine derivative [^18^F]**5o** (for structure, see Scheme 4) was observed in the reaction mixture. At a ratio of 10:1, [^18^F]**5o** became the major product and was produced in RCCs of 25%. Based on these observations, [^18^F]AFAs could potentially be applied for selective radiolabeling of the ε-amino group (or other sterically less hindered amino groups) in peptides and proteins. In order to evaluate this assumption, we are currently investigating the acylation of insulins by [^18^F]**3e**. The preliminary results confirm that, under suitable conditions, [^18^F]AFAs can indeed be applied for site-selective radiolabeling of polypeptides at the more sterically accessible amino group. Once these investigations are concluded, a comprehensive report will be published as a separate article.

### Radiosynthesis of [^18^F]JK-PSMA-15 ([^18^F]**5p**)

**Scheme 5:**
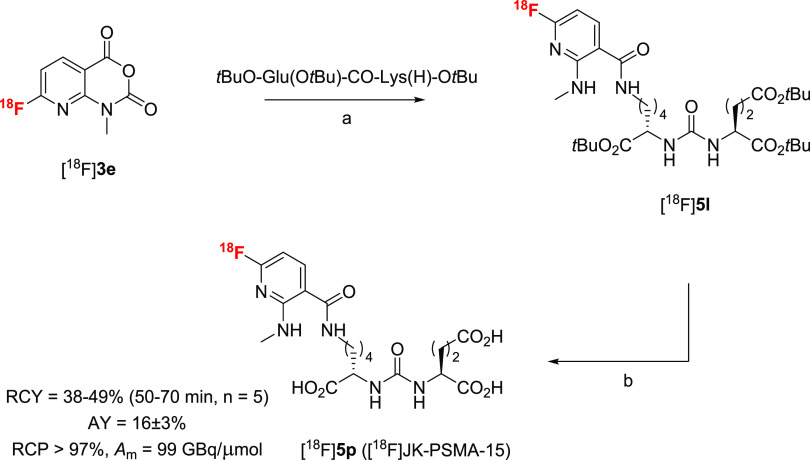
Preparation of [^18^F]**5p** ([^18^F]JK-PSMA-15) Reaction conditions: (a) 60 °C, 15 min, MeCN; (b) 60 °C, 15 min, 6 M HCl.

Having established efficient protocols for the preparation and conjugation of [^18^F]AFAs, we next evaluated their applicability for the preparation of PET tracers. Being interested in the development of novel PET tracers for PCa imaging,^10,32,33^ we applied [^18^F]**3e** as amine-reactive PG to prepare the novel PSMA radioligand [^18^F]JK-PSMA-15 (Scheme 5).

To this end, [^18^F]**3e** was allowed to react with *t*BuO-Glu(O*t*Bu)-CO-Lys(H)-O*t*Bu in MeCN for 15 min at 60 °C, furnishing the *t*Bu-protected intermediate [^18^F]**5l** in >95% RCC. Deprotection of [^18^F]**5l** was achieved by adding an equal volume of 37% HCl directly to the reaction mixture and heating for an additional 15 min at 60 °C. Finally, isolation of [^18^F]JK-PSMA-15 ([^18^F]**5p**) by semipreparative HPLC and formulation afforded the desired candidate PET tracer in AYs of 14–18% (*n* = 5) over three steps within a total synthesis time of 70–90 min at end of synthesis (EOS). Molar activity (EOS) of the probe amounted to 99 GBq/μmol (for 365 MBq [^18^F]JK-PSMA-15).

### Application of [^18^F]AFAs for Radiolabeling Using CuAAC

The operational simplicity of [^18^F]AFA production raised the question whether they might also be useful building blocks for indirect radiofluorination via Cu-catalyzed click conjugation. Therefore, we produced ^18^F-labeled *N*-propargyl- and *N*-3-azidopropyl-substituted azaisatoic anhydrides [^18^F]**3g** and [^18^F]**3h** using the “on cartridge” protocol in AYs of 52 ± 3 and 59 ± 4%, respectively.

**Scheme 6:**
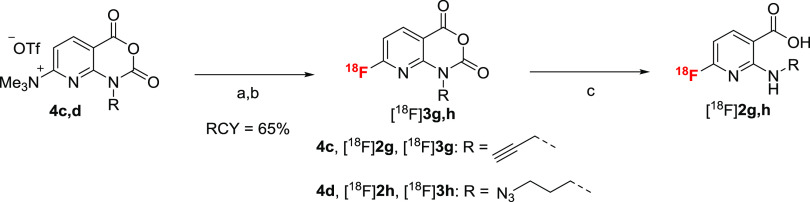
Preparation of Radiolabeled Nicotinic Acids [^18^F]**2g** and [^18^F]**2h** Reaction conditions: (a) [^18^F]F^–^-elution (from PS-HCO_3_^–^ cartridge) with a solution of the respective precursor in MeCN:*t*BuOH (1:4) over 2 min; (b) removal of unreacted precursor and [^18^F]F^–^ by solid-phase extraction (SPE); (c) 60 °C, 5 min, 10 mmol NaOH.

**Scheme 7:**
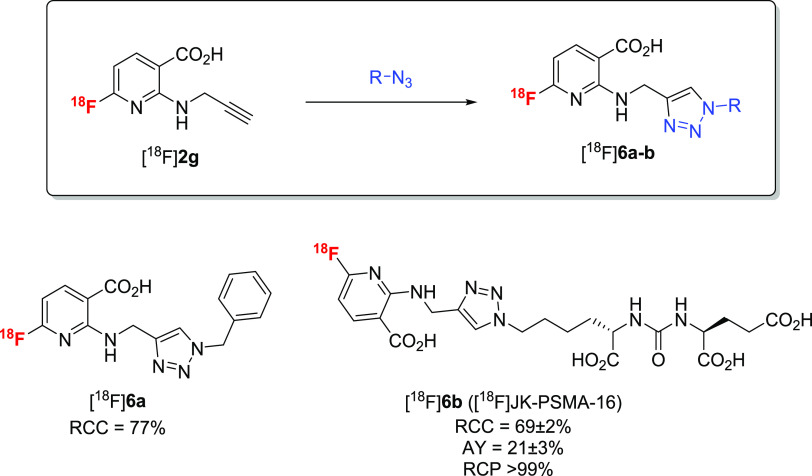
Copper(I)-Catalyzed Azide Alkyne Cycloaddition (*CuAAC*) of [^18^F]**2g** with Different Azides Reaction conditions: [^18^F]**2g** in MeCN (100 μL), 0.2 M CuSO_4_·5H_2_O (100 μL), 0.5 M l-histidine (100 μL), 1 M sodium ascorbate (100 μL), R-N_3_ (5 μmol) in MeCN (100 μL; [^18^F]**6a**) or 50% aq. MeCN (100 μL; [^18^F]**6b**), 60 °C, 15 min.

**Scheme 8:**
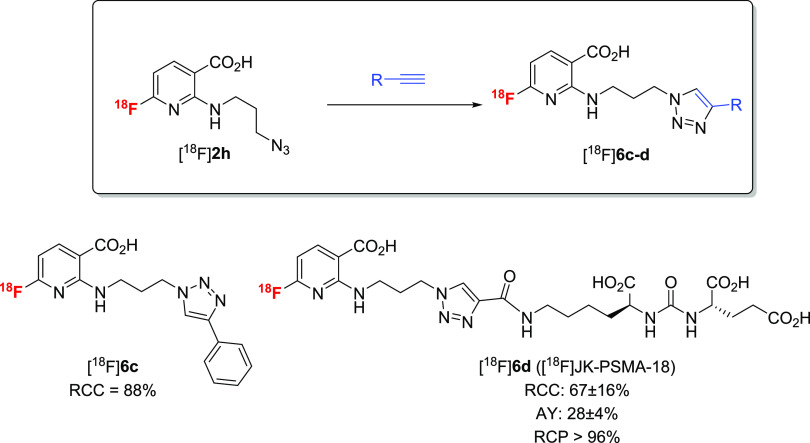
Copper(I)-Catalyzed Azide Alkyne Cycloaddition (*CuAAC*) of [^18^F]**2h** with Different Alkynes Reaction conditions: [^18^F]**2h** in MeCN (25 μL), 0.2 M CuSO_4_·5H_2_O (25 μL), l-histidine (2 mg, 13 μmol) in H_2_O (25 μL), sodium ascorbate (5 mg, 25 μmol) in H_2_O (25 μL), alkyne (5 μmol) in MeCN (100 μL; [^18^F]**6c**) or in 50% aq. MeCN (100 μL; [^18^F]**6d**), 60 °C, 15 min.

Before conjugation with different azides or alkynes, both [^18^F]AFAs were hydrolyzed into the corresponding 2-alkylamino-6-[^18^F]fluoronicotinic acids ([^18^F]**2g** and [^18^F]**2h**) using 10 mM NaOH at 60 °C for 5 min (note that almost complete hydrolysis of the *N*-propargyl-substituted [^18^F]**3g** to [^18^F]**2g** was observed already during SPE purification) (Scheme 6). The resulting solutions were directly used for Cu-mediated click cycloadditions under the reaction conditions described by Krapf et al.^34^ To this end, alkyne-substituted [^18^F]**2g** was treated with CuSO_4_ (20 μmol), l-histidine (50 μmol), sodium ascorbate (100 μmol), and benzyl azide (20 μmol) for 15 min at 60 °C to yield the desired radiolabeled triazole [^18^F]**6a** in RCCs of 77 ± 2% (*n* = 3) (Scheme 7). Under the same conditions, azido-substituted [^18^F]**2h** was reacted with phenylacetylene as a model alkyne to give [^18^F]**6c** in RCCs of 88 ± 6% (*n* = 3) (Scheme 8).

Both building blocks were also applied for the preparation of novel PSMA radioligands as candidate PET probes. For this purpose, propargyl-substituted [^18^F]**2g** was allowed to react with HO-Glu(OH)-CO-6-N_3_-Nle-OH (**7**),^34^ which furnished (after HPLC purification) [^18^F]JK-PSMA-16 in AYs of 21 ± 3% (EOS) over two steps (Scheme 7).

The “click” conjugation of [^18^F]**2h** with HO-Glu(OH)-CO-Lys(propiolyl)-OH (**8**) in turn afforded the desired radioligand [^18^F]JK-PSMA-18 in RCCs of 67 ± 16%. Semipreparative HPLC purification and formulation furnished [^18^F]JK-PSMA-18 in AYs of 28 ± 4% (EOS) over two steps with RCP >96% and a molar activity (EOS) of 75 GBq/μmol (for 630 MBq tracer) (Scheme 8).

### *In Vivo* Evaluation of the Conjugates for PSMA-Specific PET Imaging

**Figure 3:**
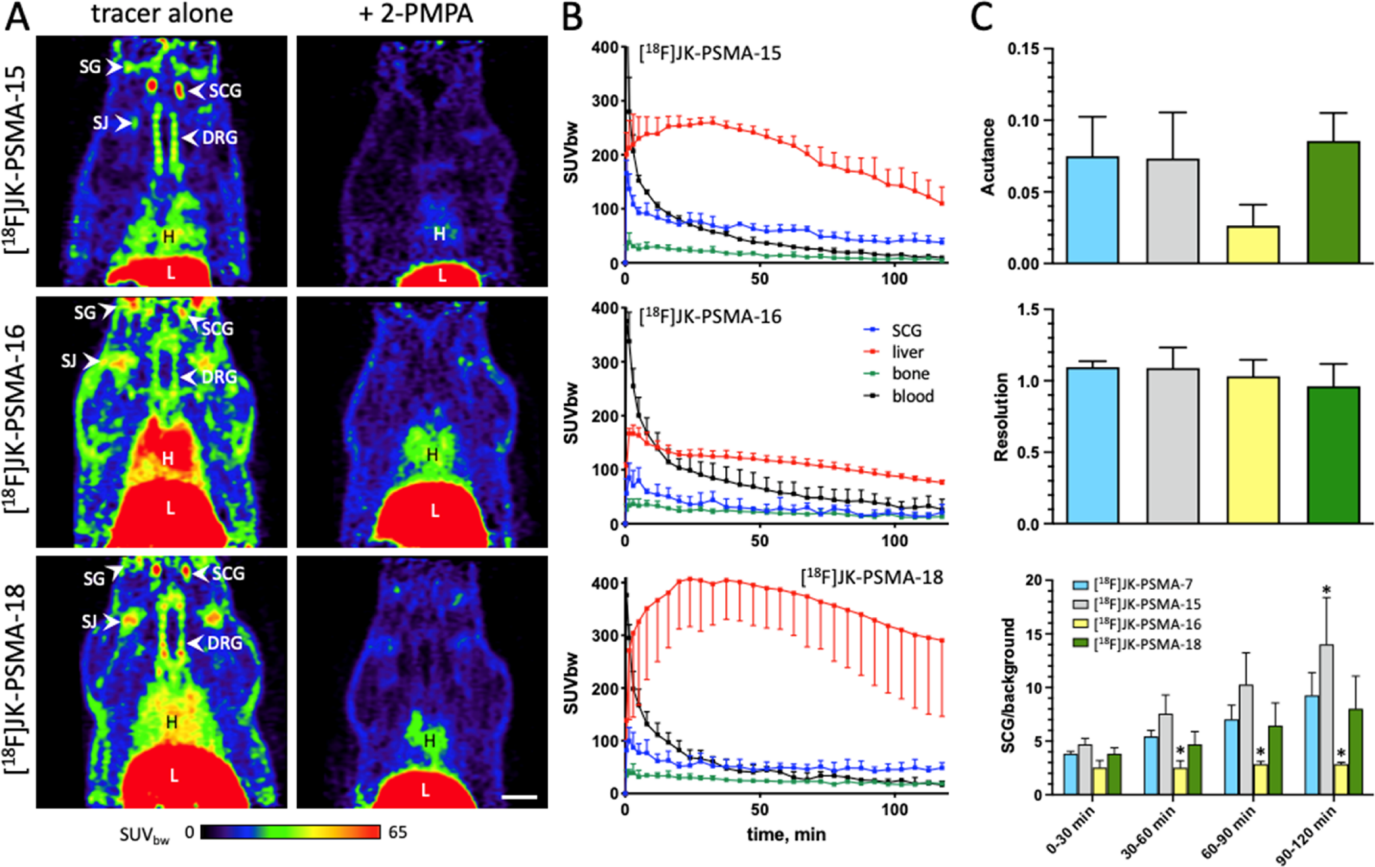
Biodistribution of [^18^F]AFA-conjugated PSMA ligands in healthy rats. (A) Horizontal PET images of the neck and thorax region with the sympathetic and dorsal root ganglia, which have a high endogenous PSMA expression. Shown are summed images (60–120 min post injection) of representative experiments. Blocking experiments (right column) were performed by co-injection of 23 mg/kg 2-PMPA. (B) Time-activity curves, calculated as mean (± standard deviation) SUV_bw_ (*n* = 3 each). (C) Image properties quantified in terms of acutance (=edge contrast, top) and resolution (=ability to distinguish two neighboring ganglia, middle) during the uptake period 60–120 min p.i., as well as the signal-to-background ratios (″signal″ extracted from the biggest ganglion SCG) during four consecutive 30 min uptake periods (bottom). * Significant differences compared to the established tracer [^18^F]JK-PSMA-7 (data from ref (10)). For full statistics and details on the analysis, see Section 7 in the Supporting Information. Abbreviations: DRG: cervical dorsal root ganglia; H: heart; L: liver; SCG: superior cervical ganglion; SG: salivary gland; SJ: shoulder joint. Scale bar: 1 cm.

The PET probes (60.3 ± 5.6 MBq) were evaluated by μPET imaging in healthy rats, using the PSMA-expressing sympathetic and dorsal root ganglia as surrogates for small PSMA-positive lesions (for details, see refs (4, 10) and Section 7 in the Supporting Information). This approach is particularly useful for the initial screening of new PSMA radioligands, as the lower inter- and intra-individual variance of tracer uptake compared to tumor models enables a more reliable comparison of multiple tracers.^4,10^ All tracers accumulated in PSMA-expressing structures, and uptake was strongly reduced by co-injection of the PSMA-inhibitor 2-PMPA (23 mg/kg) (Figure 3; for time-activity curves with 2-PMPA, see Section 7.2 in the Supporting Information). In comparison to the established tracer [^18^F]JK-PSMA-7,^10,35^ [^18^F]JK-PSMA-15 showed a significantly higher signal-to-background ratio during the uptake period of 90–120 min p.i. (Figure 3C, bottom). All other parameters for the two tracers, such as the acutance (a measure for the perceived sharpness of the image and the ability to measure the size of small PSMA-positive tissues) and the resolution (a measure for the ability to delineate two small PSMA-positive tissues located close to each other), were similar (for details on the quantification and significance of the parameters, see Section 7.1 in Supporting Information). In contrast, the signal-to-background ratio for [^18^F]JK-PSMA-16 was significantly lower when compared to [^18^F]JK-PSMA-7, which was mainly attributable to low uptake into PSMA-expressing structures and high retention in blood (see Figure 3B, middle). The acutance of [^18^F]JK-PSMA-16 was also considerably lower than that of the other tracers, although this difference did not reach statistical significance. The imaging properties of [^18^F]JK-PSMA-18 were comparable to those of [^18^F]JK-PSMA-7, although it is noteworthy that this tracer showed a high and more variable liver uptake (Figure 3B, bottom, red curve).

## Conclusions

[^18^F]AFAs are novel, easily accessible radiolabeled building blocks that enable the efficient and fast production of metabolically stable radiolabeled conjugates. Their applicability for PET tracer syntheses was confirmed by the preparation of novel PSMA radioligands via acylation or click conjugation. One of the new probes, [^18^F]JK-PSMA-15, showed promising results in the preclinical *in vivo* μPET studies and could be suitable for high-quality visualization of small PSMA-positive lesions, although further studies in appropriate tumor models may be required to corroborate these findings. Additionally, amine-reactive [^18^F]AFAs demonstrated a remarkable preference for acylation of the sterically more accessible amino group, which could be exploited for the regioselective radiolabeling of peptides and proteins.

## Experimental Section

### General Procedures

All chemicals and solvents were purchased from Aldrich (Taufkirchen, Germany), BLDPharm (Kaiserslautern, Germany), Fluorochem (Hadfield, United Kingdom), or Merck (Darmstadt, Germany) and used without further purification. Thin-layer chromatography (TLC) was performed on precoated plates of silica gel 60 F254 (Merck, Darmstadt, Germany), and the compounds were detected by UV at 254 nm and/or phosphomolybdic acid. All reactions were carried out with magnetic stirring and, if sensitive to moisture, under argon and in reaction flasks dried overnight at 140 °C prior to use. Unless noted otherwise, organic extracts were dried over anhydrous MgSO_4_. Nuclear magnetic resonance (NMR) spectra were recorded in 5% solutions at 25 °C using a Bruker Avance Neo 400 (^1^H: 400 MHz; ^13^C: 101 MHz; ^19^F: 376 MHz) or a Bruker DPX Avance 200 (^1^H: 200 MHz; ^13^C: 50 MHz). The measured chemical shifts (δ) are reported in parts per million (ppm) relative to the residual peaks of deuterated solvents. The observed signal multiplicities are characterized as follows: s = singlet, d = doublet, t = triplet, m = multiplet, dd = doublet of doublets, ddd = doublet of doublets of doublets, dt = doublet of triplets, td = triplet of doublets, dq = doublet of quartets, ddq = doublet of doublets of quartets, q = quartet, p = pentet. Coupling constants *J* are reported in hertz (Hz). High-resolution mass spectrometry (HRMS) analyses were performed using a hybrid linear ion trap FTICR mass spectrometer LTQ-FT (Thermo Fisher Scientific, Bremen, Germany) equipped with a 7 T superconducting magnet by infusion. The mass spectrometer was tuned and calibrated in the positive mode following the standard optimization procedure for all voltages and settings. Mass spectra were recorded in full scan from 200 to 1000 Da with a resolution of 100,000 at *m*/*z* 400. All data were processed using the Xcalibur software. Elemental analyses were carried out on a TCH 600 Nitrogen/Oxygen/Hydrogen and a CS 600 Carbon/Sulfur Determinator (Leco Corporation, St. Joseph, MI). Unless noted otherwise, all synthesized compounds had a purity of >95%, as determined by analytical HPLC with a Multokrom 100-5 C18 AQ column (250 mm × 4.6 mm, CS Chromatographie, Langerwehe, Germany) using a gradient from 0 to 100% MeCN over 20 min with or without addition of 0.1% TFA.

### Materials

3-Azidopropylamine,^36^ benzyl azide,^37^ Ac-Lys(H)-O*t*Bu,^38^ di-*tert*-butyl {[(*S*)-6-amino-1-(*tert*-butoxy)-1-oxohexan-2-yl]carbamoyl}-(*S*)-glutamate (*t*BuO-Glu(O*t*Bu)-CO-Lys(H)-O*t*Bu),^39^ (*S*)-2-({[(*S*)-5-azido-1-carboxypentyl]carbamoyl}amino)pentanedioic acid (**7**),^34^ 1,5-di-*tert*-butyl (*S*)-2-({[(*S*)-6-azido-1-(*tert*-butoxy)-1-oxohexan-2-yl]carbamoyl}amino)pentanedioate (**9**),^34^ and pentafluorophenyl propiolate^40^ were prepared according to known procedures. “Click” reactions were performed based on a published procedure.^41^

#### 6-Chloro-2-(methylamino)nicotinic Acid (**2a**)^26^

Methylamine (22.7 mL 33% solution in EtOH, 553 mmol, 27.0 equiv) was added to a solution of 2,6-dichloronicotinic acid (**1a**) (3.99 g, 20.8 mmol, 1.00 equiv) in anhydrous THF (5 mL) in a thick-walled glass reactor. The reactor was closed, and the reaction mixture was stirred at 80 °C for 3 days. The mixture was then concentrated under reduced pressure and the residue was taken up in MeOH (40 mL) and EtOAc (160 mL). The extract was successively washed with 1 M HCl (4 × 50 mL) and brine (100 mL), dried, and concentrated under reduced pressure to afford **2a** (3.25 g, 84% yield) as a colorless solid. ^1^H-NMR [400 MHz, (CD_3_)_2_SO]: δ 2.91 (s, 3H), 6.57 (d, *J* = 8.0 Hz, 1H), 8.02 (d, *J* = 8.0 Hz, 1H). ^13^C-NMR [101 MHz, (CD_3_)_2_SO]: δ 27.8, 106.0, 109.7, 142.8, 153.0, 158.8, 168.3. HRMS (ESI): *m*/*z* calcd for C_7_H_6_ClN_2_O_2_^–^: 185.01233, found, 185.01199 [M – H^+^].

#### 6-Chloro-2-(ethylamino)nicotinic Acid (**2b**)^42^

**2b** (68% yield, colorless solid) was prepared according to the same procedure as described for **2a** using 2 M EtNH_2_ in THF. ^1^H-NMR [400 MHz, (CD_3_)_2_SO]: δ 1.16 (t, *J* = 7.2 HZ, 3H), 3.42 (q, *J* = 7.1 Hz, 2H), 6.59 (d, *J* = 8.0 Hz, 1H), 8.03 (d, *J* = 8.0 Hz, 1H). ^13^C-NMR [101 MHz, (CD_3_)_2_SO]: δ 15.0, 35.7, 105.3, 110.4, 143.5, 153.9, 158.5, 168.7. HRMS (ESI): *m*/*z* calcd for C_8_H_8_ClN_2_O_2_^–^: 199.02798, found, 199.02794 [M – H^+^].

#### 6-Chloro-2-(prop-2-yn-1-ylamino)nicotinic Acid (**2c**)

Propargylamine (3.22 mL, 2.77 g, 50.22 mmol) was added to a solution of **1a** (2.3 g, 11.16 mmol) in anhydrous THF (5 mL) in a thick-walled glass reactor. The reactor was closed, and the reaction mixture was stirred at 50–55 °C for 4 days. The semisolid mixture was allowed to cool to ambient temperature and transferred into 1 M HCl (60 mL). The resulting precipitate was isolated by filtration, washed with H_2_O until the pH of the washings was neutral, and dried (first in air for 16 h and then at 2 mbar for 3 h). Thereafter, the precipitate was thoroughly washed with CH_2_Cl_2_:MeOH (12:1) followed by CH_2_Cl_2_ and dried, which furnished **2c** (1.6 g, 68%) as a colorless solid. The mother liquor was concentrated under reduced pressure, and the residue was purified as above to obtain a second crop of the product (0.25 g, 79% total yield). ^1^H-NMR (400 MHz, CDCl_3_): δ 11.80 (s, 1H), 8.21 (s, 1H), 7.94 (d, *J* = 8.0 Hz, 1H), 6.43 (d, *J* = 8.0 Hz, 1H), 4.16 (dd, *J* = 5.2, 2.2 Hz, 2H), 2.23–1.94 (m, 1H). ^13^C-NMR (101 MHz, CDCl_3_): δ 168.71, 157.51, 154.12, 142.59, 111.19, 105.53, 80.39, 70.81, 30.34. HRMS (ESI) calcd for C_9_H_8_ClN_2_O_2_^+^ ([M + H^+^]): 210.01906, found, 210.01889.

#### 2-[(3-Azidopropyl)amino]-6-chloronicotinic Acid (**2d**)

3-Azidopropylamine^36^ (6.2 g, 61.88 mmol) was added to a solution of **1a** (2 g, 10.42 mmol) in THF (10 mL). The resulting mixture was stirred at 45 °C for 7 days under argon and protection from light, and then concentrated under reduced pressure. The residue was taken up in EtOAc and 1 M HCl (70 mL of each), and the organic fraction was separated, washed with brine (2 × 10 mL), dried, and concentrated under reduced pressure. The residue was recrystallized from Et_2_O/pentane to afford **2d** (1.7 g, 68%) as an off-white solid. **2d** is light-sensitive and should be stored under argon and protected from light in the fridge. ^1^H-NMR (400 MHz, CDCl_3_): δ 8.13 (d, *J* = 8.1 Hz, 1H), 8.03 (t, *J* = 5.1 Hz, 1H), 6.60 (d, *J* = 8.1 Hz, 1H), 3.67 (dd, *J* = 12.5, 6.6 Hz, 2H), 3.45 (t, *J* = 6.6 Hz, 2H), 1.98 (p, *J* = 6.7 Hz, 2H). ^13^C-NMR (101 MHz, CDCl_3_): δ 172.29, 158.66, 156.54, 143.37, 111.33, 102.99, 49.36, 38.58, 28.61. Elemental Anal.: Calcd for C_9_H_10_ClN_5_O_2_: C, 42.3; H, 3.94; N, 27.4. Found: C, 42.6; H, 3.96; N, 27.0.

#### 6-Fluoro-2-(methylamino)nicotinic Acid (**2e**)

A solution of 2,6-difluoronicotinic acid (**1b**) (2 g, 12.57 mmol) in 2 M MeNH_2_ in THF (30 mL) was heated in a thick-walled glass reactor at 55–60 °C for 4 days and then concentrated under reduced pressure. The residue was taken up in H_2_O (100 mL) and filtered. The filter cake was washed with 0.5 M NaOH (2 × 10 mL), and the combined filtrate and washings were acidified to pH 2 using 0.2 M HCl in H_2_O. The resulting precipitate was isolated by filtration, washed with H_2_O until the pH of the washings was neutral, dried, and recrystallized from Et_2_O/pentane to afford **2e** (1 g, 47% yield) as an off-white solid. ^1^H-NMR [400 MHz, (CD_3_)_2_SO]: δ 2.90 (d, *J* = 4.4 Hz, 3H), 6.21 (dd, *J* = 8.3 Hz, 1H), 8.18 (d, *J* = 8.4 Hz, 1H). ^13^C-NMR [101 MHz, (CD_3_)_2_SO]: δ 168.4, 165.2 (d, *J* = 240.1 Hz), 159.4 (d, *J* = 19.4 Hz), 146.3 (d, *J* = 10.8 Hz), 104.4 (d, *J* = 3.7 Hz), 94.5 (d, *J* = 38.5 Hz), 28.16. ^19^F-NMR [376 MHz, (CD_3_)_2_SO]: δ -61.00. HRMS (ESI): *m*/*z* calcd for C_7_H_6_FN_2_O_2_^–^: 169.04188, found, 169.04154 [M – H^+^].

#### 6-Fluoro-2-(ethylamino)nicotinic Acid (**2f**)

**2f** (60% yield, off-white solid) was prepared according to the same procedure as described for **2e** but using 2 M EtNH_2_ in THF. ^1^H-NMR [400 MHz, (CD_3_)_2_SO]: δ 1.16 (t, *J* = 7.2 Hz, 3H), 3.39 (q, *J* = 7.1 Hz, 2H), 6.21 (dd, *J* = 8.3, 2.8 Hz, 1H), 8.19 (t, *J* = 8.5 Hz, 1H). ^13^C-NMR [101 MHz, (CD_3_)_2_SO]: δ 168.5, 165.2 (d, *J* = 239 Hz) 158.7 (d, *J* = 19.2 Hz), 146.4 (d, *J* = 10.1 Hz), 104.1 (d, *J* = 4.0 Hz), 94.7 (d, *J* = 39.4 Hz), 35.7, 15.0. ^19^F-NMR [376 MHz, (CD_3_)_2_SO]: δ -60.92. HRMS (ESI): *m*/*z* calcd for C_8_H_8_FN_2_O_2_^–^: 183.05753, found, 183.05753 [M – H^+^].

#### 6-Fluoro-2-(prop-2-yn-1-ylamino)nicotinic Acid (**2g**)

Propargylamine (2.4 mL, 2.09 g, 37.9 mmol) was added to a solution of **1b** (1 g, 6.29 mmol) in anhydrous THF (5 mL). The resulting mixture was stirred at 45 °C for 3 days and then concentrated under reduced pressure. The residue was taken up in EtOAc and 1 M HCl (70 mL of each), and the organic fraction was separated, washed with brine (2 × 10 mL), and dried. After concentration under reduced pressure, the crude product was purified by column chromatography (CH_2_Cl_2_/MeOH 10:1) to afford **2g** (0.8 g, 66% yield) as an off-white solid. ^1^H-NMR (400 MHz, CD_3_CN): δ 8.28 (dd, *J* = 25.0, 16.7 Hz, 2H), 6.24 (dd, *J* = 8.4, 2.7 Hz, 1H), 4.20 (dd, *J* = 5.7, 2.5 Hz, 2H), 2.44 (t, *J* = 2.5 Hz, 1H). ^13^C-NMR (101 MHz, CD_3_CN): δ 168.4, 166.3 (d, *J* = 242.1 Hz), 159.2 (d, *J* = 19.4 Hz), 147.2 (d, *J* = 10.8 Hz), 104.6 (d, *J* = 3.9 Hz), 96.7 (d, *J* = 38.5 Hz), 71.6, 31.0. ^19^F-NMR (376 MHz, CD_3_CN): δ -61.99 (s). ESI-MS: *m*/*z* 195.20 [M + H^+^]. HRMS (ESI): *m*/*z* calcd for C_9_H_8_FN_2_O_2_^+^: 195.05643, found, 195.05671 [M + H^+^].

#### 2-[(3-Azidopropyl)amino]-6-fluoronicotinic Acid (**2h**)

3-Azidopropylamine^36^ (3.77 g, 37.26 mmol) was added to a solution of **1b** (1 g, 6.29 mmol) in THF (10 mL). The resulting mixture was stirred at 45 °C for 7 days while under argon and protected from light, and then concentrated under reduced pressure. The residue was taken up in EtOAc and 1 M HCl (70 mL of each), and the organic fraction was separated, washed with brine (2 × 10 mL), dried, and concentrated under reduced pressure. The residue was purified by column chromatography (CH_2_Cl_2_/MeOH 10:1 with 0.1% AcOH) to afford **2h** (1.2 g, 95% pure according to the ^1^H-NMR spectrum, 76% yield) as a gray solid. **2h** is light-sensitive and should be stored under argon and protected from light in the fridge. ^1^H-NMR (400 MHz, CDCl_3_): δ 8.30 (t, *J* = 8.3 Hz, 1H), 8.14 (s, 1H), 6.18 (dd, *J* = 8.4, 2.8 Hz, 1H), 3.76–3.62 (m, 2H), 3.45 (t, *J* = 6.6 Hz, 2H), 1.97 (p, *J* = 6.7 Hz, 2H). ^13^C-NMR (101 MHz, CDCl_3_): δ 172.0, 165.5 (d, *J* = 241.2 Hz), 159.2 (d, *J* = 19.6 Hz), 146.1 (d, *J* = 11.0 Hz), 102.9 (d, *J* = 3.8 Hz), 94.7 (d, *J* = 39.1 Hz), 77.4, 77.0, 76.7, 49.3, 38.5, 28.6. 63. ^19^F-NMR (376 MHz, CDCl_3_): δ -57.12. ESI-MS: *m*/*z* 240.14 [M + H^+^]. HRMS (ESI): *m*/*z* calcd for C_9_H_11_FN_5_O_2_^+^: 240.08913, found, 240.08945 [M + H^+^].

#### 7-Chloro-1-methyl-2*H*-pyrido[2,3-*d*][1,3]oxazine-2,4(1*H*)-dione (**3a**)

Triphosgene (160 mg, 0.53 mmol, 0.6 equiv) was added in a stream of argon to a solution of 6-chloro-2-(methylamino)nicotinic acid (**2a**) (170 mg, 0.91 mmol, 1.0 equiv) in anhydrous dioxane (30 mL). The reaction mixture was refluxed for 3 days and concentrated under reduced pressure. The residue was purified by flash column chromatography (silica, petrol ether/EtOAc) to afford **3a** (155 mg, 80% yield) as a colorless solid. ^1^H-NMR (400 MHz, CDCl_3_): δ 3.46 (s, 3H), 7.46 (d, *J* = 8.1 Hz, 1H), 8.40 (d, *J* = 8.1 Hz, 1H). ^13^C-NMR (101 MHz, CDCl_3_): δ 30.3, 107.2, 119.6, 141.6, 147.5, 152.8, 157.5, 168.0. Elemental Anal.: Calcd for C_8_H_5_ClN_2_O_3_: C, 45.2; H, 2.37; N, 13.2. Found: C, 45.4; H, 2.09; N, 12.9. MS (ESI): *m*/*z* calcd for C_8_H_6_ClN_2_O_3_^+^: 213.59, found, 213.11 [M + H^+^].

#### 7-Chloro-1-ethyl-2*H*-pyrido[2,3-*d*][1,3]oxazine-2,4(1*H*)-dione (**3b**)

(43)**3b** (82% yield, colorless solid) was prepared from 6-chloro-2-(ethylamino)nicotinic acid (**2b**) according to the same procedure as described for **3a**. ^1^H-NMR [400 MHz, (CD_3_)_2_SO]: δ 1.24 (t, *J* = 7.1, 3H, H-1), 4.11 (q, *J* = 7.1 Hz, 2H), 7.46 (d, *J* = 8.1 Hz, 1H), 8.40 (d, *J* = 8.1 Hz, 1H). ^13^C-NMR [101 MHz, (CD_3_)_2_SO]: δ 12.6, 39.2, 107.9, 120.1, 142.2, 147.5,152.6, 155.9, 158.1. Elemental Anal.: Calcd for C_9_H_7_ClN_2_O_3_: C, 47.7; H, 3.11; N, 12.4. Found: C, 47.6; H, 3.33; N, 12.0. MS(ESI): *m*/*z* calcd for C_9_H_8_ClN_2_O_3_^+^: 227.02, found, 227.15 [M + H^+^].

#### 7-Chloro-1-(prop-2-yn-1-yl)-2*H*-benzo[*d*][1,3]oxazine-2,4(1*H*)-dione (**3c**)

Triphosgene (1.37 g, 4.6 mmol) was added to a suspension of **2c** (1 g, 5.15 mmol) in anhydrous dioxane (10 mL), and the resulting mixture was stirred at 60 °C for 3 days. The mixture was concentrated under reduced pressure, and the residue was triturated with H_2_O, after which the resulting precipitate was isolated by filtration and thoroughly dried. The solid was taken up in boiling EtOAc, filtered while still hot, and the filtrate was diluted with boiling hexane. The resulting solution was allowed to cool to ambient temperature and then cooled further in a water/ice bath. The resulting precipitate was isolated by filtration to afford **3c** (1.15 g, 63% yield, >95% HPLC purity) as an off-white solid. ^1^H-NMR (400 MHz, CD_3_CN): δ 8.4 (d, *J* = 8.1 Hz, 1H), 7.4 (d, *J* = 8.1 Hz, 1H), 4.9 (d, *J* = 2.5 Hz, 2H), 2.6 (t, *J* = 2.5 Hz, 1H). ^13^C-NMR [101 MHz, (CD_3_)_2_SO]: δ 157.1, 155.3, 151.3, 146.8, 141.8, 120.3, 107.6, 77.8, 74.7, 33.0. Elemental Anal.: Calcd for C_10_H_5_ClN_2_O_3_: C, 50.8; H, 2.13; N, 11.8. Found: C, 50.5; H, 1.98; N, 11.6.

#### 1-(3-Azidopropyl)-7-chloro-2*H*-pyrido[2,3-*d*][1,3]oxazine-2,4(1*H*)-dione (**3d**)

Triphosgene (148 mg, 0.5 mmol) was added to a solution of 2-[(3-azidopropyl)amino]-6-chloronicotinic acid (**2d**) (255 g, 0.1 mmol) in anhydrous dioxane (3 mL), and the resulting mixture was stirred at 60 °C for 3 days while protected from light. The mixture was concentrated under reduced pressure, and the residue was recrystallized from EtOAc/hexane to afford **3d** (241 mg, 86% yield) as an off-white solid. ^1^H-NMR (400 MHz, CDCl_3_): δ 8.36 (d, *J* = 8.1 Hz, 1H), 7.46–7.14 (m, 1H), 4.38 (t, *J* = 6.9 Hz, 2H), 3.49 (t, *J* = 6.4 Hz, 2H), 2.09 (t, *J* = 6.7 Hz, 2H). ^13^C-NMR (101 MHz, CDCl_3_): δ 158.40, 156.68, 152.27, 147.06, 141.69, 120.63, 105.73, 49.04, 41.93, 26.82. Elemental Anal.: Calcd for C_10_H_8_ClN_5_O_3_: C, 42.6; H, 2.86; N, 24.9. Found: C, 42.6; H, 2.88; N, 24.8. MS(ESI): *m*/*z* calcd for C_10_H_9_ClN_5_O_3_^+^: 282.03, found, 282.10 [M + H^+^].

#### 7-Fluoro-1-methyl-2*H*-pyrido[2,3-*d*][1,3]oxazine-2,4(1*H*)-dione (**3e**)

**3e** (58% yield, colorless solid) was prepared from **2e** according to the same procedure as described for **3a**. ^1^H-NMR [400 MHz, (CD_3_)_2_SO]: δ 3.45 (s, 3H), 7.14 (dd, *J* = 8.4, 2.2 Hz, 1H), 8.58 (dd, *J* = 8.3, 7.7 Hz, 1H). ^13^C-NMR [101 MHz, (CD_3_)_2_SO]: δ 165.4 (d, *J* = 248.5 Hz), 164.2, 157.2, 152.9 (d, *J* = 18.2 Hz), 147.7, 145.0 (d, *J* = 11.1 Hz) 106.0 (d, *J* = 4.0 Hz), 104.9 (d, *J* = 37.4 Hz), 30.3. ^19^F-NMR [376 MHz, (CD_3_)_2_SO]: δ −56.82. HRMS (ESI): *m*/*z* calcd for C_8_H_5_FN_2_O_3_^+^: 196.02787, found, 196.02770 [M + H^+^].

#### 1-Ethyl-7-fluoro-2*H*-pyrido[2,3-*d*][1,3]oxazine-2,4(1*H*)-dione (**3f**)

**3f** (62% yield, colorless solid) was prepared from **2f** according to the same procedure as described for **3b**. ^1^H-NMR [400 MHz, (CD_3_)_2_SO]: δ 1.24 (t, *J* = 7.1 Hz, 3H), 4.08 (q, *J* = 7.1 Hz, 2H), 7.13 (dd, *J* = 8.4, 2.1 Hz, 1H), 8.57 (t, *J* = 8.0 Hz, 1H). ^13^C-NMR [101 MHz, (CD_3_)_2_SO]: δ 166.0 (d, *J* = 248.4 Hz), 157.7, 152.8 (d, *J* = 18.2 Hz), 147. 7, 145.5 (d, *J* = 11.1 Hz), 106.7 (d, *J* = 3.8 Hz), 105.4 (d, *J* = 37.1 Hz), 49.1, 40.6, 40.4, 40.2, 39.9, 39.8, 39.6, 39.4, 39.3, 12.6. HRMS (ESI): *m*/*z* calcd for C_9_H_7_FN_2_O_3_^+^: 210.04352, found, 210.04337 [M + H^+^].

#### 7-Fluoro-1-(prop-2-yn-1-yl)-2*H*-pyrido[2,3-*d*][1,3]oxazine-2,4(1*H*)-dione (**3g**)

Triphosgene (0.75 g, 2.53 mmol) was added to a solution of 6-fluoro-2-(prop-2-yn-1-ylamino)nicotinic acid (**2g**) (1.5 g, 7.67 mmol) in anhydrous dioxane (10 mL), and the resulting mixture was stirred at 60 °C for 3 days. The mixture was concentrated under reduced pressure, and the residue was purified by column chromatography (EtOAc:hexane 1:1.8) to afford **3g** (0.74 g, 44% yield) as an off-white solid. ^1^H-NMR (400 MHz, CDCl_3_): δ 8.53 (dd, *J* = 8.4, 7.2 Hz, 1H), 6.91 (dd, *J* = 8.4, 2.4 Hz, 1H), 4.96 (d, *J* = 2.5 Hz, 2H), 2.27 (t, *J* = 2.5 Hz, 1H). ^13^C-NMR (101 MHz, CDCl_3_): δ 166.7 (d, *J* = 256.5 Hz), 155.9, 151.8 (d, *J* = 18.2 Hz), 146.51, 145.3 (d, *J* = 10.1 Hz), 106.4 (d, *J* = 37.4 Hz), 104.9 (d, *J* = 3.8 Hz), 72.7, 33.4. ^19^F-NMR (376 MHz, CDCl_3_): δ −52.35. Elemental Anal.: Calcd for C_10_H_5_FN_2_O_3_: C, 54.56, H, 2.29; N, 12.72. Found: C, 53.97; H, 2.46; N, 12.47. HRMS (ESI): *m*/*z* calcd for C_10_H_5_FN_2_O_3_^+^: 220.02787, found, 220.02779 [M + H^+^].

#### 1-(3-Azidopropyl)-7-fluoro-2*H*-pyrido[2,3-*d*][1,3]oxazine-2,4(1*H*)-dione (**3h**)

**3h** (83% yield, off-white solid) was prepared from **2h** according to the same procedure as described for **3d**. ^1^H-NMR (400 MHz, CDCl_3_): δ 8.53 (dd, *J* = 8.4, 7.3 Hz, 1H), 6.90 (dd, *J* = 8.4, 2.4 Hz, 1H), 4.51–4.28 (m, 2H), 3.48 (t, *J* = 6.6 Hz, 2H), 2.23–1.89 (m, 2H). ^13^C-NMR (101 MHz, CDCl_3_): δ 166.7 (d, *J* = 254.5 Hz), 156.3, 152.7 (d, *J* = 17.9 Hz), 147.2, 145.2 (d, *J* = 11.0 Hz), 106.0 (d, *J* = 36.5 Hz), 104.9 (d, *J* = 4.1 Hz), 49.0, 42.0, 26.8. ^19^F-NMR (376 MHz, CDCl_3_): δ -52.44. Elemental Anal.: Calcd for C_10_H_8_FN_5_O_3_: C, 45.29; H, 3.04; N, 26.41. Found: C, 45.15; H, 3.09; S, 7.66; N, 26.12. MS(ESI): *m*/*z* calcd for C_10_H_9_FN_5_O_3_^+^: 265.06, found, 266.12 [M + H^+^].

#### *N,N,N*,1-Tetramethyl-2,4-dioxo-1,4-dihydro-2*H*-pyrido[2,3-*d*][1,3]oxazin-7-aminium Triflate (**4a**)

2 M Me_3_N in THF (14.8 mL, 29.63 mmol, dried with CaH_2_ for at least 24 h and stored under argon) was added to a solution of **3a** (2.1 g, 9.88 mmol) in anhydrous THF (20 mL), and the resulting white suspension was stirred for 1 h. All volatiles were removed under partial vacuum in a stream of argon, and the residue (chloride salt) was dried at 2 mbar and ambient temperature for 10 min. TMSOTf (3.31 ml, 4.06 g, 18.27 mmol) from a freshly opened glass ampule (Cat. Nr. 225649, Sigma-Aldrich/Merck, Darmstadt, Germany) was added to a suspension of the dried crude chloride salt in anhydrous CH_2_Cl_2_ (30 mL), and the reaction mixture was stirred for 40 min. That followed, all volatiles were removed under partial vacuum in a stream of argon, the residue was dried at 2 mbar and ambient temperature for 10 min and then triturated with Et_2_O. The resulting precipitate was isolated by filtration, washed with Et_2_O, and taken up in EtOAc (50 mL). The suspension was stirred under reflux for 10 min, cooled to 4 °C, and filtered. The filter cake was washed with EtOAc followed by Et_2_O and dried to afford **4a** (3.69 g, 97% yield) as a colorless solid. ^1^H-NMR [400 MHz, (CD_3_)_2_SO]: δ 3,56 (s, 3H), 3.66 (s, 9H), 7.93 (d, *J* = 8.4 Hz, 1H), 8.80 (d, *J* = 8.4 Hz, 1H). ^13^C-NMR [101 MHz, (CD_3_)_2_SO]: δ 30.9, 55.1, 110.6, 110.7, 144.2, 147.9, 152.2, 157.4, 160.0. ^19^F-NMR [376 MHz, (CD_3_)_2_SO]: δ -77.76. HRMS (ESI): *m*/*z* calcd for C_10_H_11_F_3_N_3_O_3_^+^: 221.07926, found, 221.07949 [M – CH_3_] (in HRMS, loss of CH_3_ was observed for several anhydrides). ESI-MS: *m*/*z* calcd for C_11_H_14_N_3_O_3_^+^: 236.10, found, 236.20 [M^+^].

#### 1-Ethyl*-N,N,N*-trimethyl-2,4-dioxo-1,4-dihydro-2*H*-pyrido[2,3-*d*][1,3]oxazin-7-aminium Triflate (**4b**)

**3b** (0.67 g, 2.96 mmol) was added to 2 M Me_3_N in THF (12.6 mL, 25.2 mmol), and the resulting suspension was stirred for 1 h. The mixture was concentrated under reduced pressure, the residue was suspended in CH_2_Cl_2_ (5 mL), filtered, and washed successively with Et_2_O and *n*-pentane (20 mL of each). Drying at 2 mbar and ambient temperature afforded 0.8 g (94%) of the moisture-sensitive chloride salt, which was directly converted into the triflate by treatment with TMSOTf (1.10 mL, 6.30 mmol) in anhydrous CH_2_Cl_2_ (12 mL) for 40 min under stirring at ambient temperature. After removal of volatiles under reduced pressure, the residue was washed successively with Et_2_O and EtOAc and dried at 2 mbar and ambient temperature, which afforded **4b** (0.8 g, 68% yield) as a colorless solid. ^1^H-NMR [400 MHz, (CD_3_)_2_SO]: δ 1.28 (t, *J* = 7.0 Hz, 3H), 3.66 (s, 9H), 4.21 (q, *J* = 7.0, 2H), 7.93 (d, *J* = 8.4, 1H), 8.79 (d, *J* = 8.4, 1H). ^13^C-NMR [101 MHz, (CD_3_)_2_SO]: δ 12.6, 39.5, 55.07 (C-10), 110.6, 110.8, 144.2, 147.4, 152.3, 157.4, 160.1. ^19^F-NMR [376 MHz, (CD_3_)_2_SO]: δ -77.76. HRMS (ESI): *m*/*z* calcd for C_11_H_13_F_3_N_3_O_3_^+^: 235.09514, found, 235.09500 [M – CH_3_] (in HRMS, loss of CH_3_ was observed for several anhydrides). ESI-MS: *m*/*z* calcd for C_12_H_16_N_3_O_3_^+^: 250.12, found, 250.22 [M^+^].

#### *N,N,N*-Trimethyl-2,4-dioxo-1-(prop-2-yn-1-yl)-1,4-dihydro-2*H*-pyrido[2,3-*d*][1,3]oxazin-7-aminium Triflate (**4c**)

**4c** (97% yield, colorless solid) was prepared from **3c** according to the same procedure as described for **4a**. ^1^H-NMR (400 MHz, CD_3_CN): δ 8.7 (d, *J* = 8.4 Hz, 1H), 7.8 (d, *J* = 8.4 Hz, 1H), 5.0 (d, *J* = 2.5 Hz, 2H), 3.6 (s, 9H), 2.6 (t, *J* = 2.5 Hz, 1H). ^13^C-NMR (101 MHz, CD_3_CN): δ 160.6, 157.1, 152.0, 147.6, 145.6, 111.6, 111.5, 77.8, 73.9, 56.2, 34.7. ^19^F-NMR (376 MHz, CD_3_CN): δ -79.3. Elemental Anal.: Calcd for C_14_H_14_F_3_N_3_O_6_S: C, 41.08; H, 3.45; N, 10.27; S, 7.83. Found: C, 40.77; H, 3.59; N, 9.88; S, 7.66.

#### *N*,*N*,*N*-Trimethyl-2,4-dioxo-1-(3-azidopropyl)-1,4-dihydro-2*H*-pyrido[2,3-*d*][1,3]oxazin-7-aminium Triflate (**4d**)

**4d** (94% yield, colorless solid) was prepared from **3d** according to the same procedure as described for **4a**. ^1^H-NMR [400 MHz, (CD_3_)_2_SO]: δ 8.81 (d, *J* = 8.4 Hz, 1H), 7.94 (d, *J* = 8.4 Hz, 1H), 4.25 (t, *J* = 6.8 Hz, 2H), 3.66 (s, 9H), 3.50 (t, *J* = 6.9 Hz, 2H), 1.96 (p, *J* = 6.9 Hz, 2H). ^13^C-NMR [101 MHz, (CD_3_)_2_SO]: δ 160.03, 157.38, 151.72, 147.65, 144.24, 144.24, 110.85, 110.78, 55.09, 48.64, 41.63, 26.70. ^19^F-NMR [376 MHz, (CD_3_)_2_SO]: δ -77.0. HRMS (ESI): *m*/*z* calcd for C_13_H_17_N_6_O_6_^+^: 477.07746, found; 477.07704 [M^+^].

#### *N*-Butyl-6-fluoro-2-(methylamino)nicotinamide (**5a**)

A solution of *n*-butylamine (0.2 mmol, 2 equiv) in MeCN (500 μL) was added to **3e** (0.1 mmol), and the reaction mixture was stirred at 50 °C for 30 min. The product was isolated by flash column chromatography (silica, CH_2_Cl_2_/MeOH) to afford **5a** (18.6 mg, 82% yield) as a colorless solid. ^1^H-NMR (400 MHz, CDCl_3_): δ 8.41 (s, 1H), 7.64 (t, *J* = 8.1 Hz, 1H), 6.02 (dd, *J* = 8.2, 2.9 Hz, 1H), 3.39 (td, *J* = 7.2, 5.7 Hz, 2H), 2.99 (d, *J* = 4.9 Hz, 3H), 1.69–1.48 (m, 2H), 1.47–1.28 (m, 3H), 1.05–0.82 (m, 5H). ^13^C-NMR (101 MHz, CDCl_3_): δ 167.6, 164.7 (d, *J* = 241.2 Hz), 158.9 (d, *J* = 19.3 Hz), 139.7 (d, *J* = 10.2 Hz), 107.0 (d, *J* = 4.2 Hz), 93.3 (d, *J* = 38.7 Hz), 39.6, 31.7, 27.9, 20.2, 13.8. ^19^F-NMR (376 MHz, CDCl_3_): δ -62.83. HRMS (ESI): *m*/*z* calcd for C_11_H_17_FN_3_O^+^: 226.13502, found, 226.13524 [M + H^+^].

#### *N*-Butyl-2-(ethylamino)-6-fluoronicotinamide (**5b**)

**5b** (71% yield, colorless solid) was prepared from **3f** and *n*-butylamine according to the same procedure as described for **5a**. ^1^H-NMR (400 MHz, CDCl_3_): δ 7.63 (t, *J* = 8.1 Hz, 1H), 6.02 (dd, *J* = 8.2, 3.0 Hz, 1H), 5.91 (s, 1H), 3.60–3.17 (m, 4H), 1.84–1.53 (m, 2H), 1.53–1.15 (m, 5H), 1.08–0.92 (m, 3H). ^13^C-NMR (101 MHz, CDCl_3_): δ 167.7, 164.8 (d, *J* = 240.1 Hz), 158.2 (d, *J* = 19.1 Hz), 139.8 (d, *J* = 10.2 Hz), 106.7 (d, *J* = 3.9 Hz), 93.5 (d, *J* = 38.9 Hz), 39.7, 35.9, 31.8, 29.8, 20.3, 14.8, 13.9. ^19^F-NMR (376 MHz, CDCl_3_): δ -62.62. HRMS (ESI): *m*/*z* calcd for C_12_H_19_FN_3_O^+^: 240.15068, found, 240.15085 [M + H^+^].

#### Methyl 6-[6-fluoro-2-(methylamino)nicotinamido]hexanoate (**5c**)

A solution of 6-fluoro-2-(methylamino)nicotinic acid (**2e**) (0.6 g, 3.53 mmol) in SOCl_2_ (0.64 mL, 1.05 g, 14.08 mmol) was stirred for 1 h and then concentrated under reduced pressure. The residue was taken up in anhydrous toluene (20 mL), and the resulting solution was concentrated under reduced pressure (×3) to afford the corresponding chloroanhydride, which was dissolved in anhydrous CH_2_Cl_2_ (10 mL). Methyl 6-aminohexanoate hydrochloride (1.9 g, 10.45 mmol) followed by DIEA (1.6 mL, 1.19 g, 9.19 mmol) were added to the solution and the reaction mixture was stirred for 3 h, and then concentrated under reduced pressure. The residue was taken up in Et_2_O and 1 M HCl (50 mL of each). The organic fraction was separated; successively washed with 1 M HCl (3 × 10 mL), H_2_O (3 × 10 mL), 5% NaHCO_3_ (3 × 10 mL), and brine (2 × 10 mL); dried; and concentrated under reduced pressure. The residue was recrystallized from pentane (low-temperature recrystallization) to afford **5c** (0.67 g, 64% yield) as a colorless solid. ^1^H-NMR (400 MHz, CDCl_3_): δ 8.42 (s, 1H), 7.68 (t, *J* = 8.1 Hz, 1H), 6.16 (s, 1H), 6.02 (dd, *J* = 8.2, 2.9 Hz, 1H), 3.68 (s, 3H), 3.40 (td, *J* = 7.1, 5.6 Hz, 2H), 2.99 (s, 3H), 2.35 (t, *J* = 7.3 Hz, 2H), 1.74–1.54 (m, 4H), 1.45–1.35 (m, 2H). ^13^C-NMR (101 MHz, CDCl_3_): δ 174.1, 167.6, 164.7 (d, *J* = 241.1 Hz), 159.0 (d, *J* = 19.2 Hz), 139.8 (d, *J* = 10.2 Hz), 106.9 (d, *J* = 4.2 Hz), 93.3 (d, *J* = 38.8 Hz), 51.6, 39.4, 33.7, 29.1, 27.8, 26.3, 24.2. ^19^F-NMR (376 MHz, CDCl_3_): δ -62.80. ESI-MS: *m*/*z* 298.32 [M + H^+^], 320.31 [M + Na^+^], 336.28 [M + K^+^]. HRMS (ESI): *m*/*z* calcd for C_14_H_22_FN_3_O_3_Na^+^: 320.1386, found, 320.1386 [M + Na^+^].

#### Methyl 6-[6-fluoro-2-(ethylamino)nicotinamido]hexanoate (**5d**)

**5d** (72% yield; colorless solid) was prepared from **2f** and methyl 6-aminohexanoate hydrochloride according to the same procedure as described for **5c**. ^1^H-NMR (400 MHz, CDCl_3_): δ 8.42 (s, 1H), 7.68 (t, *J* = 8.1 Hz, 1H), 6.15 (s, 1H), 6.01 (dd, *J* = 8.2, 3.0 Hz, 1H), 3.68 (s, 3H), 3.49–3.31 (m, 4H), 2.35 (t, *J* = 7.3 Hz, 2H), 1.79–1.53 (m, 4H), 1.50–1.36 (m, 2H), 1.25 (t, *J* = 7.3 Hz, 3H). ^13^C-NMR (101 MHz, CDCl_3_): δ 174.1, 167.6, 164.6 (d, *J* = 241.0 Hz), 158.2 (d, *J* = 19.0 Hz), 139.9 (d, *J* = 10.2 Hz), 106.6 (d, *J* = 4.4 Hz), 93.3 (d, *J* = 38.8 Hz), 51.6, 39.4, 35.8, 33.8, 29.1, 26.3, 24.2, 14.7. ^19^F-NMR (376 MHz, CDCl_3_): δ -62.66. ESI-MS: *m*/*z* 312.35 [M + H^+^], 334.31 [M + Na^+^], 350.27 [M + K^+^]. HRMS (ESI): *m*/*z* calcd for C_15_H_24_FN_3_O_3_Na^+^: 334. 1543, found, 334.1544 [M + Na^+^].

#### *N*-Benzyl-6-fluoro-2-(methylamino)nicotinamide (**5e**)

A solution of benzylamine (0.2 mmol, 2 equiv) in MeCN (500 μL) was added to **3e** (0.1 mmol), and the reaction mixture was stirred at 80 °C for 30 min. The product was isolated by flash column chromatography (silica, CH_2_Cl_2_/MeOH) to afford **5e** (25.2 mg, 92% yield) as a colorless solid. ^1^H-NMR (400 MHz, CDCl_3_): δ 8.47 (s, 1H, NH), 7.50–7.18 (m, 5H), 6.24 (s, 1H, NH), 6.02 (dd, *J* = 8.2, 2.9 Hz, 1H), 4.59 (d, *J* = 5.6 Hz, 2H), 3.02 (s, 3H). ^13^C-NMR (101 MHz, CDCl_3_): δ 167.4, 164.8 (d, *J* = 241.6 Hz), 158.9 (d, *J* = 10.2 Hz), 139.9 (d, *J* = 10.2 Hz), 137.9, 128.9, 127.9, 127.8, 106.5 (d, *J* = 6.2 Hz), 103.6, 93.5 (d, *J* = 38.9 Hz), 43.89, 27.89. ^19^F-NMR (376 MHz, CDCl_3_): δ -62.17. HRMS (ESI): *m*/*z* calcd for C_14_H_14_FN_3_O^+^: 260.119367, found, 260.11940 [M + H^+^].

#### *N*-Benzyl-2-(ethylamino)-6-fluoronicotinamide (**5f**)

**5f** (74% yield, colorless solid) was prepared from **3f** and benzylamine according to the same procedure as described for **5e**. ^1^H-NMR (400 MHz, CDCl_3_): δ 8.49 (s, 1H), 7.67 (t, *J* = 8.1 Hz, 1H), 7.44–7.26 (m, 5H), 6.33 (s, 1H), 6.00 (dd, *J* = 8.2, 3.0 Hz, 1H), 4.58 (d, *J* = 5.5 Hz, 2H), 3.50 (q, *J* = 7.2 Hz, 2H), 1.26 (t, *J* = 7.2 Hz, 3H). ^13^C-NMR (101 MHz, CDCl_3_): δ 167.4, 164.7 (d, *J* = 241.4 Hz), 158.3 (d, *J* = 19.2 Hz), 140.0 (d, *J* = 10.4 Hz), 137.9, 128.9, 127.8, 127.7, 106.1 (d, *J* = 4.3 Hz), 93.5 (d, *J* = 39.0 Hz), 43.8, 35.9, 14.7. ^19^F-NMR (376 MHz, CDCl_3_): δ -62.11. HRMS (ESI): *m*/*z* calcd for C_15_H_17_FN_3_O^+^: 274.13502, found, 274.13508 [M + H^+^].

#### Methyl [6-fluoro-2-(methylamino)nicotinoyl]phenylalaninate (**5g**)

**5g** (41% yield, colorless solid) was prepared from **3e** and HCl·H-Phe-OMe according to the same procedure as described for **5a**. ^1^H-NMR [400 MHz, (CD_3_)_2_SO]: δ 8.80 (d, *J* = 7.6 Hz, 1H), 8.44 (q, *J* = 5.0 Hz, 1H), 8.12 (t, *J* = 8.2 Hz, 1H), 7.28 (d, *J* = 4.3 Hz, 4H), 7.21 (q, *J* = 4.3 Hz, 1H), 6.24 (dd, *J* = 8.2, 2.7 Hz, 1H), 4.60 (ddd, *J* = 10.0, 7.4, 5.3 Hz, 1H), 3.64 (s, 3H), 3.20–3.00 (m, 2H), 2.82 (d, *J* = 4.7 Hz, 3H). ^13^C-NMR [101 MHz, (CD_3_)_2_SO]: δ 172.6, 167.4, 164.3 (d, *J* = 238.5 Hz), 158.7 (d, *J* = 19.1 Hz), 142.8 (d, *J* = 10.2 Hz), 138.1, 129.5, 128.7, 127.0, 106.7 (d, *J* = 4.0 Hz), 93.5 (d, *J* = 38.5 Hz), 54.6, 52.5, 36.6, 28.1. ^19^F-NMR [376 MHz, (CD_3_)_2_SO]: δ -63.13. HRMS (ESI): *m*/*z* calcd for C_17_H_19_FN_3_O_3_^+^: 332.14050, found, 332.14084 [M + H^+^].

#### Methyl [2-(ethylamino)-6-fluoronicotinoyl]phenylalaninate (**5h**)

**5h** (27% yield, colorless solid) was prepared from **3f** and HCl·H-Phe-OMe according to the same procedure as described for **5a**. ^1^H-NMR (400 MHz, CDCl_3_): δ 8.34 (s, 1H), 7.57 (t, *J* = 8.1 Hz, 1H), 7.35–7.24 (m, 3H), 7.20–7.08 (m, 2H), 6.38 (d, *J* = 7.4 Hz, 1H), 6.01 (dd, *J* = 8.2, 3.0 Hz, 1H), 5.00 (dt, *J* = 7.4, 5.7 Hz, 1H), 3.80 (s, 3H), 3.47 (q, *J* = 7.2 Hz, 2H), 3.23 (qd, *J* = 13.9, 5.8 Hz, 2H), 1.26 (t, *J* = 7.3 Hz, 3H). ^13^C-NMR (101 MHz, CDCl_3_): δ 172.1, 166.9, 164.8 (d, *J* = 241.8 Hz), 158.2 (d, *J* = 19.3 Hz), 140.4 (d, *J* = 10.5 Hz), 135.7, 129.3, 128.7, 127.3, 105.7 (d, *J* = 4.2 Hz), 93.8 (d, *J* = 39.0 Hz), 53.3, 52.6, 37.9, 35.8, 14.7. ^19^F-NMR (376 MHz, CDCl_3_): δ -61.58. HRMS (ESI): *m*/*z* calcd for C_18_H_21_FN_3_O_3_^+^: 346.15614, found, 346.15654 [M + H^+^].

#### *N*-*tert*-Butyl-6-fluoro-2-(methylamino)nicotinamide (**5i**)

**5i** (57% yield, colorless solid) was prepared from **3e** and *N*-*tert*-butylamine according to the same procedure as described for **5e**. ^1^H-NMR (400 MHz, CDCl_3_): δ 8.24 (s, 1H, NH), 7.46 (td, *J* = 8.1, 1.2 Hz, 1H), 5.94–5.85 (m, 1H), 5.61 (s, 1H, NH), 2.87 (s, 3H), 1.34 (d, *J* = 1.3 Hz, 9H). ^13^C-NMR (101 MHz, CDCl_3_): δ 167.4, 163.8 (d, *J* = 237.3 Hz), 157.9 (d, *J* = 18.8 Hz), 143.0 (d, *J* = 9.8 Hz), 108.5 (d, *J* = 4.2 Hz), 93.1 (d, *J* = 38.1 Hz), 51.5, 35.6, 29.0, 15.1. ^19^F-NMR (376 MHz, CDCl_3_): δ -63.42. HRMS (ESI): *m*/*z* calcd for C_11_H_17_FN_3_O^+^: 226.1344, found, 226.1346 [M + H^+^].

#### N-*tert*-Butyl-2-(ethylamino)-6-fluoronicotinamide (**5j**)

**5j** (82% yield, colorless solid) was prepared from **3f** and *N*-*tert*-butylamine according to the same procedure as described for **5e**. ^1^H-NMR [400 MHz, (CD_3_)_2_SO]: δ 8.56 (d, *J* = 5.6 Hz, 1H), 8.08 (t, *J* = 8.3 Hz, 1H), 7.70 (s, 1H), 6.16 (dd, *J* = 8.2, 2.9 Hz, 1H), 3.38–3.17 (m, 6H), 1.36 (s, 9H), 1.14 (t, *J* = 7.2 Hz, 3H). ^13^C-NMR (101 MHz, (CD_3_)_2_SO) δ 166.2 (d, *J* = 244.8 Hz), 162.6, 158.0 (d, *J* = 18.8 Hz), 143.0 (d, *J* = 9.8 Hz), 108.5 (d, *J* = 4.2 Hz), 93.1 (d, *J* = 38.1 Hz), 51.5, 35.6, 29.0, 15.1. ^19^F-NMR [376 MHz, (CD_3_)_2_SO]: δ -64.65. HRMS (ESI): *m*/*z* calcd for C_12_H_19_FN_3_O^+^: 240.15068, found, 240.15069 [M + H^+^].

#### [6-Fluoro-2-(methylamino)pyridin-3-yl](piperidin-1-yl)methanone (**5k**)

**5k** (92% yield, colorless solid) was prepared from **3e** and piperidine according to the same procedure as described for **5e**. ^1^H-NMR (400 MHz, CDCl_3_): δ 7.39 (t, *J* = 8.1 Hz, 1H), 6.24 (s, 1H, NH), 6.09 (dd, *J* = 8.0, 2.7 Hz, 1H), 3.56 (t, *J* = 5.5 Hz, 4H), 2.96 (d, *J* = 4.8 Hz, 3H), 1.74–1.58 (m, 7H). ^13^C-NMR (101 MHz, CDCl_3_): δ 168.7, 163.8 (d, *J* = 240.4 Hz), 157.9 (d, *J* = 18.2 Hz), 140.7 (d, *J* = 9.1 Hz), 109.5 (d, *J* = 5.1 Hz), 93.5 (d, *J* = 38.4 Hz), 29.7, 28.2, 26.2, 24.6. ^19^F-NMR (376 MHz, CDCl_3_): δ -65.86. HRMS (ESI): *m*/*z* calcd for C_12_H_17_FN_3_O^+^: 238.13502, found, 238.13506 [M + H^+^].

#### Di-*tert*-butyl [(*S*)-{1-(*tert*-butoxy)-6-[6-fluoro-2-(methylamino)nicotinamido]-1-oxohexan-2-yl}-carbamoyl]-(*S*)-glutamate (**5l**)

**5l** (47% yield, colorless solid) was prepared from **3e** and *t*BuO-Glu(O*t*Bu)-CO-Lys(H)-O*t*Bu^39^ according to the same procedure as described for **5a**. ^1^H-NMR (400 MHz, CDCl_3_) δ 8.56 (s, 1H), 7.97 (t, *J* = 8.0 Hz, 1H), 6.02 (dd, *J* = 8.2, 2.7 Hz, 1H), 5.59 (s, 1H), 5.32 (s, 1H), 4.30–4.16 (m, 2H), 3.42 (dd, *J* = 8.5, 4.1 Hz, 1H), 3.31 (d, *J* = 4.8 Hz, 1H), 2.97 (d, *J* = 4.7 Hz, 3H), 2.37–2.17 (m, 2H), 2.03 (s, 1H), 1.71 (m, 7H), 1.43 (2×s, 18H), 1.40 (s, 9H). ^13^C-NMR (101 MHz, CDCl_3_): δ 173.4, 173.5, 172.5, 172.5, 172.3, 168.1, 164.7 (d, *J* = 241.4 Hz), 159.2 (d, *J* = 19.2 Hz), 157.44, 141.2 (d, *J* = 10.1 Hz), 107.2 (d, *J* = 4.2 Hz), 93.3 (d, *J* = 38.4 Hz), 82.8, 81.9, 80.9, 76.8, 53.6, 53.3, 39.8, 32.9, 31.7, 28.7, 28.2, 28.1, 28.1, 28.0, 27.9, 23.4. ^19^F-NMR (376 MHz, CDCl_3_): δ -63.45. HRMS (ESI): *m*/*z* calcd for C_31_H_50_FN_5_O_8_Na^+^: 662.35356, found, 662.35404 [M + H^+^].

#### *tert-*Butyl *N*^2^-acetyl-*N*^6^-[6-fluoro-2-(methylamino)nicotinoyl]lysinate (**5m**)

**5m** (78% yield, colorless solid) was prepared from **3e** and Ac-Lys(H·HCl)-O*t*Bu according to the same procedure as described for **5a**. ^1^H-NMR (400 MHz, CDCl_3_) δ 8.52 (s, 1H), 7.86 (t, *J* = 8.1 Hz, 1H), 6.44 (s, 1H), 6.16 (d, *J* = 7.9 Hz, 1H), 6.03 (dd, *J* = 8.2, 2.9 Hz, 1H), 4.53 (td, *J* = 8.6, 4.4 Hz, 1H), 3.40 (dt, *J* = 6.7, 3.6 Hz, 2H), 3.00 (s, 3H), 2.01 (s, 3H), 1.76 (m, 2H), 1.71–1.60 (m, 2H), 1.48 (s, 9H), 1.45–1.38 (m, 2H). ^13^C-NMR (101 MHz, CDCl_3_) δ 171.79 (s), 170.2, 167.8, 164.6 (d, *J* = 240.9 Hz), 159.0 (d, *J* = 18.8 Hz), 140.3 (d, *J* = 10.1 Hz), 106.8 (d, *J* = 4.2 Hz), 93.3 (d, *J* = 38.7 Hz), 82.5, 52.0, 39.4, 32.9, 28.1, 27.8, 22.4. ^19^F-NMR (376 MHz, CDCl_3_) δ -63.00. ESI-MS: *m*/*z* 397.53 [M + H^+^], 419.12 [M + Na^+^]. HRMS (ESI): *m*/*z* calcd for C_19_H_30_FN_4_O_4_^+^: 397.22456, found, 397.22494 [M + H^+^].

#### Methyl [6-fluoro-2-(methylamino)nicotinoyl]glycinate (**5n**)

**5n** (94% yield, colorless solid) was prepared from **2e** and HCl·H-Gly-OMe according to the same procedure as described for **5c**. ^1^H-NMR (400 MHz, CDCl_3_) δ 8.37 (s, 1H), 7.75 (t, *J* = 8.1 Hz, 1H), 6.51 (s, 1H), 6.06 (dd, *J* = 8.2, 2.9 Hz, 1H), 4.18 (d, *J* = 5.1 Hz, 2H), 3.83 (s, 3H), 3.00 (s, 3H). ^13^C-NMR (101 MHz, CDCl_3_) δ 170.6, 167.5, 164.9 (d, *J* = 242.3 Hz), 159.0 (d, *J* = 19.0 Hz), 140.4 (d, *J* = 10.4 Hz), 105.8 (d, *J* = 4.2 Hz), 93.8 (d, *J* = 39.0 Hz), 52.6, 41.5, 27.9. ^19^F-NMR (376 MHz, CDCl_3_) δ -61.51. ESI-MS: *m*/*z* 242.23 [M + H^+^], 264.21 [M + Na^+^]. HRMS (ESI): *m*/*z* calcd for C_12_H_25_FN_2_O_3_^+^: 242.09354, found,242.09369 [M + H^+^].

#### *tert-*Butyl *N*^6^-[(benzyloxy)carbonyl]-*N*^2^-[6-fluoro-2-(methylamino)nicotinoyl]lysinate (**5o**)

**5o** (91% yield, colorless solid) was prepared from **2e** and HCl·H-Lys(Z)-O*t*Bu according to the same procedure as described for **5c**. ^1^H-NMR (400 MHz, CDCl_3_): δ 8.39 (s, 1H), 7.82 (t, *J* = 8.0 Hz, 1H), 7.46–7.31 (m, 5H), 6.67 (d, *J* = 7.0 Hz, 1H), 6.04 (dd, *J* = 8.2, 2.8 Hz, 1H), 5.17–4.94 (m, 2H), 4.83 (s, 1H), 4.56 (dd, *J* = 12.0, 7.2 Hz, 1H), 3.29–3.14 (m, 2H), 2.99 (s, 3H), 1.99–1.72 (m, 2H), 1.68–1.33 (m, 4H), 1.51 (s, 9H). ^13^C-NMR (101 MHz, CDCl_3_): δ 171.8, 167.3, 166.6 (d, *J* = 122.6 Hz), 159.0 (d, *J* = 19.0 Hz), 156.7, 140.5 (d, *J* = 10.2 Hz), 136.5, 128.6, 128.2, 128.0, 106.3 (d, *J* = 3.9 Hz), 93.6 (d, *J* = 38.8 Hz), 82.50, 66.69, 52.77, 40.37, 32.01, 29.65, 28.04, 27.85, 22.26. ^19^F-NMR (376 MHz, CDCl_3_): δ -62.14. ESI-MS: *m*/*z* 489.31 [M + H^+^], 511.07 [M + Na^+^]. HRMS (ESI): *m*/*z* calcd for C_19_H_30_FN_4_O_4_^+^: 489.25132, found, 489.25106, [M + H^+^].

#### ((*S*)-{1-Carboxy-5-[6-fluoro-2-(methylamino)nicotinamido]pentyl}carbamoyl)-(*S*)-glutamic Acid (**5p**, JK-PSMA-15)

**5l** (65 mg, 0.10 mmol) was treated with 95% TFA (1 mL) for 3 h at ambient temperature. TFA was removed *in vacuo* and another portion of TFA (1 mL) was added. This was concentrated slowly over the course of 1 h on the rotary evaporator. The residue was dissolved in a small amount of MeOH and precipitated with Et_2_O. The mixture was sonicated, and the precipitate was isolated by filtration to afford JK-PSMA-15 (30 mg, 63% yield) as a colorless solid. ^1^H-NMR (400 MHz, CD_3_OD) δ7.77 (td, *J* = 8.1, 2.9 Hz, 1H), 5.92 (dd, *J* = 8.2, 2.2 Hz, 1H), 4.16–4.01 (m, 1H), 3.27–3.00 (m, 1H), 2.74 (s, 3H), 2.54–2.35 (m, 1H), 2.30–2.16 (m, 2H), 2.00–1.88 (m, 1H), 1.83 (d, *J* = 7.1 Hz, 1H), 1.82–1.55 (m, 2H), 1.56–1.36 (m, 3H), 1.36–1.18 (m, 3H). ^19^F-NMR (376 MHz, CD_3_OD) δ -62.93, -77.06. ^13^C-NMR (101 MHz, CD_3_OD) δ 174.7, 174.5, 174.2, 168.4, 165.3 (d, *J* = 237.7 Hz), 159.3 (s, *J* = 2.2 Hz), 142.1 (d, *J* = 10.2 Hz), 108.4 (d, *J* = 4.4 Hz), 93.9 (d, *J* = 41.6 Hz), 39.8, 32.1, 30.5, 29.5, 27.9, 23.5. HRMS (ESI): *m*/*z* calcd for C_16_H_14_FN_5_O_2_Na^+^: 350.10237, found, 350.10228 [M + H^+^].

#### 2-{[(1-Benzyl-1*H*-1,2,3-triazol-4-yl)methyl]amino}-6-fluoronicotinic Acid (**6a**)

Sodium ascorbate (0.7 equiv) was added to a 1.8 M solution of **2g** (423 mg, 2.18 mmol, 1 equiv) and benzyl azide (290 mg, 2.18 mmol, 1 equiv) in 75% MeOH, and the mixture was briefly sonicated. 1 M CuSO_4_·5H_2_O (0.2 equiv) was added, which induced a color change to yellow-green. The reaction mixture was briefly sonicated again, kept in a water bath at ambient temperature for 2 h, and then concentrated under reduced pressure. The remaining oil was taken up in CH_2_Cl_2_ or EtOAc and washed with a saturated aqueous solution of EDTA until the aqueous phase was colorless. The organic fraction was dried over Na_2_SO_4_, and the solvent was removed under reduced pressure to afford **6a** (524 mg, 74% yield) as a colorless solid. ^1^H-NMR [400 MHz, (CD_3_)_2_SO]: δ 8.6 (t, *J* = 5.9 Hz, 1H), 8.1 (td, *J* = 8.3, 1.9 Hz, 1H), 7.7–7.5 (m, 1H), 7.4–7.1 (m, 5H), 6.2–5.9 (m, 1H), 5.4 (s, 2H), 4.6 (d, *J* = 5.6 Hz, 2H). ^13^C-NMR [101 MHz, (CD_3_)_2_SO]: δ 167.7, 164.2 (d, *J* = 242.7 Hz), 157.5 (d, *J* = 19.6 Hz), 145.0 (d, *J* = 10.5 Hz), 144.6, 134.3, 128.1, 127.6, 127.1, 121.6, 111.4, 103.4 (d, *J* = 4.4 Hz), 94.3 (d, *J* = 38.1 Hz), 52.9, 35.6. ^19^F-NMR [376 MHz, (CD_3_)_2_SO]: δ -60.8. HRMS (ESI): *m*/*z* calcd for C_16_H_14_FN_5_O_2_Na^+^: 350.10237, found, 350.10228 [M + H^+^].

#### 2-({[(*S,S*)-1-{6-(*tert*-Butoxy)-5-[3-(1,5-di-*tert*-butoxy-1,5-dioxopentan-2-yl)ureido]-6-oxohexyl}-1*H*-1,2,3-triazol-4-yl]methyl}amino)-6-fluoronicotinic Acid (**10**)

**10** (87% yield, colorless solid) was prepared from **2g** and 1,5-di-*tert*-butyl (*S*)-2-({[(*S*)-6-azido-1-(*tert*-butoxy)-1-oxohexan-2-yl]carbamoyl}amino)pentanedioate (**9**; for preparation, see Scheme S1 in the Supporting Information) according to the same procedure as described for **6a**. ^1^H-NMR (400 MHz, CDCl_3_): δ 9.04 (s, 1H), 8.27 (t, *J* = 8.2 Hz, 1H), 7.71 (s, 1H), 6.12 (dd, *J* = 8.3, 2.5 Hz, 1H), 5.46 (d, *J* = 8.1 Hz, 1H), 5.39 (d, *J* = 7.9 Hz, 1H), 4.82 (t, *J* = 4.6 Hz, 2H), 4.33 (d, *J* = 6.1 Hz, 4H), 2.32 (dq, *J* = 16.4, 9.8 Hz, 2H), 2.13–2.00 (m, 1H), 1.99–1.70 (m, 4H), 1.69–1.56 (m, 1H), 1.45 (s, 9H). 1.44 (s, 9H), 1.43 (s, 9H). ^13^C-NMR (101 MHz, CDCl_3_): δ 172.67, 172.20, 166.57, 164.15, 158.48, 158.29, 157.13, 146.00, 145.19, 123.16, 104.17, 95.30, 94.92, 82.20, 80.75, 53.20, 53.16, 53.16, 50.20, 35.58, 32.30, 31.65, 29.60, 28.19, 28.06, 27.99, 27.96, 21.92. ^19^F-NMR (376 MHz, CDCl_3_): δ -60.40. HRMS (ESI): *m*/*z* calcd for C_313_H_51_FN_7_O_9_Na^+^: 708.37268, found, 708.37272 [M + Na^+^].

#### ({1-Carboxy-5-[(*S*)-4-{[(3-carboxy-6-fluoropyridin-2-yl)amino]methyl}-1*H*-1,2,3-triazol-1-yl]pentyl}carbamoyl)-(*S*)-glutamic Acid (**6b**, JK-PSMA-16)

**6b** (20% yield, colorless solid) was prepared from **10** according to the same procedure as described for JK-PSMA-15. ^1^H-NMR (400 MHz, CD_3_OD): δ 8.29 (t, *J* = 8.3 Hz, 1H), 7.95 (s, 1H), 6.20 (dd, *J* = 8.3, 2.6 Hz, 1H), 4.74 (s, 2H), 4.40 (t, *J* = 7.1 Hz, 2H), 4.31 (ddd, *J* = 15.4, 8.4, 5.0 Hz, 2H), 3.33 (dt, *J* = 3.2, 1.6 Hz, 1H), 2.54–2.34 (m, 2H), 2.26–2.10 (m, 1H), 2.06–1.79 (m, 4H), 1.70 (dq, *J* = 14.8, 8.1 Hz, 1H), 1.44 (dd, *J* = 14.6, 6.9 Hz, 2H). ^13^C-NMR (101 MHz, CD_3_OD): δ 177.4, 175.2, 174.9, 173.6, 168.5, 166.3 (d, *J* = 243.4 Hz), 158.5 (d, *J* = 41.4 Hz), 152.5, 145.8 (d, *J* = 10.1 Hz), 104.1 (d, *J* = 5.1 Hz), 94.8 (d, *J* = 38.4 Hz), 52.56, 49.68, 35.64, 31.43, 31.21, 29.66, 29.37, 22.10. ^19^F-NMR (376 MHz, CD_3_OD): δ -60.88. MS (ESI): *m*/*z* calcd for C_21_H_25_FN_7_O_9_^–^: 538.17, found, 538.26 [M – H^+^]. HRMS (ESI): *m*/*z* calcd for C_21_H_27_FN_7_O_9_^+^: 540.18488, found, 540.18491 [M + H^+^].

#### 6-Fluoro-2-{[3-(4-phenyl-1*H*-1,2,3-triazol-1-yl)propyl]amino}nicotinic Acid (**6c**)

**6c** (85% yield, colorless solid) was prepared from **2h** and phenylacetylene according to the same procedure as described for **6a**. ^1^H-NMR (400 MHz, CDCl_3_) δ 8.30 (td, *J* = 8.4, 3.3 Hz, 2H), 8.02 (s, 1H), 7.87 (d, *J* = 7.2 Hz, 2H), 7.45 (t, *J* = 7.2 Hz, 2H), 7.36 (t, *J* = 7.3 Hz, 1H), 6.17 (td, *J* = 8.4, 2.8 Hz, 1H), 4.56 (t, *J* = 6.6 Hz, 2H), 3.63 (q, *J* = 5.9 Hz, 2H), 2.44–2.26 (m, 2H). ^13^C-NMR (101 MHz, CD_3_OD): δ 168.6, 165.4 (d, *J* = 242.0 Hz), 158.9 (d, *J* = 19.1 Hz), 147.4, 145.8 (d, *J* = 10.6 Hz), 130.4, 128.5, 127.9, 125.3, 121.0, 104.2 (d, *J* = 6.2 Hz), 94.2 (d, *J* = 38.4 Hz), 47.9, 37.4, 29.6. ^19^F-NMR (376 MHz, CD_3_OD): δ -63.23. HRMS (ESI): *m*/*z* calcd for C_17_H_15_FN_5_O_2_^–^: 340.12153, found, 340.12171 [M – H^+^].

#### Di-*tert*-butyl {[(*S*)-1-(*tert*-butoxy)-1-oxo-6-propiolamidohexan-2-yl]carbamoyl}-(*S*)-glutamate (**11**)

A solution of pentafluorophenyl propiolate^40^ (1.28 g, 5.50 mmol) in DMF (1.5 mL) was rapidly added to a stirred solution of *t*BuO-Glu(O*t*Bu)-CO-Lys(H)-O*t*Bu (2.44 g, 5.00 mmol) and DIEA (1.15 mL, 0.85 g, 6.57 mmol) in DMF (10 mL) at −10 °C (see Scheme S2 in the Supporting Information). Stirring was continued at −10 °C for 30 min, after which the reaction mixture was allowed to reach ambient temperature and stirring was continued for another 2 h. The solution was then concentrated under reduced pressure, taken up in 0.25 M HCl (20 mL), and extracted three times with ether. The combined organic fractions were washed with water and brine, dried over sodium sulfate, and purified by flash chromatography (EtOAc/petrol ether 1:1). After lyophilization from MeCN, **11** (2.35 g, 87% yield) was obtained as a white amorphous powder. R_f_: 0.29 (EtOAc/petrol ether 1:1) ^1^H-NMR (200 MHz, CD_3_OD): δ 6.41 (1H), 6.37 (1H), 4.22 (m, 2H) 3.58 (s, 1H), 3.25 (t, *J* = 6.7 Hz, 2H), 2.35 (m, 2H), 2.18-1.52 (m, 8H), 1.51 (s, 9H). 1.50 (s, 9H), 1.48 (s, 9H). ^13^C-NMR (50 MHz, CD_3_OD): δ 173.83, 173.71, 173.43, 159.89, 154.64, 82.77, 82.58, 81.70, 78.30, 75.64, 54.74, 54.14, 40.36, 33.16, 32.46, 29.60, 28.96, 28.36, 28.32, 28.29, 23.85. HRMS (ESI) calcd for C_27_H_46_N_3_O_8_: 540.32794, found, 540.32819 [M + H^+^].

#### {[(*S*)-1-Carboxy-5-propiolamidopentyl]carbamoyl}-(*S*)-glutamic Acid (**8**)

**11** (1.00 g, 1.85 mmol) was dissolved in TFA/water/TIPS (20 mL; 95:2.5:2.5) and stirred at ambient temperature for 1 h (see Scheme S2 in the Supporting Information). The solution was concentrated under reduced pressure, and the resulting oil was dissolved in MeCN (20 mL) and concentrated again under reduced pressure three times. Lyophilization from water gave **8** (0.68 g, 99% yield) as a hygroscopic white powder. ^1^H-NMR (200 MHz, D_2_O): δ 4.15 (m, 2H), 3.36 (s, 1H), 3.15 (t, *J* = 6.5 Hz, 2H), 2.42 (t, *J* = 7.2 Hz, 2H), 2.18–1.15 (m, 8H). ^13^C-NMR (50 MHz, D_2_O): δ 177.19, 177.00, 176.17, 159.27, 154.32, 76.37, 75.93, 53.13, 52.52, 39.43, 30.55, 30.03, 27.44, 26.26, 22.28. HRMS (ESI) calcd for C_15_H_22_N_3_O_8_: 372.14014, found, 372.14031 [M + H^+^].

#### {[1-Carboxy-5-(1-{3-[(*S*)-(3-carboxy-6-fluoropyridin-2-yl)amino]propyl}-1*H*-1,2,3-triazole-4-carboxamido)pentyl]carbamoyl}-(*S*)-glutamic Acid (**6d**, JK-PSMA-18)

JK-PSMA-18 (44% yield, colorless solid) was prepared from **2h** and **8** according to the same procedure as described for **6a**. ^1^H-NMR (400 MHz, CD_3_CN): δ 8.58–8.39 (m, 1H), 8.31 (t, *J* = 8.4 Hz, 1H), 8.06–7.83 (m, 1H), 6.29 (t, *J* = 11.7 Hz, 1H), 6.26–6.16 (m, 1H), 4.68 (dt, *J* = 55.4, 6.9 Hz, 1H), 4.50–4.39 (m, 1H), 4.36 (td, *J* = 8.0, 4.8 Hz, 1H), 3.66–3.53 (m, 1H), 3.45 (ddq, *J* = 20.3, 13.3, 6.9 Hz, 1H), 2.56–2.39 (m, 1H), 2.33 (p, *J* = 6.8 Hz, 1H), 2.27–2.12 (m, 1H), 2.02–1.85 (m, 1H), 1.85–1.58 (m, 2H), 1.58–1.44 (m, 1H). ^13^C-NMR (101 MHz, CD_3_CN): δ 173.7, 173.5, 173.4, 167.8, 165.4 (d, *J* = 242.4 Hz), 160.27, 159.1 (d, *J* = 30.3 Hz), 158.2, 146.0 (d, *J* = 11.1 Hz), 143.2, 126.0, 103.3 (d, *J* = 4.9 Hz), 94.7 (d, *J* = 39.4 Hz), 52.9, 52.3, 48.0, 38.3, 37.8, 31.6, 29.7, 29.2, 27.8, 22.5. ^19^F-NMR (376 MHz, CD_3_CN): δ -61.48. HRMS (ESI): *m*/*z* calcd for C_24_H_28_FN_8_O_10_^3–^: 202.39726, found, 202.39763 [M – 3H^+^].

### Radiochemistry

All radiosyntheses were carried out using anhydrous solvents (Aldrich). Anion exchange resins (PS-HCO_3_^–^, 45 mg sorbent) were obtained from Synthra GmbH (Hamburg, Germany) and preconditioned with 1 mL of H_2_O directly before use. Solid-phase extraction (SPE) cartridges (Oasis HLB Plus Short and HLB PriME Light) were obtained from Waters GmbH (Eschborn, Germany) and used without preconditioning. [^18^F]Fluoride ([^18^F]F^–^) was produced via the ^18^O(p,n)^18^F reaction by bombardment of enriched [^18^O]water with 16.5 MeV protons using a BC1710 cyclotron (The Japan Steel Works Ltd., Shinagawa, Japan) at the INM-5 (Forschungszentrum Jülich, Germany). All radiolabeling experiments were carried out under ambient atmosphere. Before radiosynthesis, [^18^F]F^–^ was processed as follows. Aqueous [^18^F]F^–^ was loaded onto the anion exchange resin from the male side, whereas flushing, washing, and [^18^F]F^–^-elution were carried out from the female side. To determine radiochemical conversions (RCCs) as a measure of the efficiency of a specific labeling reaction,^44^ reaction mixtures were diluted with an equal volume of H_2_O (to dissolve any [^18^F]F^–^ adsorbed onto the reaction vessel walls) and analyzed by radio-HPLC. The RCCs were then calculated by dividing the integrated peak area of the radiolabeled product by the integrated area of a post-column injection peak.^28^ Isolated yields of ^18^F-labeled compounds were determined for the radiochemically and chemically pure products and are reported in terms of decay-corrected radiochemical yields (RCYs) and/or non-decay-corrected activity yields (AYs), respectively, as recommended in the consensus nomenclature rules for radiopharmaceutical chemistry.^45^ HPLC analyses were carried out on a Dionex Ultimate 3000 HPLC system and a DAD UV-detector coupled in series with a Berthold NaI detector. A Multokrom C18 AQ 100-5, 250 mm × 4.6 mm column (CS-Chromatographie Service GmbH, Langerwehe, Germany) was used. UV and radioactivity detectors were connected in series, giving a time delay of 0.1–0.6 min depending on the flow rate, exact peak shapes, and the length of the capillary between both detectors. ^18^F-Labeled compounds were identified by co-injection of the nonradioactive reference compounds using HPLC. General methods for HPLC can be found in the Supporting information. The HPLC system used for the purification of crude products consisted of a Merck Hitachi L-6000 pump, a Knauer K-2500 detector, a Rheodyne 6-way valve, a Geiger-Müller counter, and a Hydro-RP, 250 mm × 10 mm, 80 Å, 10 μm column (Synergi; Phenomenex LTD, Aschaffenburg, Germany).

#### Production of [^18^F]AFAs ([^18^F]**3e-h**)—General Procedure 1 (GP 1)

[^18^F]F^–^ was loaded onto an anion exchange-cartridge (PS-HCO_3_^–^, 45 mg sorbent). The cartridge was washed with anhydrous MeCN (4 mL), and [^18^F]F^–^ was eluted dropwise with a solution of the respective *N,N,N*-trimethylammonium triflate precursor (12 mg of **4a**/**4b** or 15 mg of **4c**/**4d**) in 1 mL of MeCN/*t*BuOH (1:4) into a vial containing H_2_O (35 mL). The cartridge was additionally flushed with MeCN (1 mL), which was collected into the same vial. The resulting solution was passed through an SPE cartridge (Oasis HLB Plus Short), the cartridge was washed with H_2_O (5 mL), briefly dried in a steam of argon (1 min), and the labeled product was eluted with MeCN (1 mL).

#### Production of [^18^F]**5a-m**—General Procedure 2 (GP 2)

To [^18^F]**3e-f** in MeCN (50 μL) was added the respective amine (10 μmol) in MeCN, DMF, or 20% MeCN in 0.2 M borate buffer at pH 8.7 (450 μL). The mixture was stirred at 40–110 °C for 10–20 min as indicated (see Scheme 4), and RCCs were determined by HPLC as described in the general radiochemical section.

#### Preparation of [^18^F]**5p** ([^18^F]JK-PSMA-15)

[^18^F]**3e** was synthesized from [^18^F]F^–^ (6.9 GBq) according to GP1 and loaded onto an Oasis HLB Plus Short cartridge. The cartridge was washed with 5% acetone (6 mL) and briefly dried in a stream of argon. [^18^F]**3e** was eluted from the cartridge with MeCN (1.5 mL). The solvent was removed under reduced pressure at 100 °C before a solution of *t*BuO-Glu(O*t*Bu)-CO-Lys(H)-O*t*Bu (10 μmol) in MeCN (500 μL) was added. The vial was equipped with a septum cap and heated under stirring at 60 °C for 15 min. Thereafter, 38% HCl (500 μL) was added and the mixture was stirred for another 15 min at 60 °C. The resulting crude product was purified by semipreparative HPLC [eluent: 20% MeCN (0.1% TFA); flow rate: 8 mL/min; t_R_ = 8–9 min]. The product fraction was diluted with H_2_O to 35 mL and loaded onto an SPE cartridge (Oasis HLB Plus Short). The cartridge was washed with H_2_O (10 mL) and [^18^F]JK-PSMA-15 was eluted with MeOH (1.5 mL). MeOH was removed under reduced pressure in a stream of argon at 60 °C and [^18^F]JK-PSMA-15 was dissolved in 0.9% NaCl (450 μL) containing sodium ascorbate (4.5 mg) to afford the desired tracer as a solution ready for injection. The average isolated AYs amounted to 16 ± 3% (*n* = 5), within a total synthesis time of 90 min. The radiochemical purity amounted to >97% and the molar activity to 99 GBq/μmol (for 980 MBq [^18^F]**5p**).

#### Radiosynthesis of [^18^F]**6a-d** via the Azide Alkyne “Click” Cycloaddition—General Procedure 3 (GP 3)

A solution of [^18^F]**3g** or [^18^F]**3h** in MeCN prepared according to GP1 was concentrated under reduced pressure in a stream of argon for 5 min at 80 °C. 10 mM NaOH (0.5 mL) was added to the residue, and the reaction mixture was stirred at 60 °C for 5 min. Then, 100 μL stock solutions of the following reagents were added (in the indicated order). For [^18^F**]3g**: 0.2 M CuSO_4_, 0.5 M l-histidine, 1 M sodium ascorbate, and a 0.2 M solution of the respective azide in MeCN or MeOH. For [^18^F]**3h**: 0.2 M CuSO_4_, 0.5 M l-histidine, and 1 M sodium ascorbate were added to a 0.2 M solution of the respective alkyne in MeCN or MeOH, and the resulting mixture was added to the solution of the radiolabeled building block. The reaction mixture was stirred at 60 °C for 15 min, cooled to ambient temperature, diluted with H_2_O, and analyzed by HPLC as described above.

#### Preparation of [^18^F]**6a**

[^18^F]**6a** was synthesized in RCCs of 77 ± 2% (*n* = 3) according to GP3 by the reaction of [^18^F]**2g** with benzyl azide (20 μmol in MeCN).

#### Preparation of [^18^F]**6b** ([^18^F]JK-PSMA-16)

[^18^F]**3g** was synthesized from [^18^F]F^–^ (4.5 GBq) and **4c** (15 mg, 33 μmol) in 1 mL of MeCN/*t*BuOH (1:4) according to GP1. A solution of the crude prosthetic group was diluted with H_2_O (35 mL) and loaded onto an SPE cartridge (Oasis HLB Plus Short). The cartridge was washed with 5% acetone (6 mL) and briefly dried in a steam of argon. [^18^F]**3g** was eluted from the cartridge with MeCN (1.5 mL). The solvent was removed under reduced pressure at 100 °C, and the title radiolabeled compound was prepared according to GP 3 using azide **7** (1.9 mg; 0.2 M in MeOH; for preparation, see Scheme S1 in the Supporting Information). The crude product was purified by semipreparative HPLC [column: Hydro-RP, 250 mm × 10 mm; eluent: 30% MeCN (0.1% TFA); flow rate: 4.7 mL/min; t_R_ = 9 min]. The product fraction was diluted with H_2_O to 35 mL and loaded onto an SPE cartridge (Oasis HLB Plus Short), which was washed with H_2_O (10 mL). [^18^F]JK-PSMA-16 was eluted into a vial with MeOH (1.5 mL), the solvent was removed under reduced pressure in a stream of argon at 60 °C, and the tracer was formulated as described for [^18^F]JK-PSMA-15. [^18^F]JK-PSMA-16 was obtained in AYs of 21 ± 3% (*n* = 3) within a total synthesis time of 90 min. Radiochemical purity was >99%.

#### Preparation of [^18^F]**6c**

[^18^F]**6c** was synthesized in RCCs of 88 ± 6% (*n* = 3) according to GP3 by the reaction of [^18^F]**2h** with phenylacetylene (1.0 mg, 10 μmol as a 0.2 M solution in MeCN).

#### Preparation of [^18^F]**6d** ([^18^F]JK-PSMA-18)

[^18^F]**3h** produced according to GP1 was hydrolyzed to [^18^F]**2h** according to GP3. The latter was conjugated with **8** (1.9 mg, 5 μmol; as a 0.2 M solution in MeOH). The reaction mixture was cooled to ambient temperature, TFA (10 μL) was added, and the radiolabeled product was isolated by semipreparative HPLC [eluent: 30% MeCN (0.1% TFA); flow rate: 4.7 mL/min; *t*_R_ = 8.7 min]. The product fraction was diluted with H_2_O (35 mL), and the resulting solution was loaded onto an SPE cartridge (Oasis HLB Plus Short), which was washed with H_2_O (10 mL). The purified tracer was eluted with MeOH (1.5 mL), the solvent was removed under reduced pressure in a stream of argon at 60 °C and [^18^F]JK-PSMA-18 was formulated as described for [^18^F]JK-PSMA-15. [^18^F]JK-PSMA-18 was obtained in AYs of 28 ± 4% (*n* = 3) within a total synthesis time of 90 min. Radiochemical purity was >96%, and the molar activity was determined to be 75 GBq/μmol (for 89 MBq tracer).

### Determination of Carrier Content

The amount of nonradioactive carrier was calculated from the peak area in UV–HPLC chromatograms using a UV absorbance/concentration calibration curve. To this end, solutions of the radiolabeled products obtained after HPLC purification were allowed to stand at ambient temperature for at least 24 h, concentrated under reduced pressure, and the residues were redissolved in the appropriate HPLC eluents (200 μL). 100 μL of the resulting solution was injected into the HPLC system (20 μL loop, equals 10% of total carrier content). The peak area was determined, and the amount of carrier was calculated according to a calibration curve.

### Crystal Data for Compound **4c**

Formula: C_14_H_14_F_3_N_3_O_6_S; formula weight: 409.34 g/mol; crystal system: monoclinic; space group: *P*2_1_; unit cell parameters: *a* = 8.2196(2) Å, *b* = 13.1737(4) Å, *c* = 8.8585(2) Å, α = 90°, β = 115.1110(10)°, γ = 90°; temperature of data collection: 100(2) K; value of Z: 2; final values of R_1_, w*R*_2_ [I > 2σ(I)]: 0.0211, 0.0543; goodness-of-fit on F^2^: 1.063.

### Experimental Animals

Animal experiments were carried out in accordance with the EU directive 2010/63/EU and the German Animal Welfare Act (TierSchG, 2006) and were approved by the regional authorities (Ministry for Environment, Agriculture, Conservation and Consumer Protection of the State of North Rhine-Westphalia, license number 84-02.04.2015.A240). Nine healthy Long Evans rats (females; 237–370 g; three of them were measured twice) were used for this study. Rats were housed in groups of 2–3 in individually ventilated cages (NexGen Ecoflo, Allentown, Inc., Allentown, NJ) under controlled ambient conditions (22 ± 1 °C and 55 ± 5% relative humidity). Food and water were available *ad libitum*.

### *In Vivo* PET Experiments

Prior to PET measurements, animals were anesthetized with isoflurane in O_2_/air (3:7) [5% for induction, 1.5–2.5% for maintenance], and a catheter for tracer injection was inserted into the lateral tail vein. Rats were placed on an animal holder (Equipment Vétérinaire Minerve, Esternay, France) and fixed with a tooth bar in a respiratory mask. PET scans in list mode were performed using a Focus 220 micro PET scanner (CTI-Siemens, Germany) with a resolution at the center of field of view of 1.4 mm. Data acquisition started with the injection of the tracer (60.3 ± 5.6 MBq in 500 μL i.v.; for details, see Table S1 in the Supporting Information), continued for 120 min, and followed by a 10 min transmission scan using a ^57^Co point source. For each tracer (i.e., [^18^F]JK-PSMA-15, [^18^F]JK-PSMA-16, and [^18^F]JK-PSMA-18), three rats were measured. For PSMA blocking, 2-(phosphonomethyl)pentanedioic acid (2-PMPA; 23 mg/kg; *n* = 1 per tracer) was added directly to the radiotracer solution. The breathing rate was monitored and maintained at around 60/min by adjusting the isoflurane concentration (1.5–2.5%). Body temperature was maintained at 37 °C by a feedback-controlled system. After the scan, the rat was returned to its home cage.

The emission scans were histogrammed into time frames (2 × 1 min, 2 × 2 min, 6 × 4 min, 18 × 5 min for time-activity curves, 4 × 30 min for signal-to-background ratio, and 2 min × 60 min for display) and fully 3D rebinned (span 3, ring difference 47), followed by OSEM3D/MAP reconstruction with attenuation and decay correction.^46^ The resulting voxel sizes were 0.47 × 0.47 × 0.80 mm^3^. Postprocessing and image analysis was performed with VINCI 5.21 (Max-Planck-Institute for Metabolism Research, Cologne, Germany). Images were intensity-normalized to injected dose and corrected for body weight (SUV_bw_). To this end, every frame was divided by injected dose and multiplied by body weight times 100. Time-activity curves were determined for volumes of interest (VOIs) placed in the superior cervical ganglion (SCG), blood (lumen of the left ventricle), liver, and bone (sternum). Tracer accumulation in the same VOIs and background (neck muscles) was also measured for the 60–120 min frame and compared between tracers using one-way ANOVA followed by Dunnett’s multiple comparisons test. For SCGs, the signal-to-background ratio and edge contrast were determined, while the resolution was calculated for the first pair of DRGs (described in detail by Zlatopolskiy et al.^10^ and in the Supporting information). Data of [^18^F]JK-PSMA-7 from a previous publication^10^ were included for comparison.

## Supporting Information Available

Synthesis schemes for compounds **7–9** and **11**, NMR spectra and HPLC traces of synthesized compounds, calibration curves for determination of carrier content, supplementary methods, and additional results of the *in vivo* experiments (PDF)
Molecular formula strings (CSV)

## Accession Codes

The single-crystal structure information of compound **4c** is deposited at the Cambridge Crystallographic Data Centre (CCDC, deposition number: 2205488). The authors will release the atomic coordinates and experimental data upon article publication.

## Abbreviations Used

[^18^F]AFA: 1-alkylamino-7-[^18^F]fluoro-8-azaisatoic anhydride
AY: activity yield
[^18^F]F^–^: [^18^F]fluoride
[^18^F]SFB: *N*-succinimidyl 4-[^18^F]fluorobenzoate
[^18^F]FPy-TFP: 2,3,5,6-tetrafluorophenyl 6-[^18^F]fluoronicotinate
2-PMPA: 2-(phosphonomethyl)pentanedioic acid
CuAAC: copper-catalyzed alkyne-azide cycloaddition
DRG: cervical dorsal root ganglia
*E*_max_: maximum positron energy
EOS: end of synthesis
H: heart
L: liver
PCa: prostate cancer
PET: positron emission tomography
PG: prosthetic group
PSMA: prostate-specific membrane antigen
RCC: radiochemical conversion
RCY: radiochemical yield
SCG: superior cervical ganglion
SG: salivary gland
SJ: shoulder joint
SPE: solid-phase extraction
SUV_bw_: standardized uptake value (normalized to body weight)
*t*BuO-Glu(O*t*Bu)-CO-Lys(H)-O*t*Bu: di-*tert*-butyl {[(*S*)-6-amino-1-(*tert*-butoxy)-1-oxohexan-2-yl]carbamoyl}-(*S*)-glutamate
VOI: volume of interest
