# Supplementary material for: 7-[18F]Fluoro-8-azaisatoic Anhydrides: Versatile Prosthetic Groups for the Preparation of PET Tracers
Source: J Med Chem. 2023 Aug 25;66(17):12629–44. doi: 10.1021/acs.jmedchem.3c01310 (PMC10510393; doi:10.1021/acs.jmedchem.3c01310)
Supplement: Supplementary file 1 — jm3c01310_si_001.pdf [file jm3c01310_si_001.pdf]

Supporting Information

7-[^{18}F]Fluoro-8-azaisatoic Anhydrides: Versatile Prosthetic Groups for the Preparation of PET-Tracers

Benedikt Gröner[a,b#], Michael Willmann[a#], Lisa Donnerstag[a,b], Elizaveta A. Urusova[a,b], Felix Neumaier[a,b], Swen Humpert[a], Heike Endepols[a,b,c], Bernd Neumaier[a,b,d*], and Boris D. Zlatopolskiy[a,b,d]

[a] Forschungszentrum Jülich GmbH, Institute of Neuroscience and Medicine, Nuclear Chemistry (INM-5), Wilhelm-Johnen-Straße, 52428 Jülich, Germany.

[b] University of Cologne, Faculty of Medicine and University Hospital Cologne, Institute of Radiochemistry and Experimental Molecular Imaging, Kerpener Straße 62, 50937 Cologne, Germany.

[c] University of Cologne, Faculty of Medicine and University Hospital Cologne, Department of Nuclear Medicine, Kerpener Straße 62, 50937 Cologne, Germany.

[d] Max Planck Institute for Metabolism Research, Gleueler Straße 50, 50931, Cologne, Germany.

* Corresponding author: b.neumaier@fz-juelich.de;

# B.G. and M.W. contributed equally

Table of contents

1 Synthesis schemes 3
1.1 Compounds **7** and **9**[1] (**Scheme S1**) 3
1.2 Compounds **8** and **11** (**Scheme S2**) 3
2 NMR-spectra 4
2.1 Compound **2a** 4
2.2 Compound **2b** 5
2.3 Compound **2c** 6
2.4 Compound **2d** 7
2.5 Compound **2e** 8
2.6 Compound **2f** 10
2.7 Compound **2g** 12
2.8 Compound **2h** 14
2.9 Compound **3a** 16
2.10 Compound **3b** 17
2.11 Compound **3c** 18
2.12 Compound **3d** 19
2.13 Compound **3e** 20
2.14 Compound **3f** 22
2.15 Compound **3g** 24
2.16 Compound **3h** 26
2.17 Compound **4a** 28
2.18 Compound **4b** 30
2.19 Compound **4c** 32
2.20 Compound **4d** 34
2.21 Compound **5a** 36
2.22 Compound **5b** 38
2.23 Compound **5c** 40
2.24 Compound **5d** 42
2.25 Compound **5e** 44

2.26 Compound **5f** ... 46
2.27 Compound **5g** ... 48
2.28 Compound **5h** ... 50
2.29 Compound **5i** ... 52
2.30 Compound **5j** ... 54
2.31 Compound **5k** ... 56
2.32 Compound **5l** ... 58
2.33 Compound **5m** ... 60
2.34 Compound **5n** ... 62
2.35 Compound **5o** ... 64
2.36 Compound **5p** (JK-PSMA-15) ... 66
2.37 Compound **6a** ... 67
2.38 Compound **10** ... 69
2.39 Compound **6b** (JK-PSMA-16) ... 71
2.40 Compound **6c** ... 73
2.41 Compound **11** ... 75
2.42 Compound **8** ... 77
2.43 Compound **6d** (JK-PSMA-18) ... 78
3 General Methods for Analytical HPLC: ... 80
4 HPLC chromatograms of ^{18}F-labeled AFAs: ... 81
4.1 HPLC traces of [^{18}F]**3e** (**Figs. S1** & **S2**) ... 81
4.2 HPLC traces of [^{18}F]**3f** (**Figs. S3** & **S4**) ... 82
4.3 HPLC traces of [^{18}F]**3g** (**Figs. S5** & **S6**) ... 83
4.4 HPLC traces of [^{18}F]**3h** (**Figs. S7** & **S8**) ... 84
5 HPLC chromatograms of radiolabeled model compounds und PET-tracers: ... 85
5.1 HPLC traces of [^{18}F]**5a** (**Figs. S9** & **S10**) ... 85
5.2 HPLC traces of [^{18}F]**5b** (**Figs. S11** & **S12**) ... 86
5.3 HPLC traces of [^{18}F]**5c** (**Figs. S13** & **S14**) ... 87
5.4 HPLC traces of [^{18}F]**5d** (**Figs. S15** & **S16**) ... 88
5.5 HPLC traces of [^{18}F]**5e** (**Figs. S17** & **S18**) ... 89
5.6 HPLC traces of [^{18}F]**5f** (**Figs. S19** & **S20**) ... 90
5.7 HPLC traces of [^{18}F]**5g** (**Figs. S21** & **S22**) ... 91
5.8 HPLC traces of [^{18}F]**5h** (**Figs. S23** & **S24**) ... 92
5.9 HPLC traces of [^{18}F]**5i** (**Figs. S25** & **S26**) ... 93
5.10 HPLC traces of [^{18}F]**5j** (**Figs. S27** & **S28**) ... 94
5.11 HPLC traces of [^{18}F]**5k** (**Figs. S29** & **S30**) ... 95
5.12 HPLC trace of [^{18}F]**5l** (**Fig. S31**) ... 96
5.13 HPLC trace of [^{18}F]**5m** (**Fig. S32**) ... 97
5.14 HPLC trace of [^{18}F]**5o** (**Fig. S33**) ... 98
5.15 HPLC trace for competition between Ac-Lys(H)-O*t*Bu and H-Lys(Z)-O*t*Bu (4:1) (**Fig. S34**) ... 99
5.16 HPLC trace for competition between *n*-butylamine and H-Gly-OMe (**Fig. S35**) ... 100
5.17 HPLC traces of [^{18}F]**5p** ([^{18}F]JK-PSMA-15) (**Figs. S36-S38**) ... 101
5.18 HPLC traces of [^{18}F]**6a** (**Figs. S39** & **S40**) ... 103
5.19 HPLC traces of [^{18}F]**6b** ([^{18}F]JK-PSMA-16) (**Figs. S41-S43**) ... 104
5.20 HPLC traces of [^{18}F]**6c** (**Figs. S44** & **S45**) ... 106
5.21 HPLC traces of [^{18}F]**6d** ([^{18}F]JK-PSMA-18) (**Figs. S46-S49**) ... 107
6 Calibration curves ... 109
6.1 Calibration curve for JK-PSMA-15 ... 109
6.2 Calibration curve for JK-PSMA-16 ... 109
6.3 Calibration curve for JK-PSMA-18 ... 110
7 In vivo experiments ... 111
7.1 Methods ... 111
7.1.1 Signal-to-background ratio (SCG/background) ... 111
7.1.2 Acutance ... 111
7.1.3 Resolution ... 111
7.2 Results (**Tab. S1-S5** & **Fig. S50**) ... 112
8 References ... 113

1 Synthesis schemes

1.1 Compounds **7** and **9**[1] (**Scheme S1**)

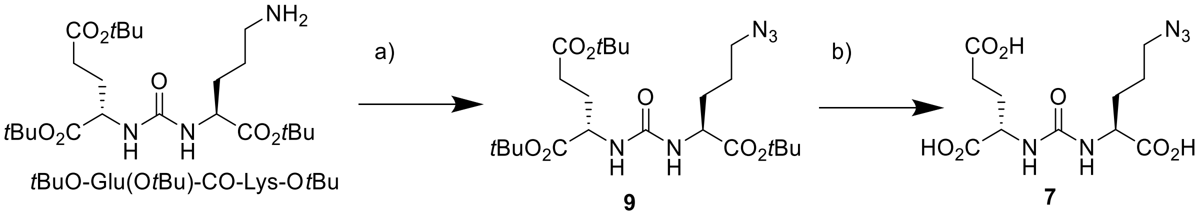

Scheme S1: Preparation of 1,5-di-*tert*-butyl (*S*)-2-({[(*S*)-6-azido-1-(*tert*-butoxy)-1-oxohexan-2-yl]carbamoyl}amino)pentanedioate (**9**) and (*S*)-2-({[(*S*)-5-azido-1-carboxypentyl]carbamoyl}amino)pentanedioic acid (**7**). Reaction conditions: a) K_2CO_3, $CuSO_4$, MeOH, imidazole-1-sulfonyl azide hydrochloride, r.t., 24 h, 64%; b) TFA, H_2O, TIPS (95:2.5:2.5), r.t., 2 h, 92%.

1.2 Compounds **8** and **11** (**Scheme S2**)

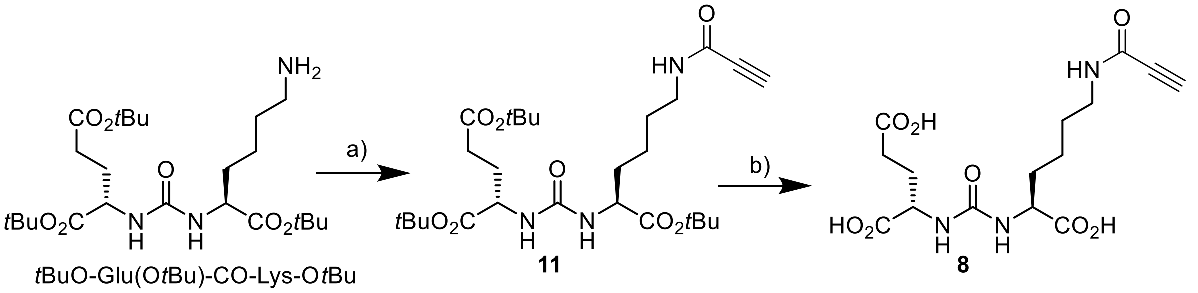

Scheme S2: Preparation of di-*tert*-butyl {[(*S*)-1-(*tert*-butoxy)-1-oxo-6-propiolamidohexan-2-yl]carbamoyl}-(*S*)-glutamate (**11**) and {[(*S*)-1-carboxy-5-propiolamidopentyl]carbamoyl}-(*S*)-glutamic acid (**8**). Reaction conditions: a) pentafluorophenyl propiolate, DIPEA, DMF, -10 °C to rt, 2 h, 87%; b) TFA, H_2O, TIPS (95:2.5:2.5), rt, 1 h, 57%.

2 NMR-spectra

2.1 Compound **2a**

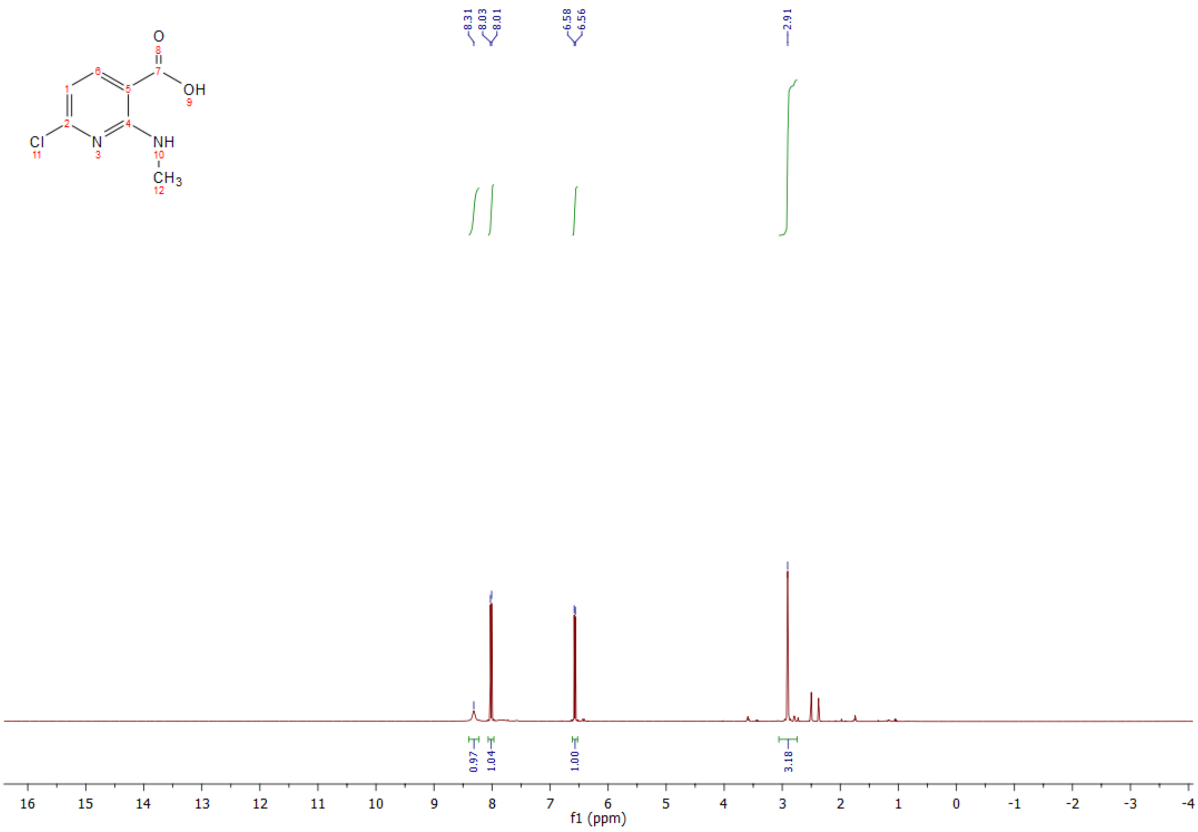

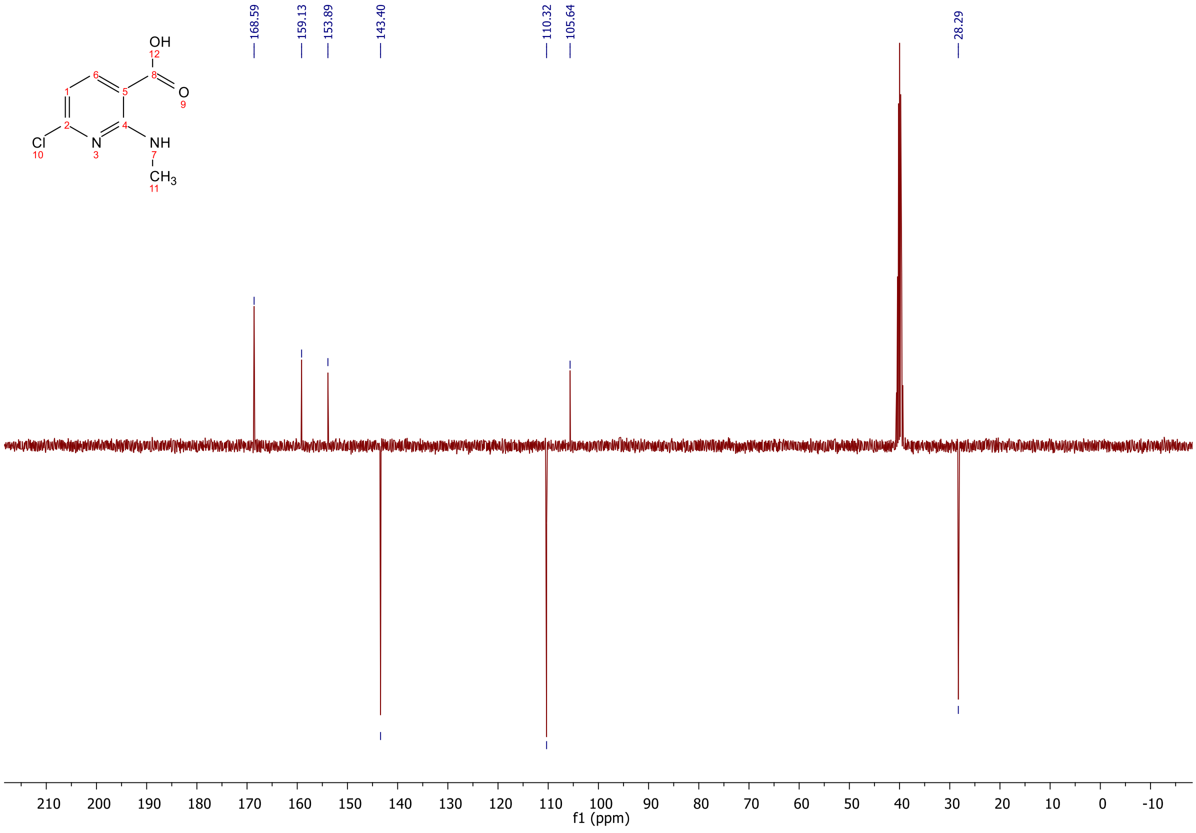

2.2 Compound **2b**

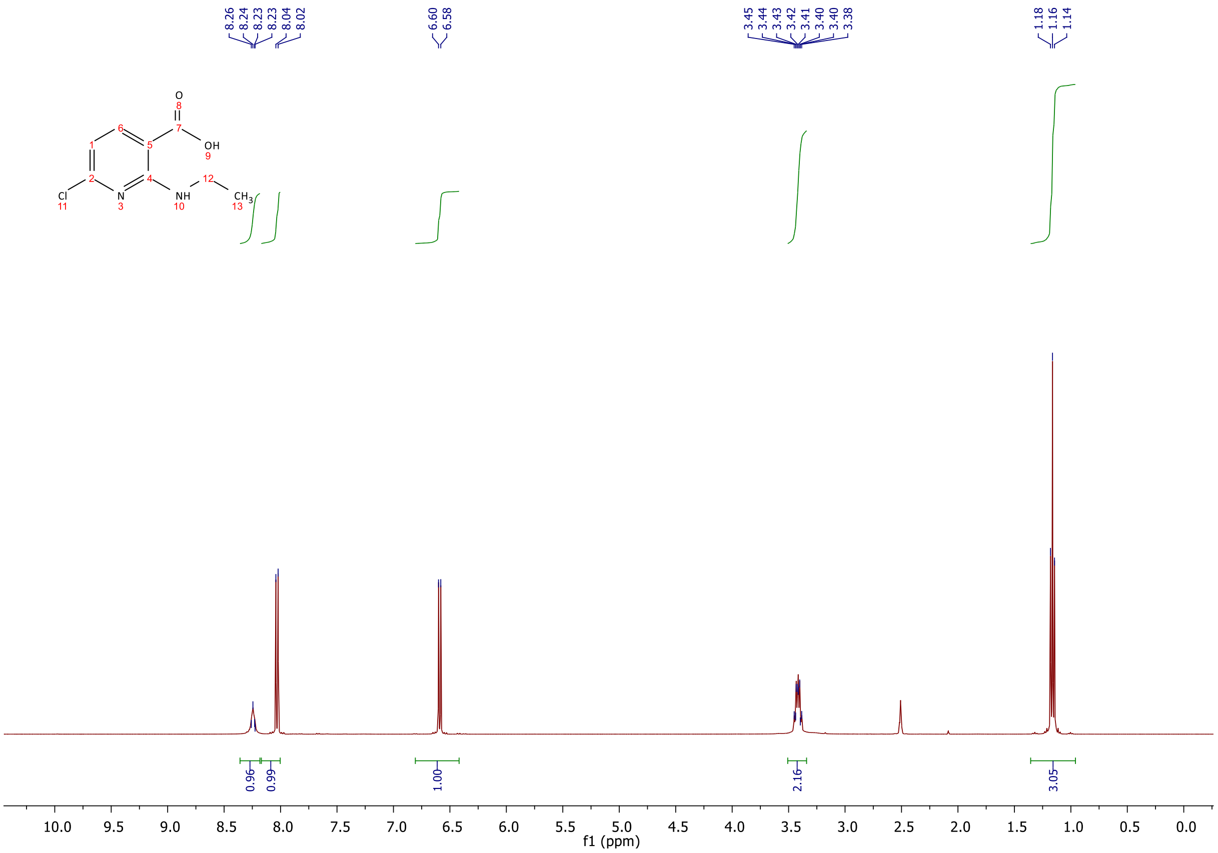

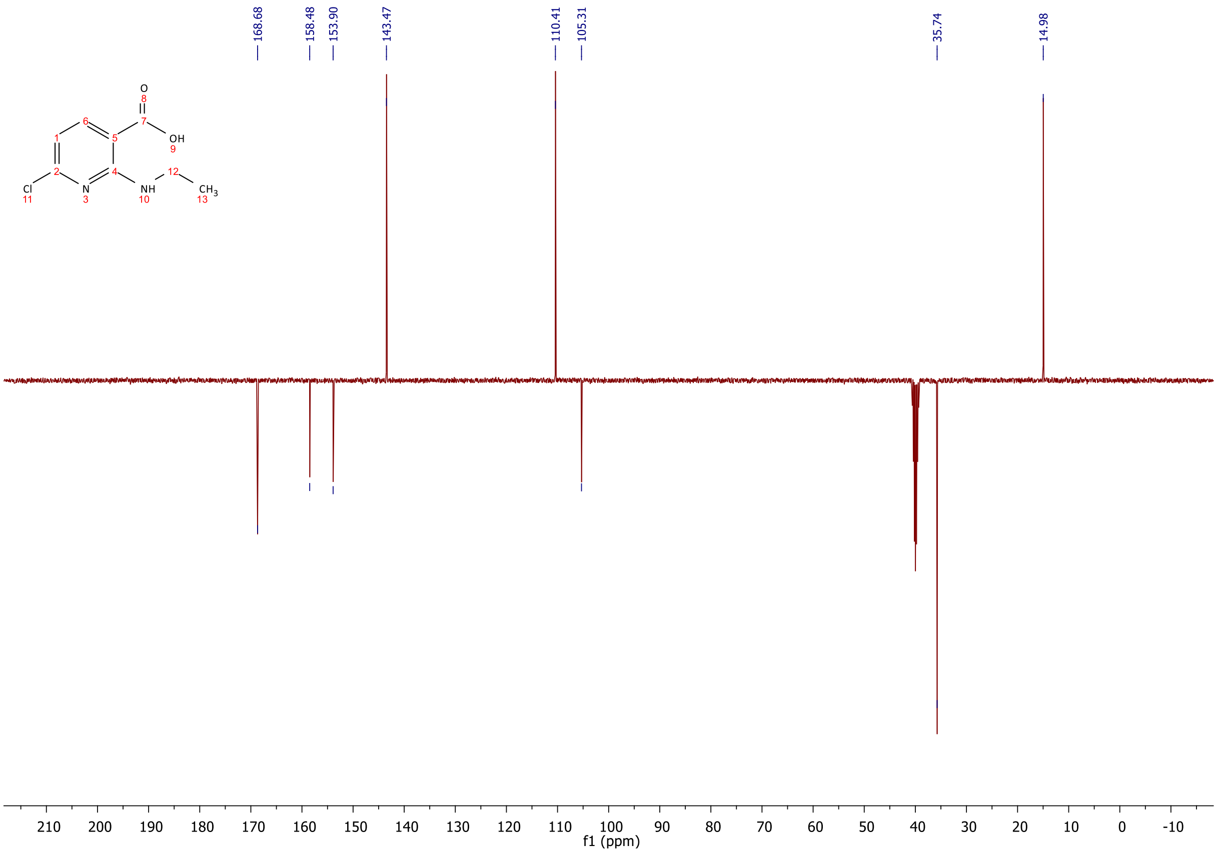

2.3 Compound **2c**

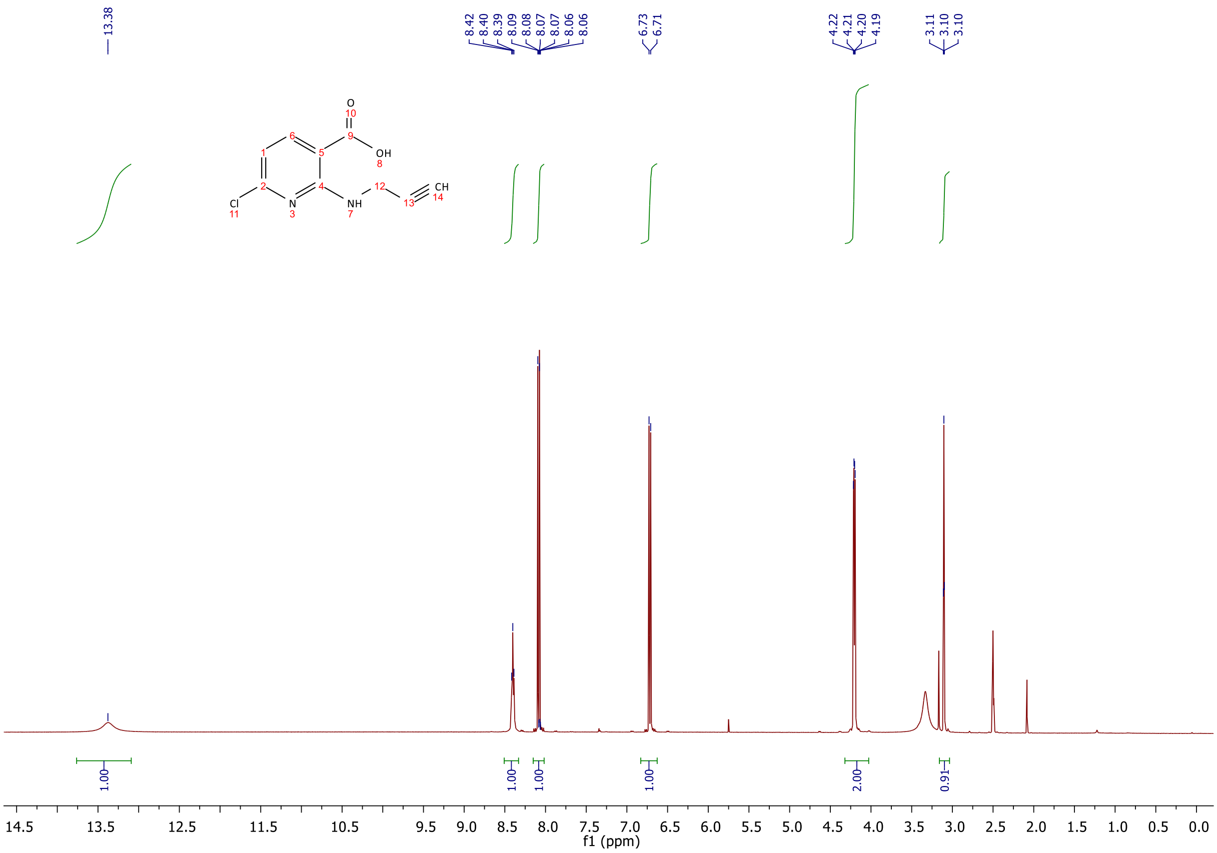

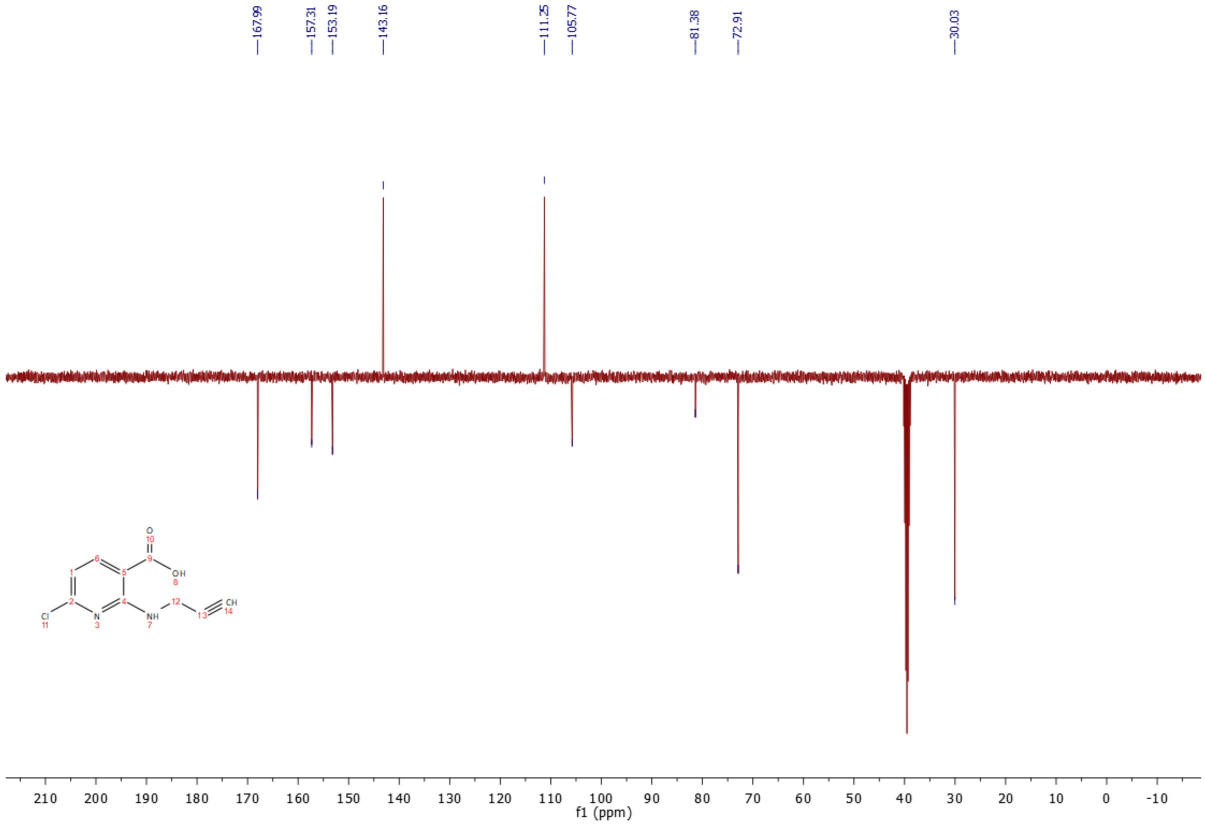

2.4 Compound **2d**

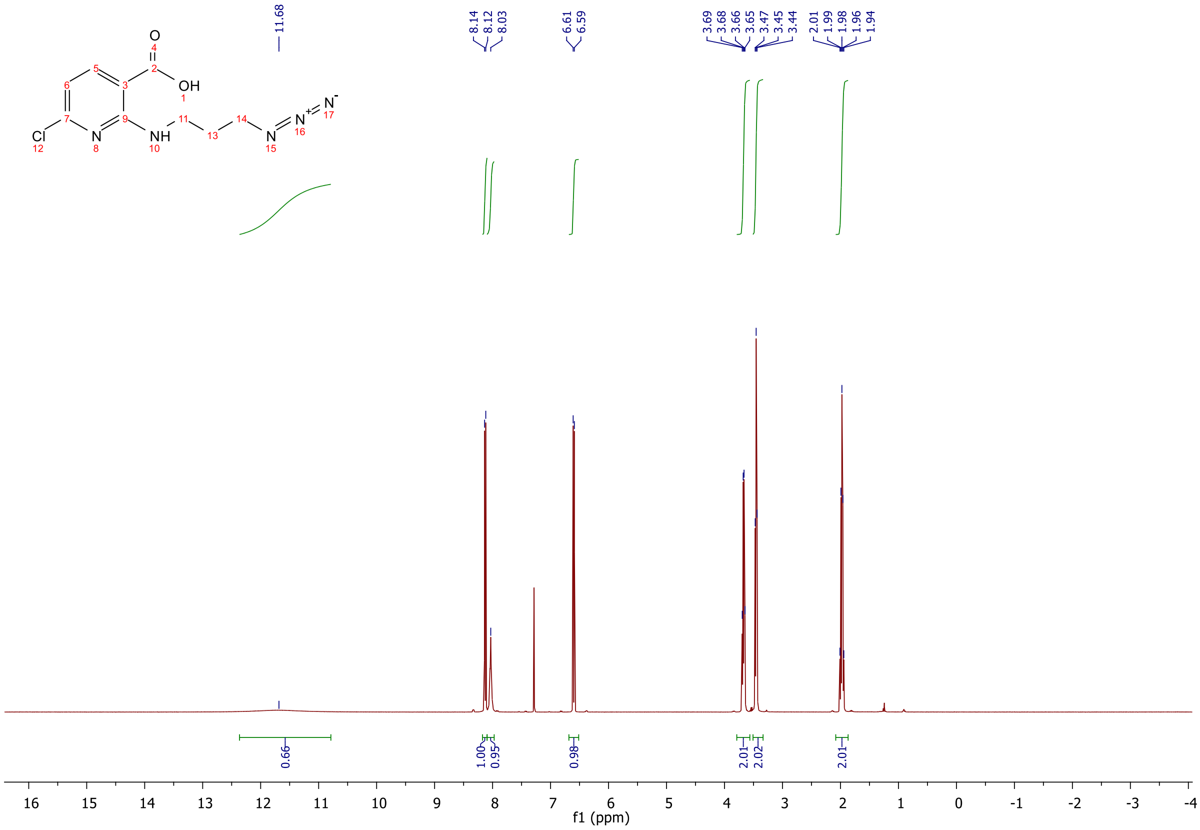

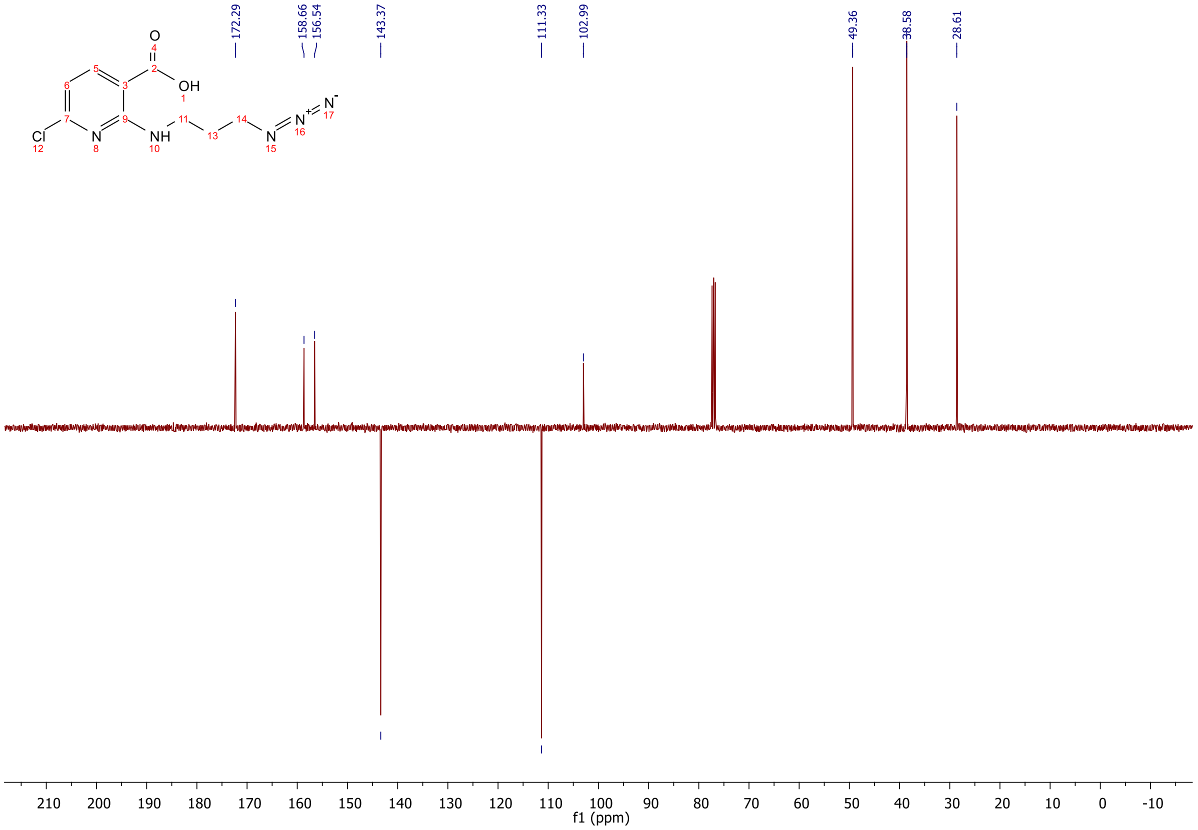

2.5 Compound **2e**

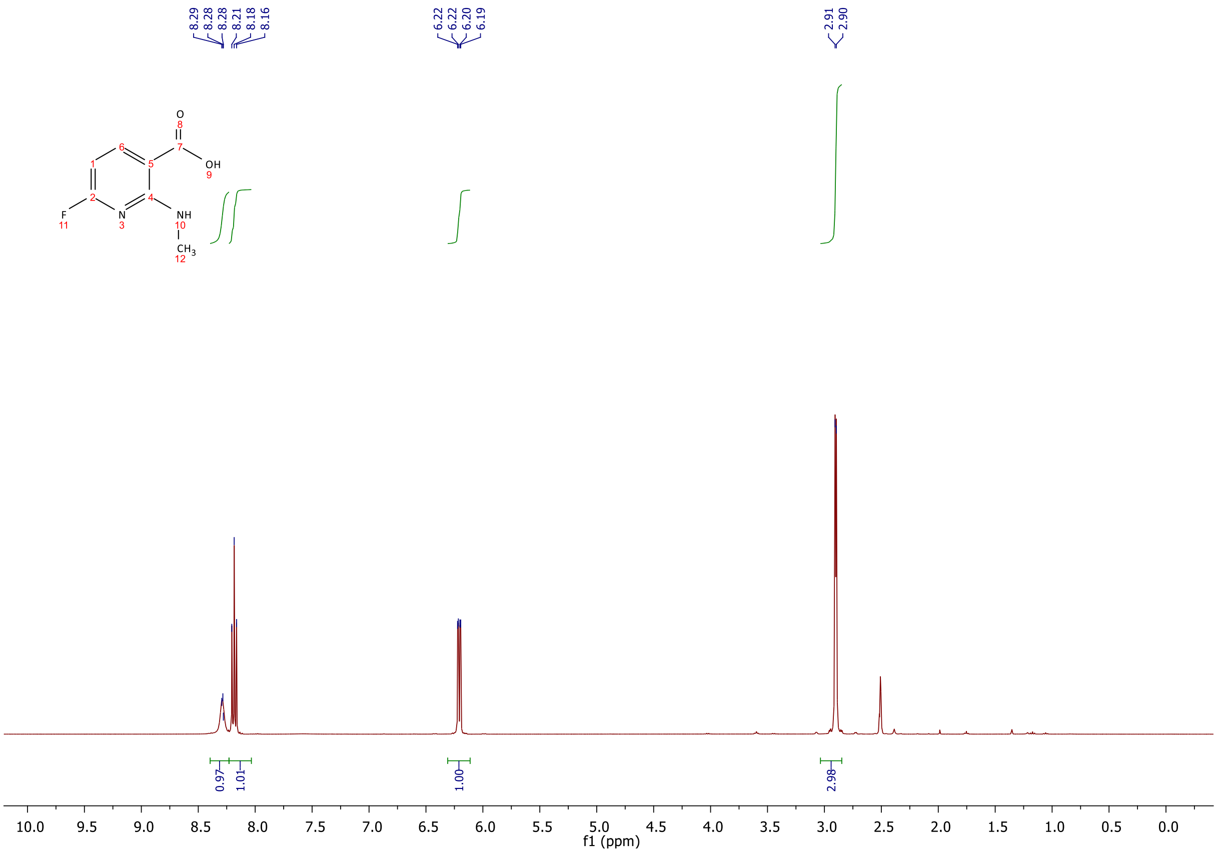

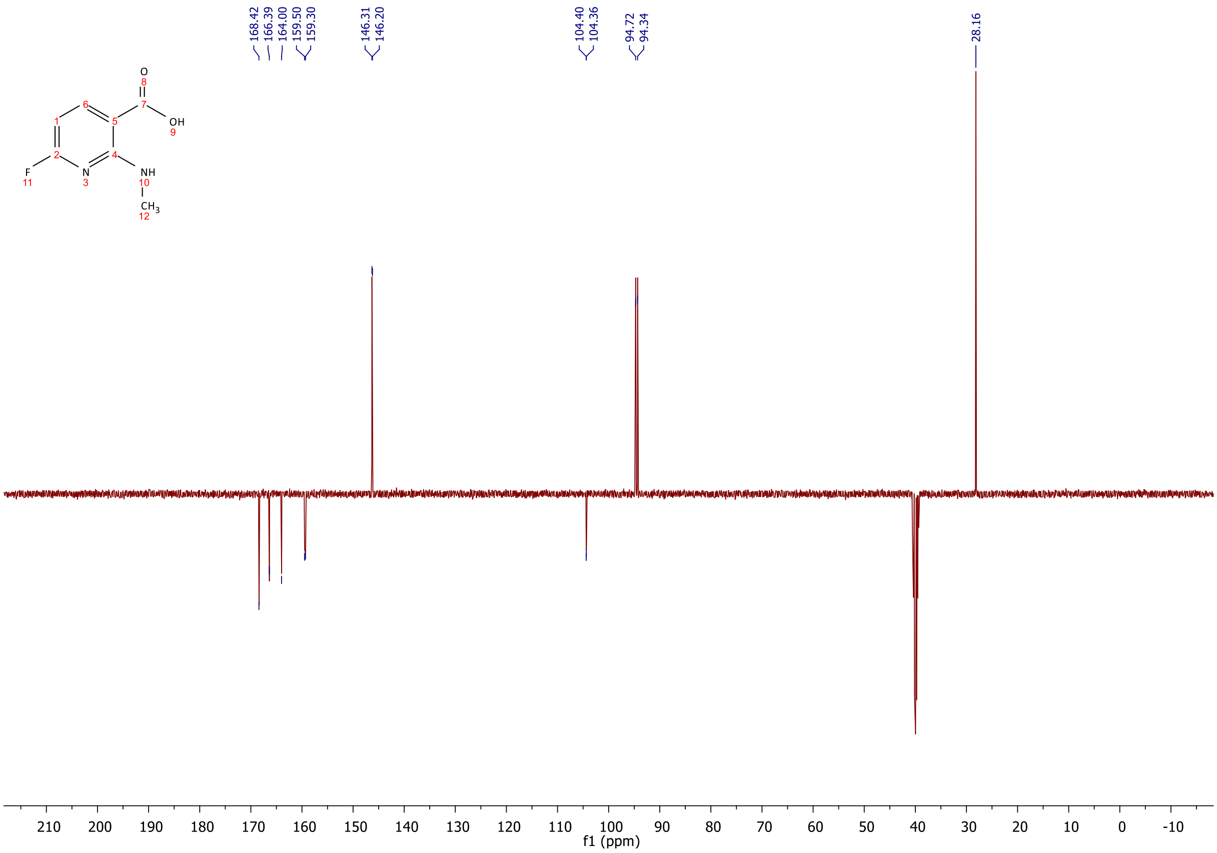

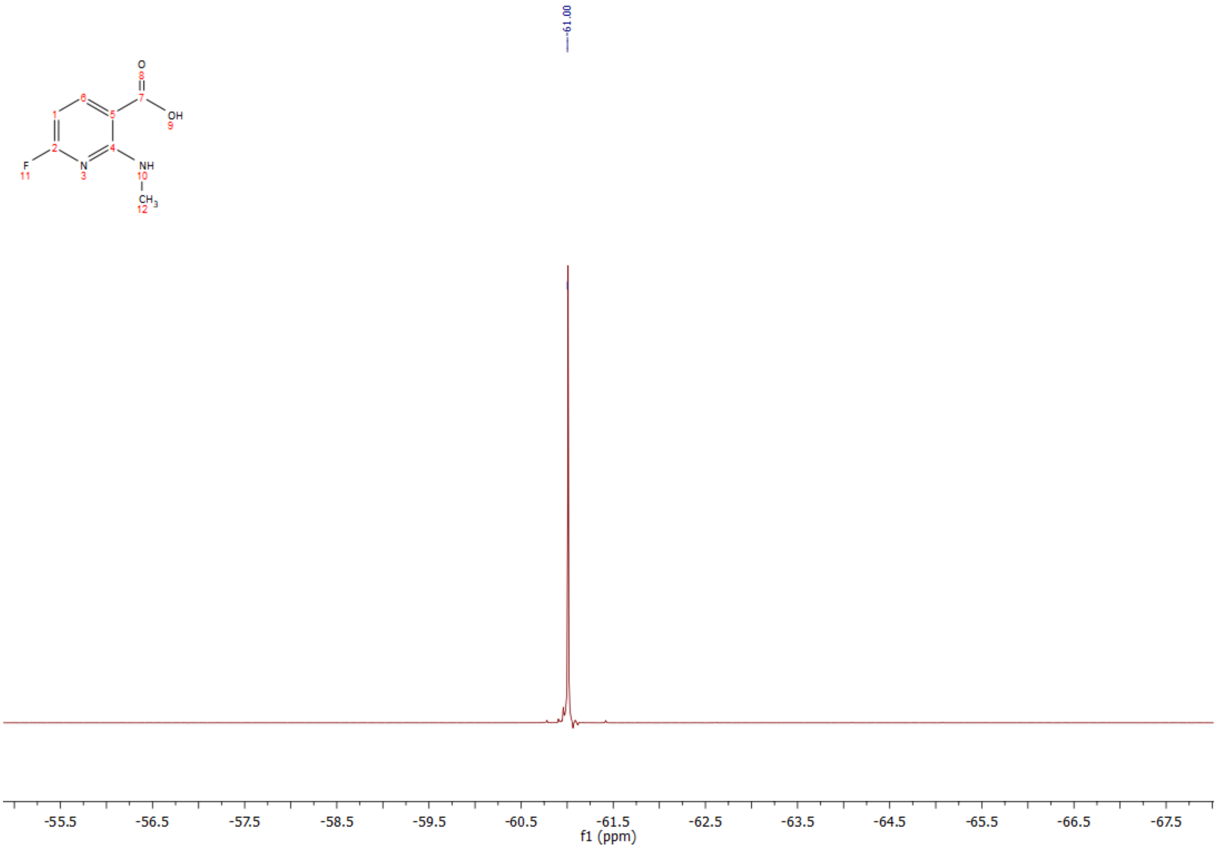
O
OH
F
N
NH
CH3
-61.00
-55.5
-56.5
-57.5
-58.5
-59.5
-60.5
-61.5
-62.5
-63.5
-64.5
-65.5
-66.5
-67.5
f1 (ppm)

2.6 Compound **2f**

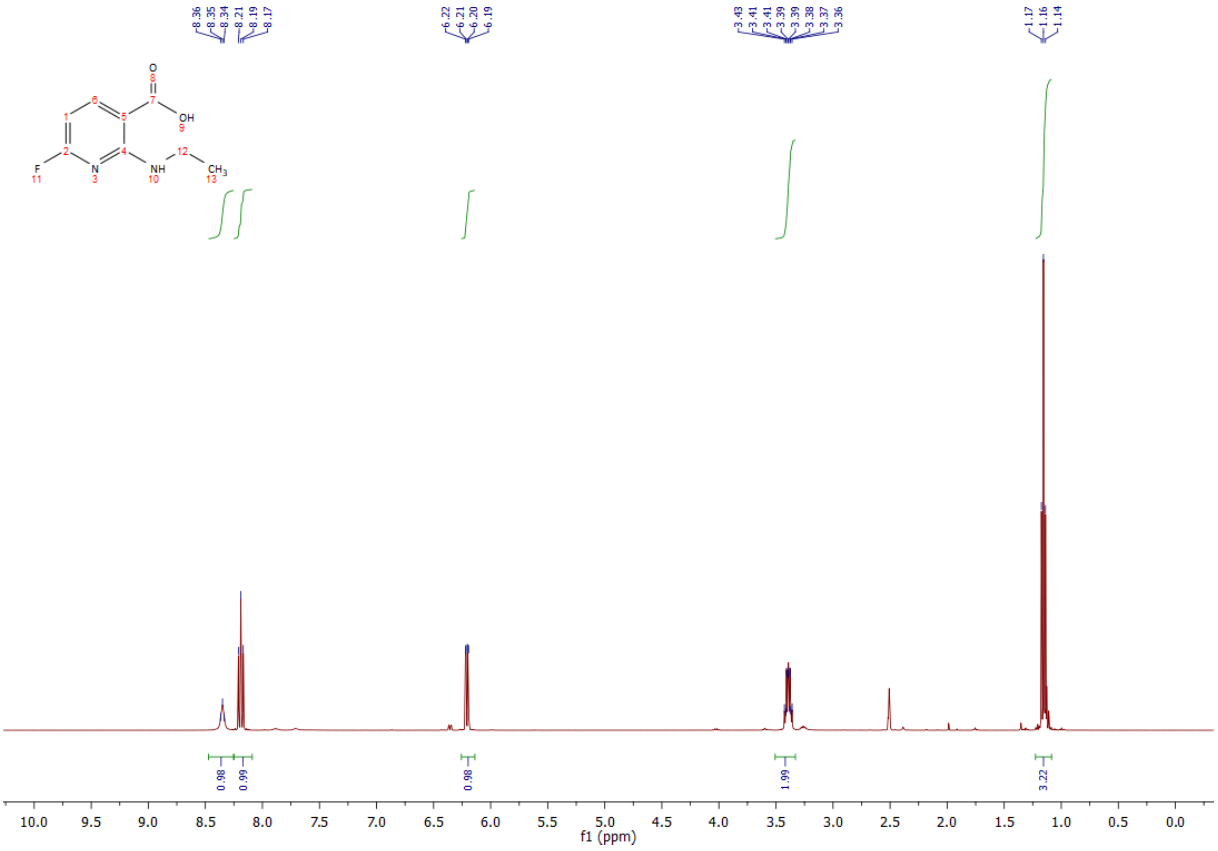

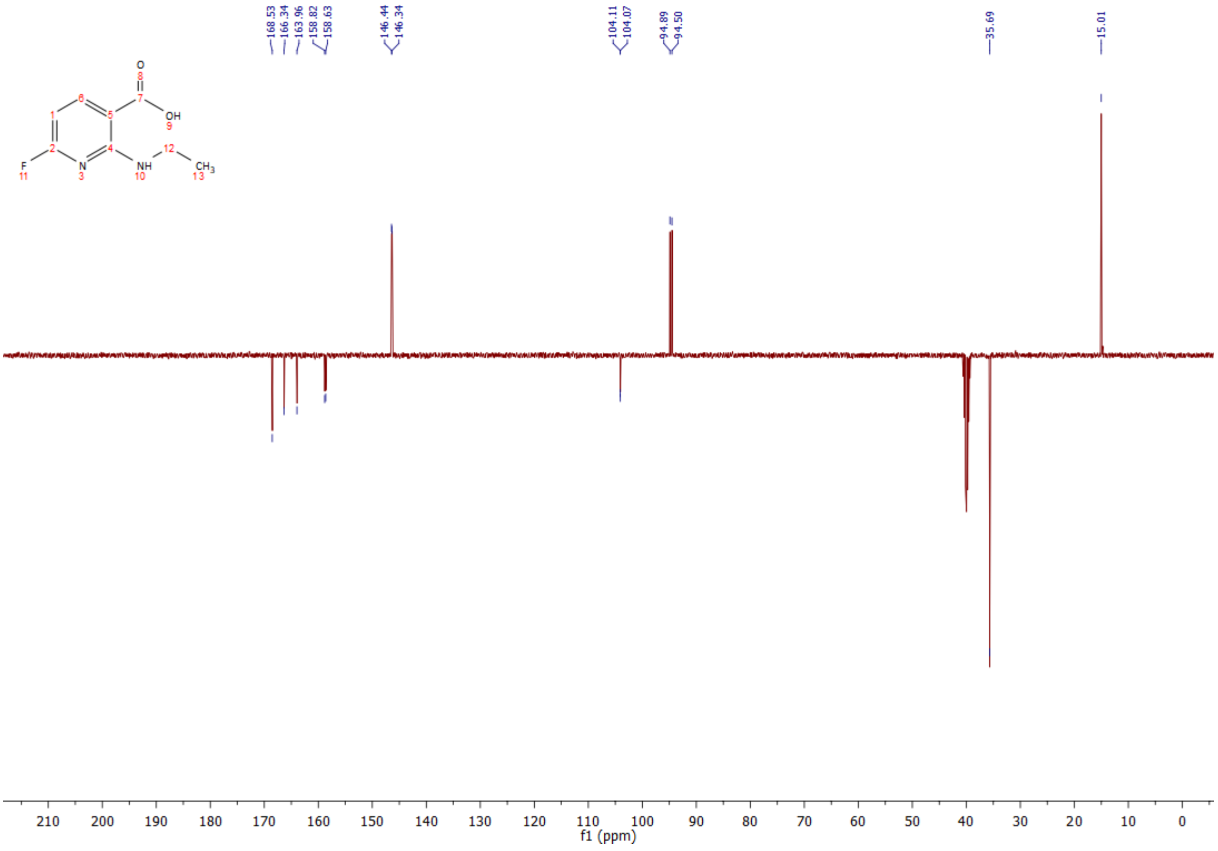

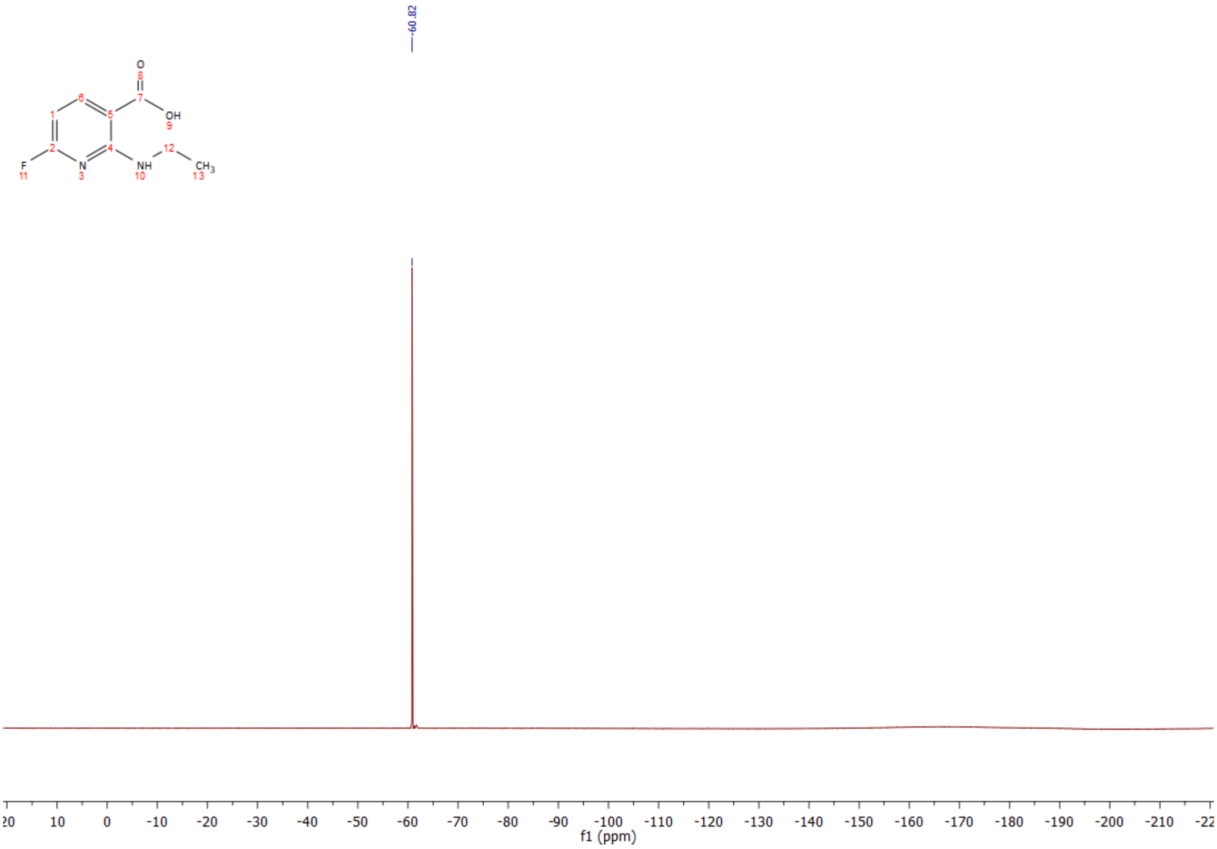
-60.82
O
OH
F
N
NH
CH3
f1 (ppm)

2.7 Compound **2g**

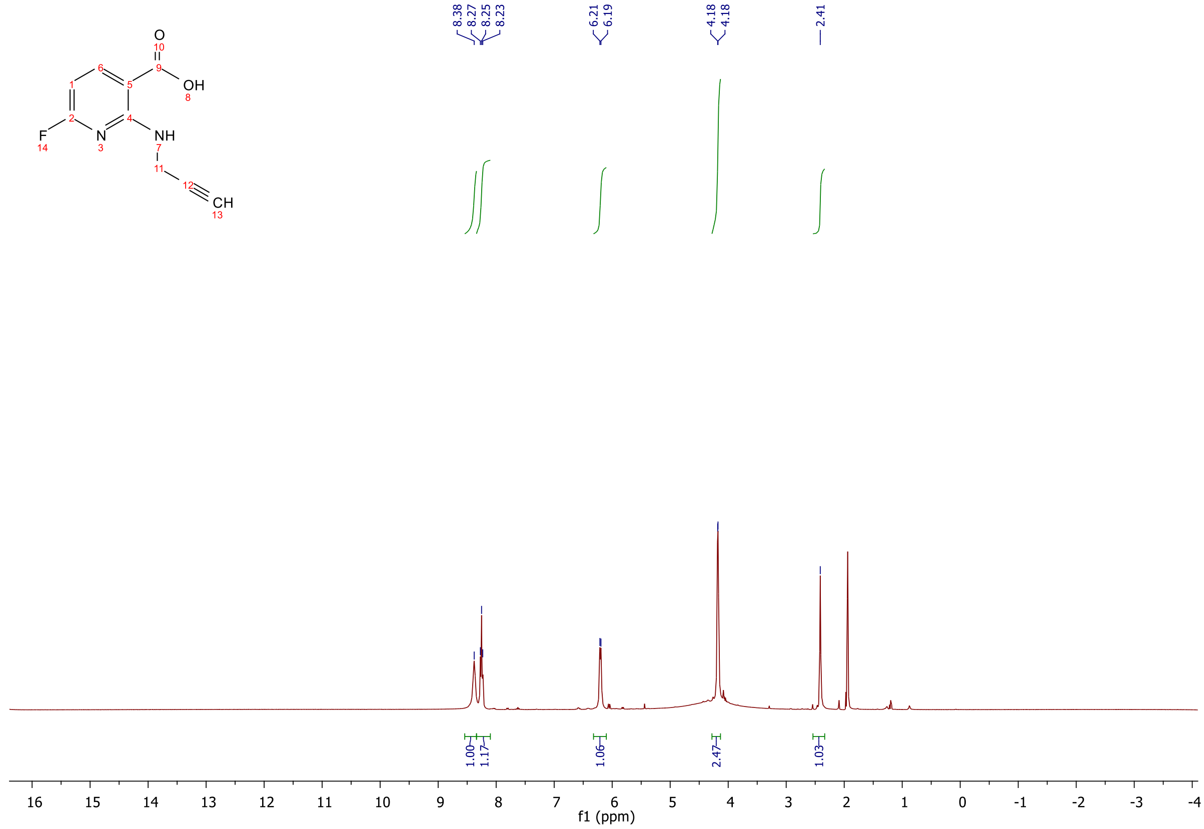

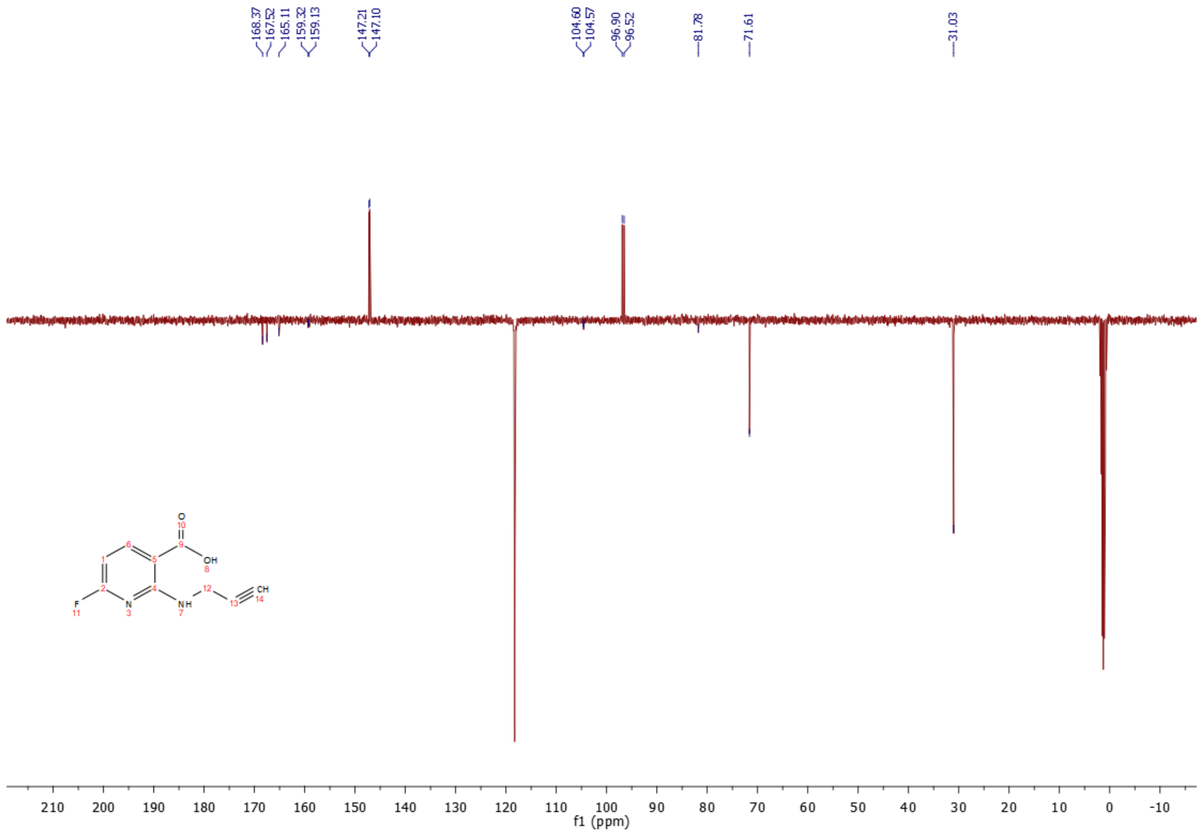

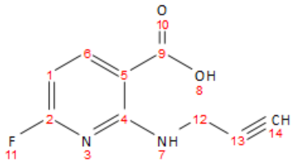
O
10
9
OH
8
6
1
5
2
4
12
CH
14
13
F
11
N
3
NH
7

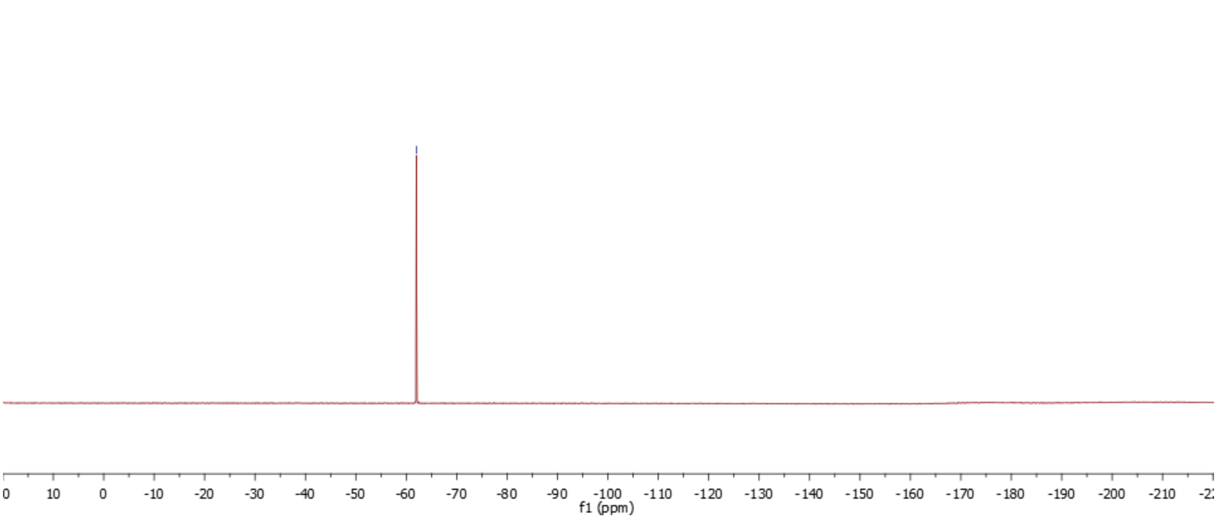
-61.99
f1 (ppm)

2.8 Compound **2h**

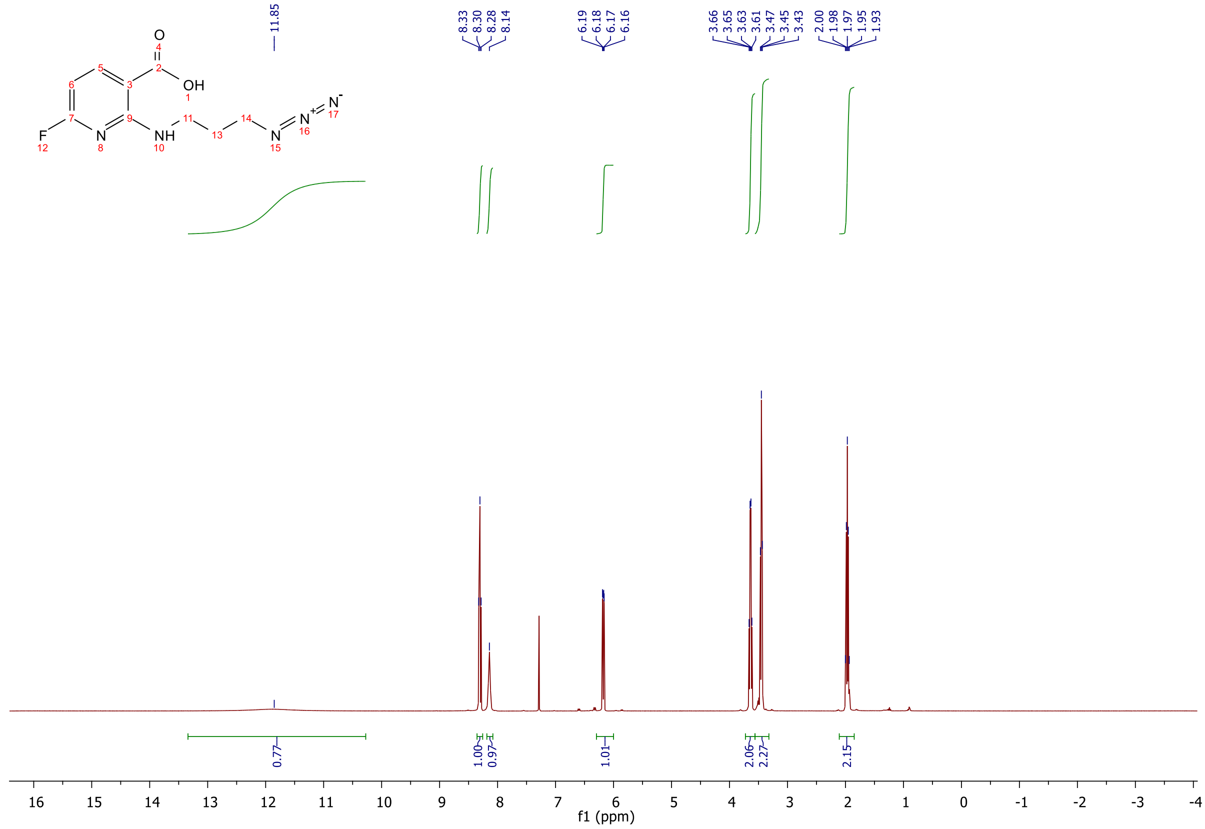

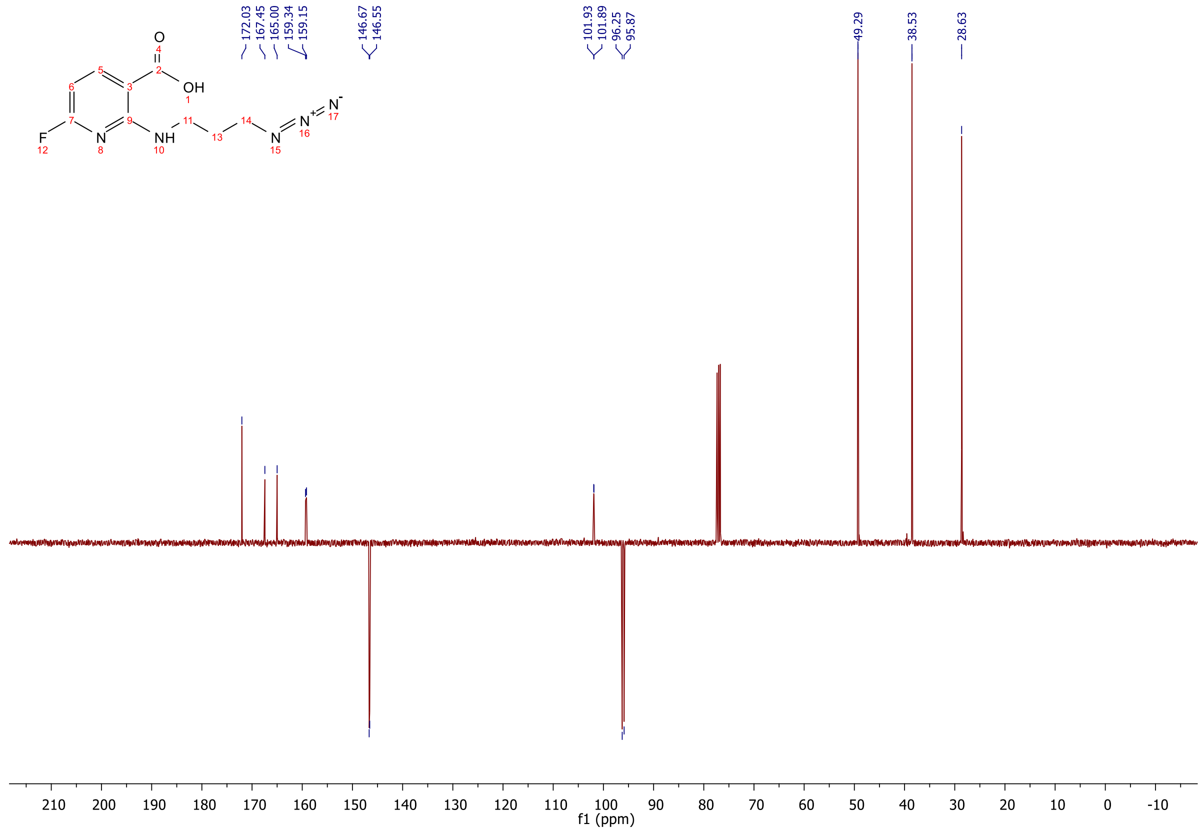

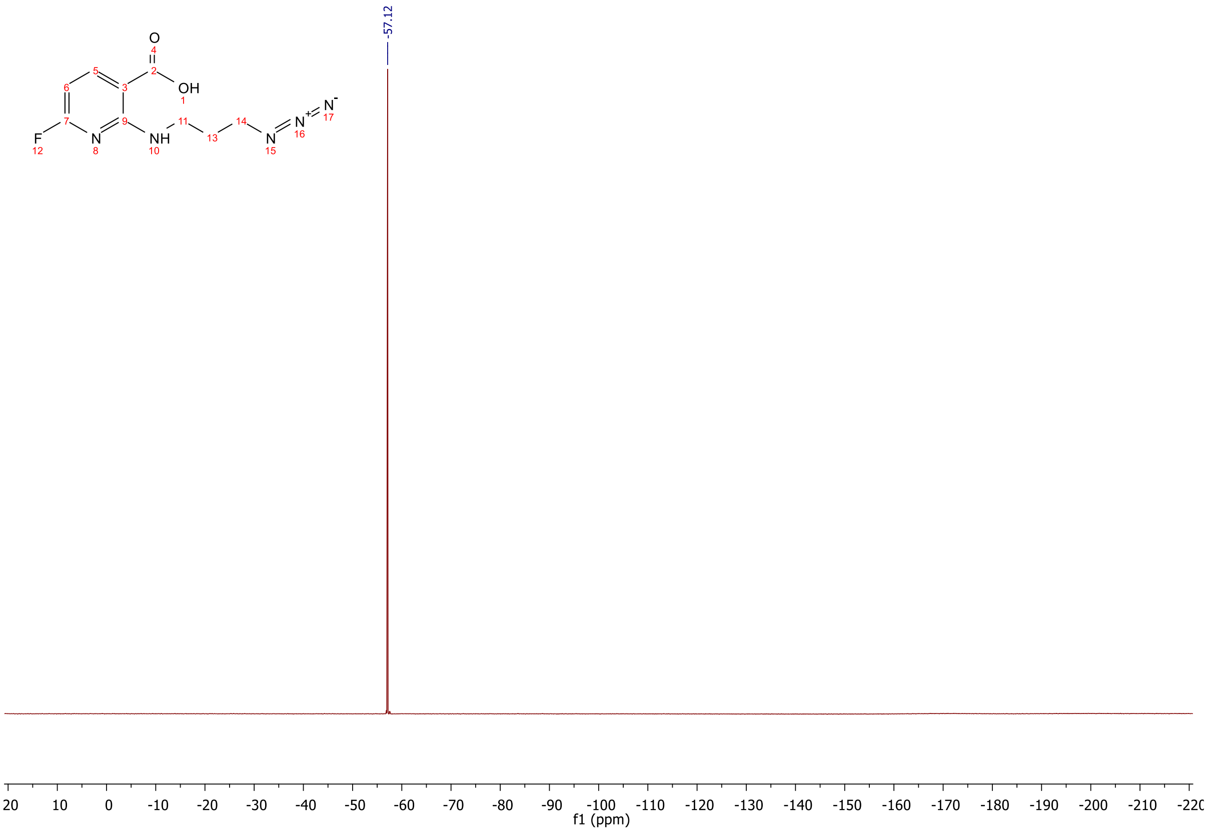
-57.12
O
OH
F
N
NH
N
f1 (ppm)

2.9 Compound **3a**

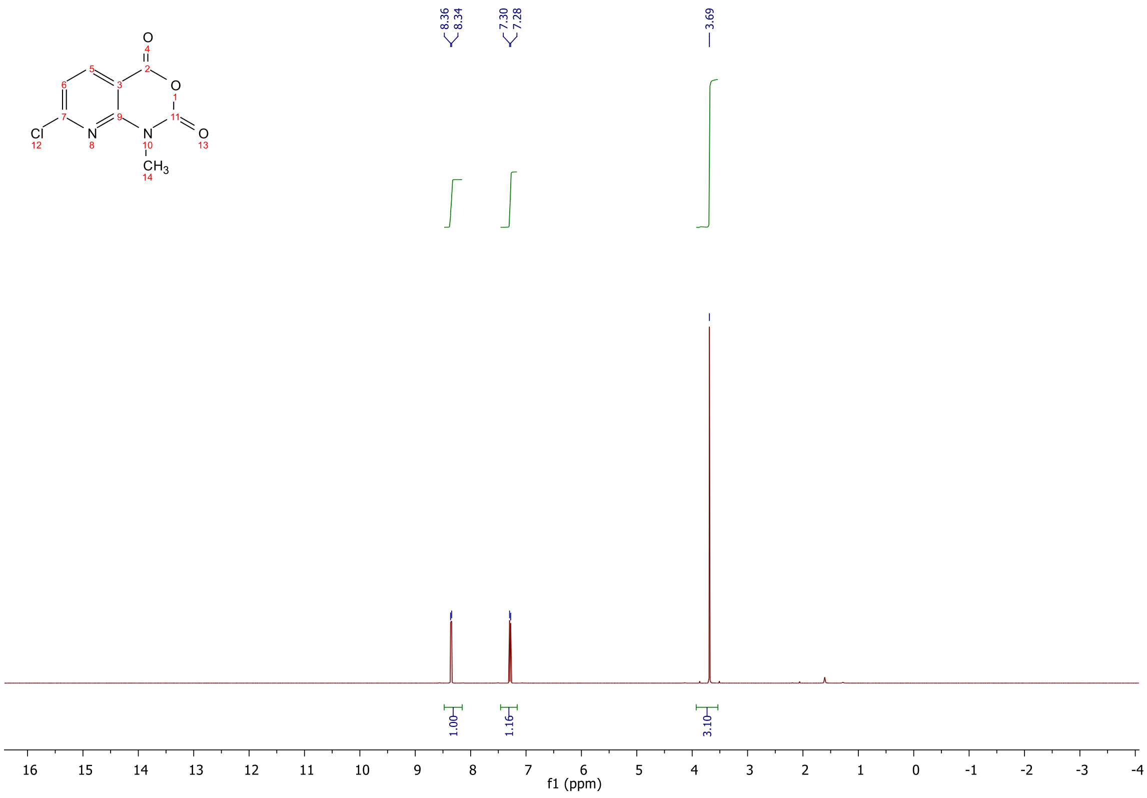

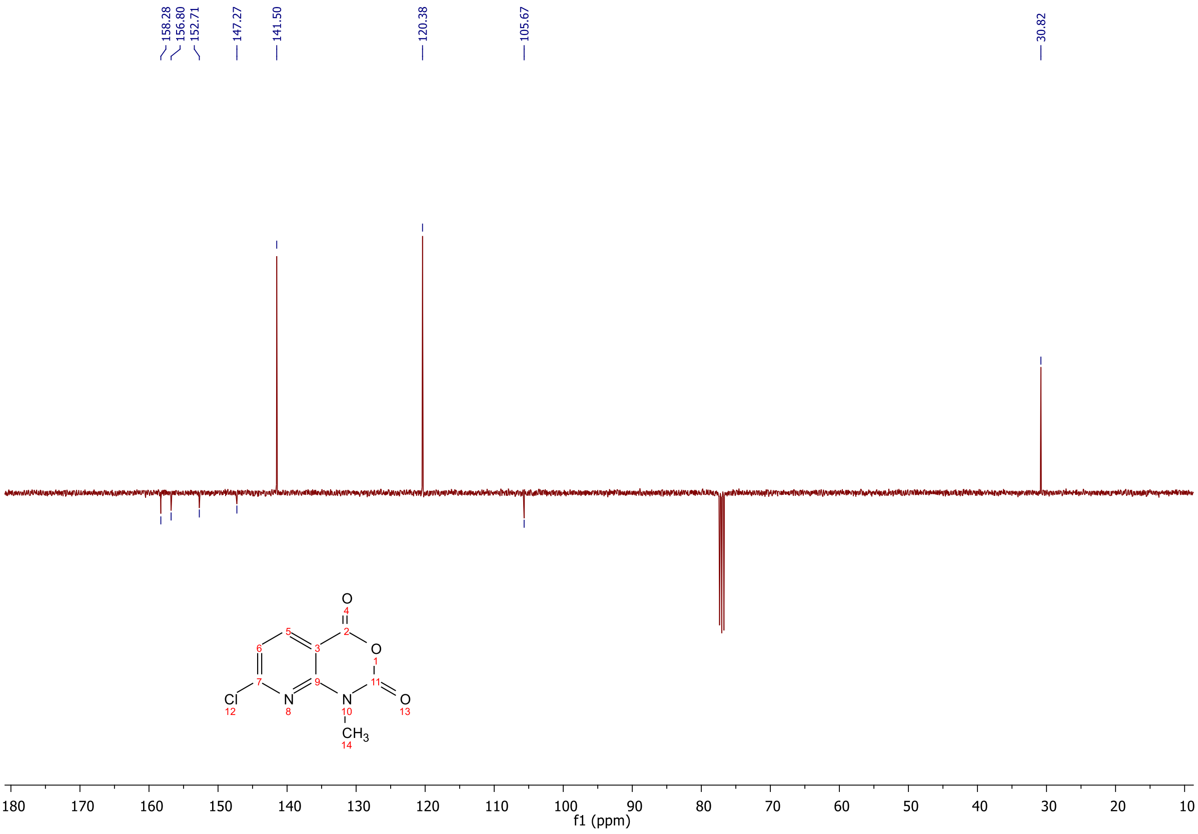

2.10 Compound **3b**

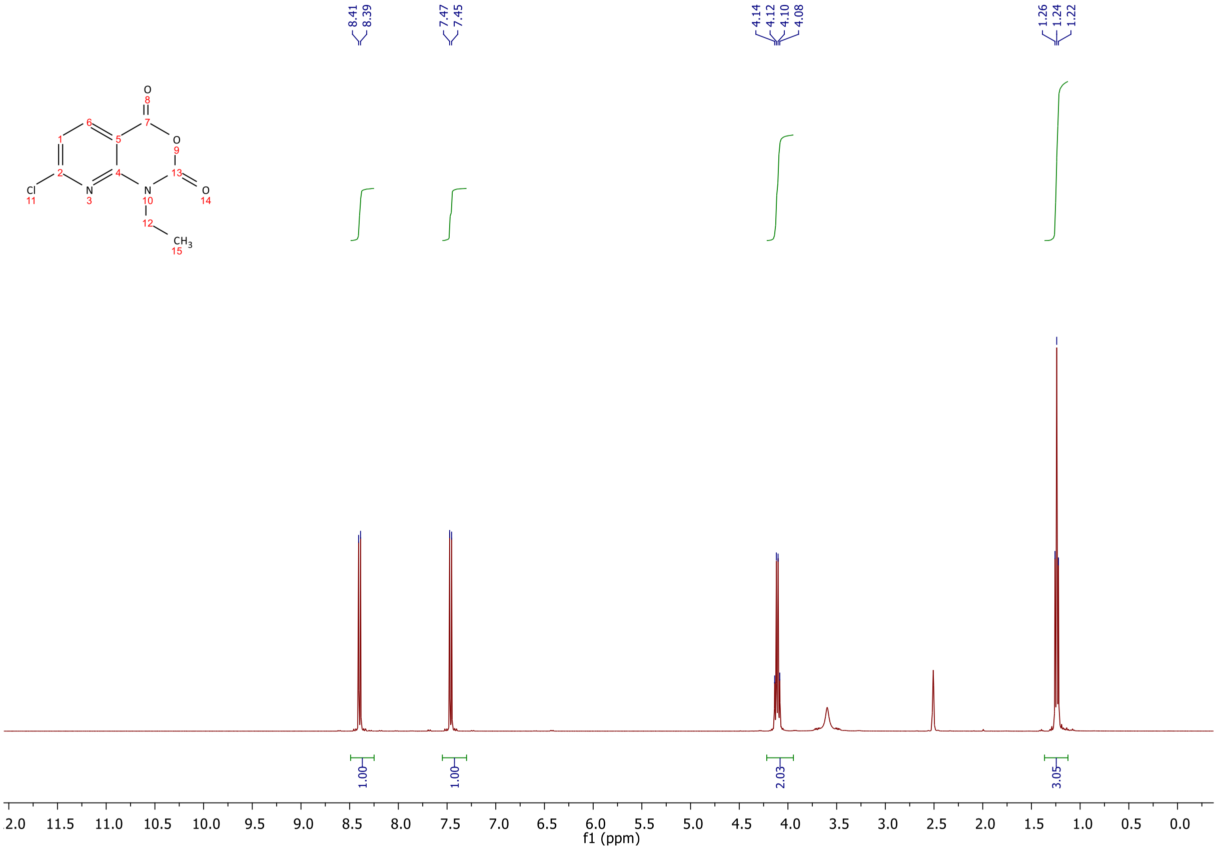

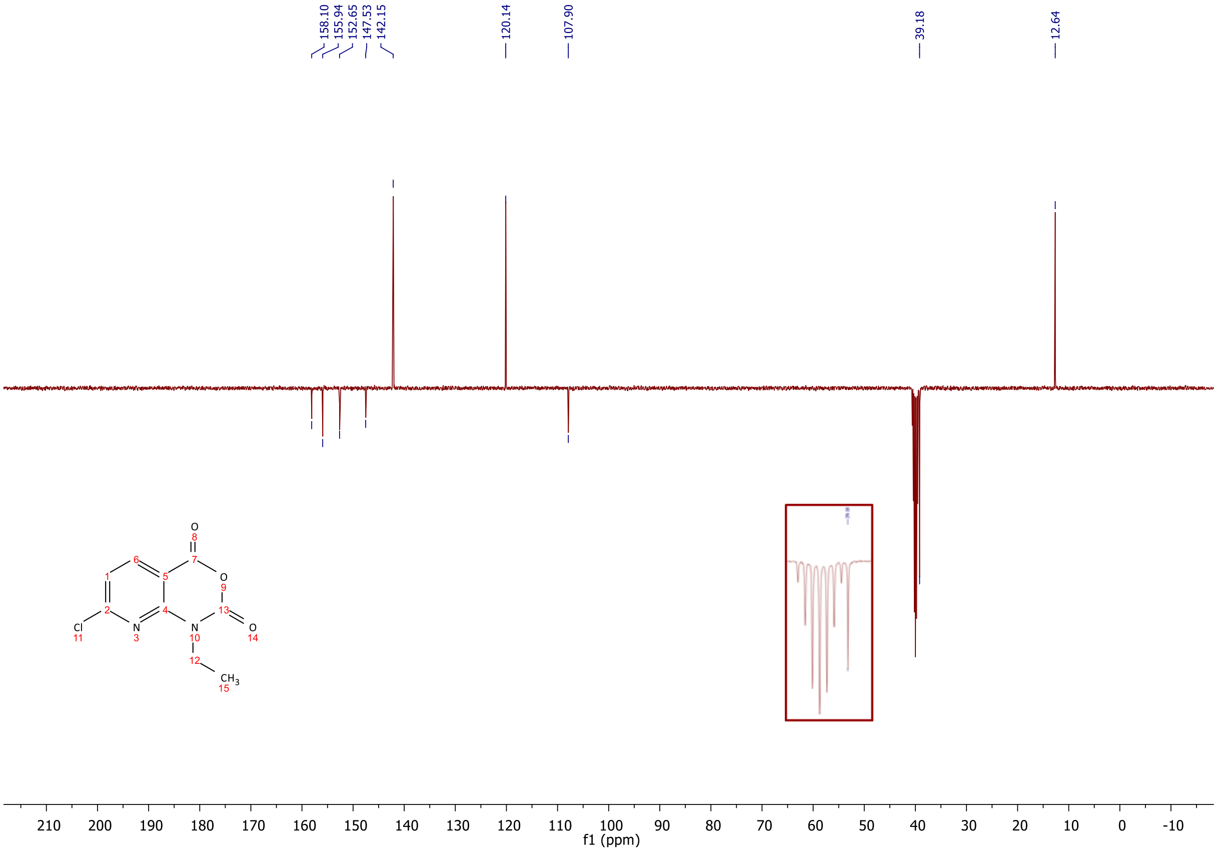

2.11 Compound **3c**

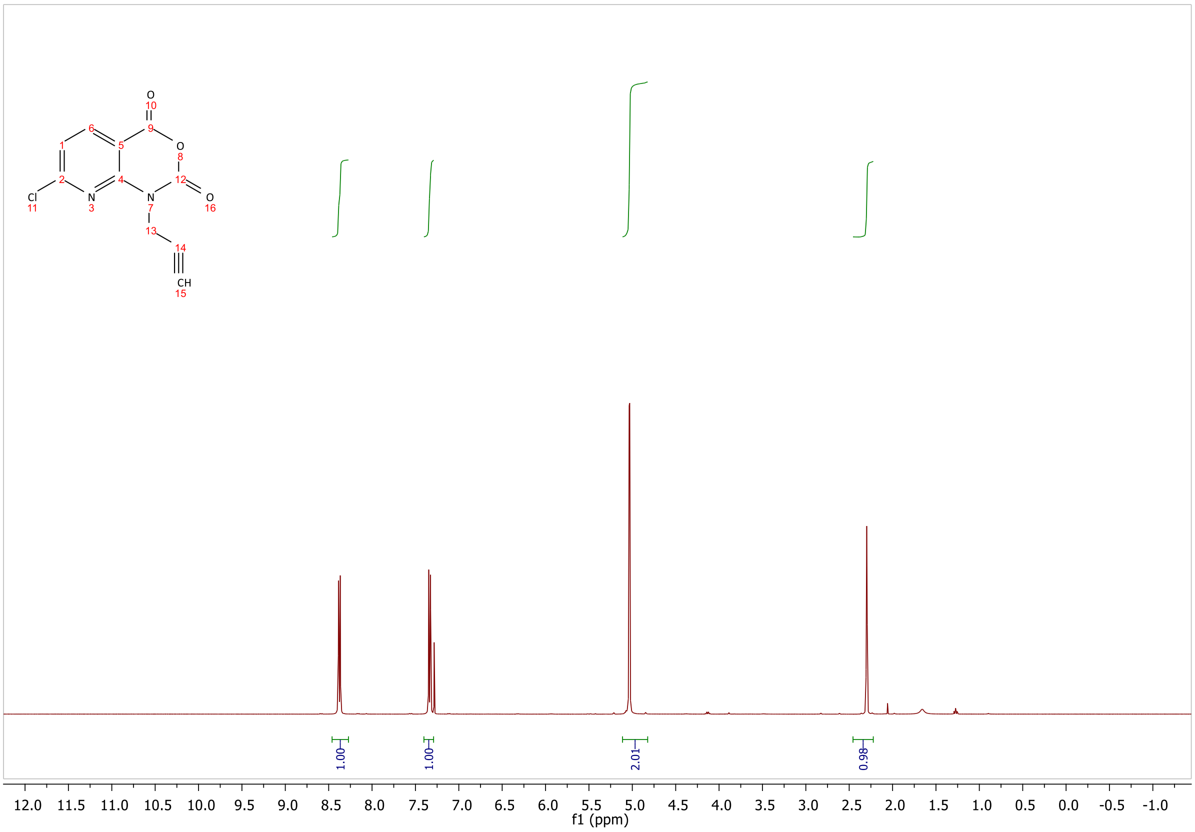

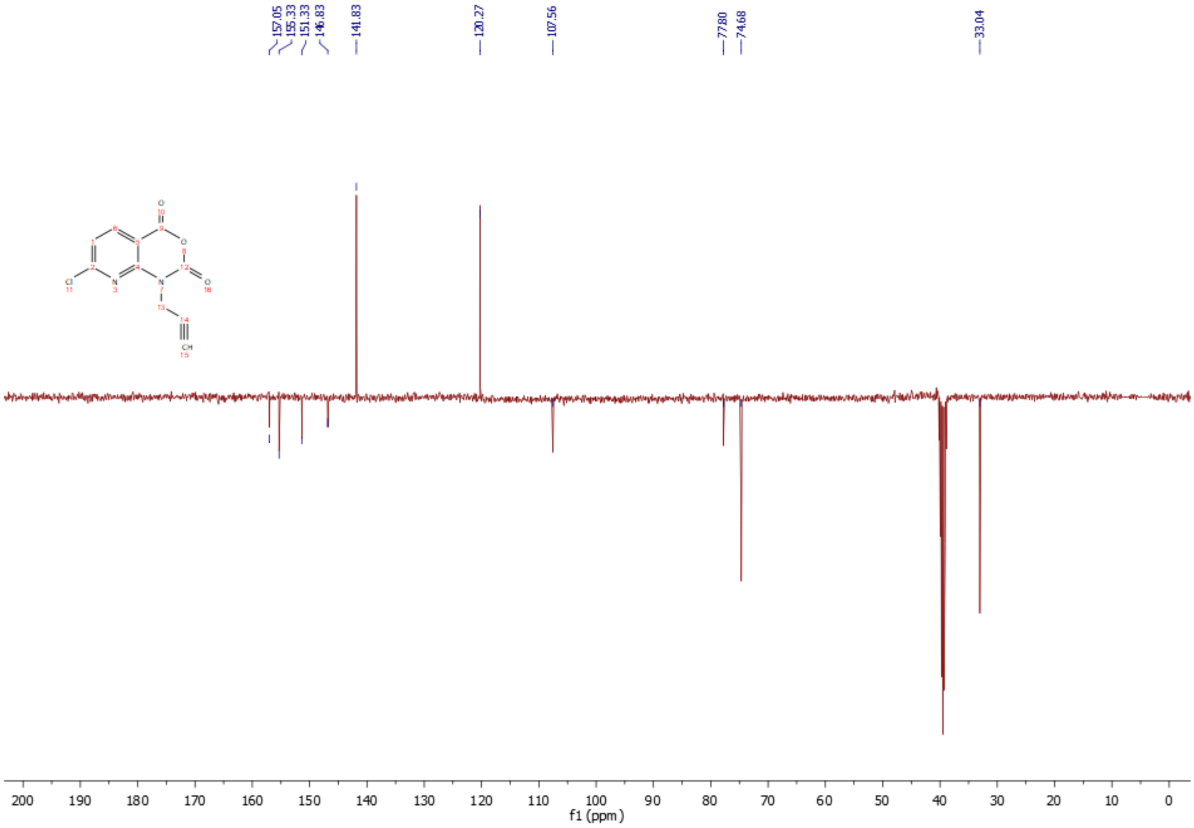

2.12 Compound **3d**

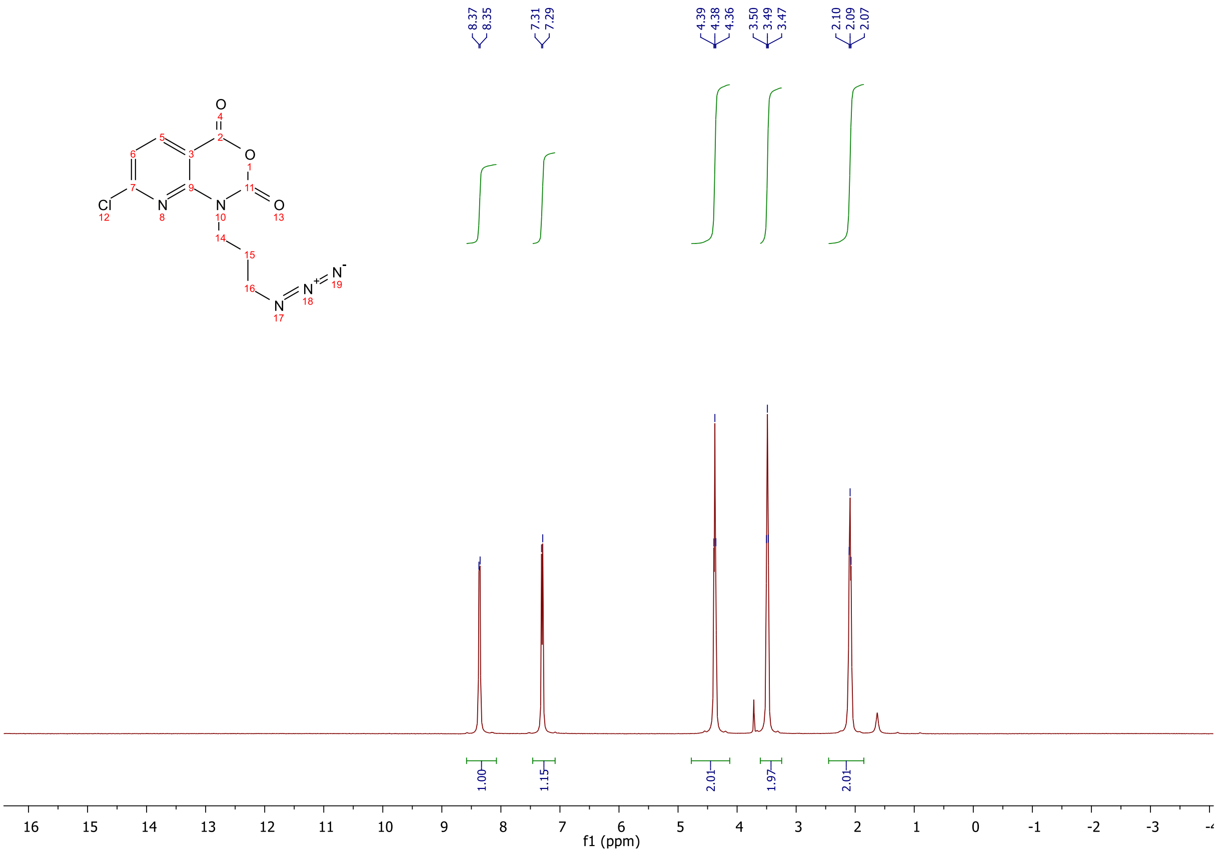

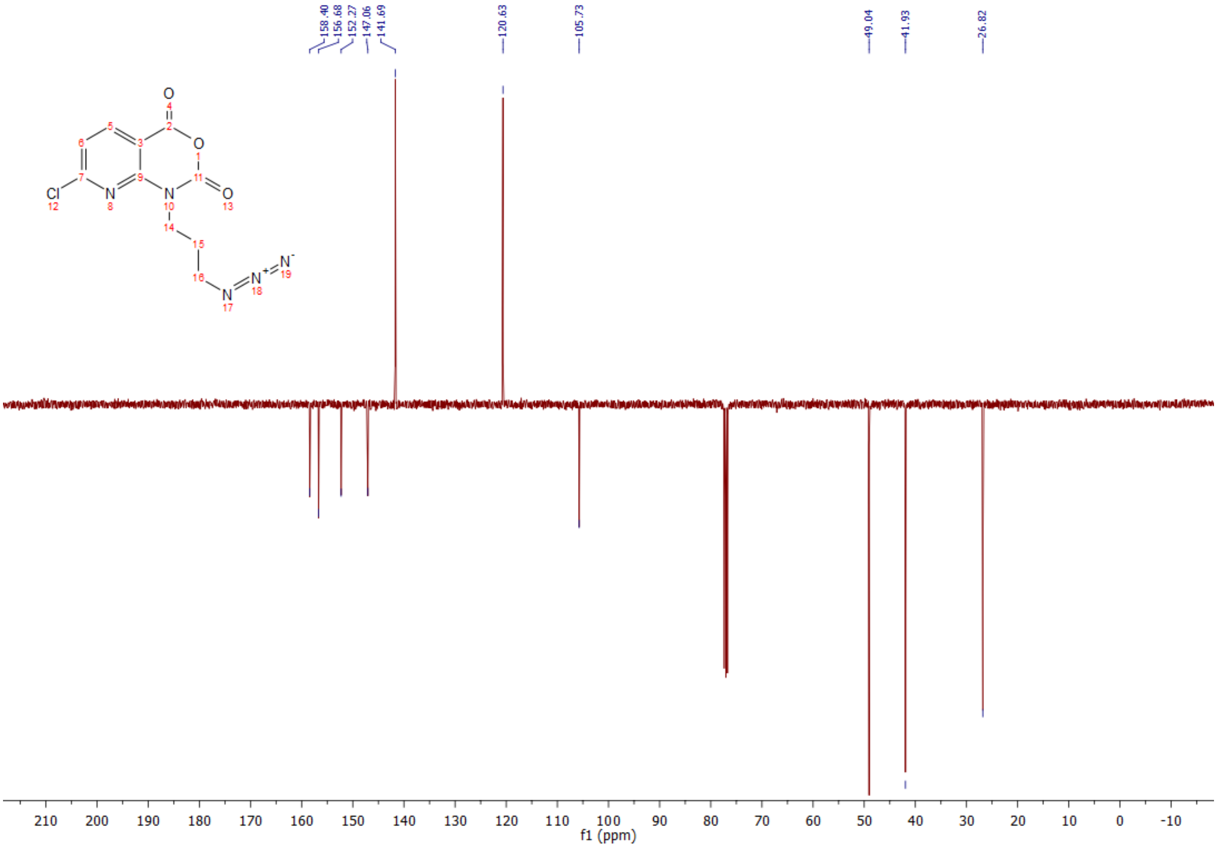

2.13 Compound **3e**

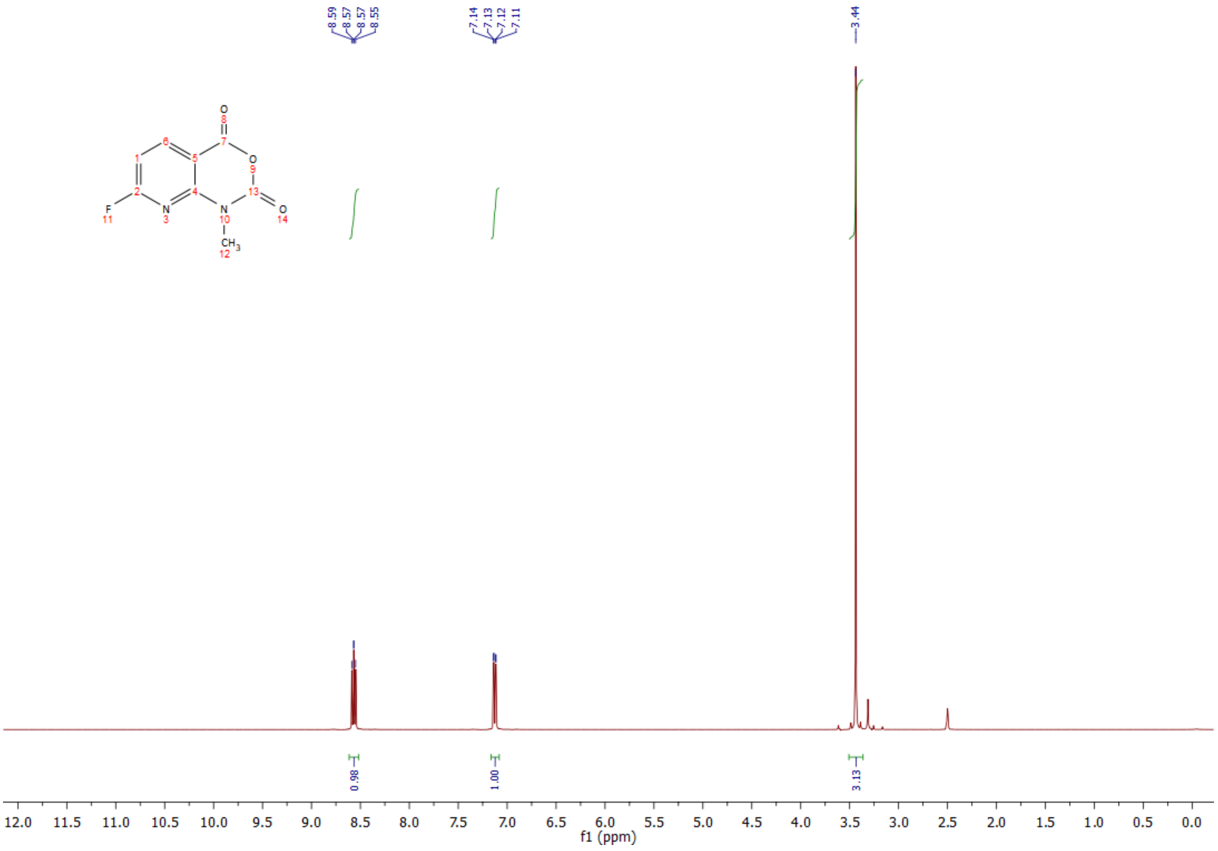

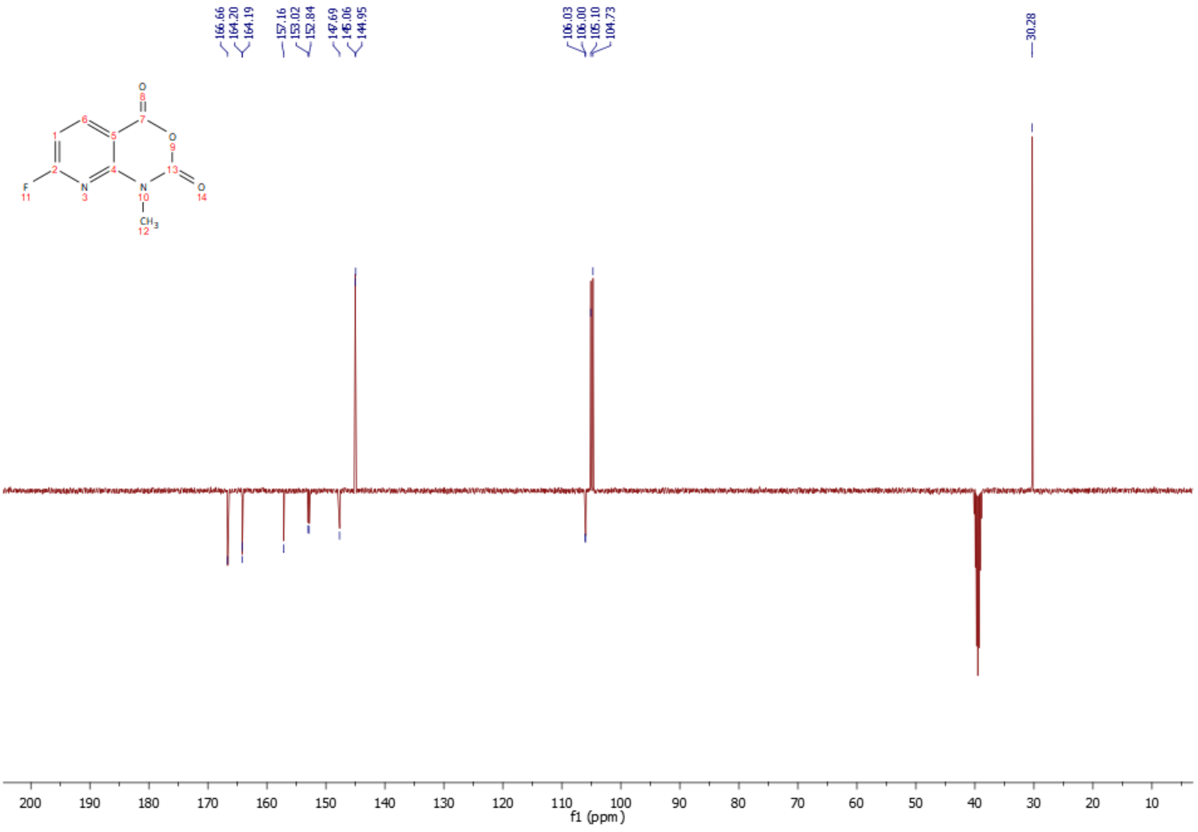

^{19}F NMR (376 MHz, DMSO) δ -56.82.

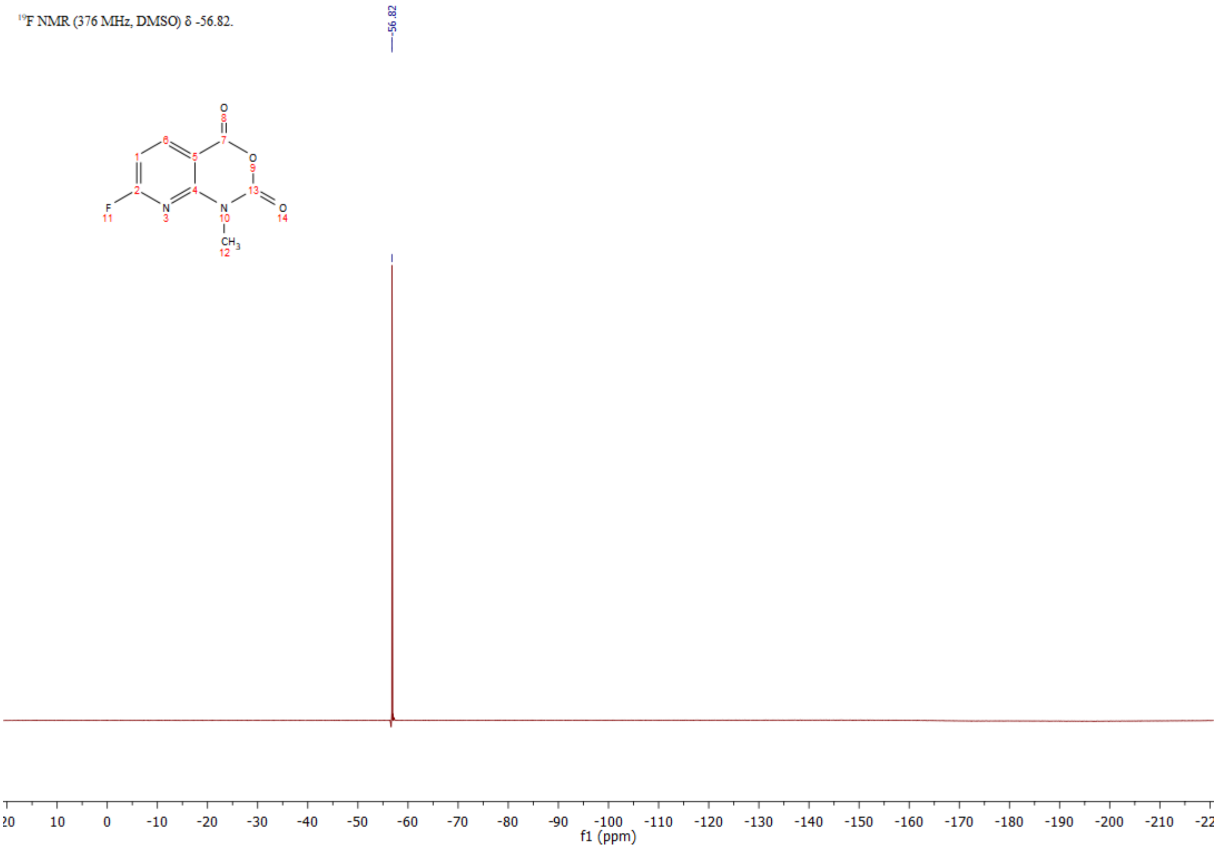

2.14 Compound **3f**

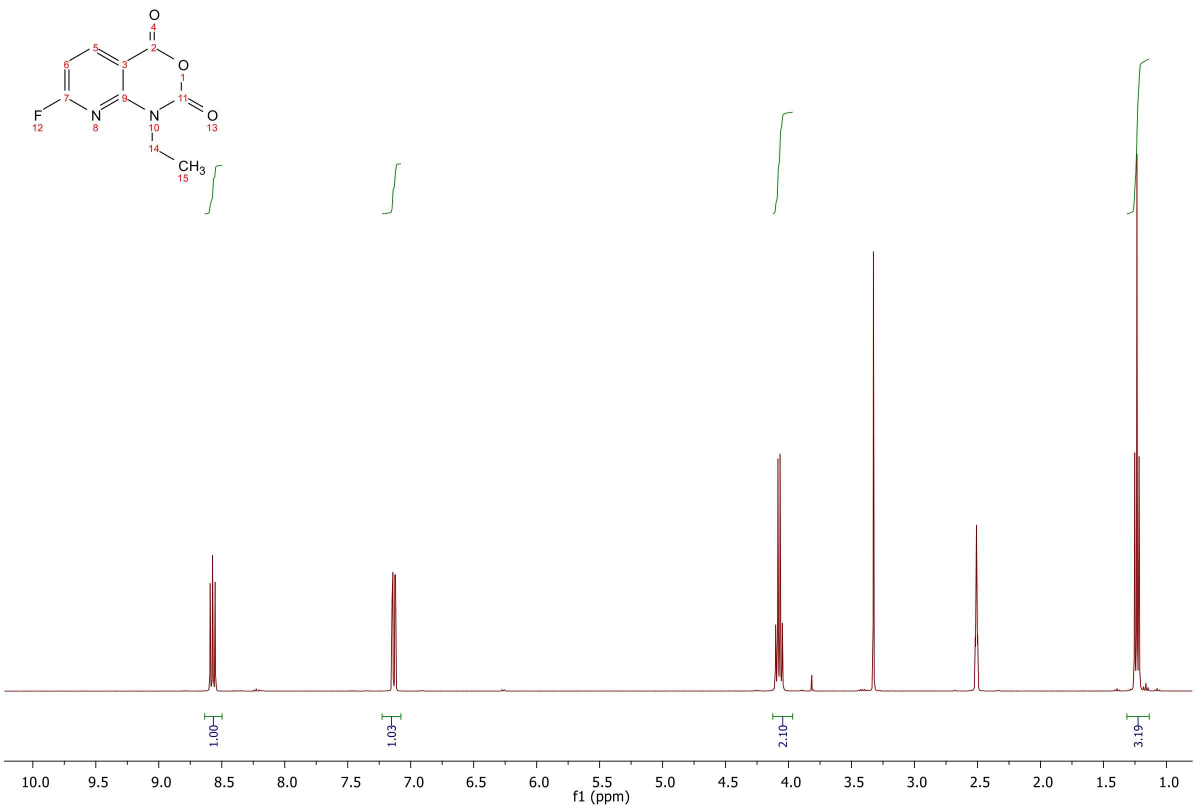

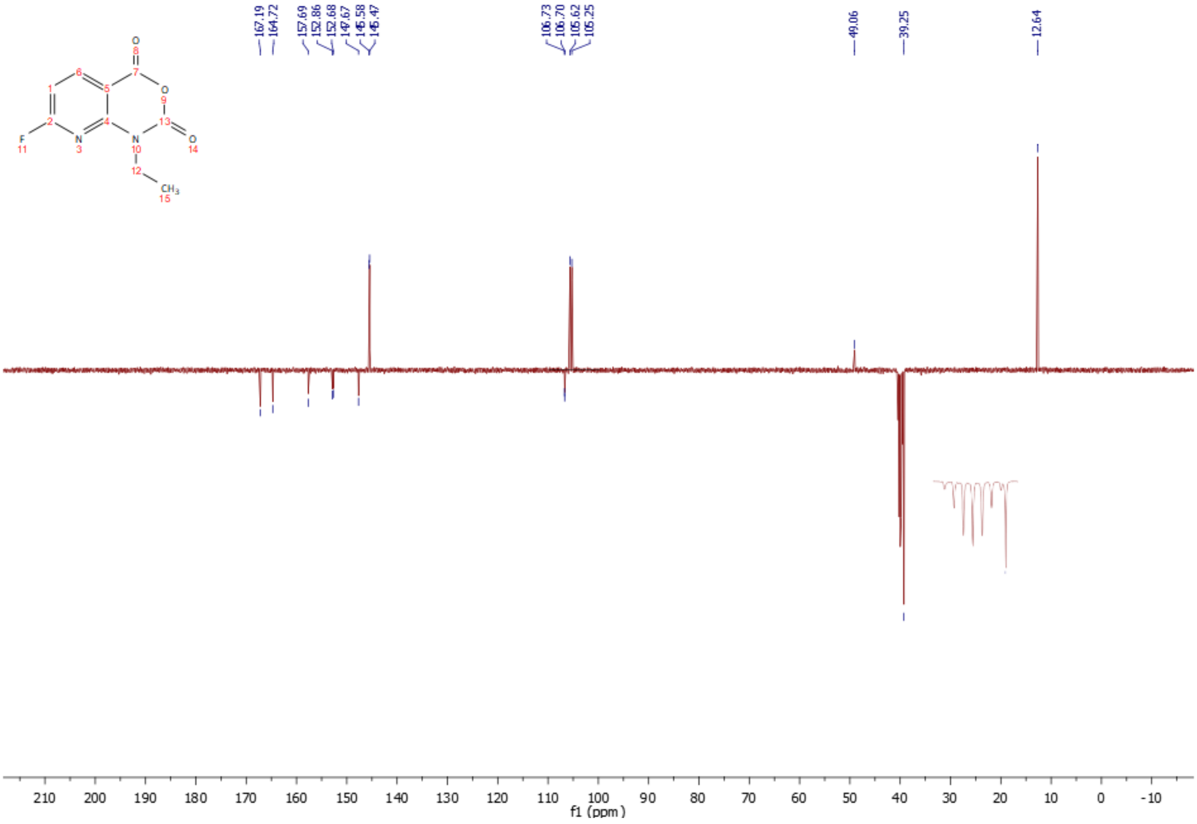

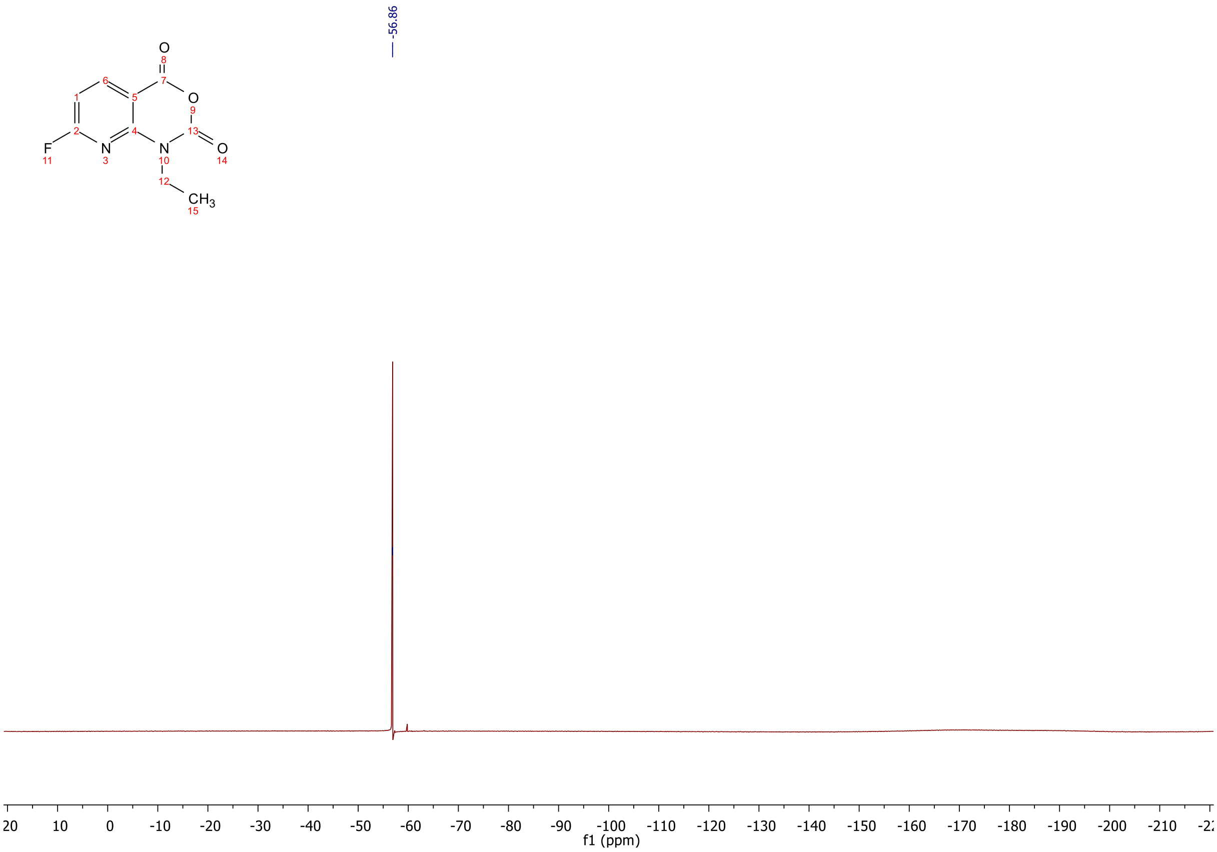
-56.86
O
O
N
F
N
O
CH3
f1 (ppm)

2.15 Compound **3g**

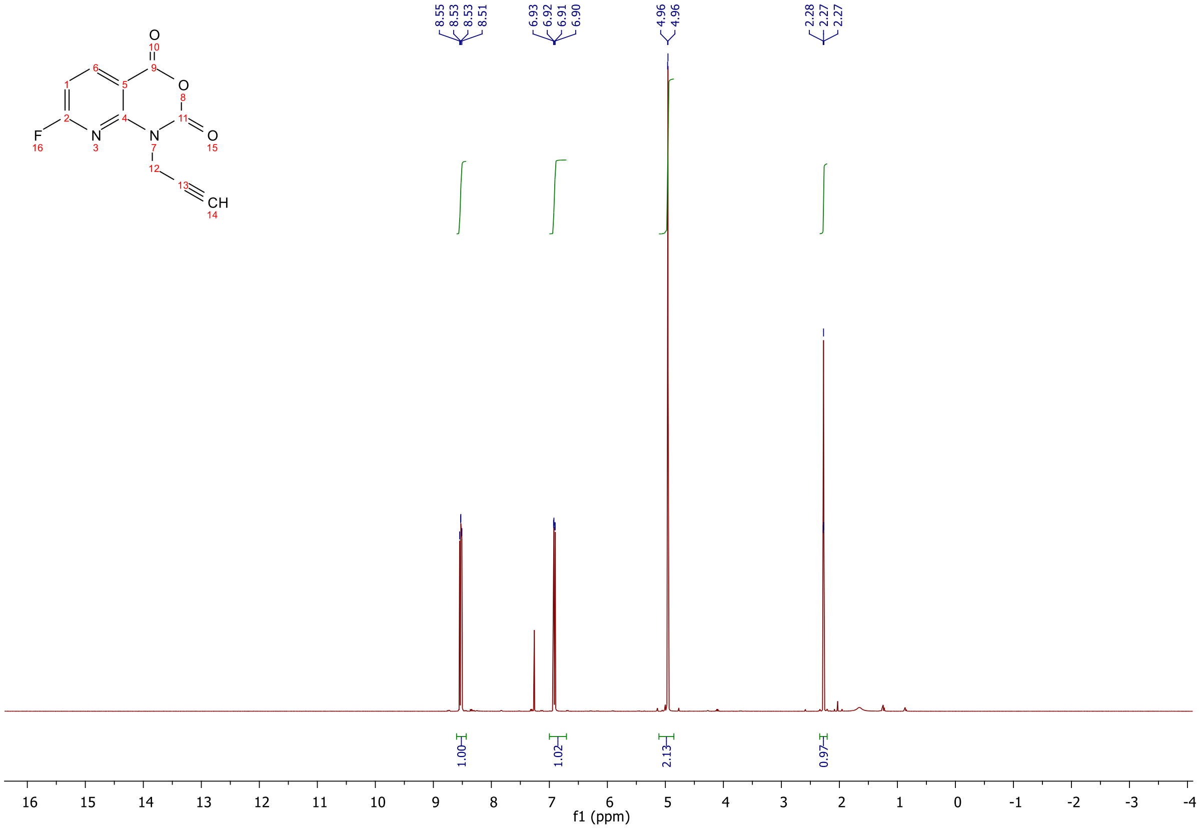

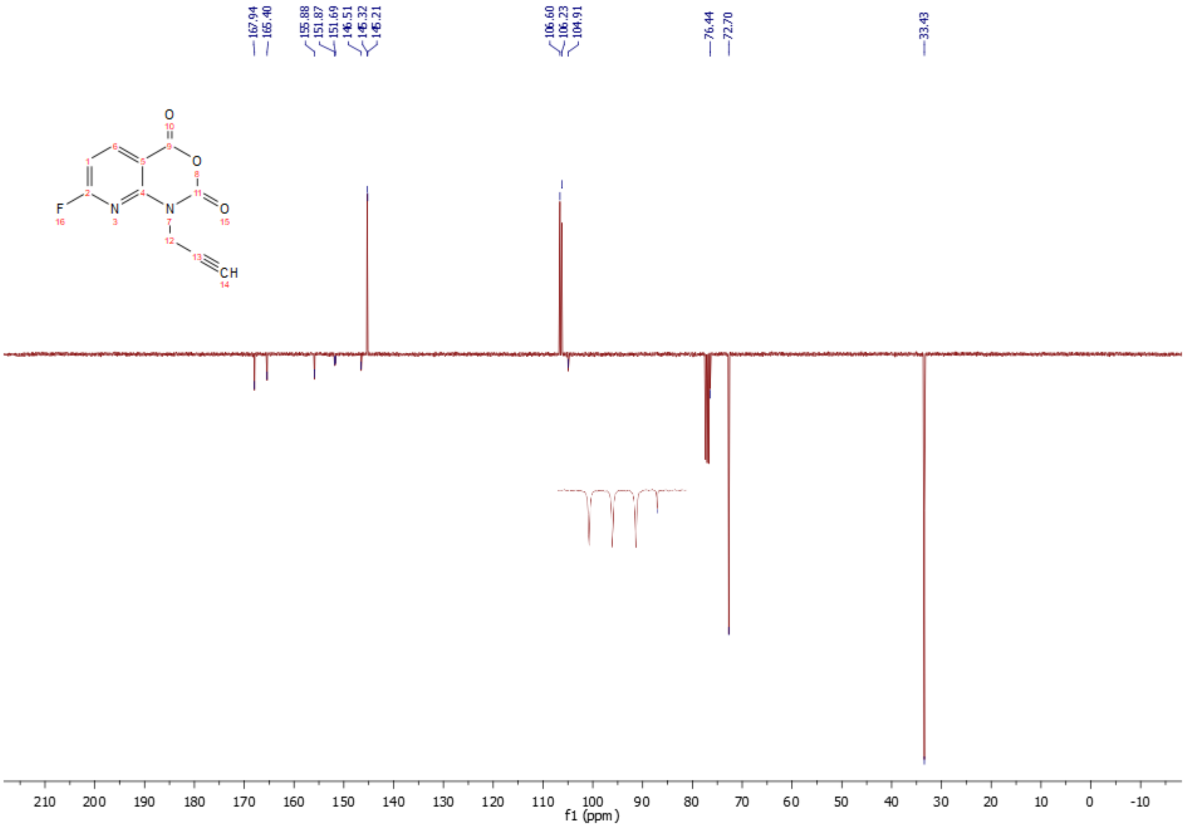

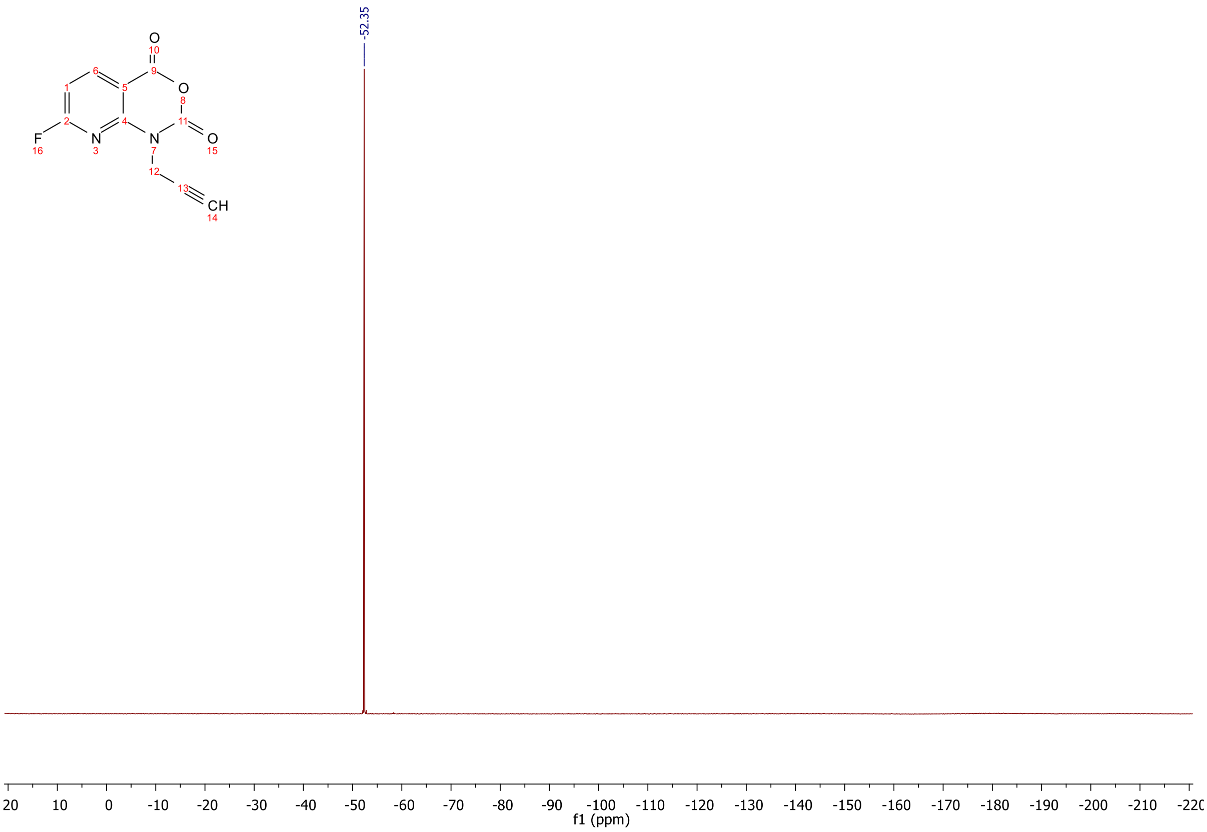
-52.35
O
O
O
F
N
N
CH
20 10 0 -10 -20 -30 -40 -50 -60 -70 -80 -90 -100 -110 -120 -130 -140 -150 -160 -170 -180 -190 -200 -210 -220
f1 (ppm)

2.16 Compound **3h**

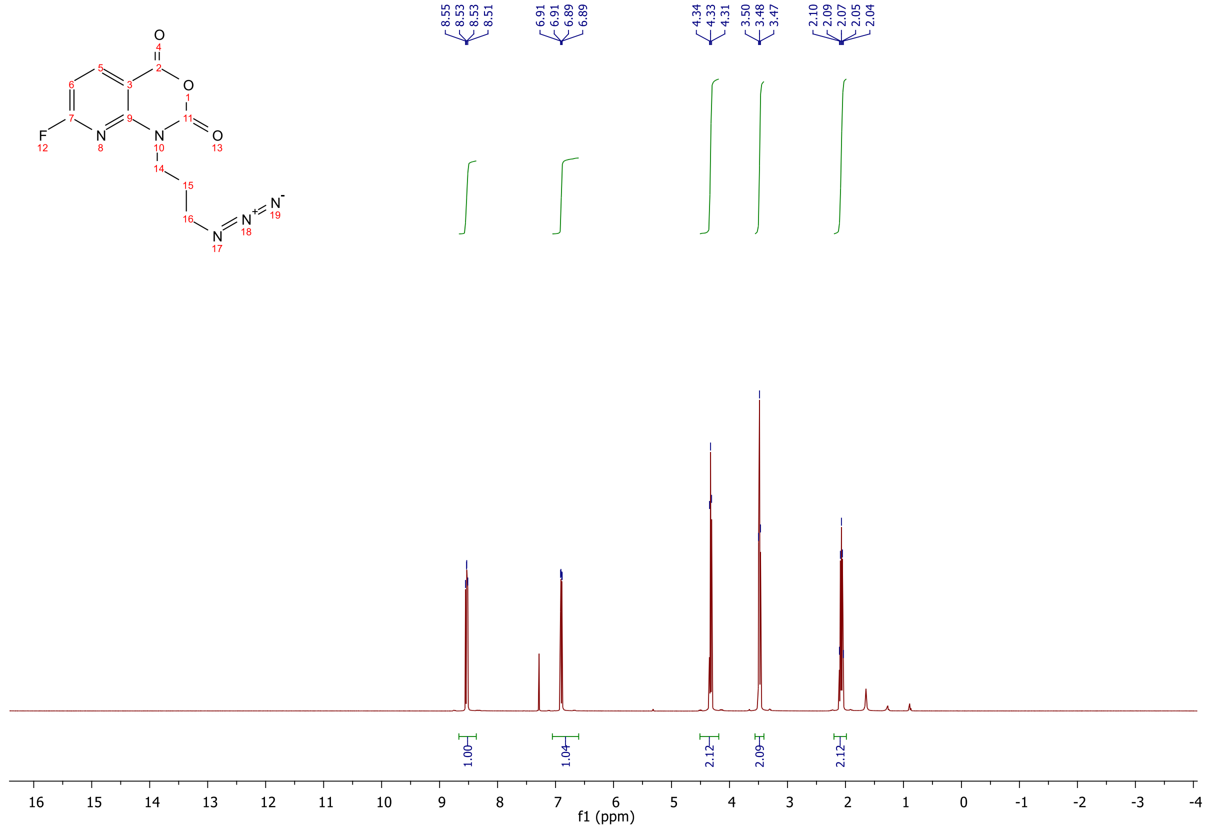

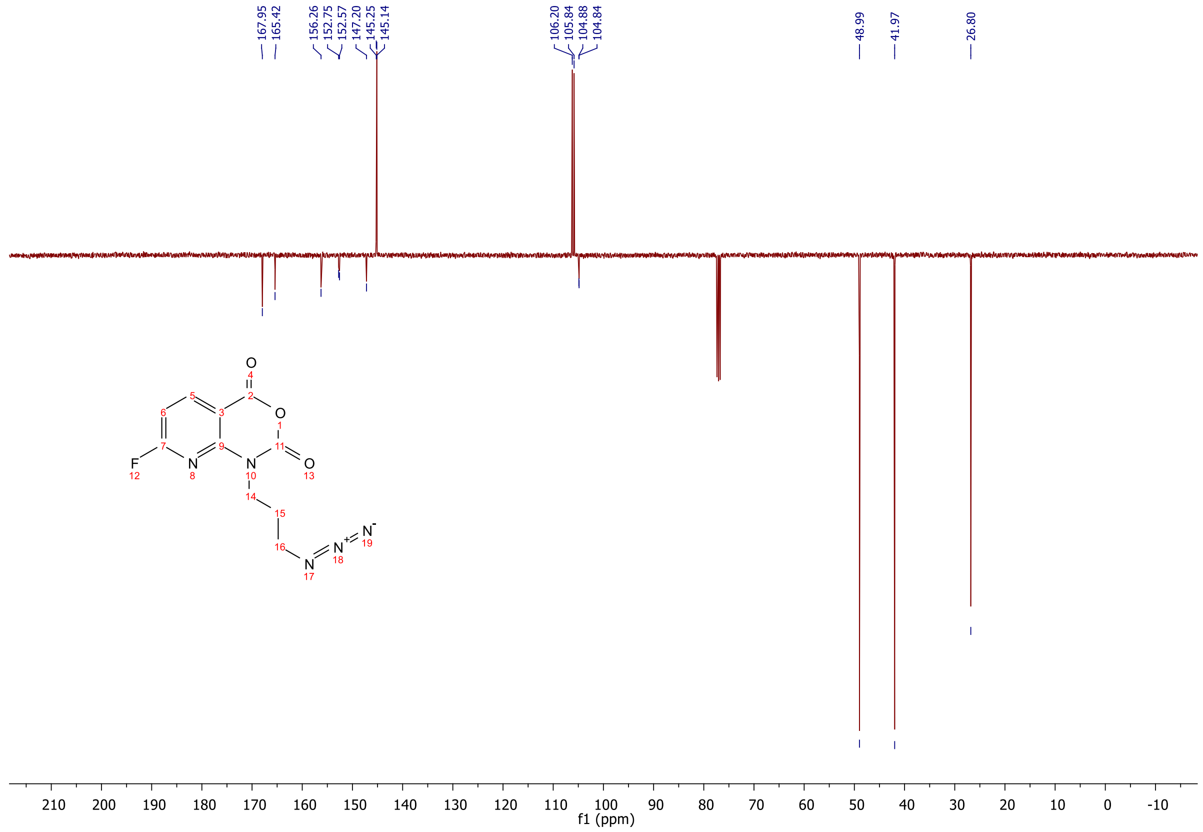

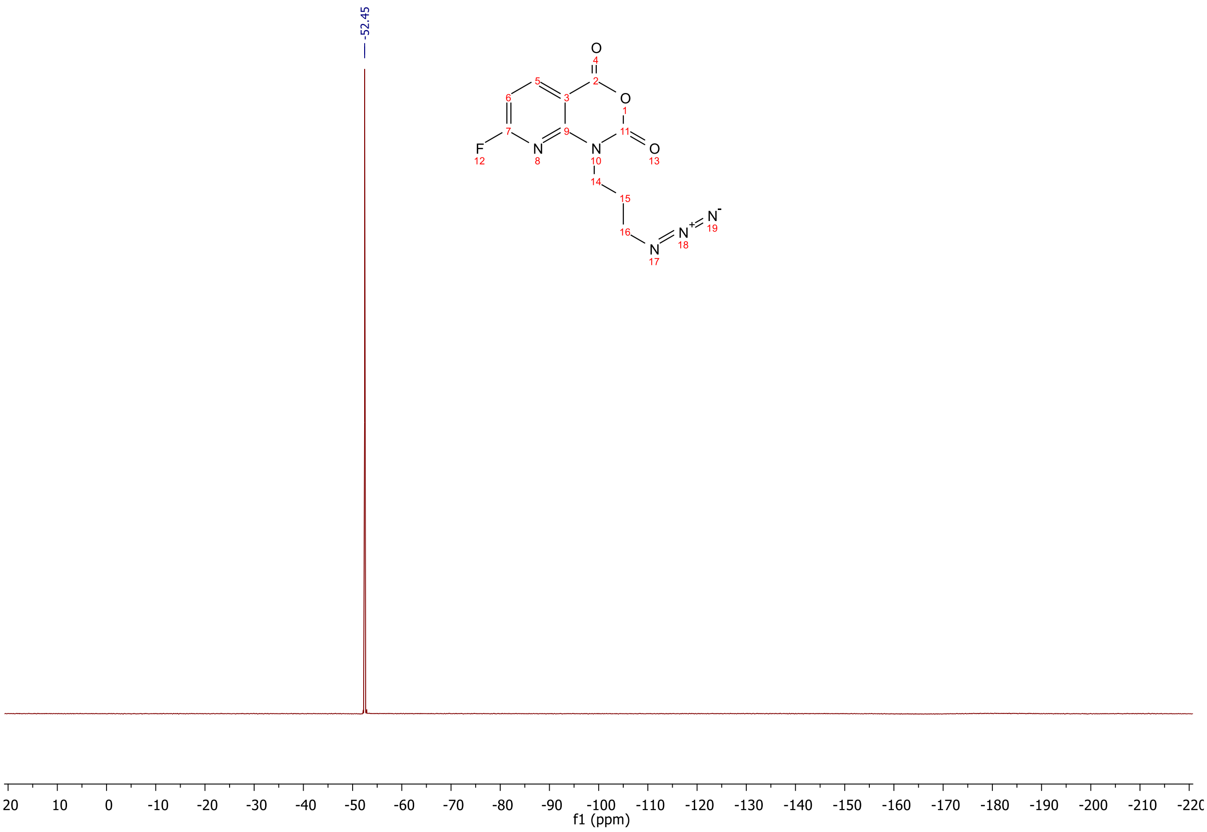

-52.45
O
4
2
O
1
11
O
13
5
6
3
7
9
F
12
N
8
N
10
14
15
16
N
17
N+
18
N-
19
20
10
0
-10
-20
-30
-40
-50
-60
-70
-80
-90
-100
-110
-120
-130
-140
-150
-160
-170
-180
-190
-200
-210
-220
f1 (ppm)

2.17 Compound **4a**

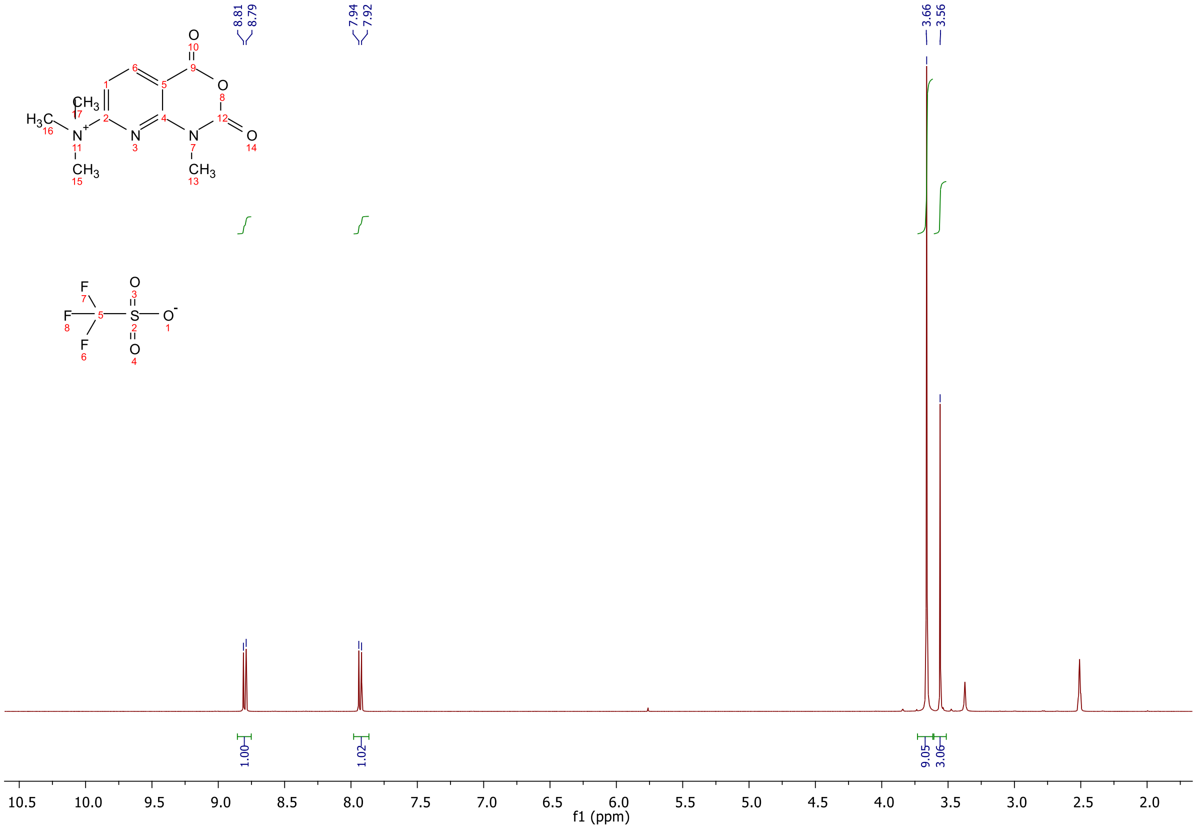

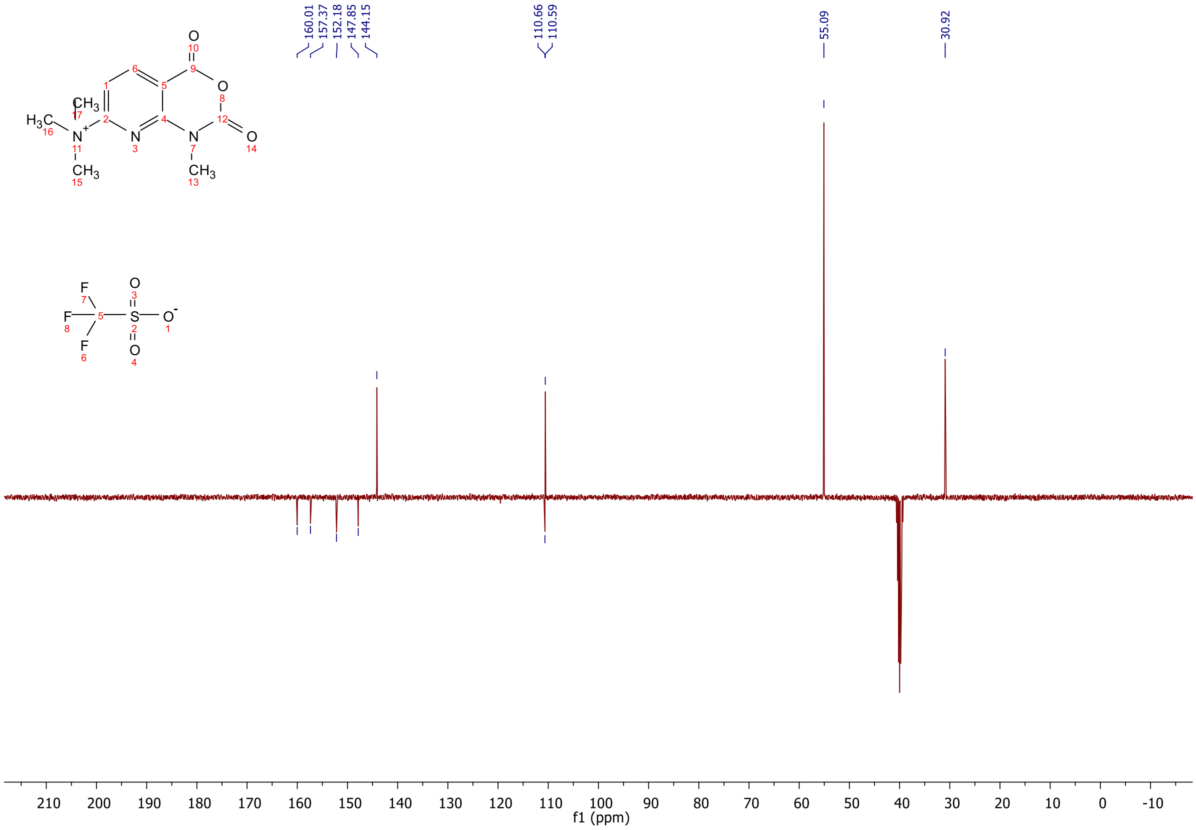

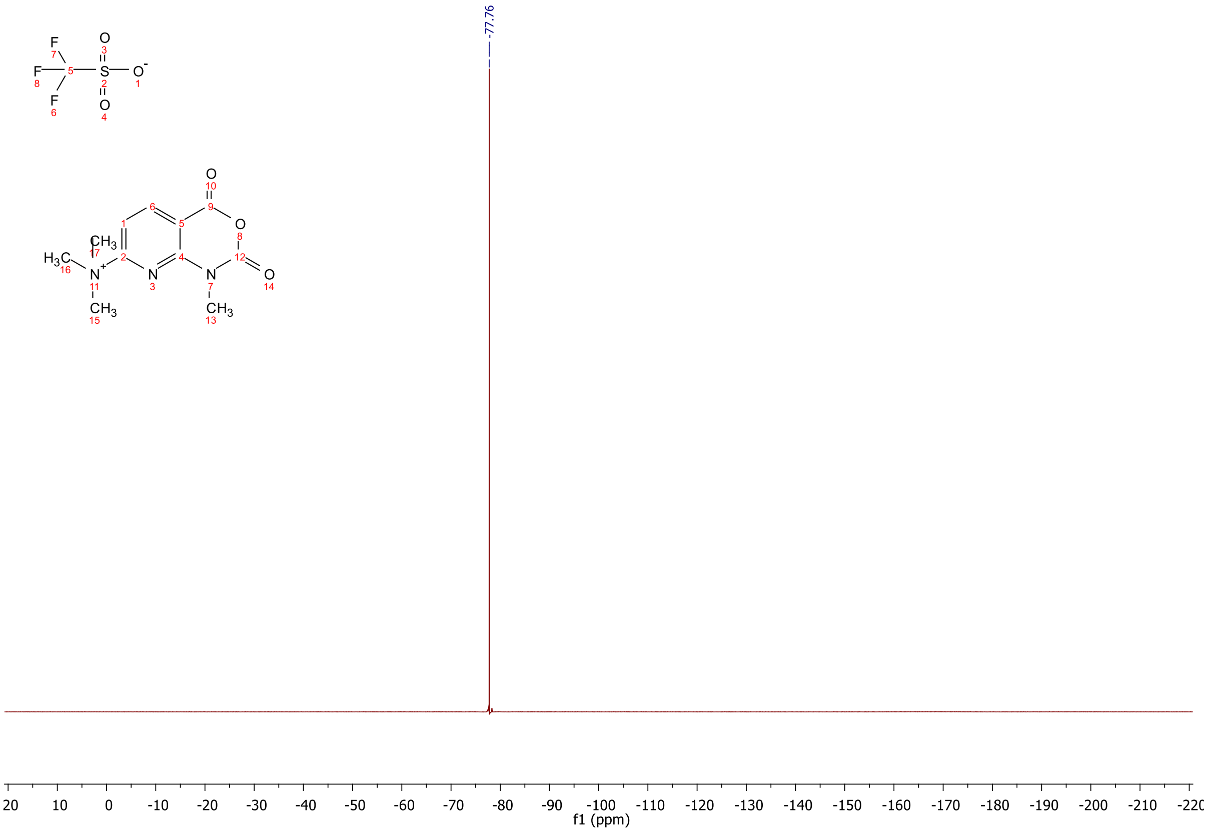
-77.76
20 10 0 -10 -20 -30 -40 -50 -60 -70 -80 -90 -100 -110 -120 -130 -140 -150 -160 -170 -180 -190 -200 -210 -220
f1 (ppm)

2.18 Compound **4b**

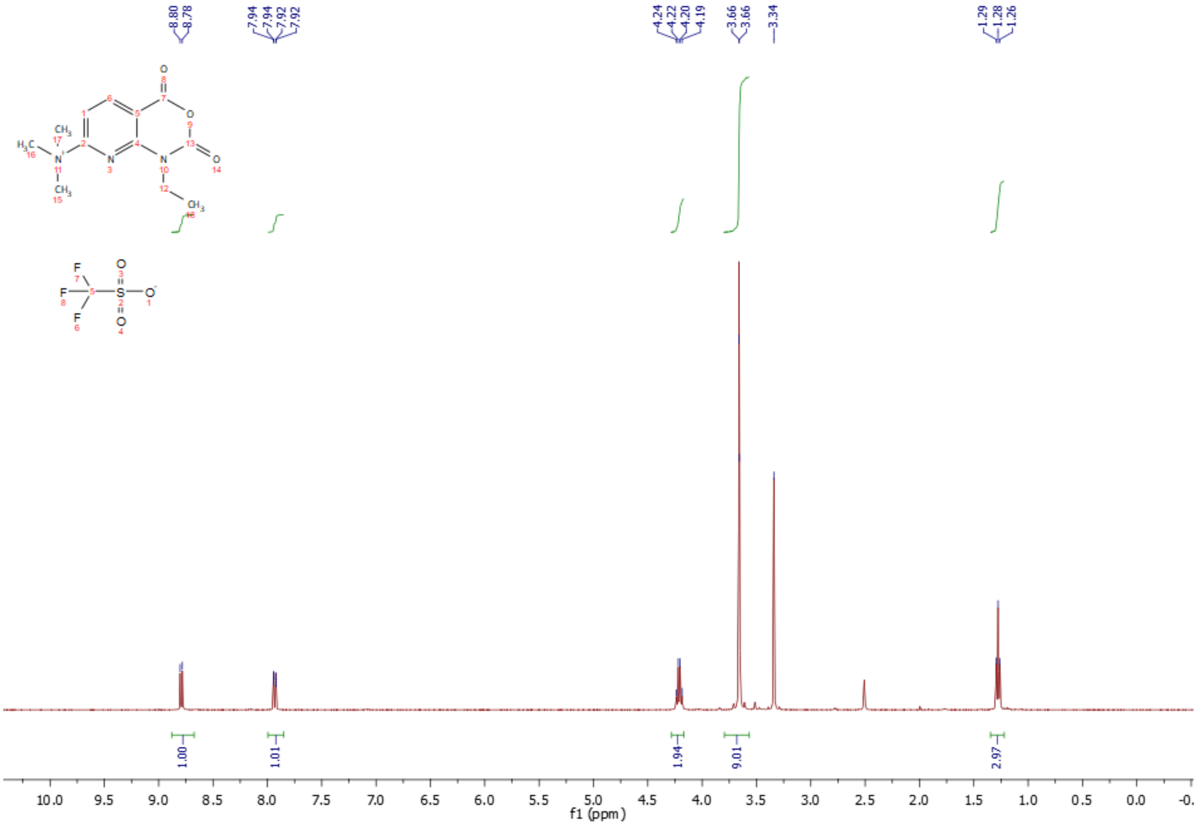

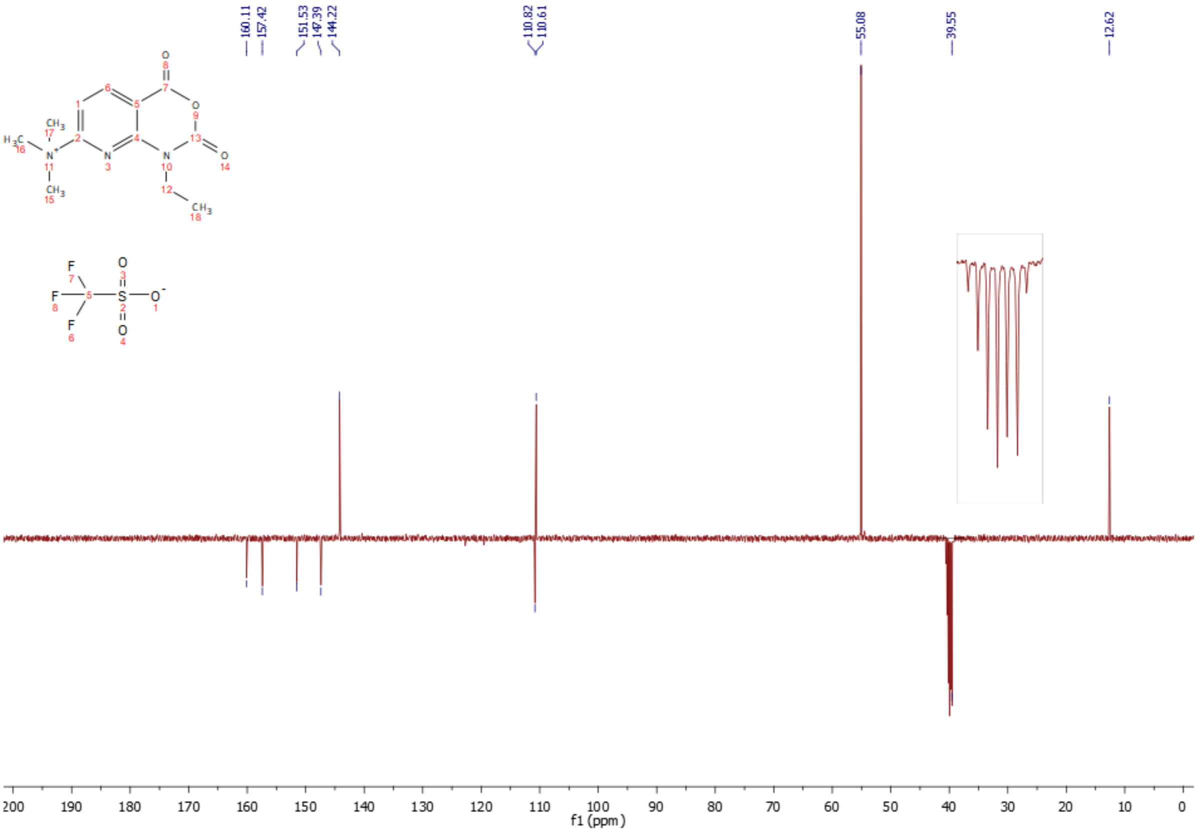

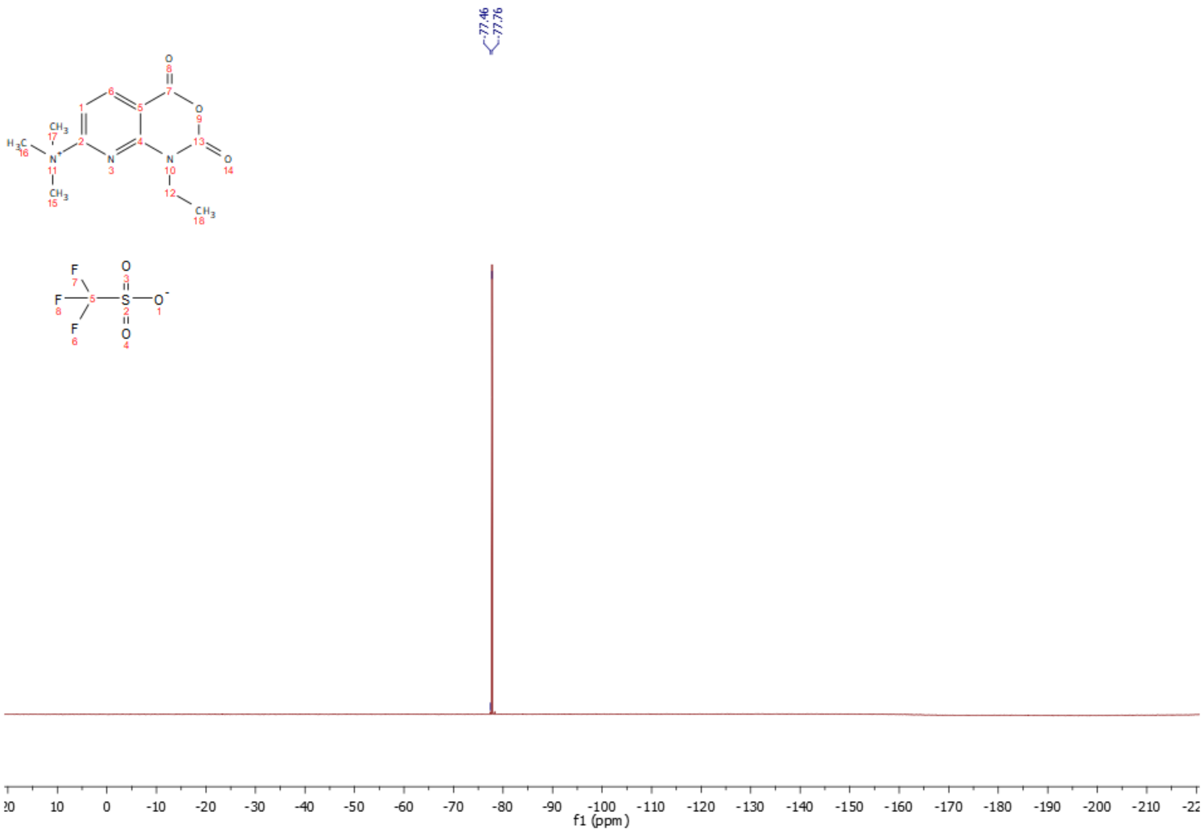

-77.46
-77.76
f1 (ppm)

2.19 Compound **4c**

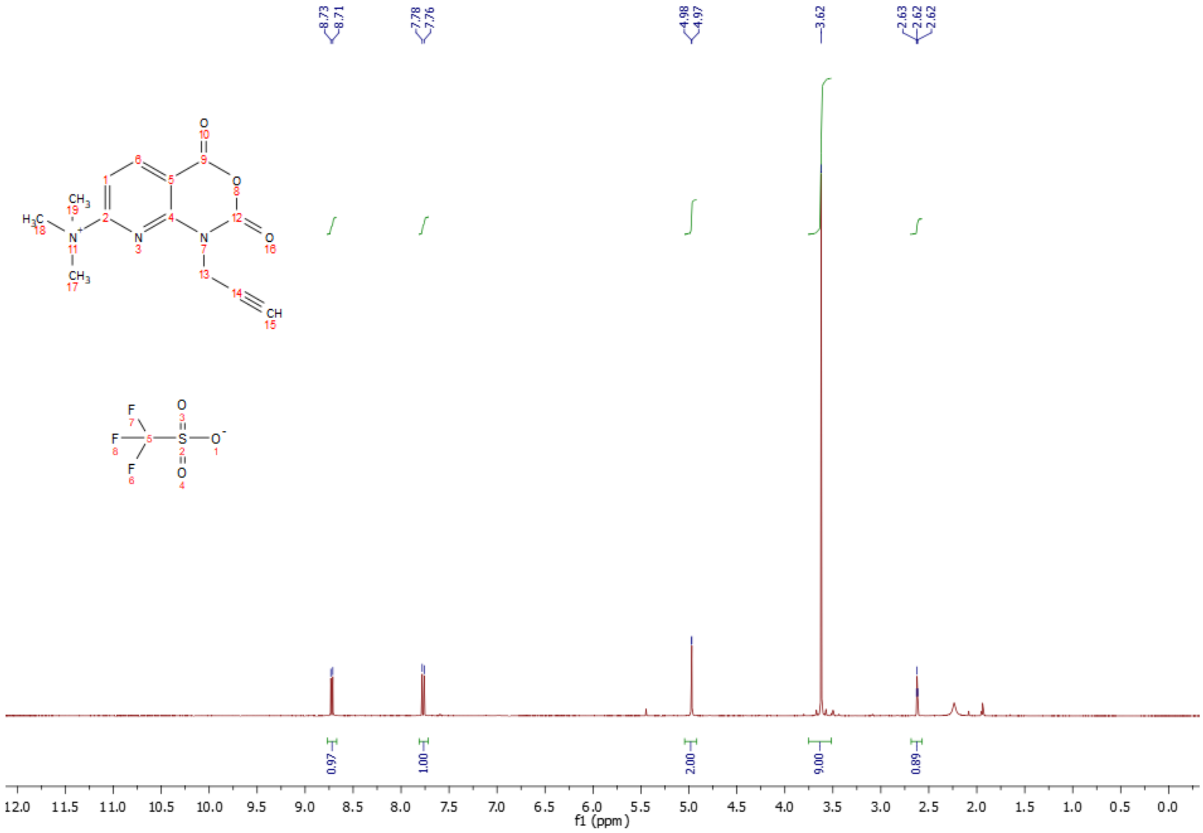

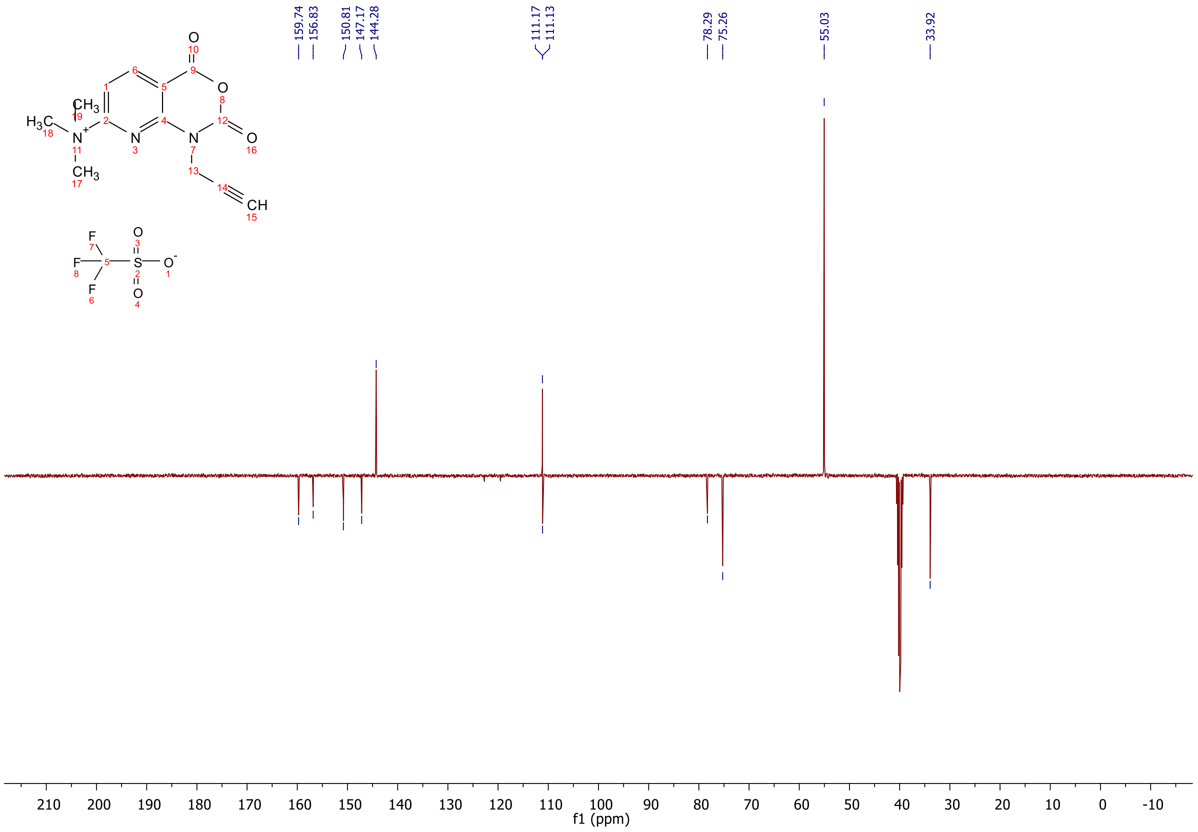

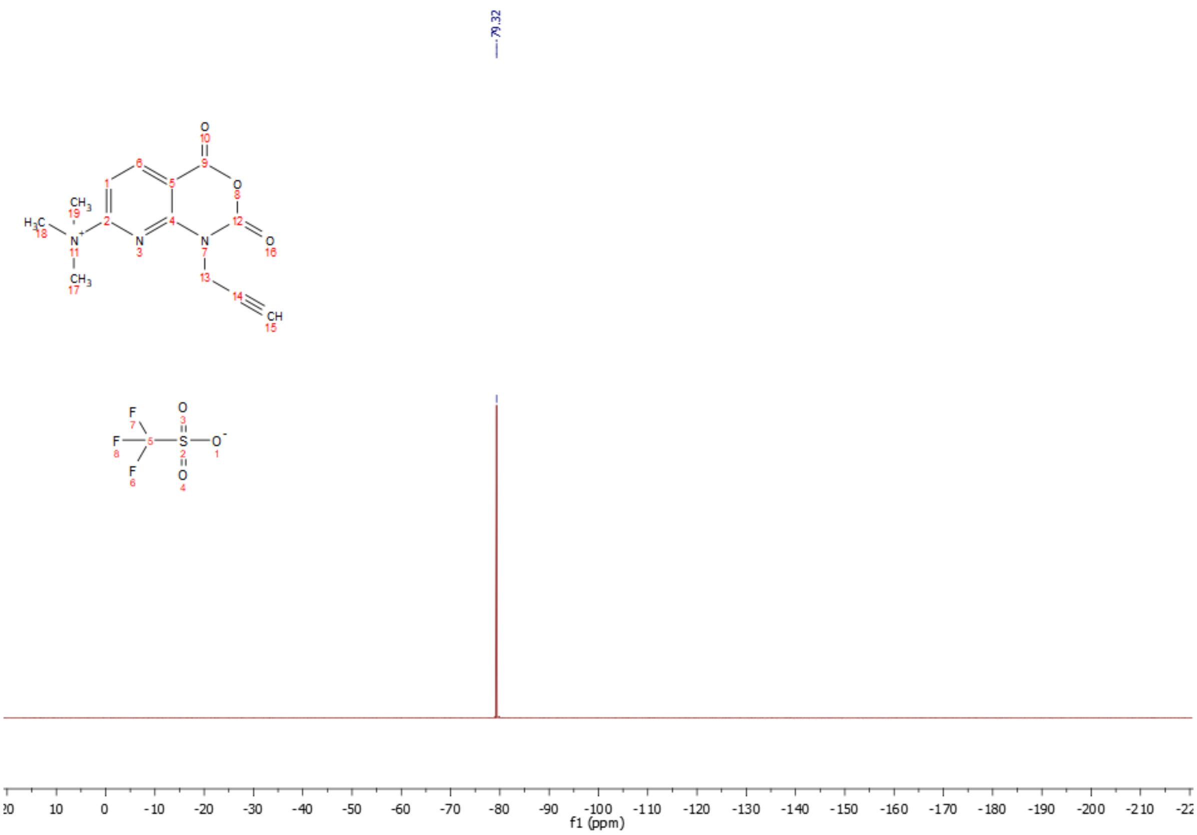
-79.32
20 10 0 -10 -20 -30 -40 -50 -60 -70 -80 -90 -100 -110 -120 -130 -140 -150 -160 -170 -180 -190 -200 -210 -22
f1 (ppm)

2.20 Compound **4d**

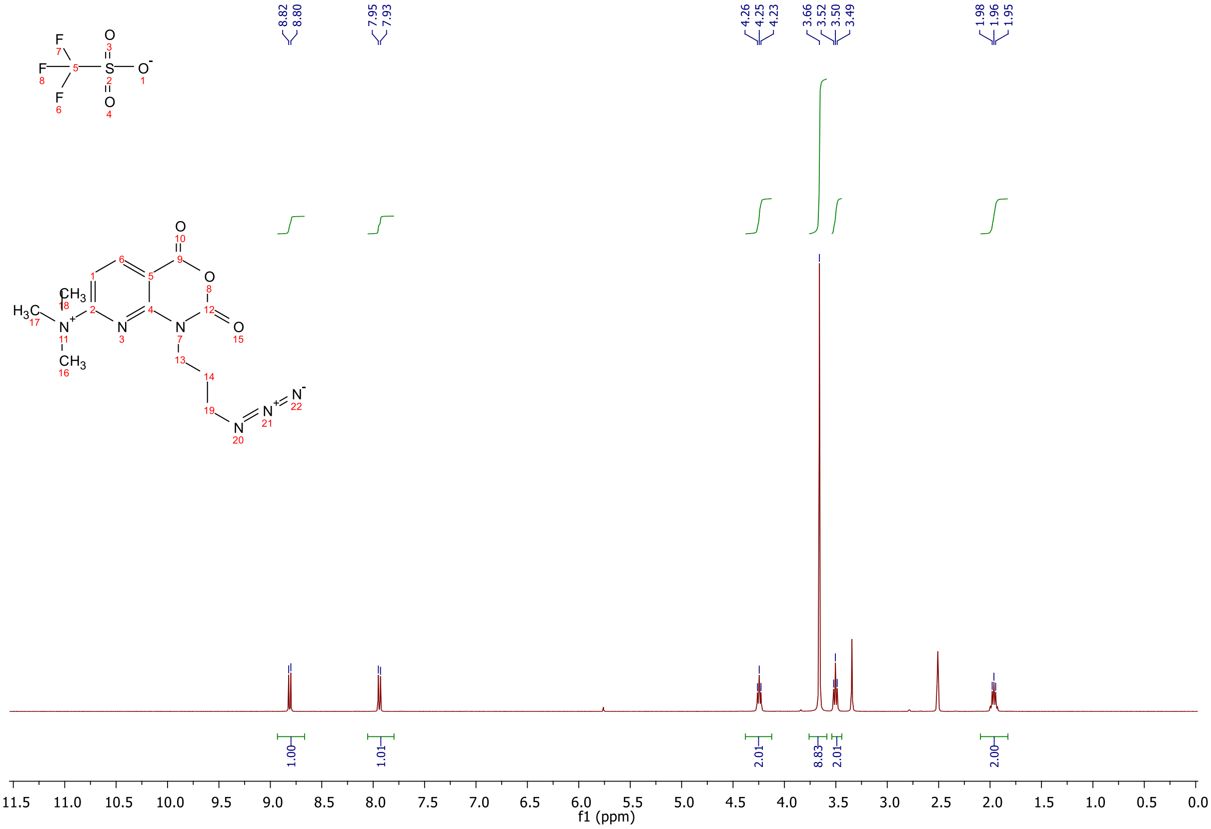

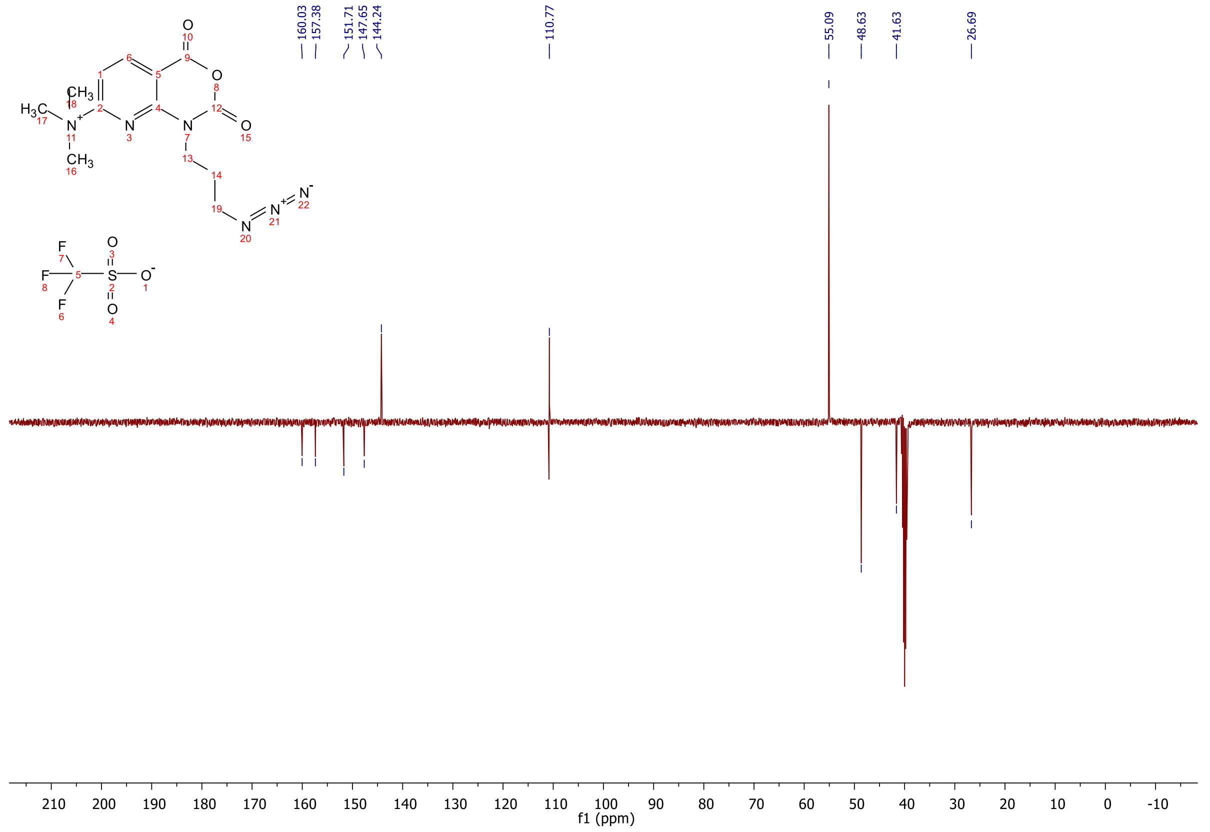

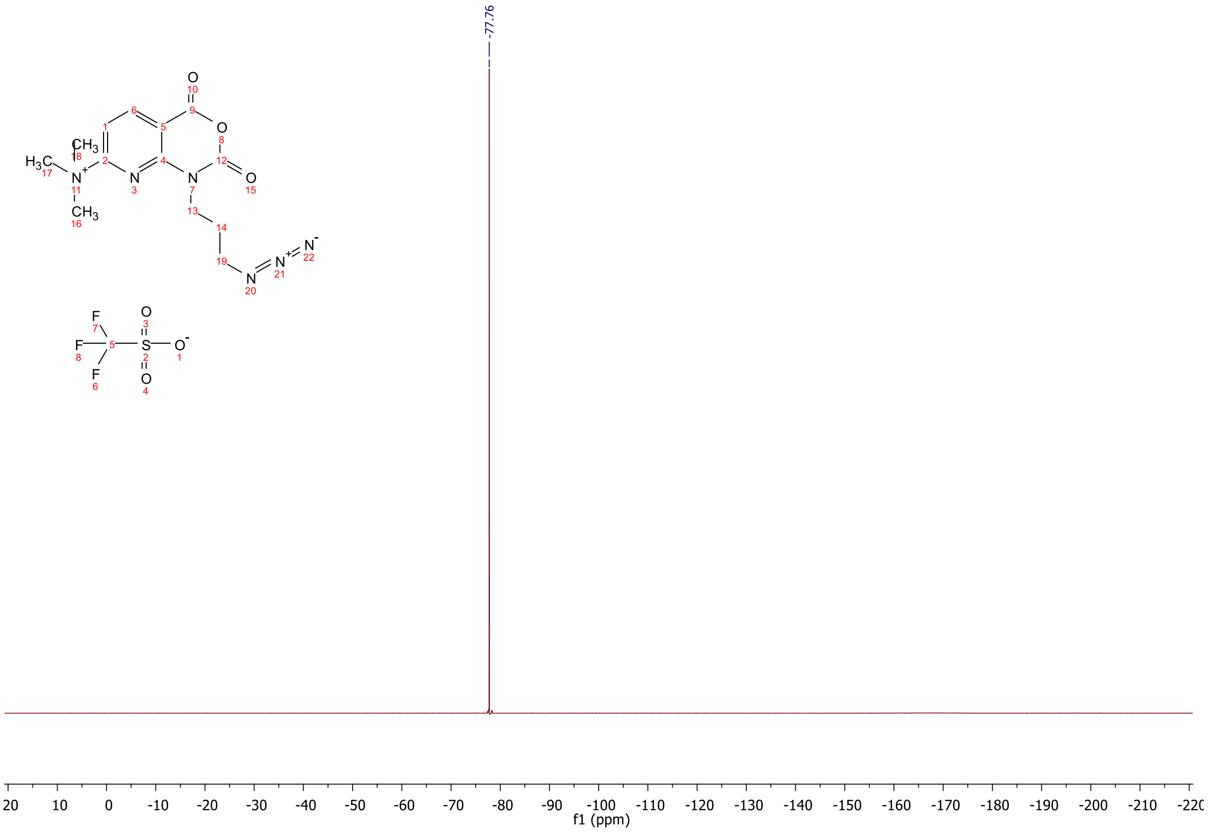
-77.76
f1 (ppm)

2.21 Compound **5a**

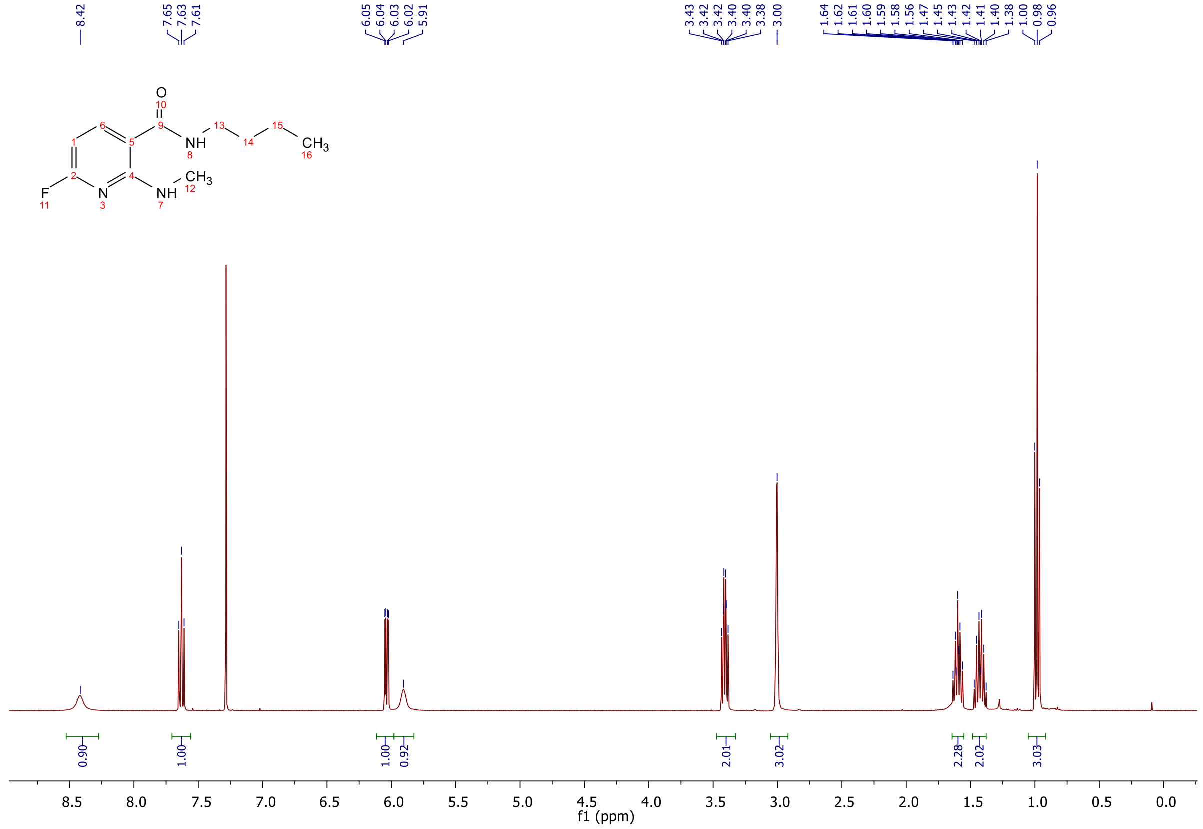

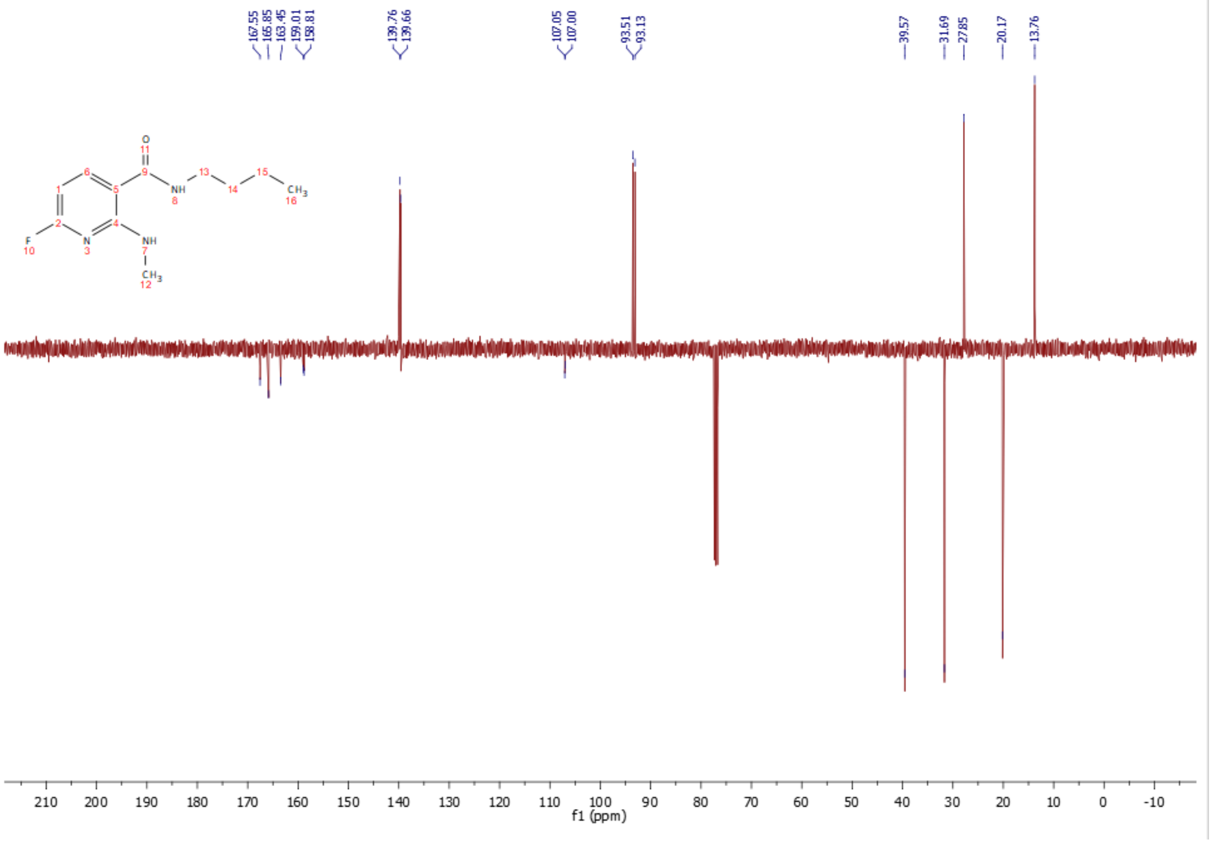

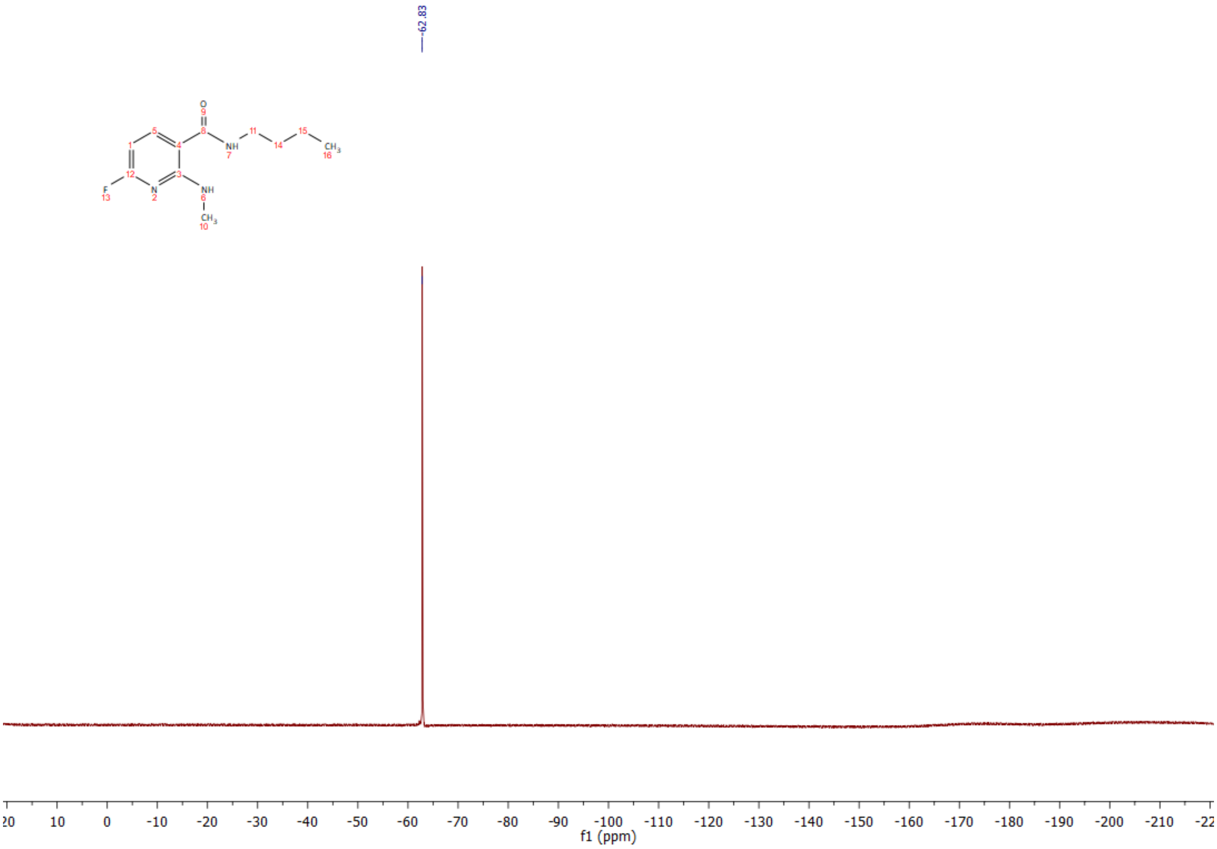
-62.83
O
9
8
5
1
4
NH
7
11
14
15
CH3
16
F
13
12
N
2
3
NH
6
CH3
10
20
10
0
-10
-20
-30
-40
-50
-60
-70
-80
-90
-100
-110
-120
-130
-140
-150
-160
-170
-180
-190
-200
-210
-22
f1 (ppm)

2.22 Compound **5b**

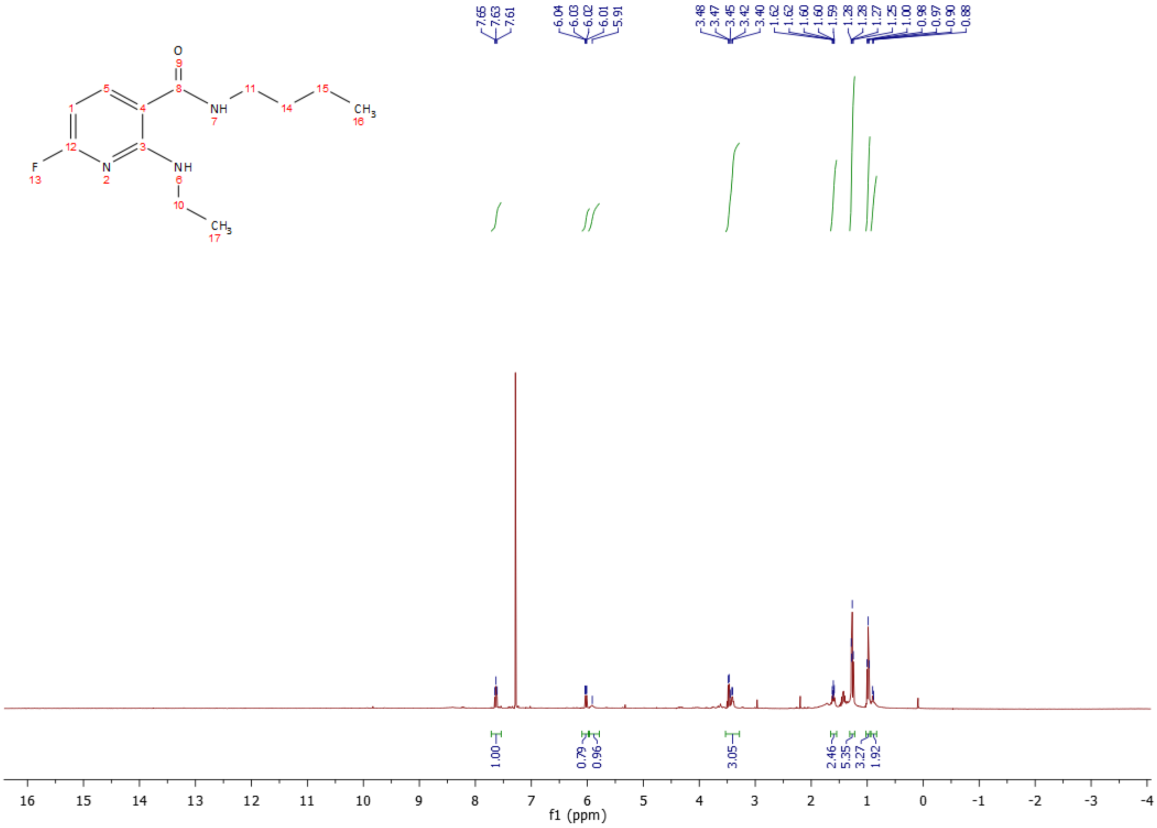

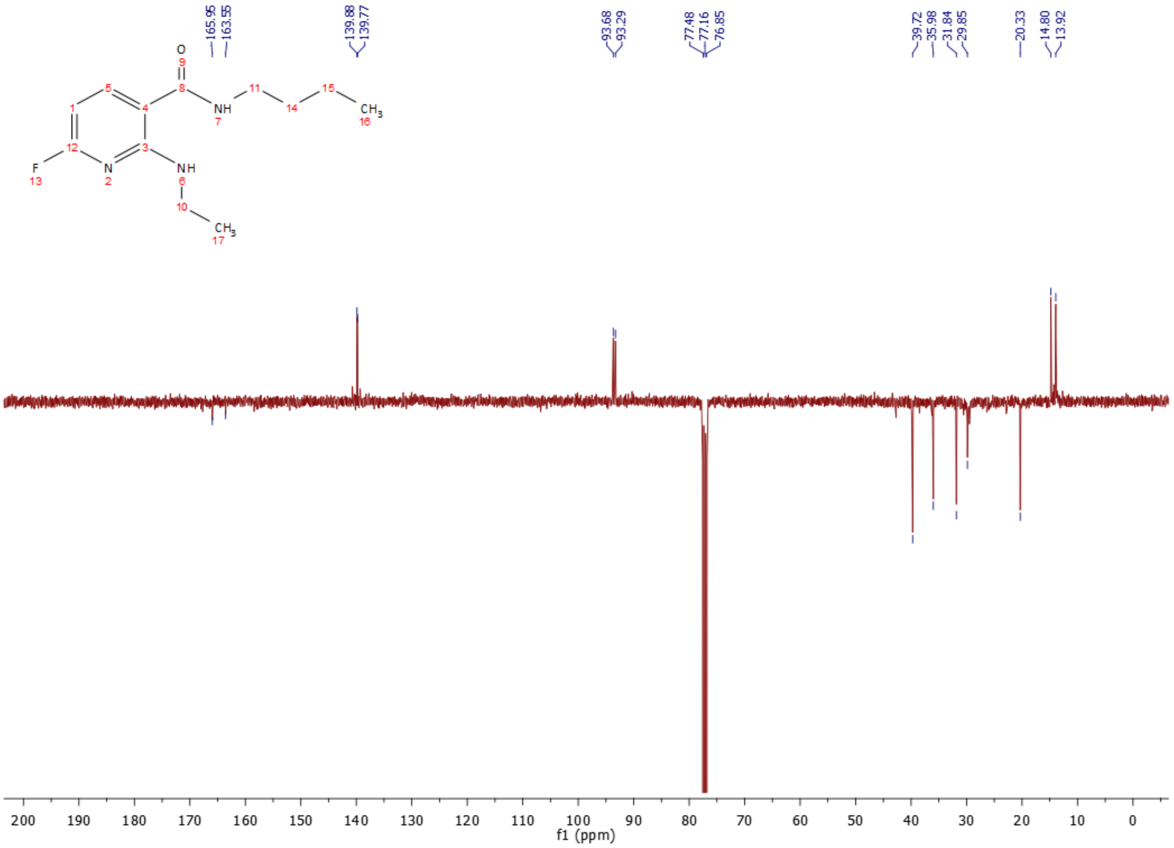

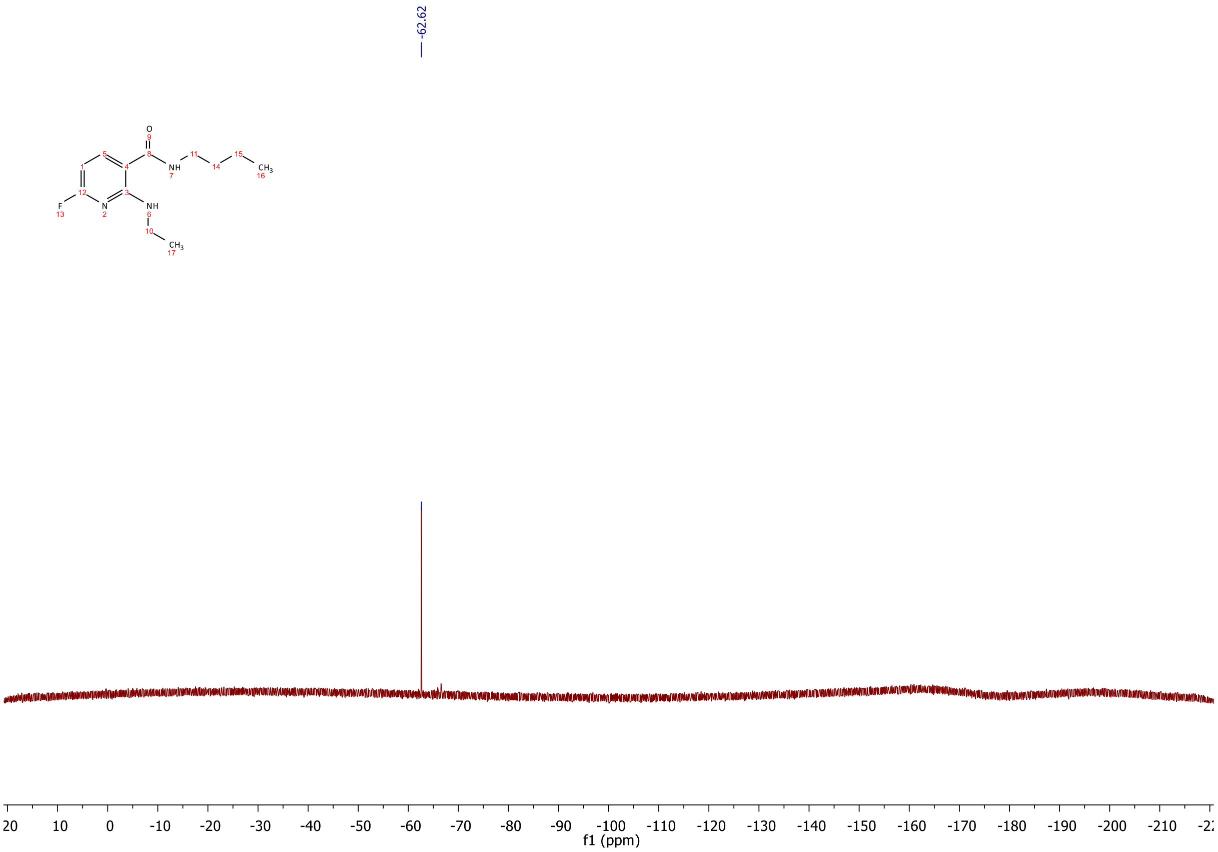
-62.62
O
9
8
NH
7
11
14
15
CH3
16
5
4
1
12
3
F
13
N
2
NH
6
10
CH3
17
20
10
0
-10
-20
-30
-40
-50
-60
-70
-80
-90
-100
-110
-120
-130
-140
-150
-160
-170
-180
-190
-200
-210
f1 (ppm)

2.23 Compound **5c**

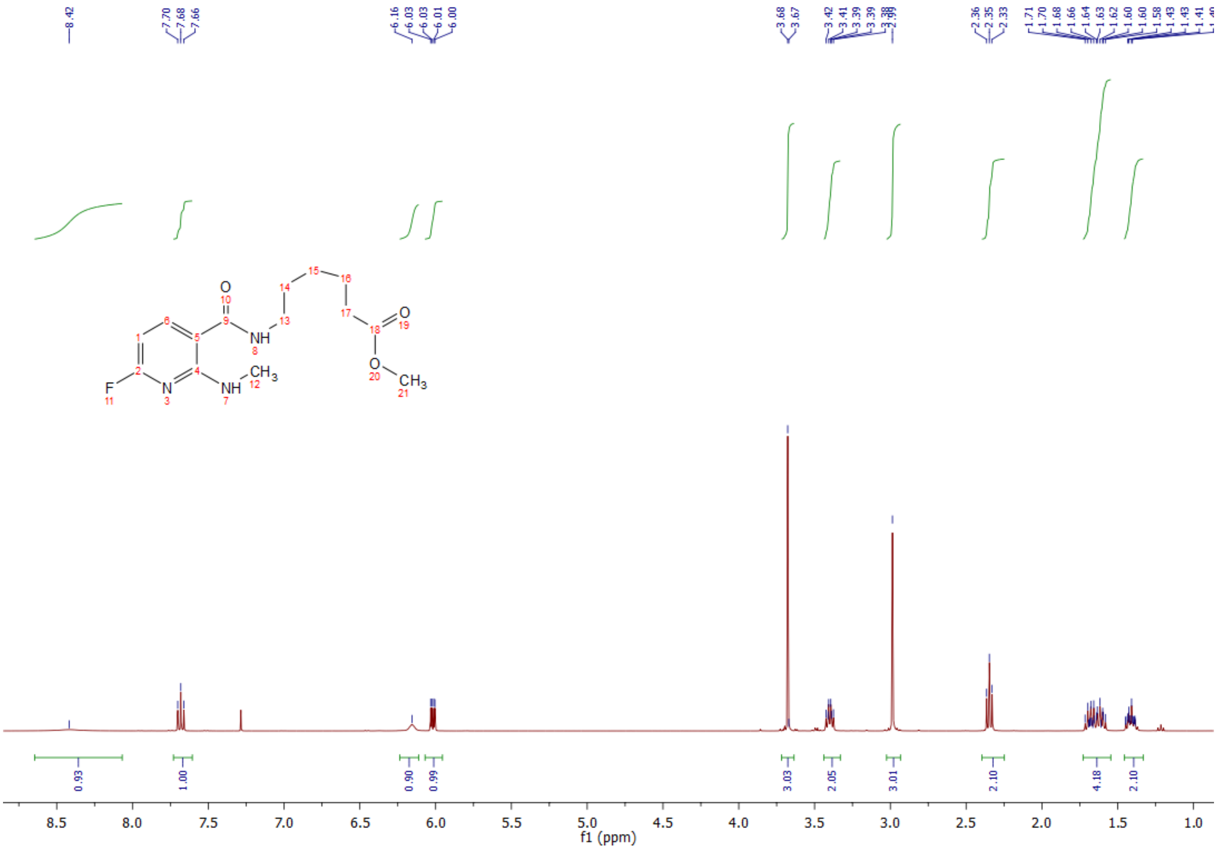

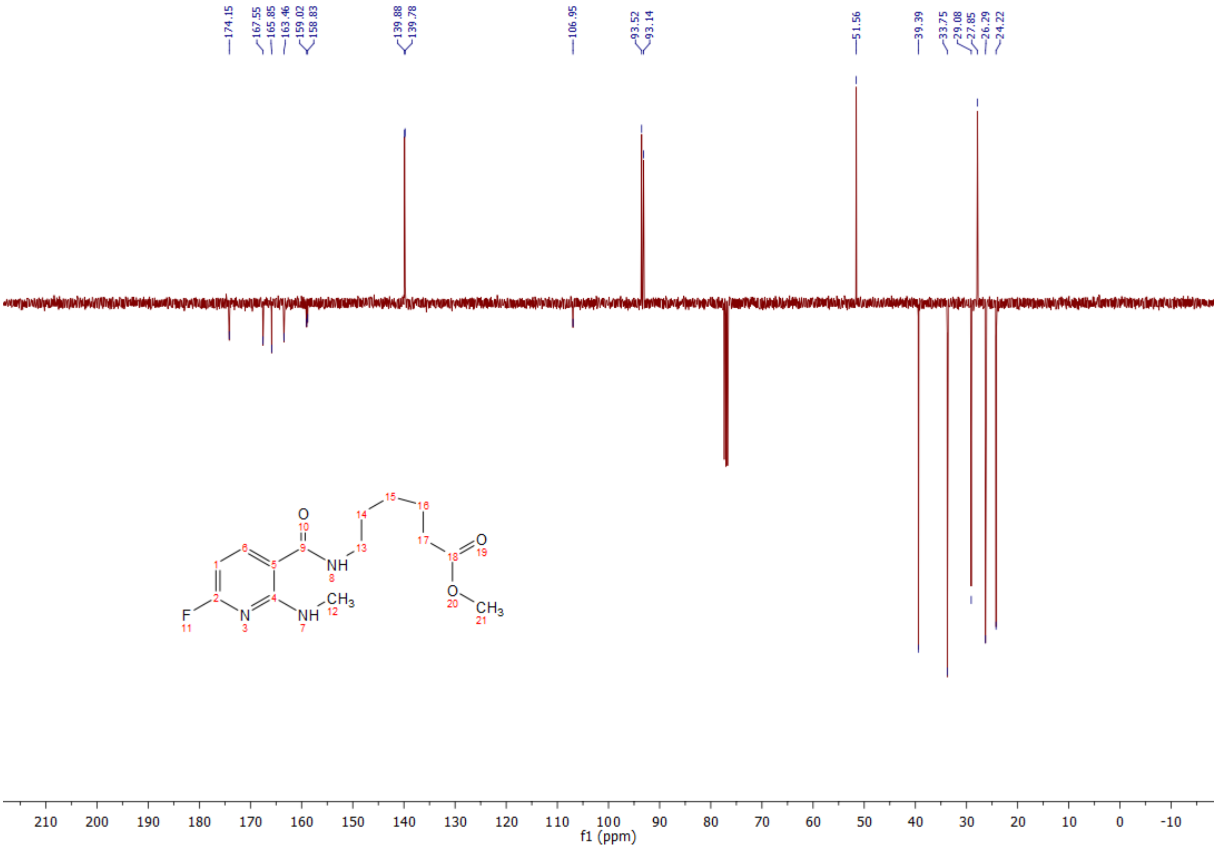

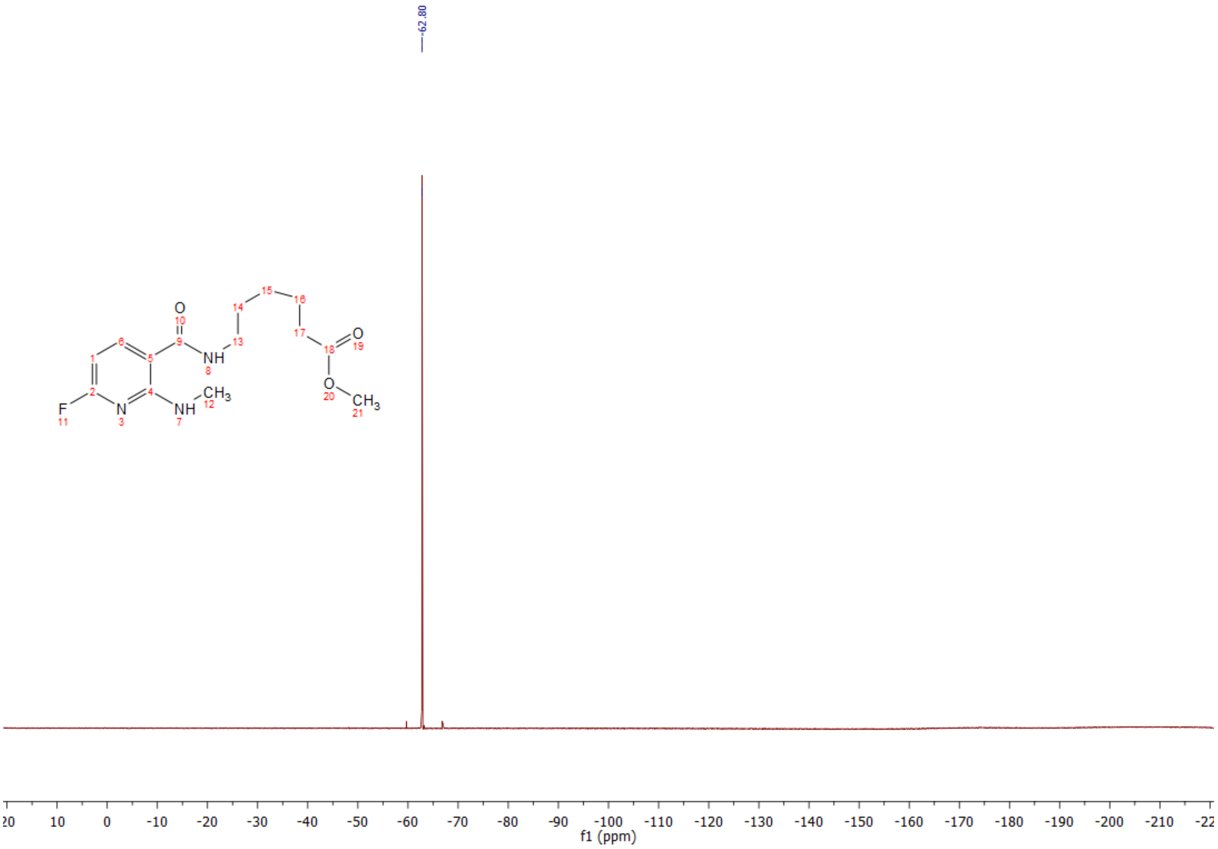
-62.80
F
N
NH
CH3
O
NH
O
O
CH3
f1 (ppm)

2.24 Compound **5d**

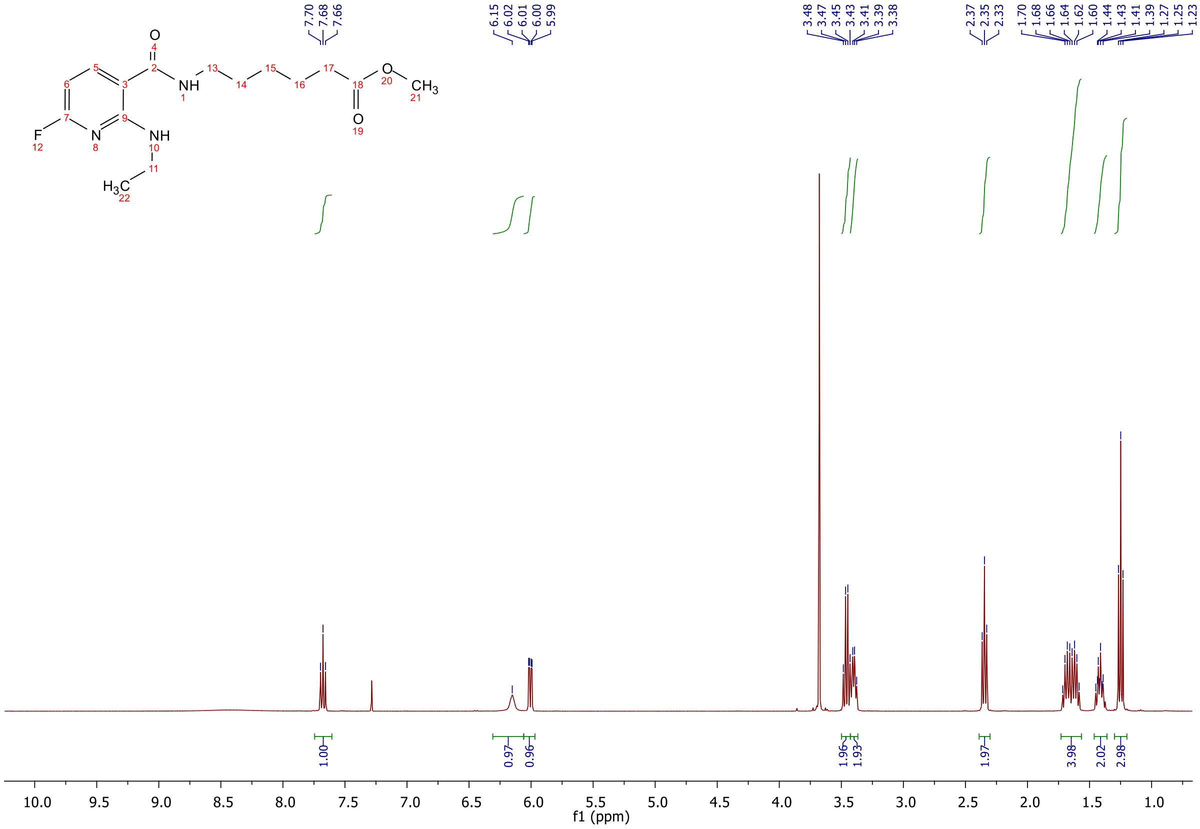

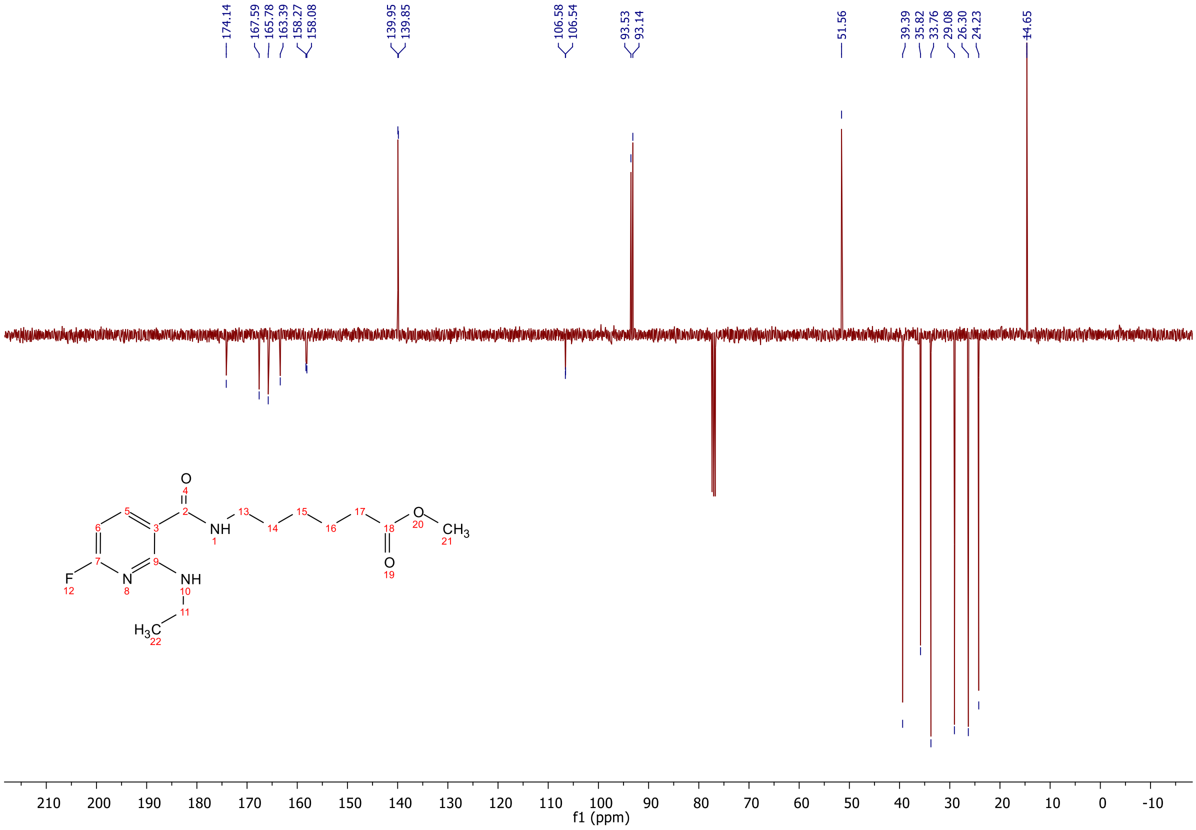

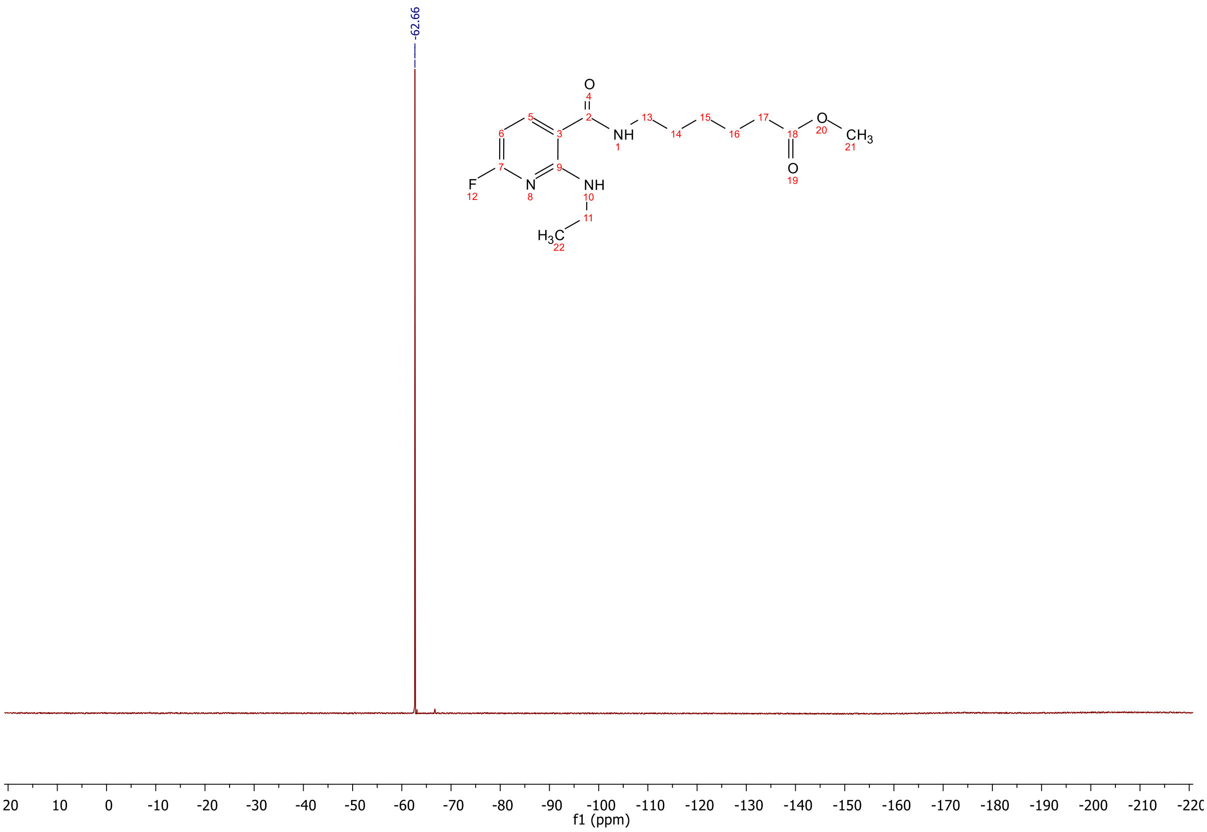

-62.66
20
10
0
-10
-20
-30
-40
-50
-60
-70
-80
-90
-100
-110
-120
-130
-140
-150
-160
-170
-180
-190
-200
-210
-220
f1 (ppm)

2.25 Compound **5e**

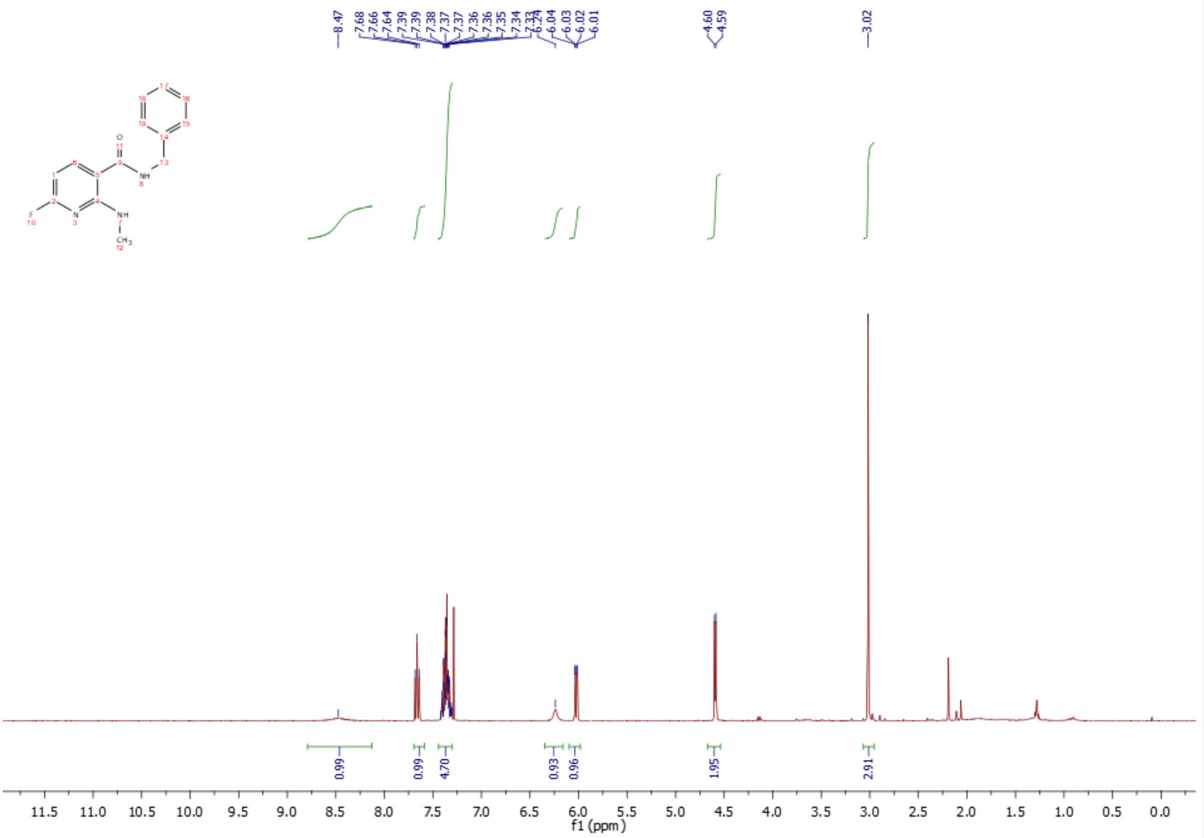

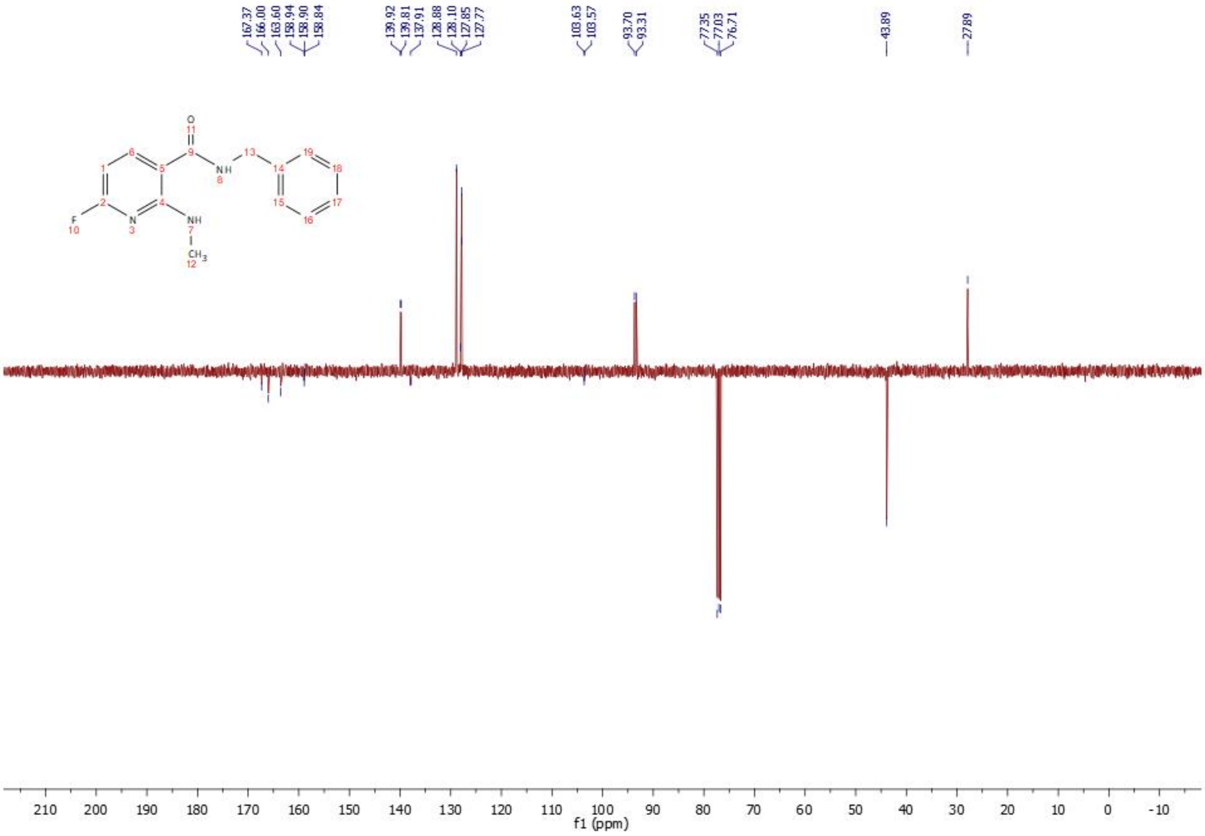

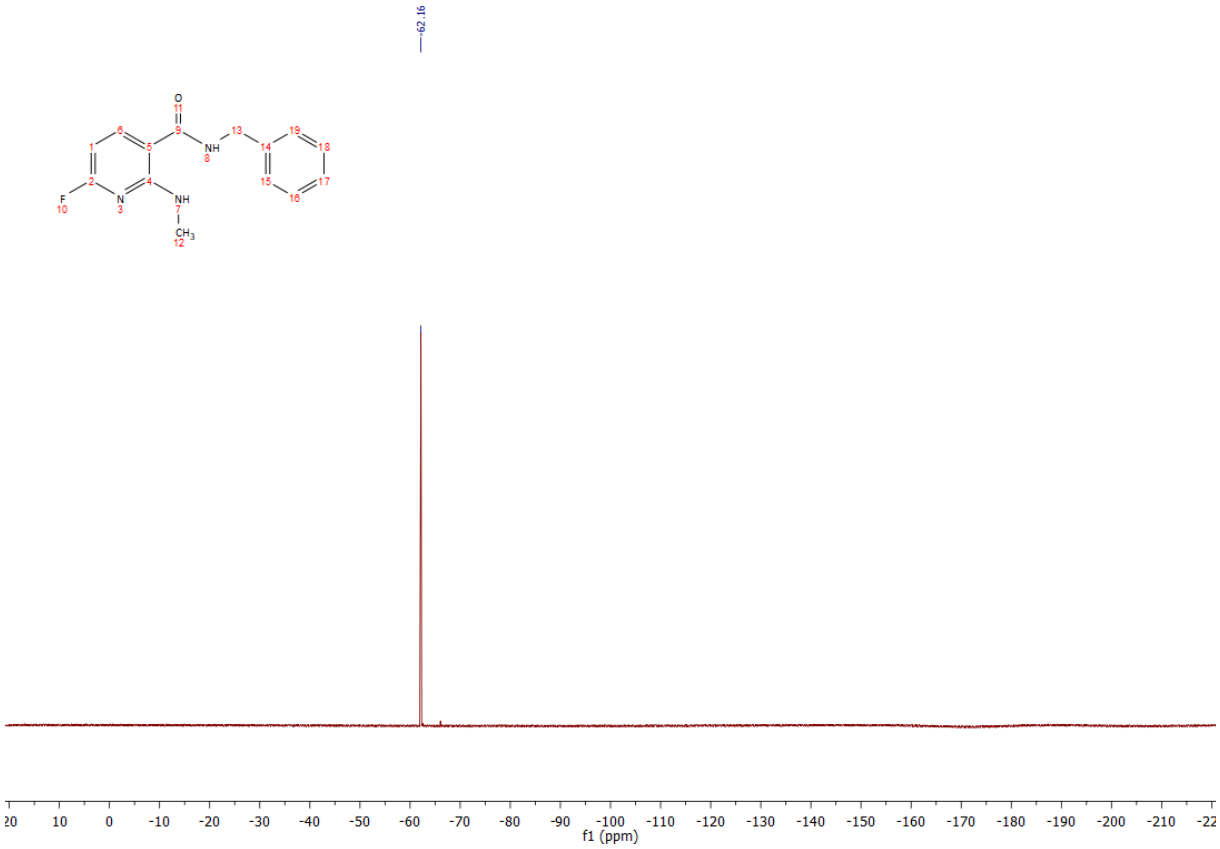
-62.16
O
NH
F
N
NH
CH3
f1 (ppm)
20 10 0 -10 -20 -30 -40 -50 -60 -70 -80 -90 -100 -110 -120 -130 -140 -150 -160 -170 -180 -190 -200 -210 -22

2.26 Compound **5f**

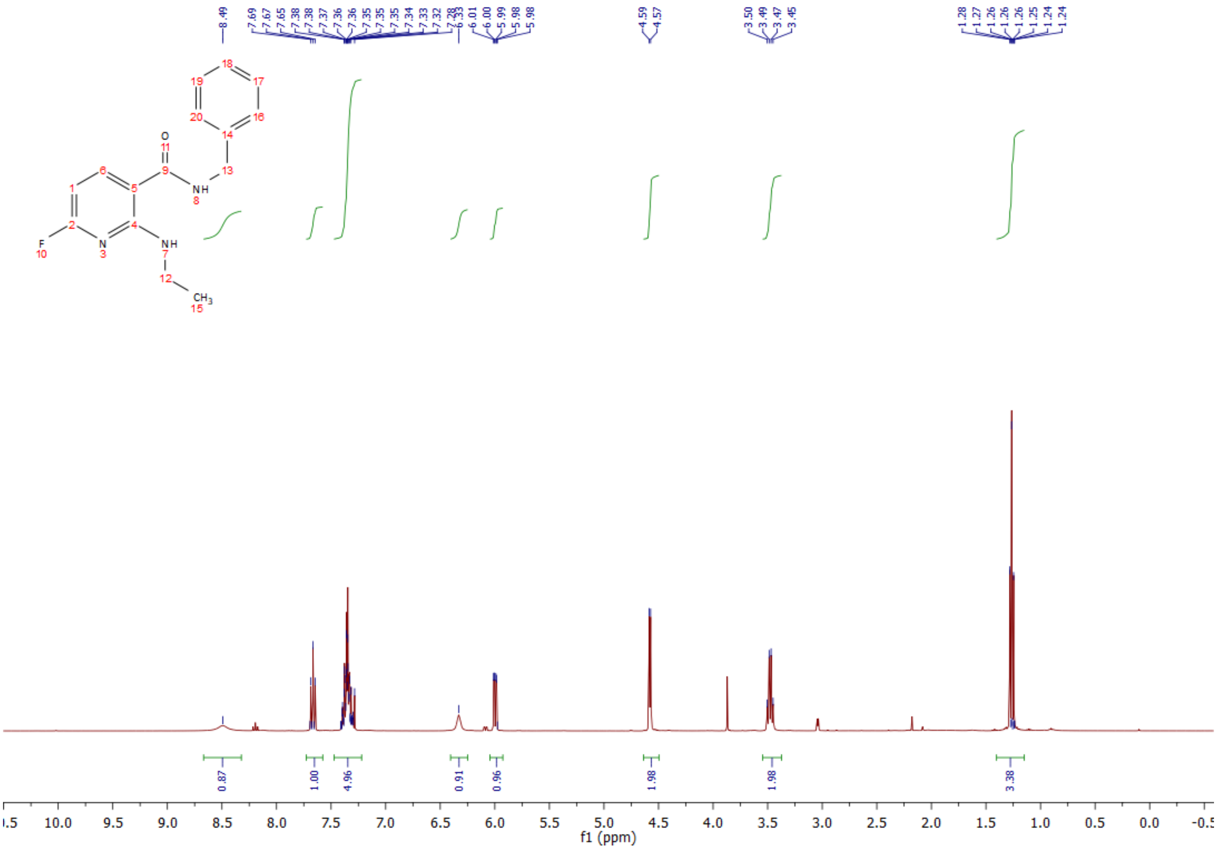

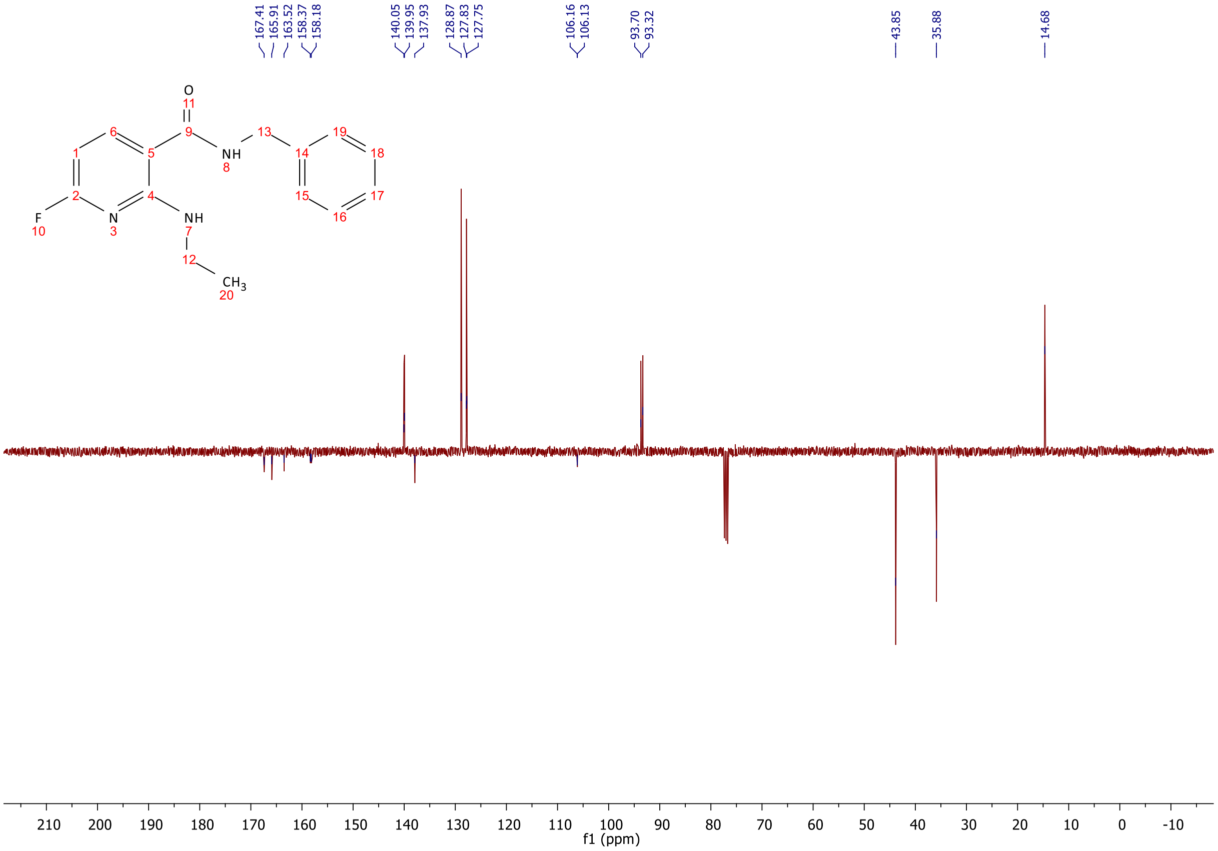

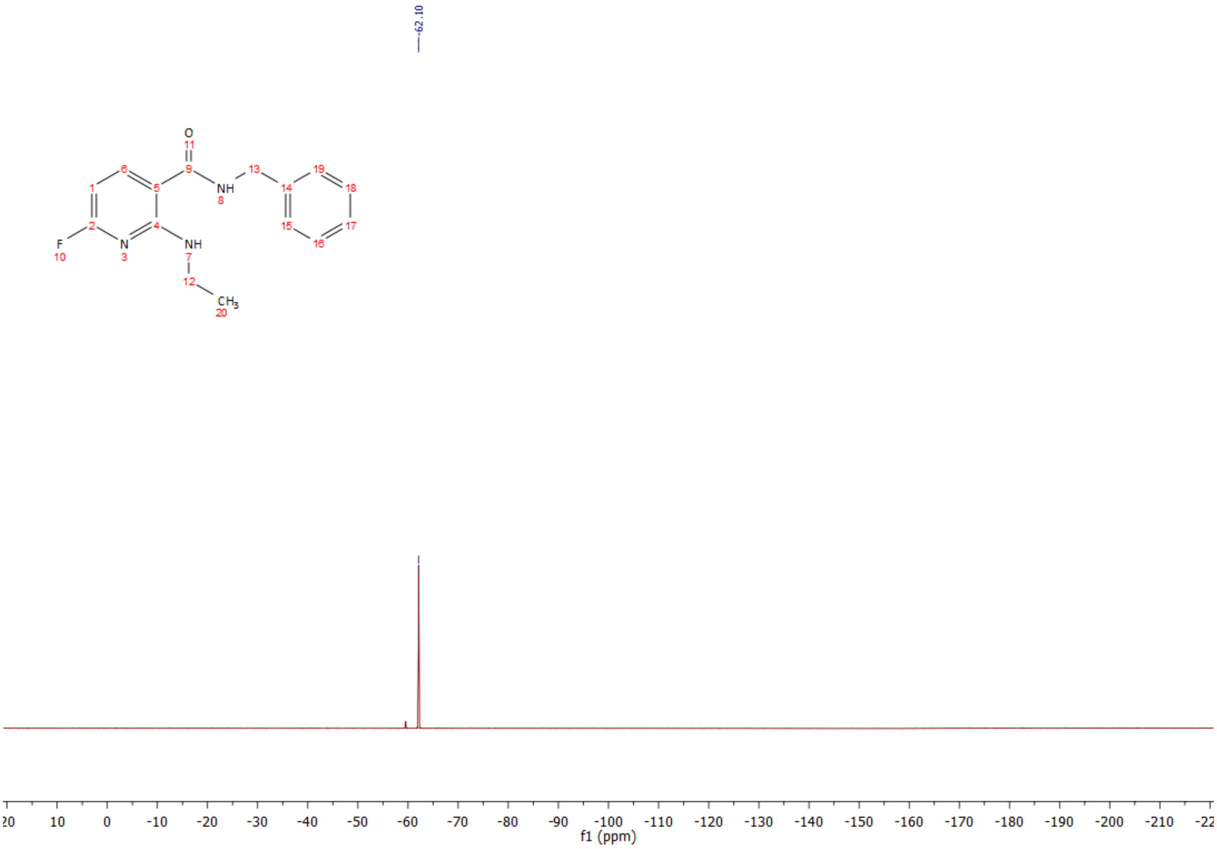
-62.10
f1 (ppm)

2.27 Compound **5g**

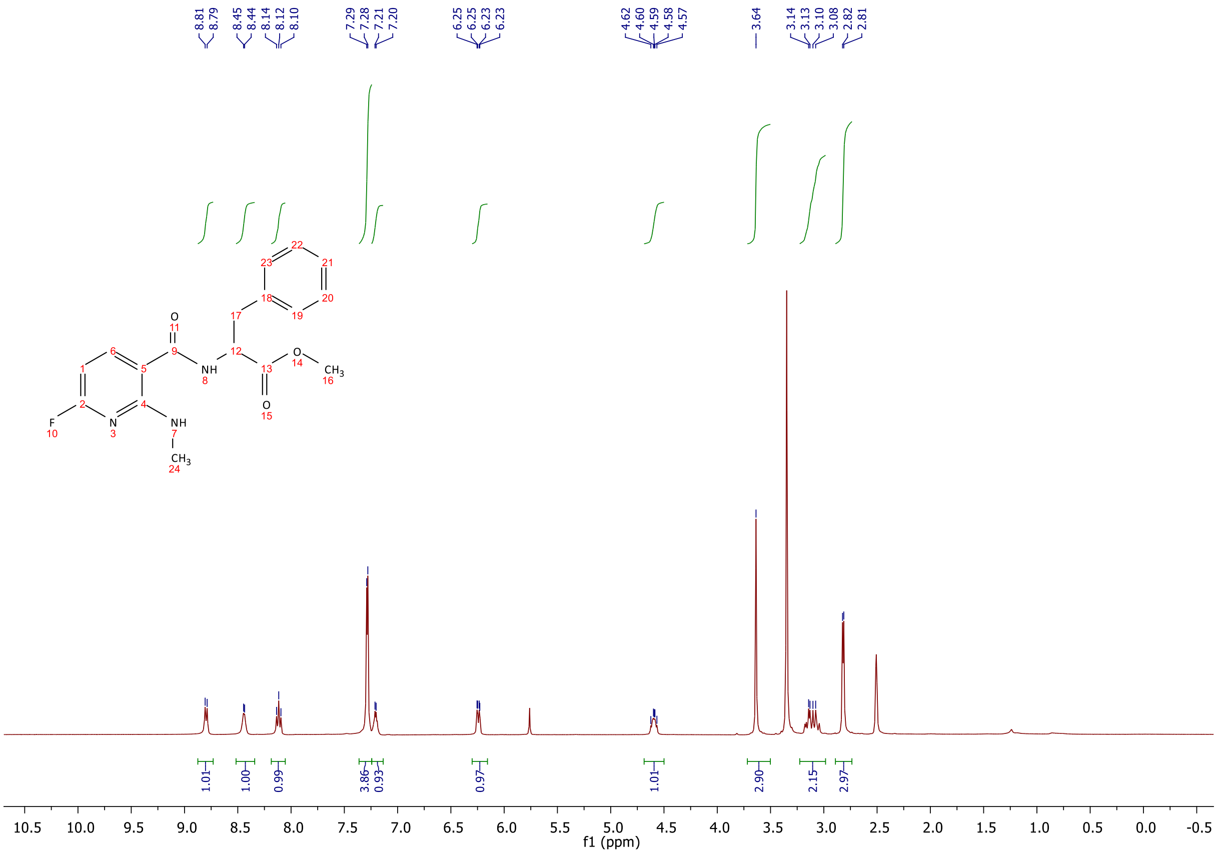

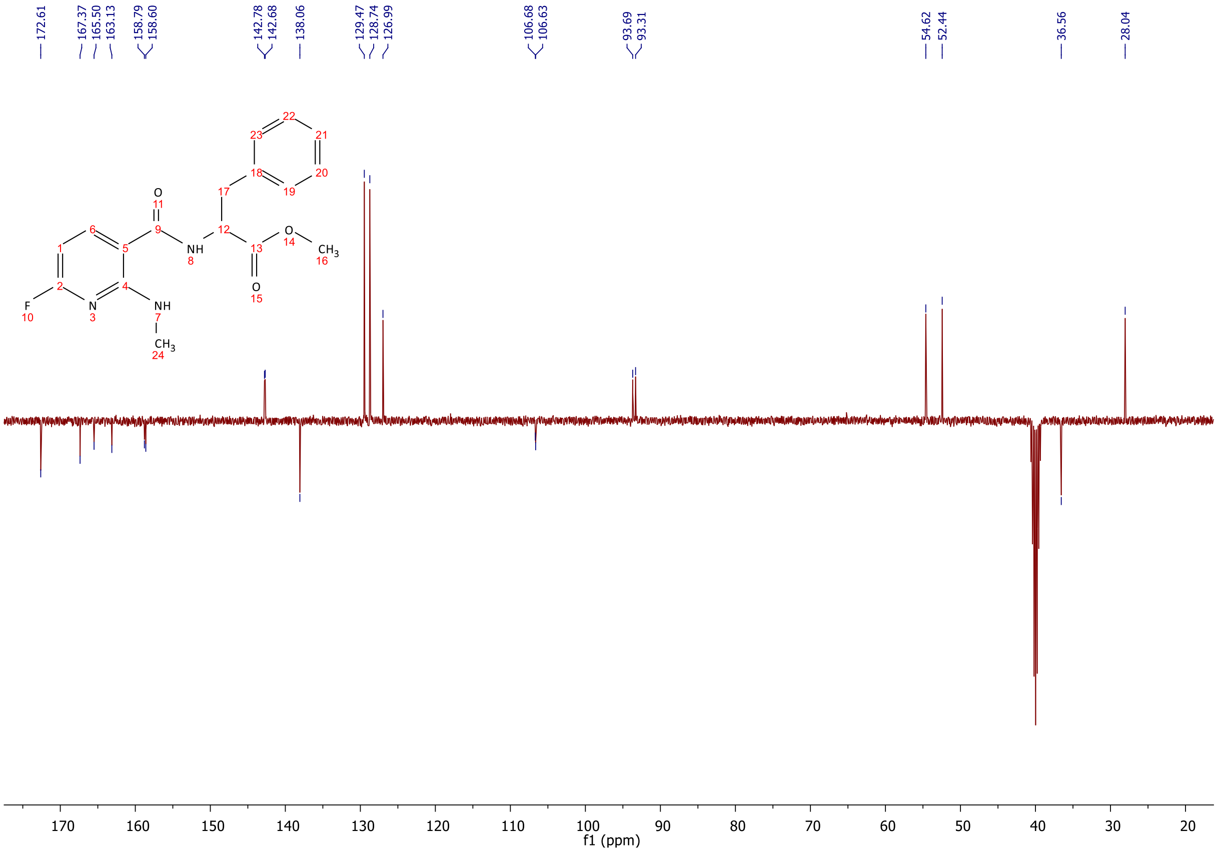

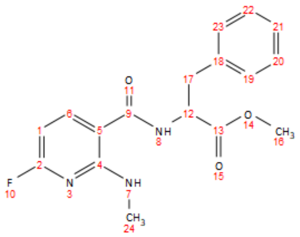

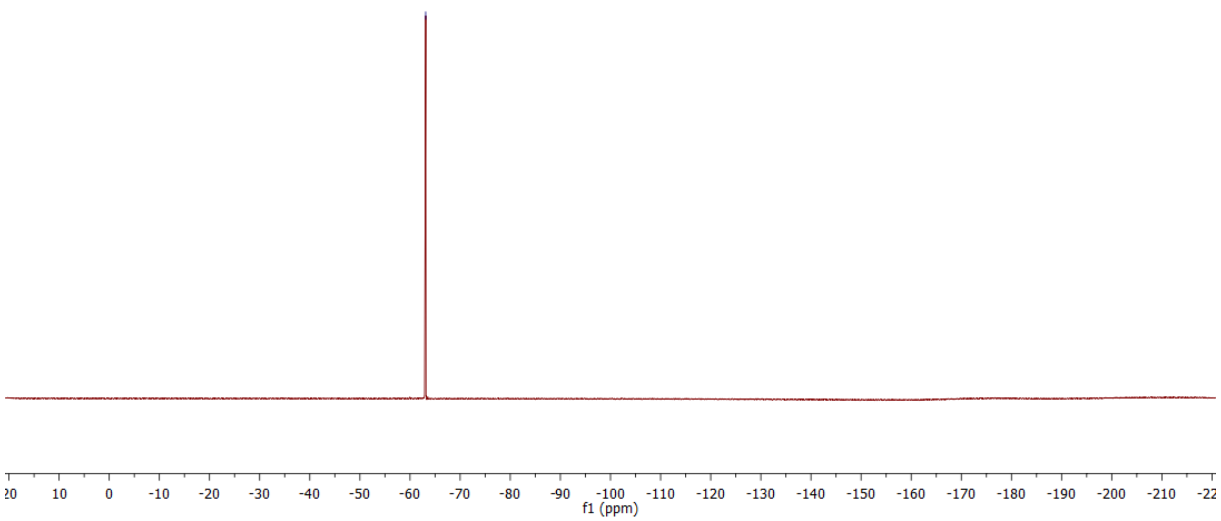
-63.13
20
10
0
-10
-20
-30
-40
-50
-60
-70
-80
-90
-100
-110
-120
-130
-140
-150
-160
-170
-180
-190
-200
-210
-22
f1 (ppm)

2.28 Compound **5h**

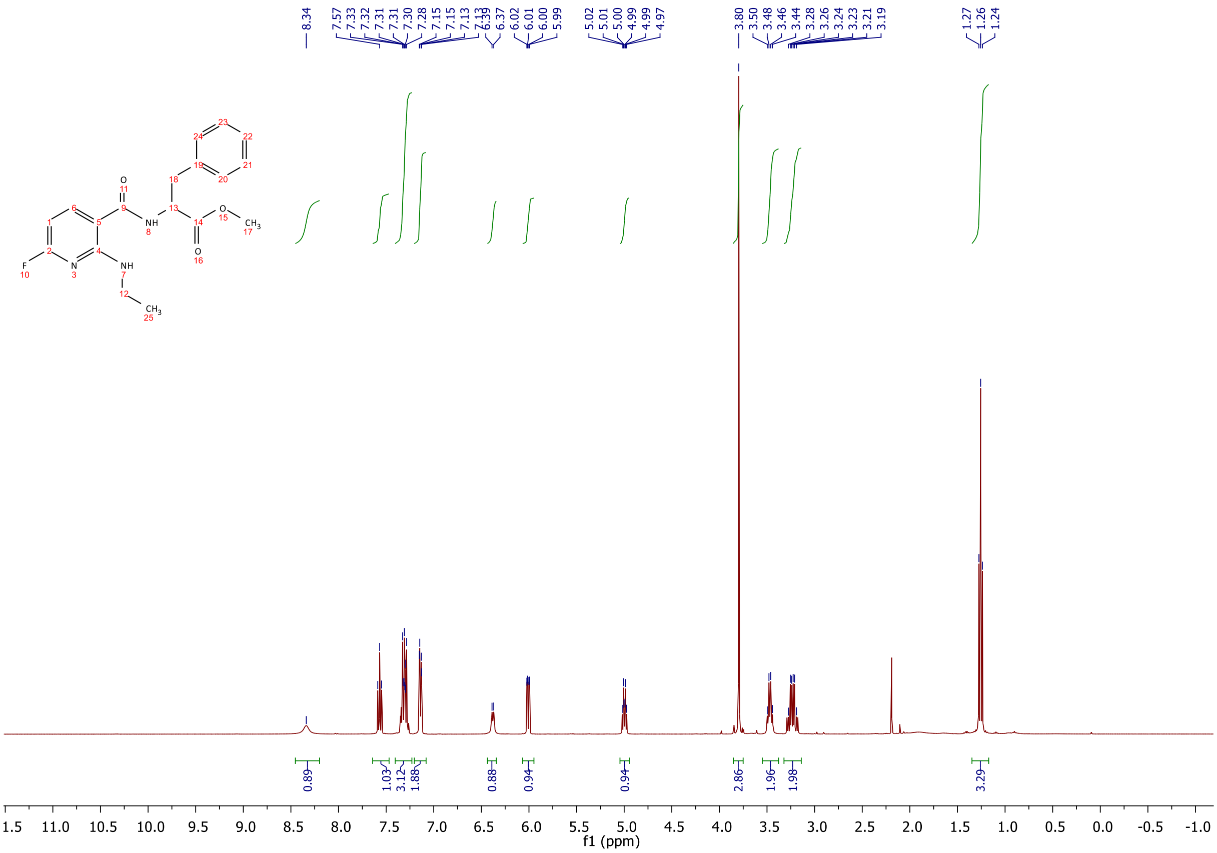

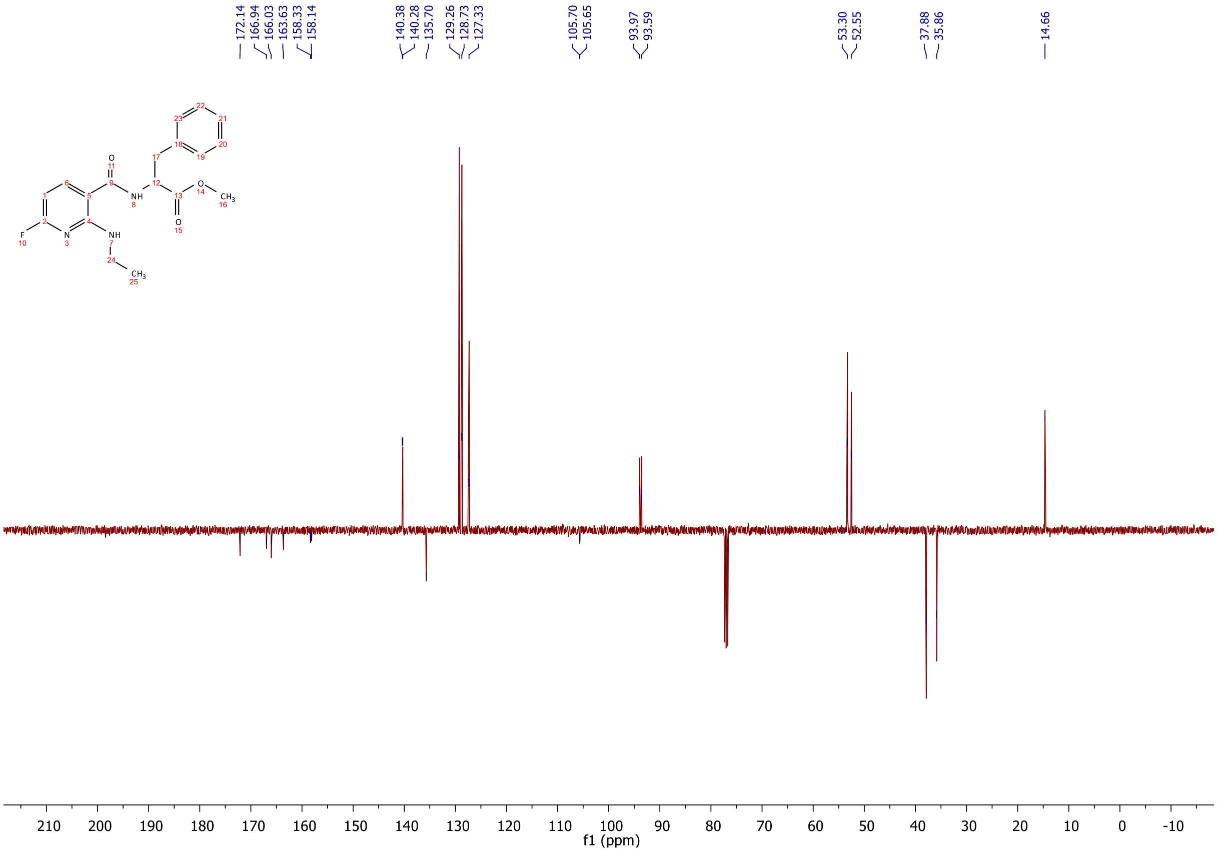

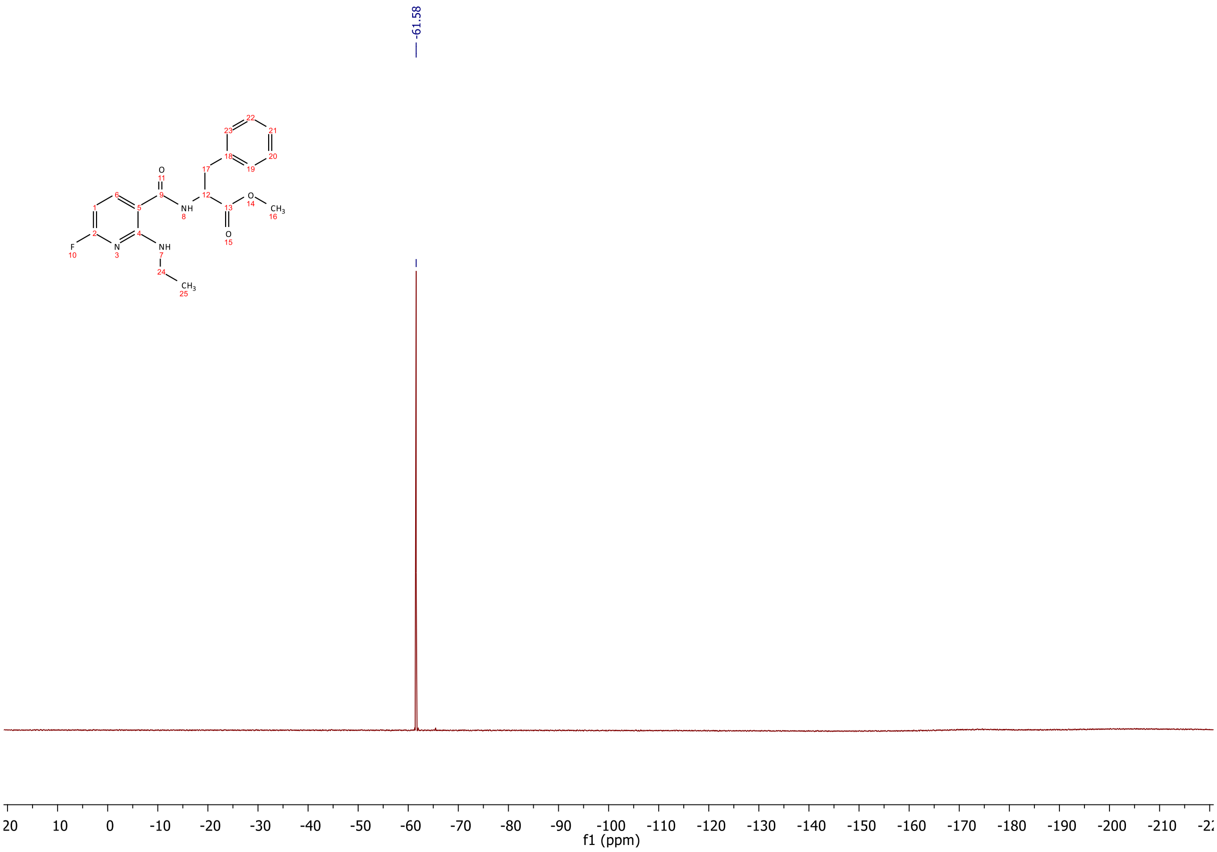

-61.58
20
10
0
-10
-20
-30
-40
-50
-60
-70
-80
-90
-100
-110
-120
-130
-140
-150
-160
-170
-180
-190
-200
-210
f1 (ppm)

2.29 Compound **5i**

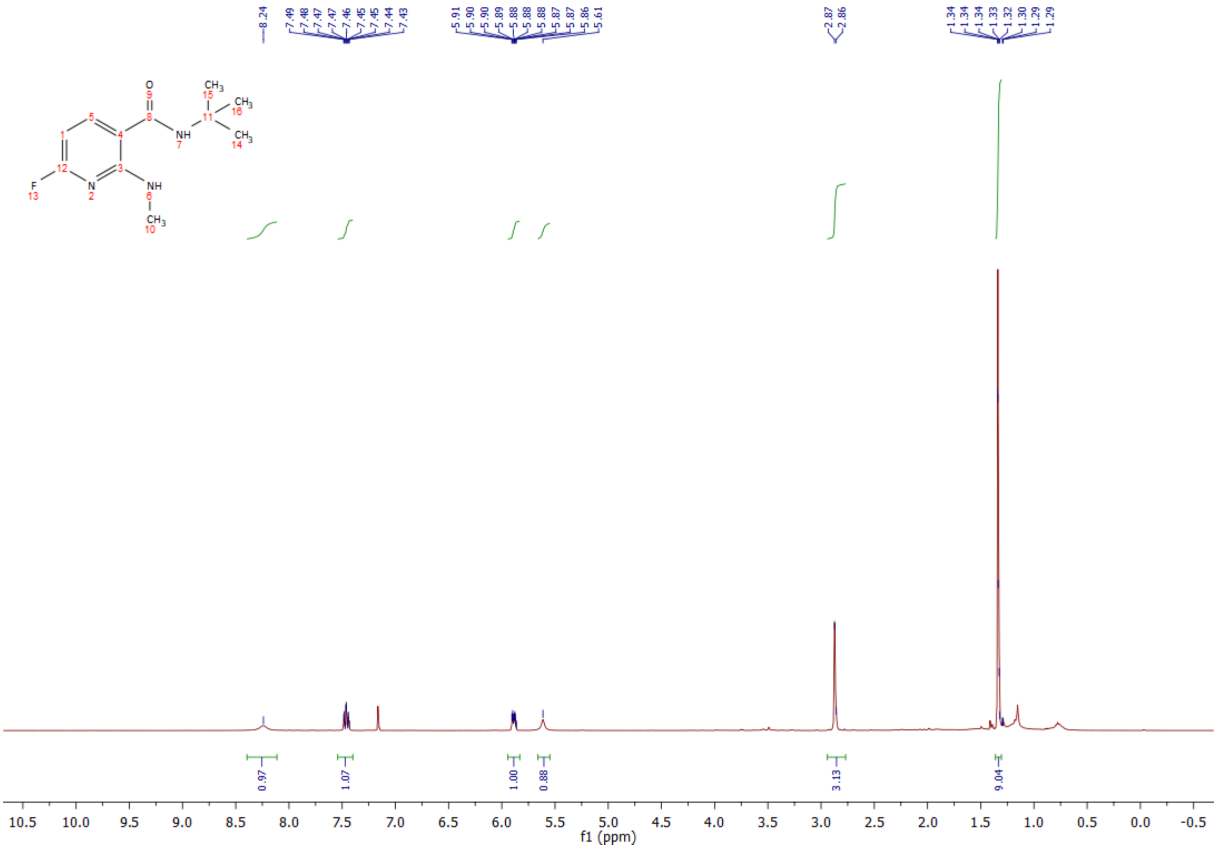

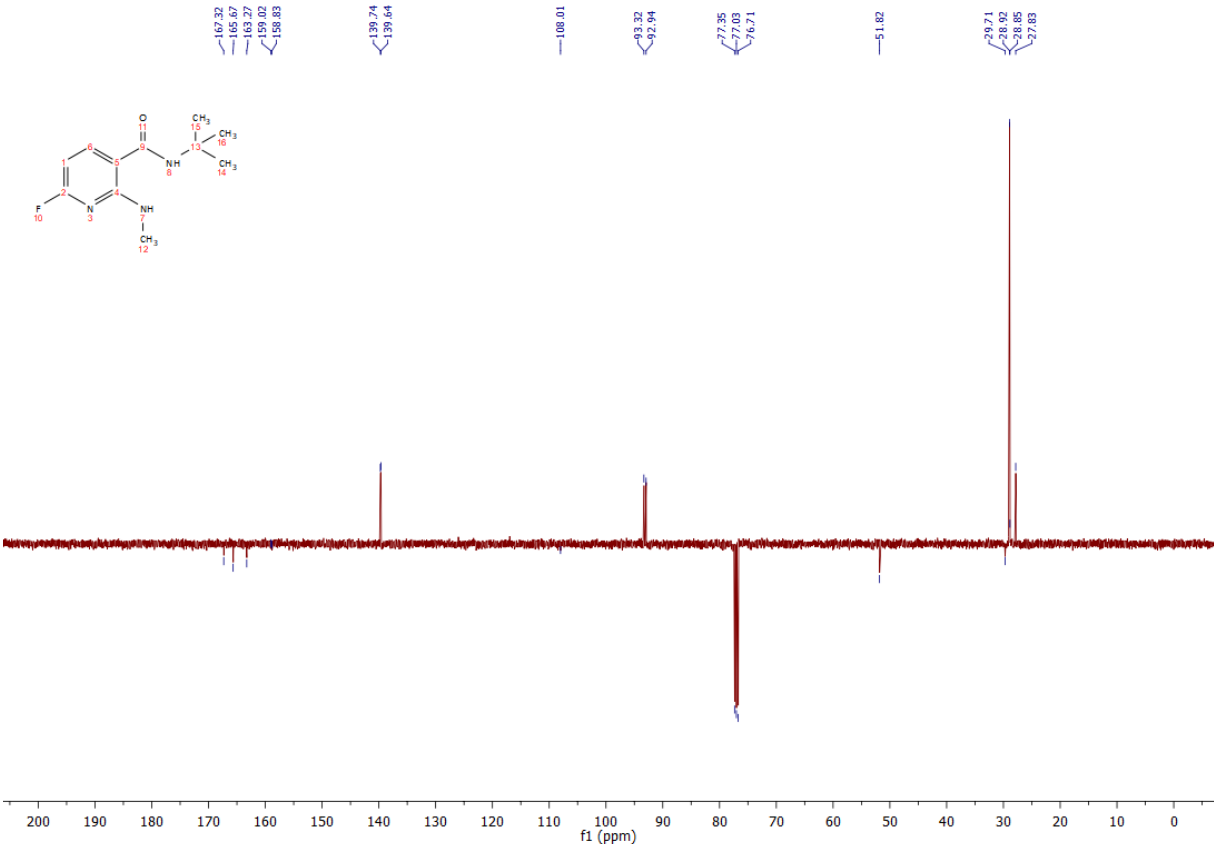

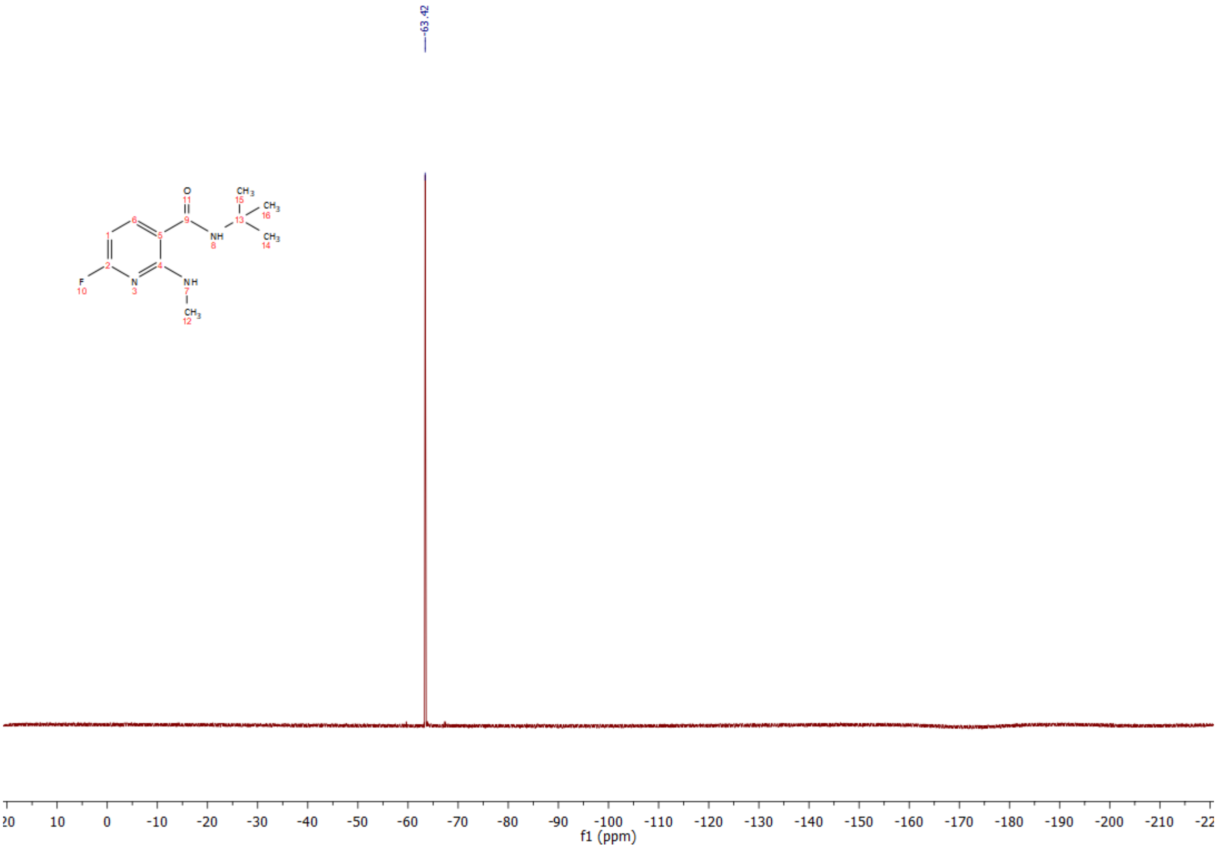
-63.42
f1 (ppm)

2.30 Compound **5j**

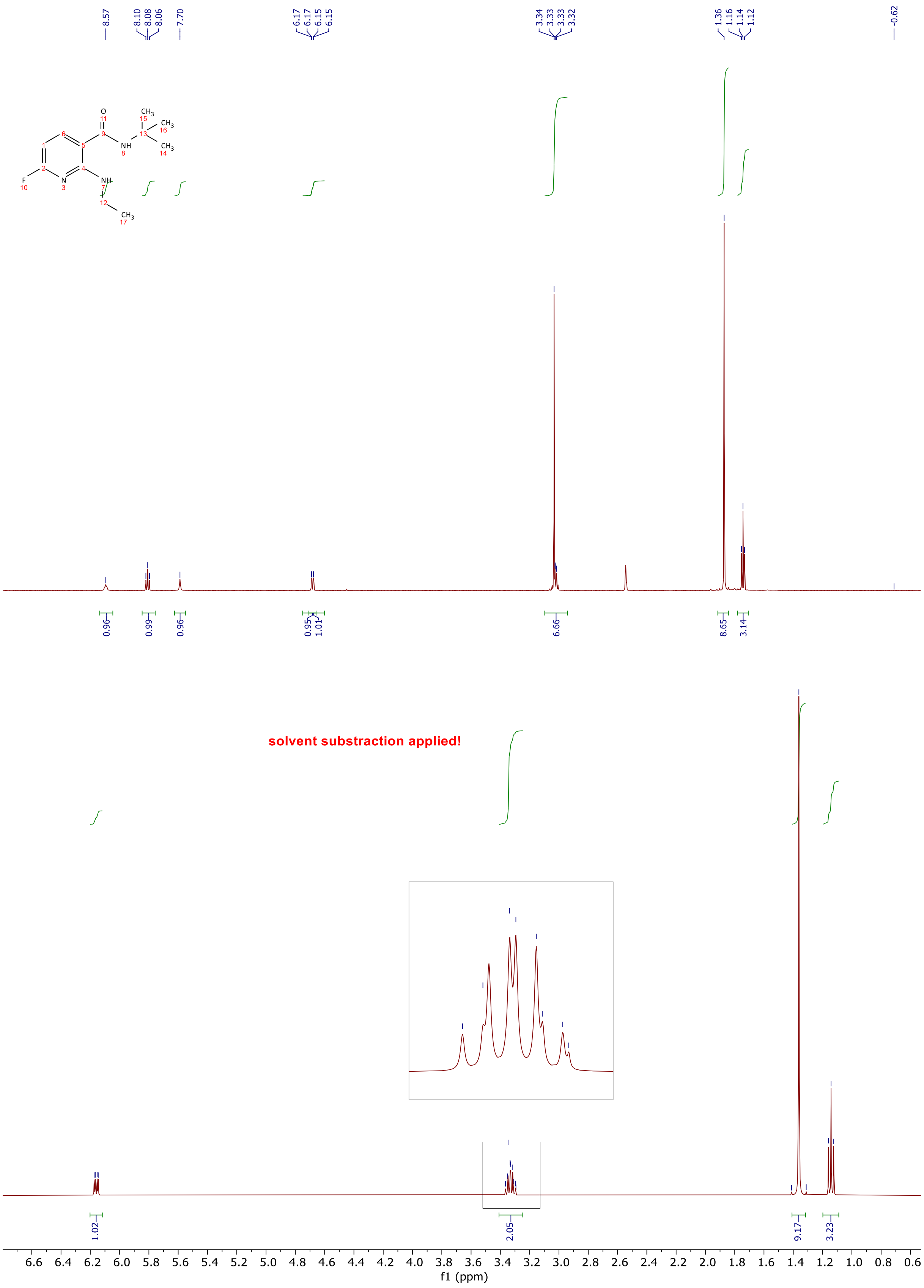

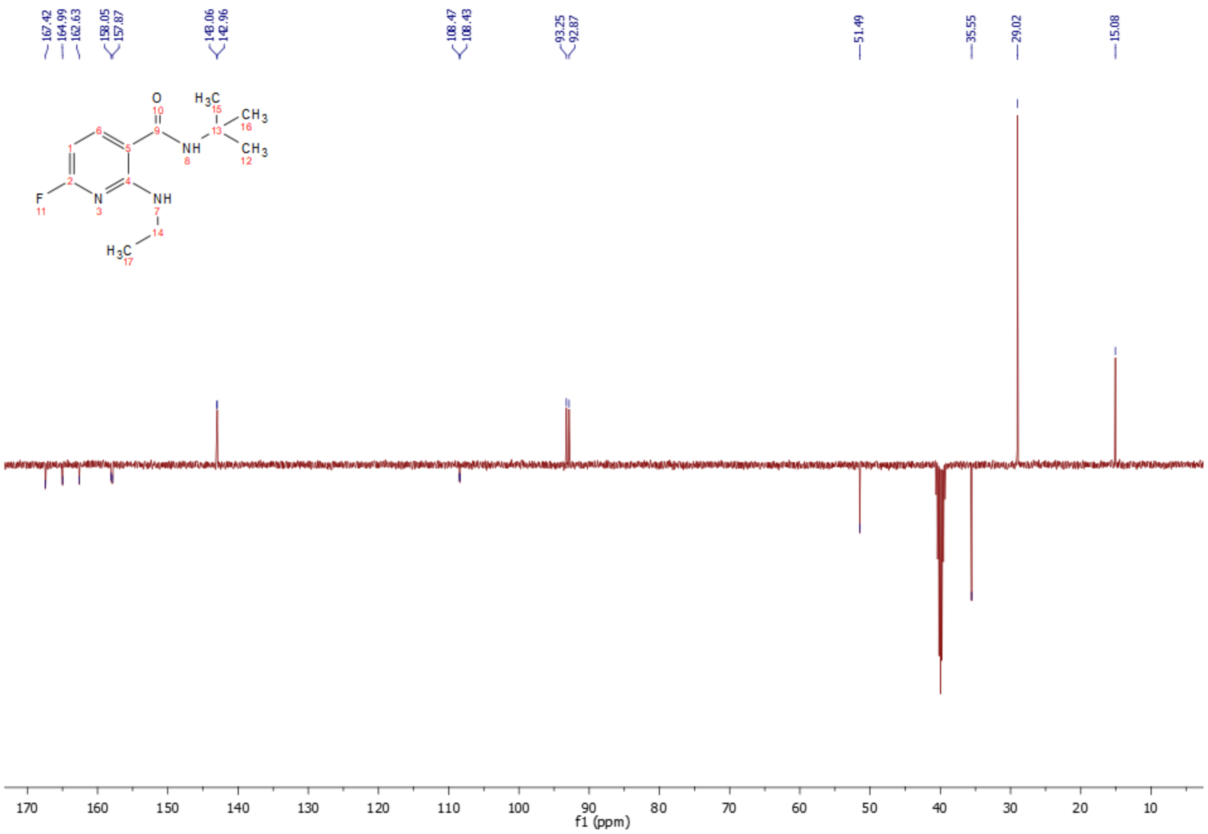
167.42
164.99
162.63
158.05
157.87
143.06
142.96
108.47
108.43
93.25
92.87
51.49
35.55
29.02
15.08
f1 (ppm)

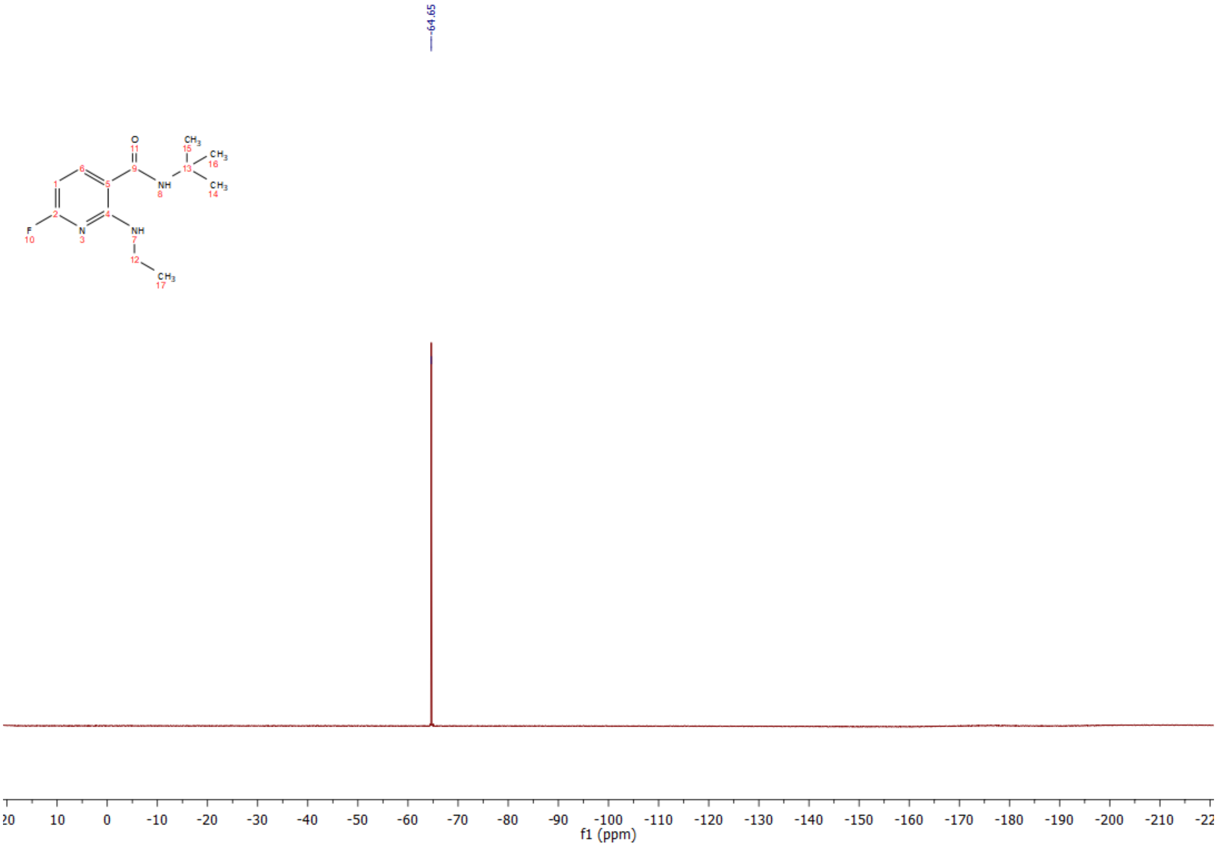
-64.65
f1 (ppm)

2.31 Compound **5k**

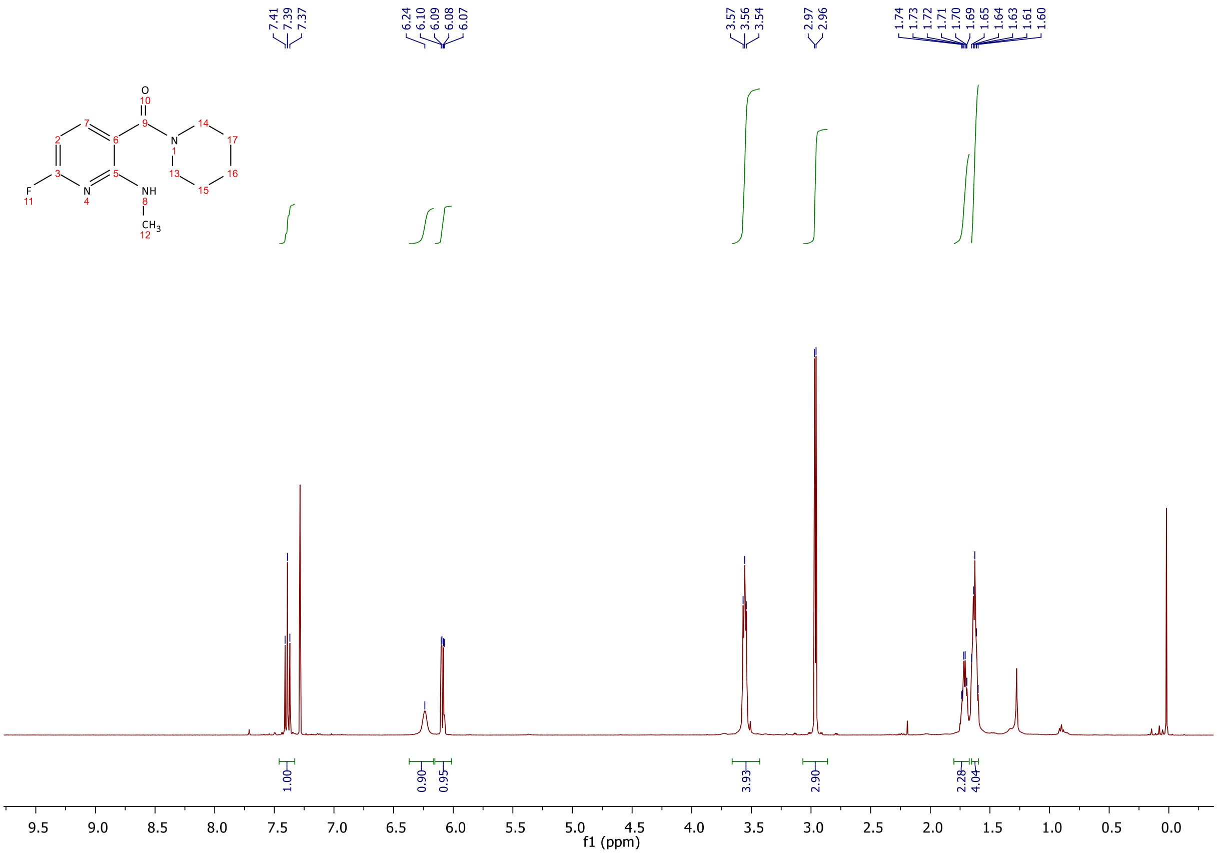

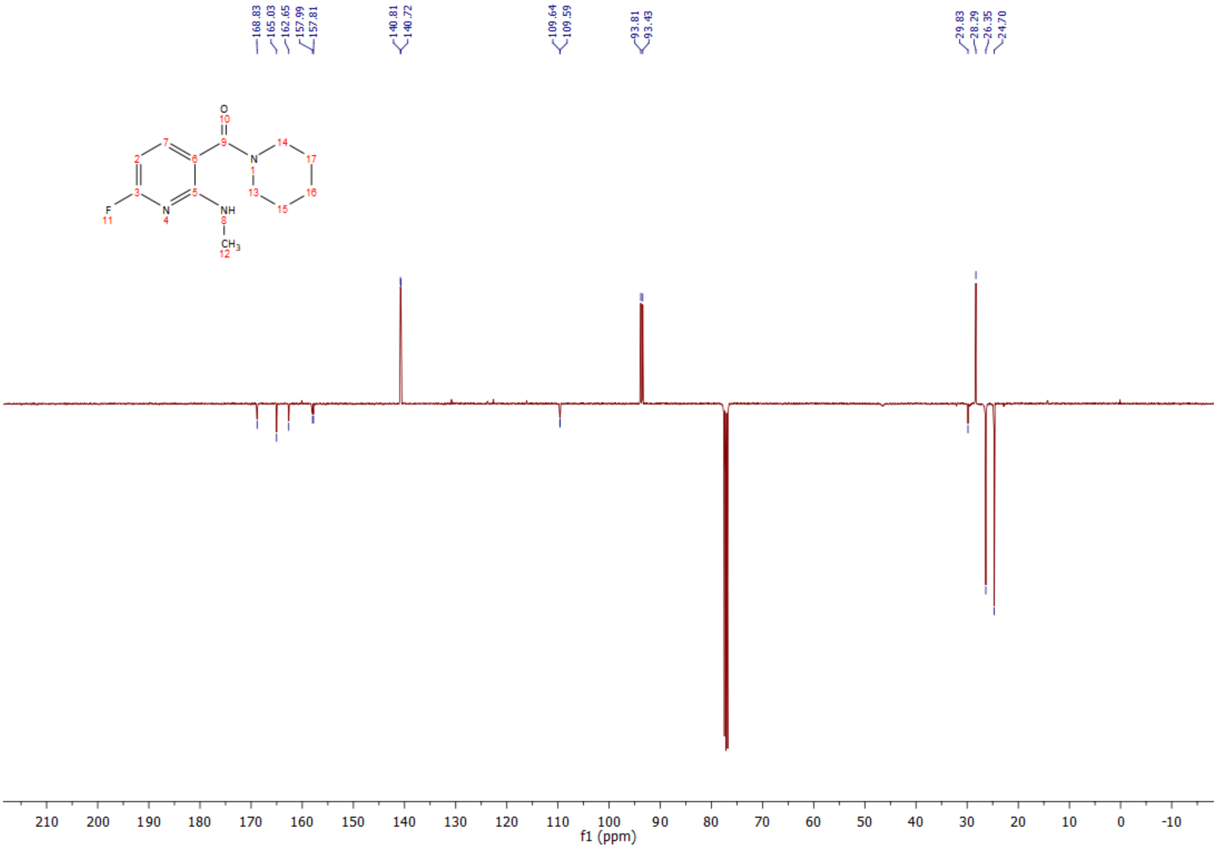

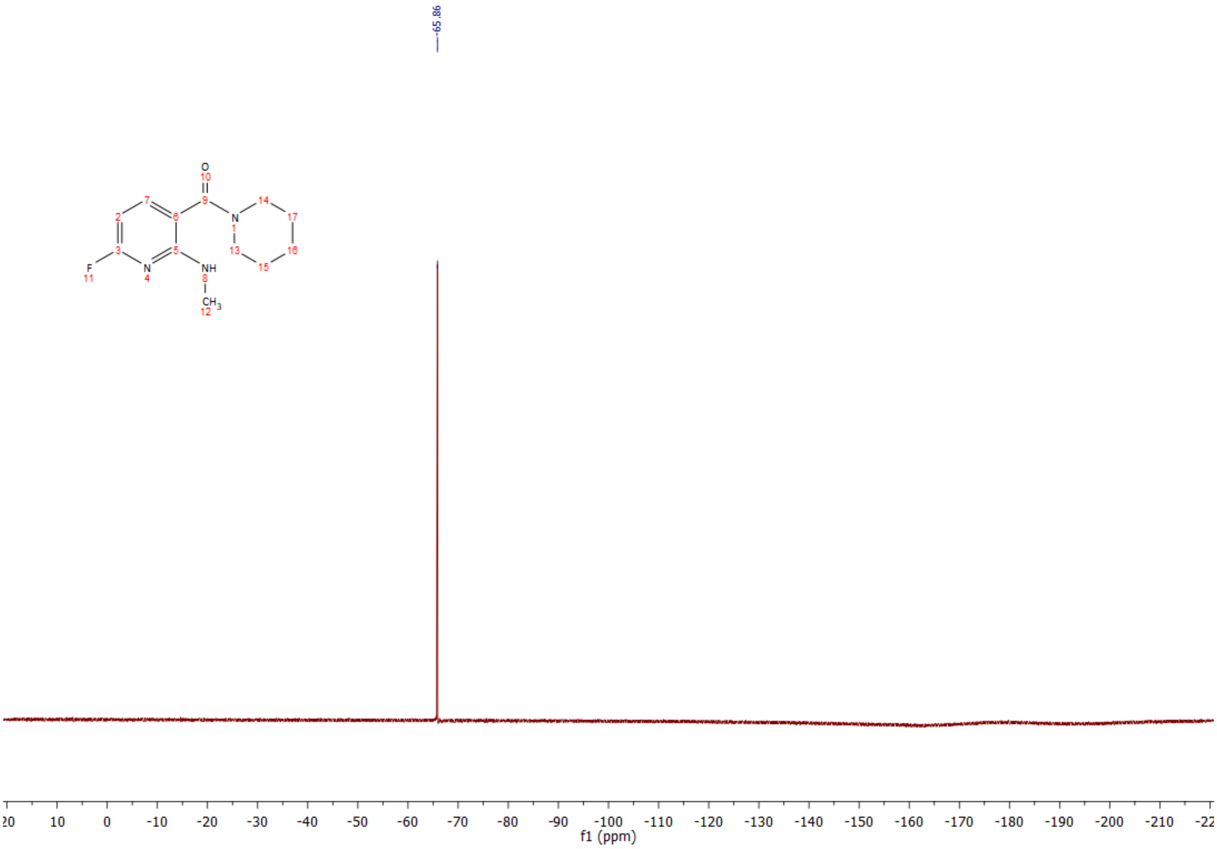

65.86
O
10
9
7
6
2
3
F
11
N
4
5
NH
8
CH3
12
N
1
14
17
16
15
13
f1 (ppm)
20
10
0
-10
-20
-30
-40
-50
-60
-70
-80
-90
-100
-110
-120
-130
-140
-150
-160
-170
-180
-190
-200
-210
-22

2.32 Compound **5l**

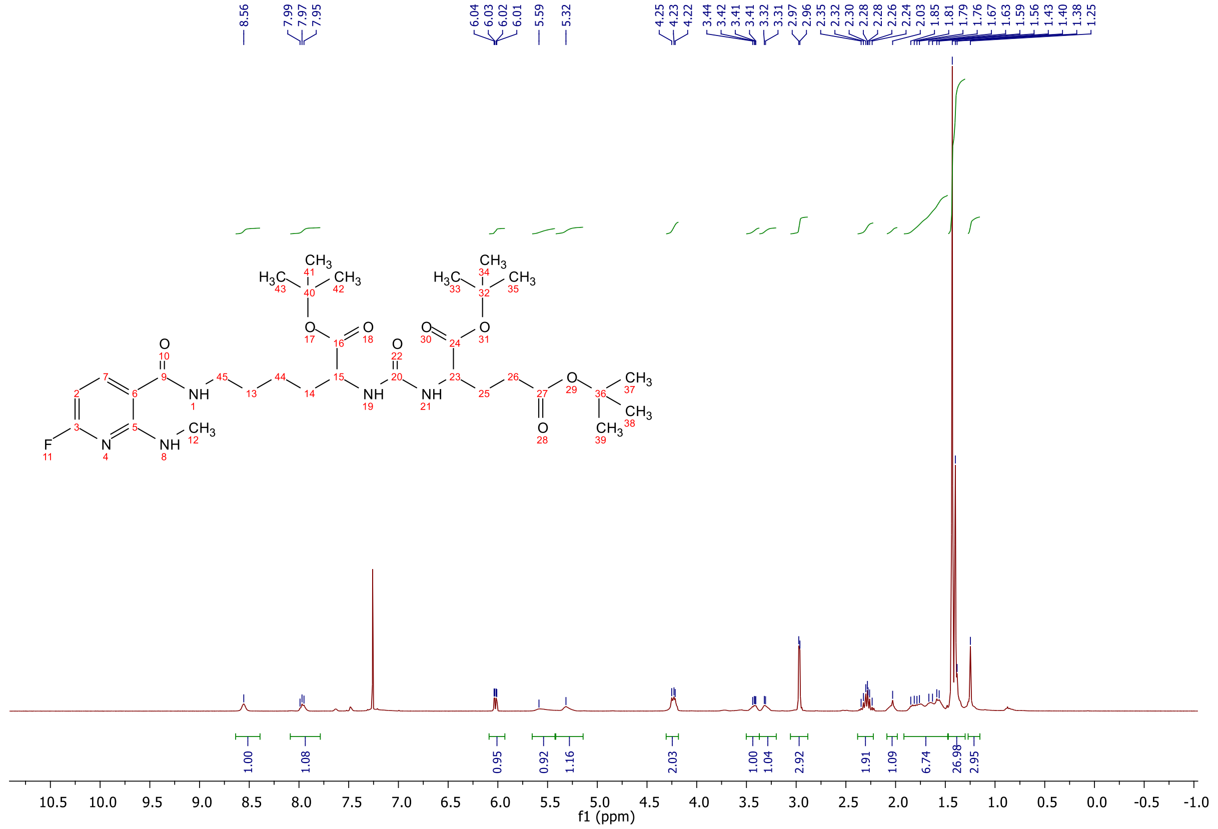

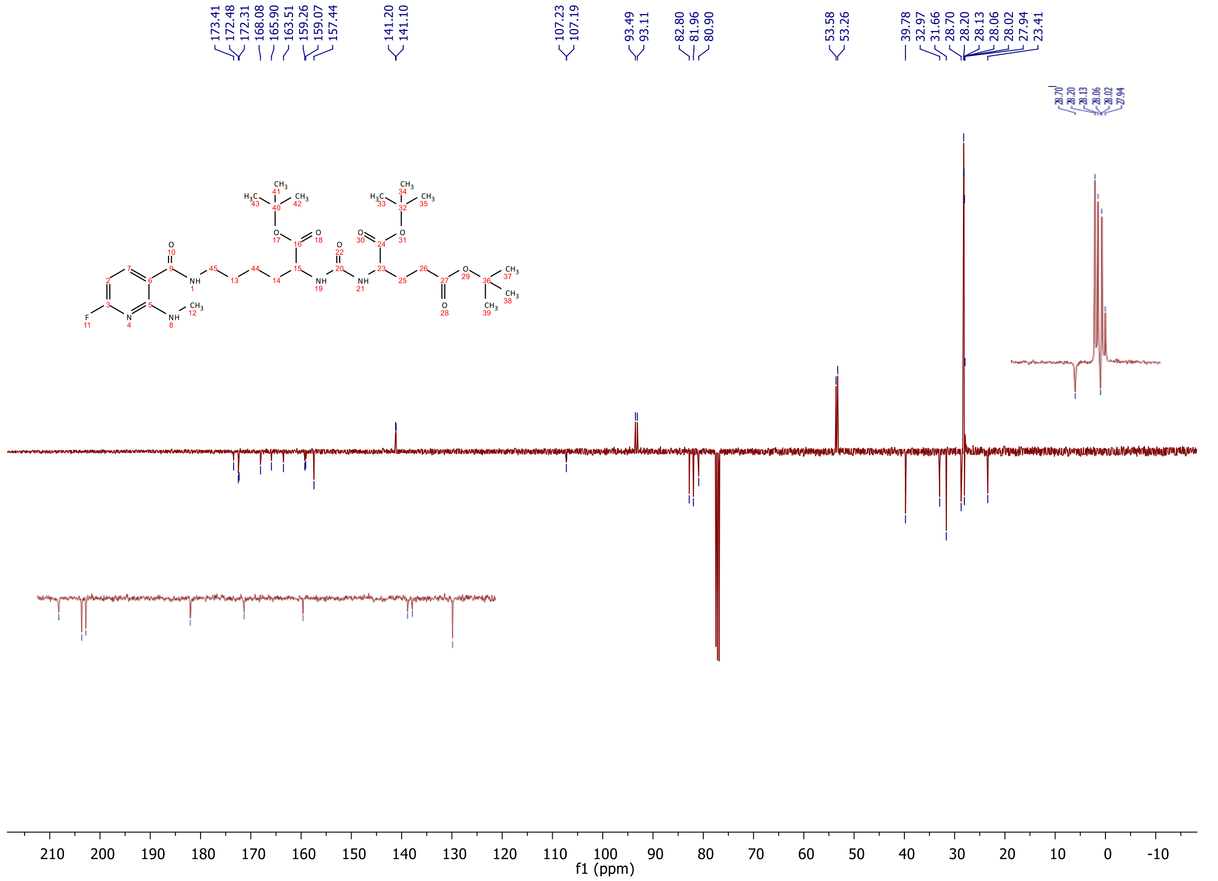

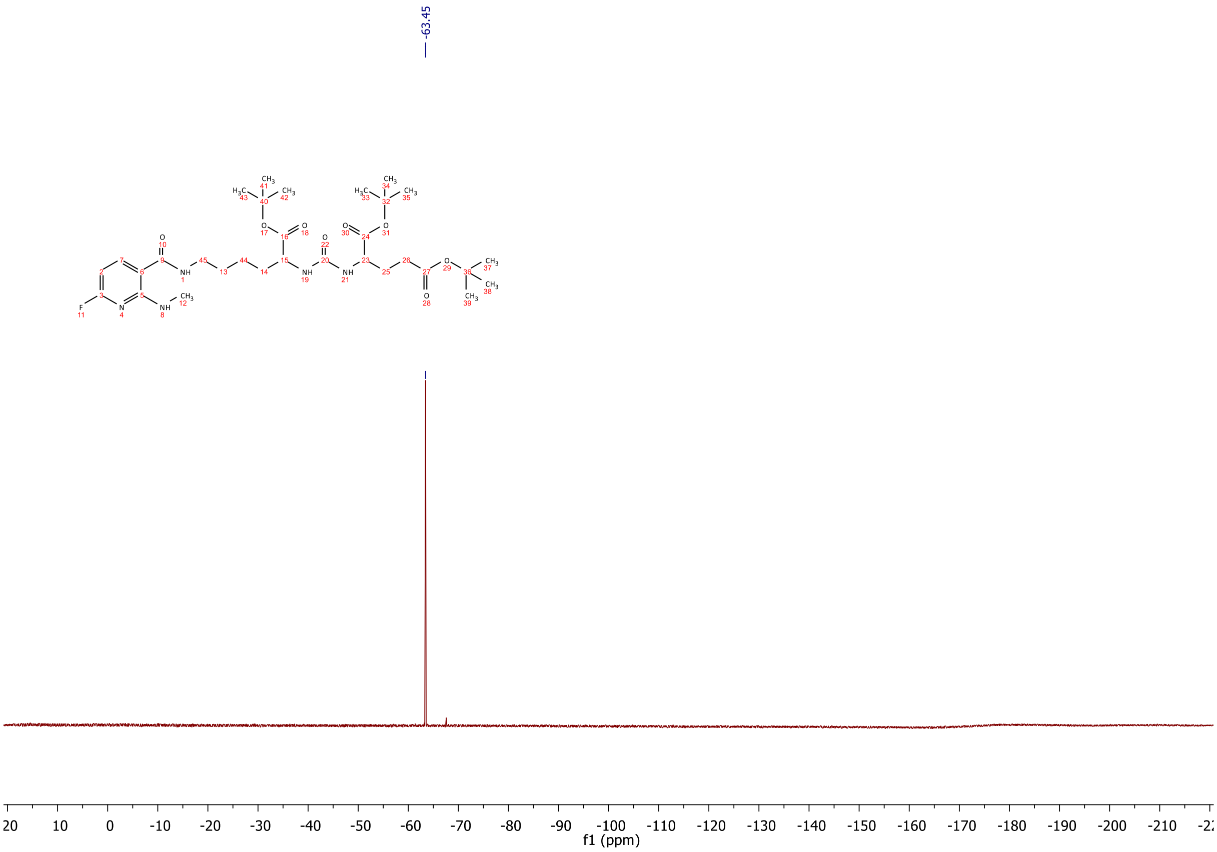
-63.45
f1 (ppm)

2.33 Compound **5m**

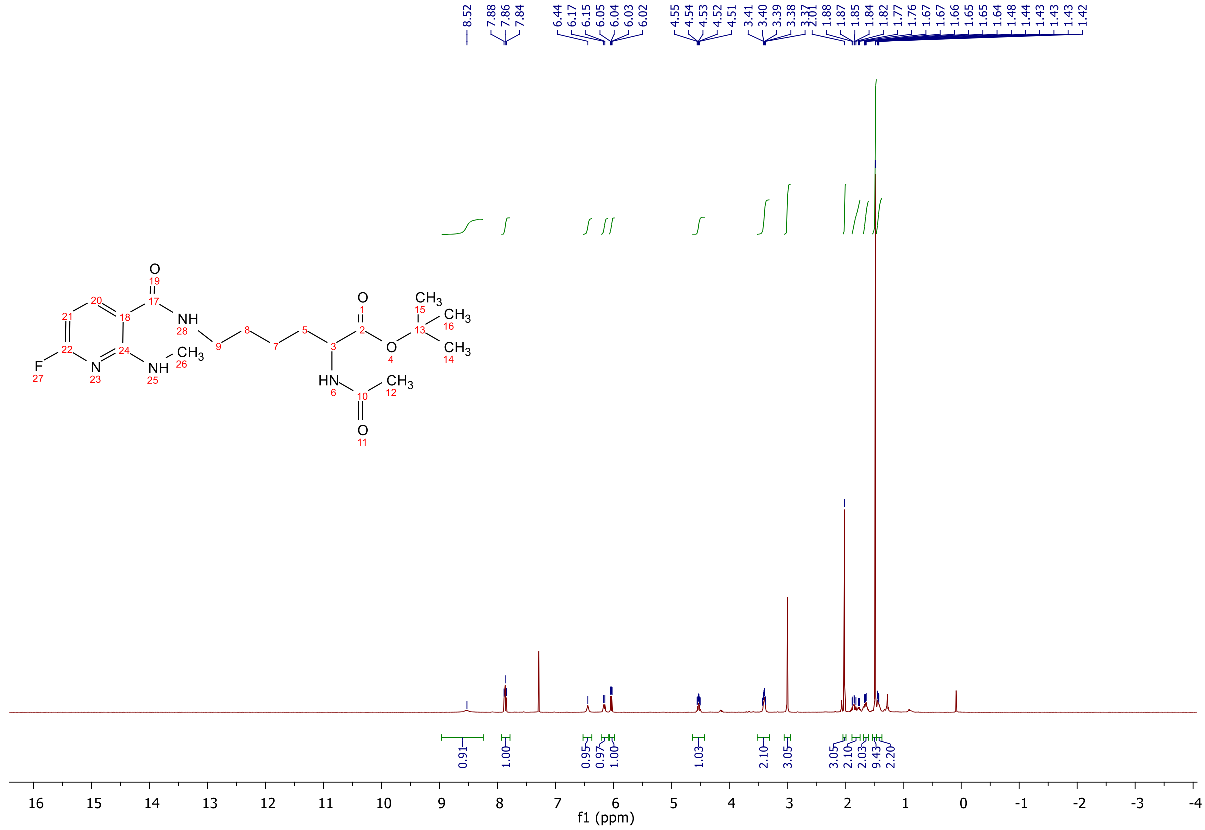

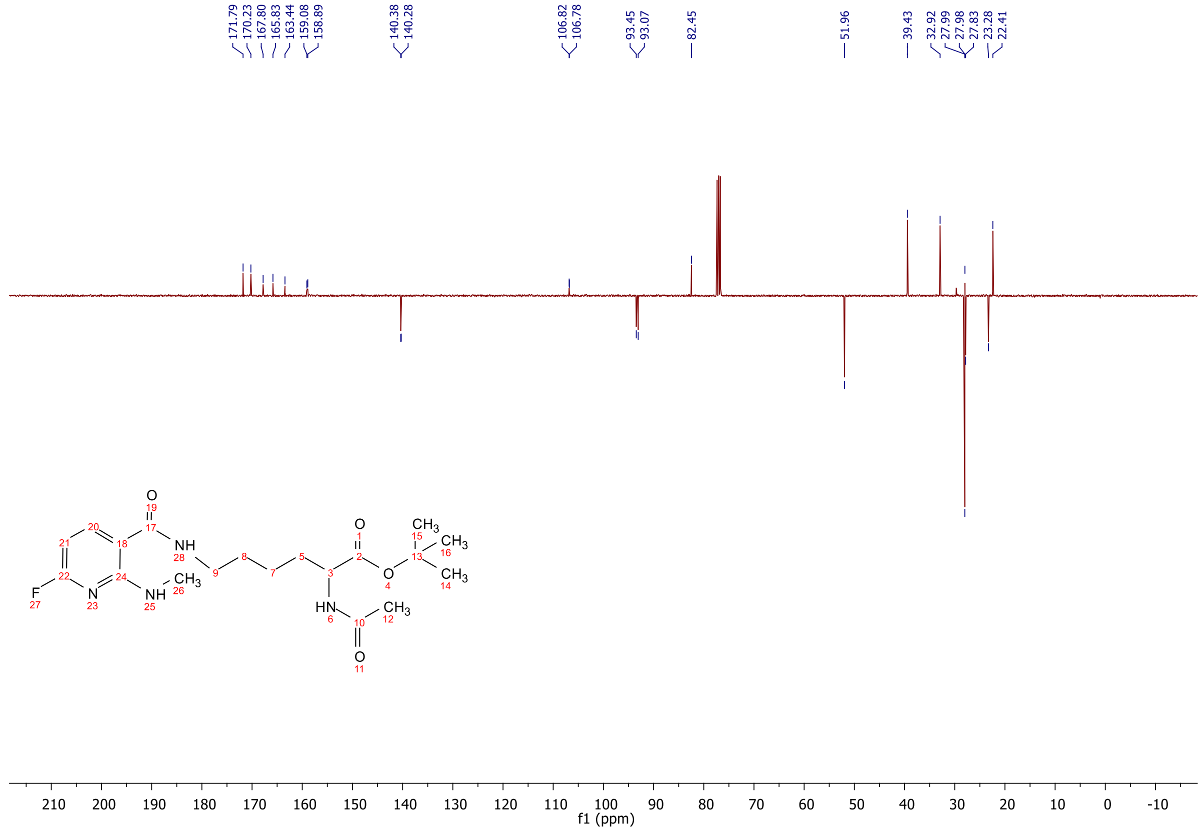

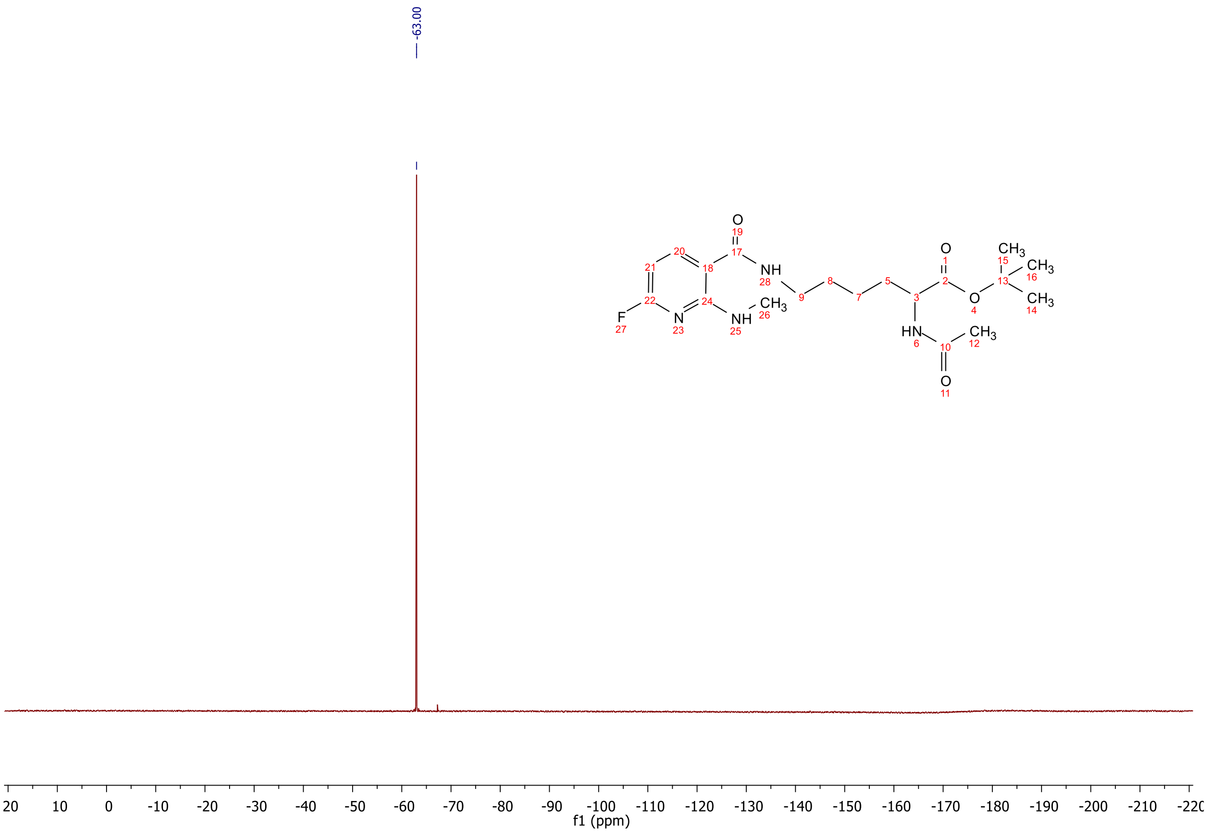
-63.00
20 10 0 -10 -20 -30 -40 -50 -60 -70 -80 -90 -100 -110 -120 -130 -140 -150 -160 -170 -180 -190 -200 -210 -220
f1 (ppm)

2.34 Compound **5n**

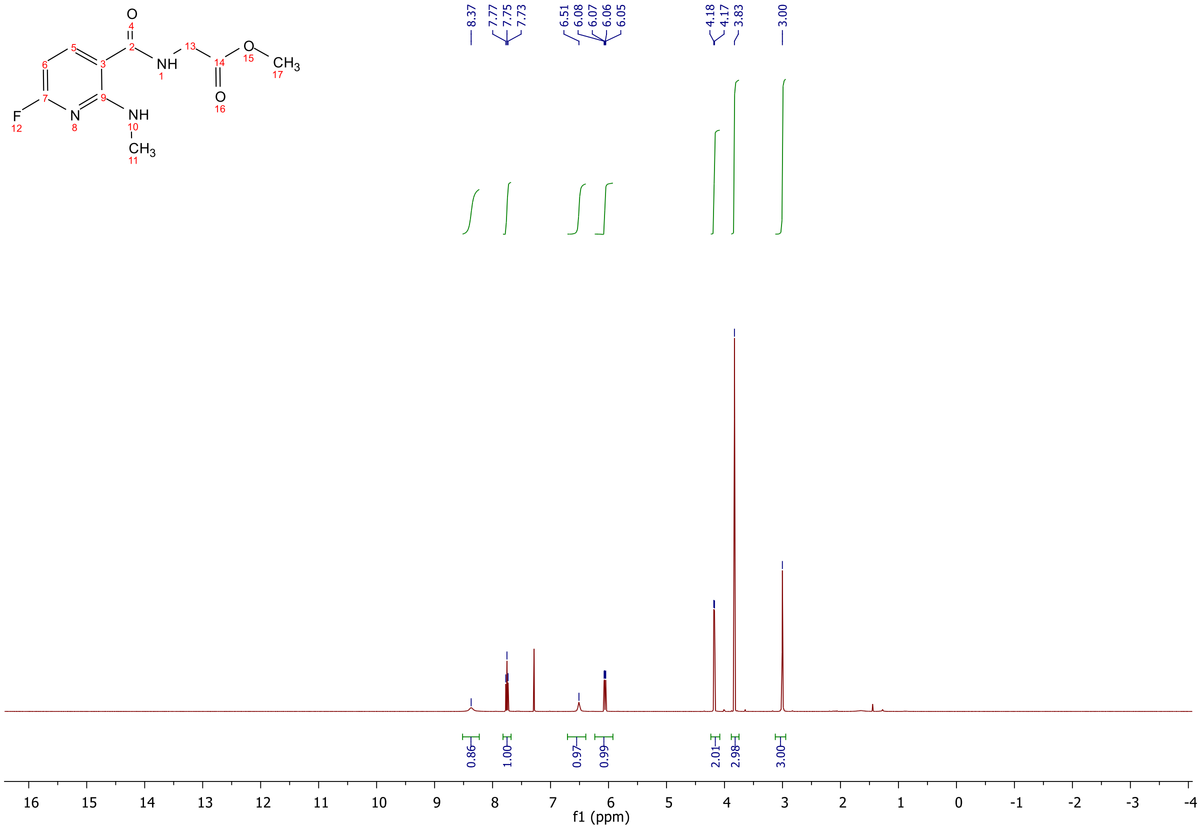

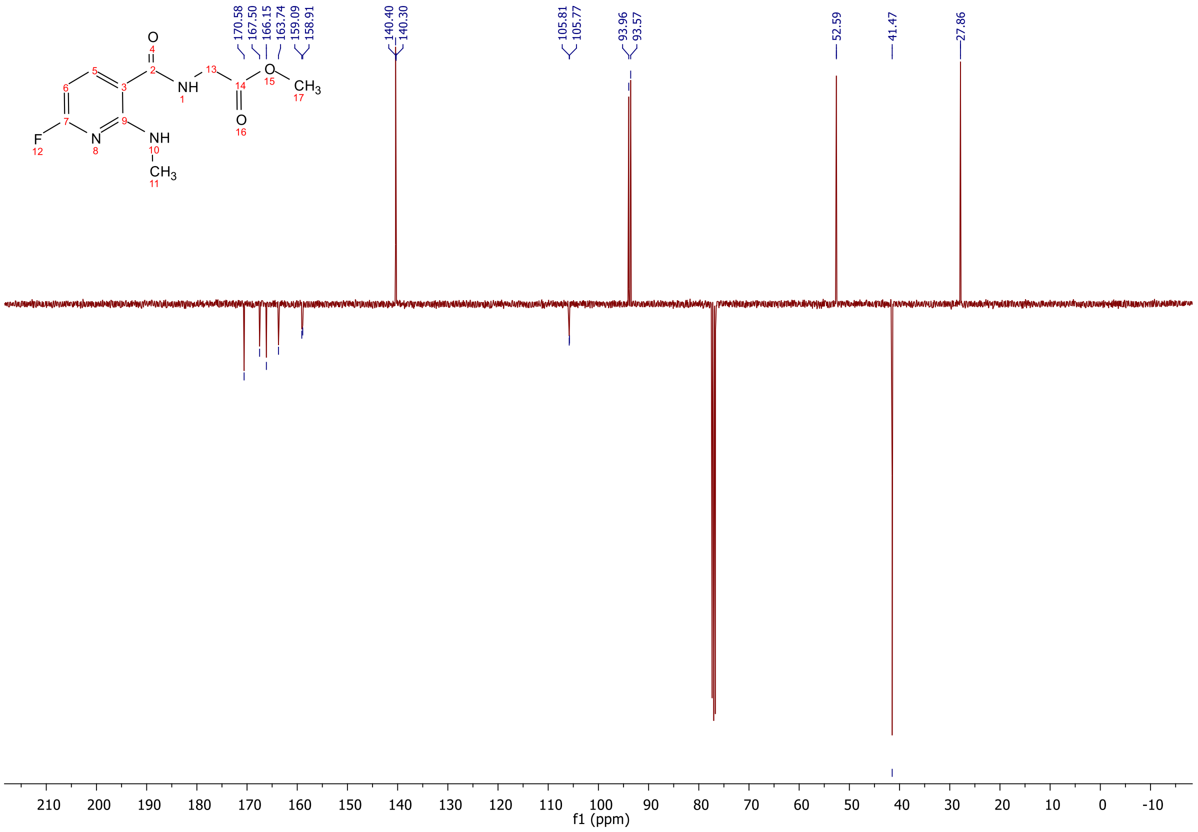

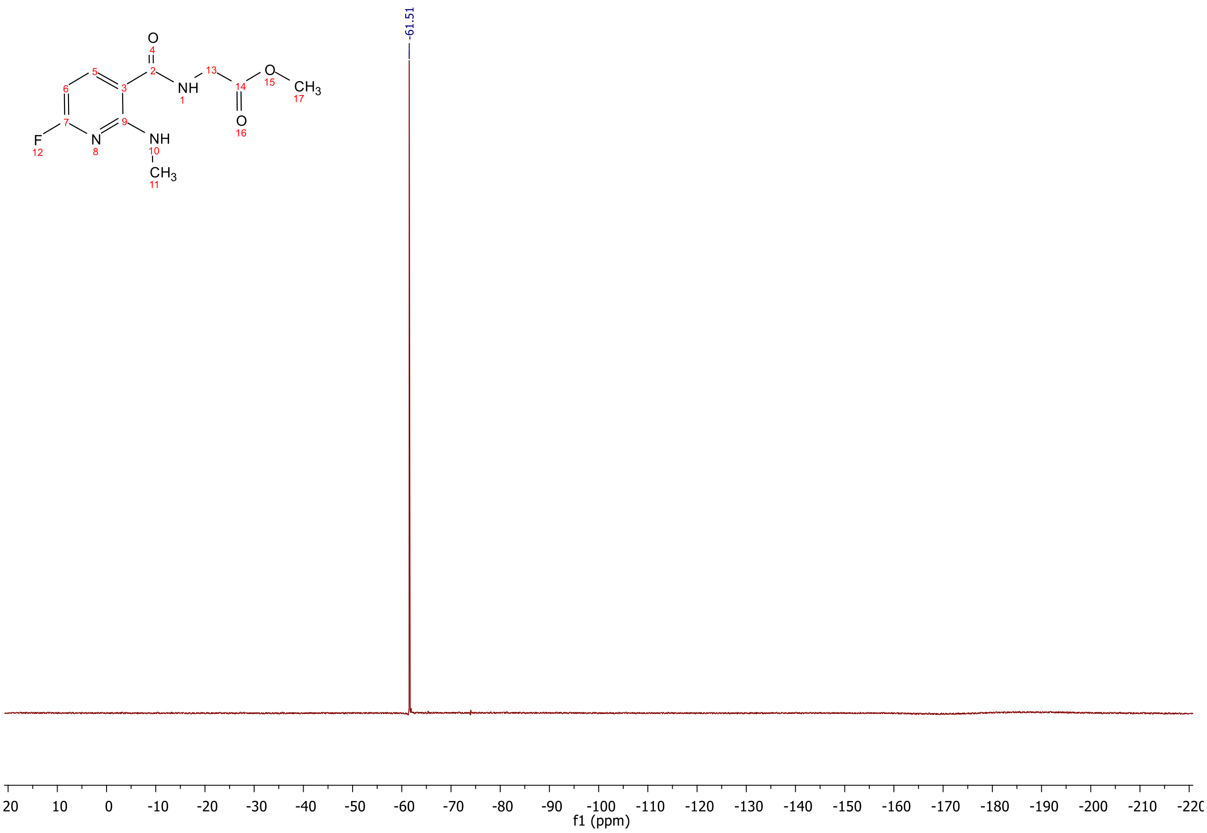
O
NH
O
CH3
F
N
NH
CH3
O
-61.51
20
10
0
-10
-20
-30
-40
-50
-60
-70
-80
-90
-100
-110
-120
-130
-140
-150
-160
-170
-180
-190
-200
-210
-220
f1 (ppm)

2.35 Compound **5o**

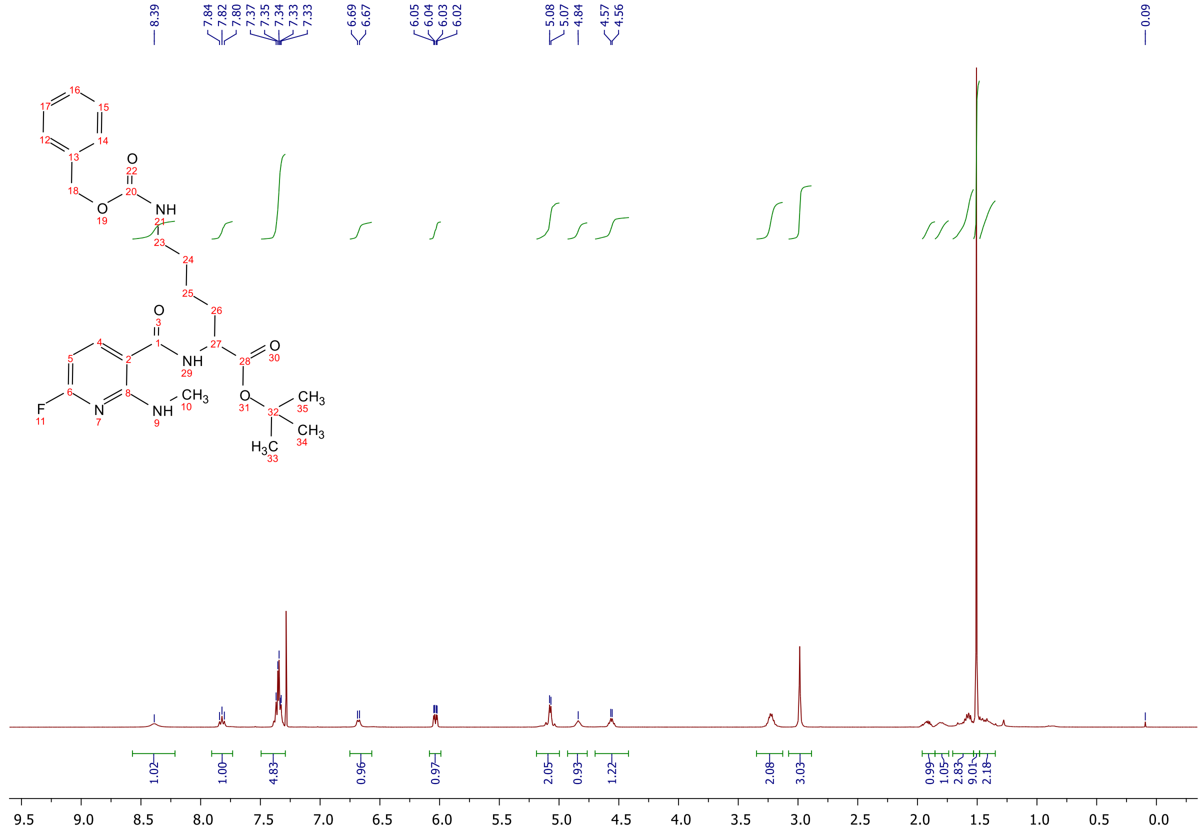

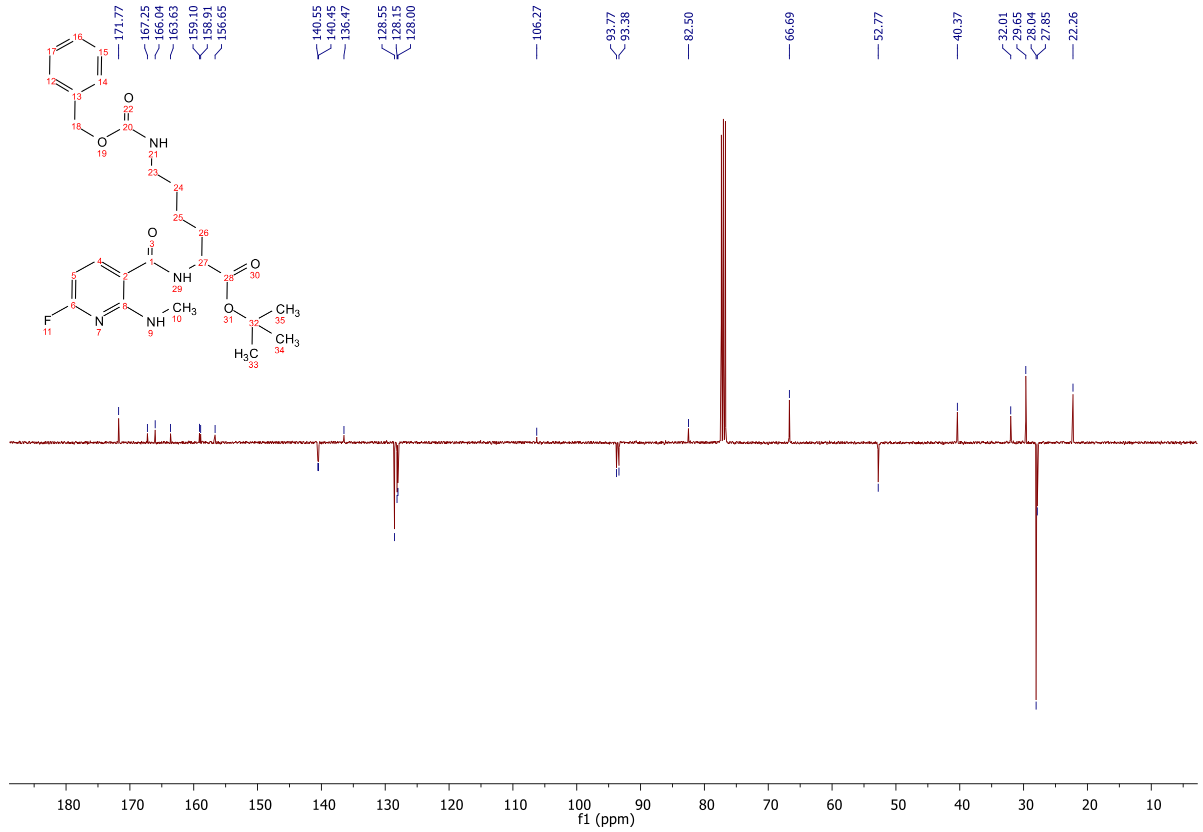

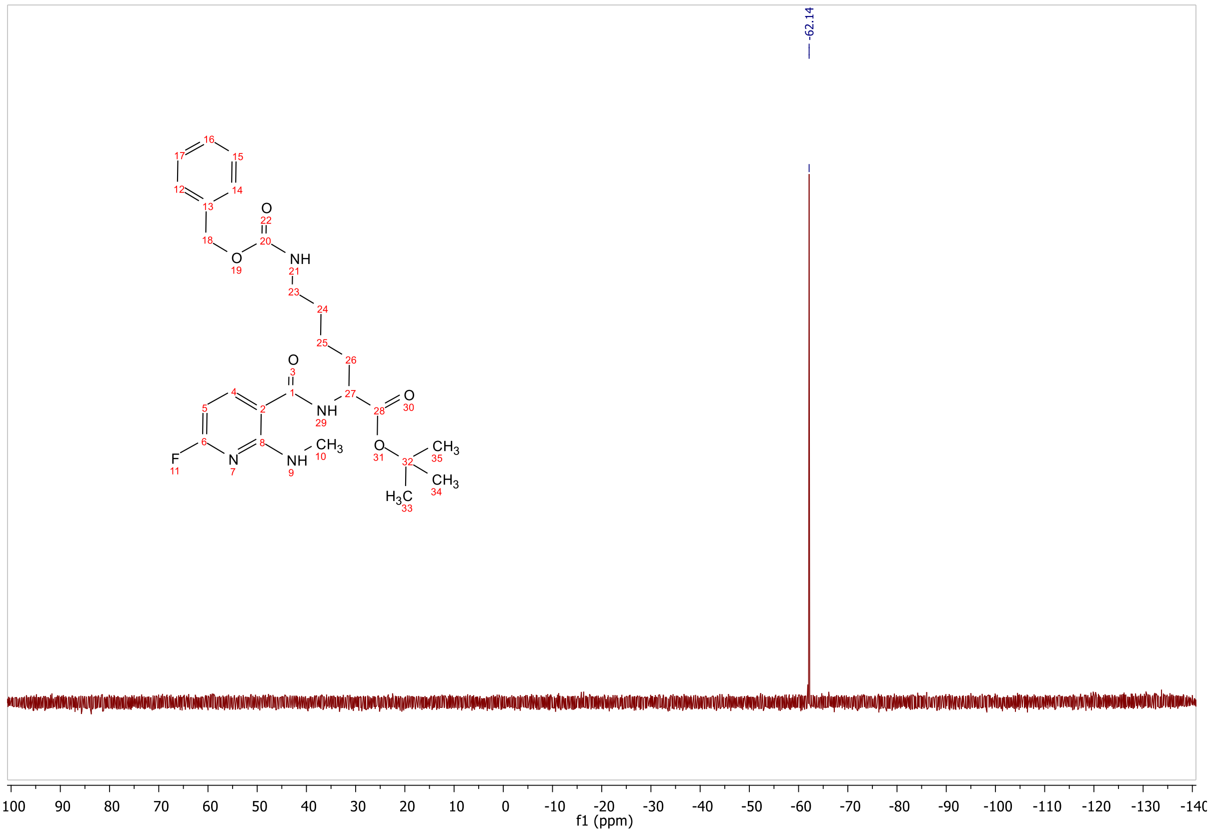
-62.14
f1 (ppm)
100 90 80 70 60 50 40 30 20 10 0 -10 -20 -30 -40 -50 -60 -70 -80 -90 -100 -110 -120 -130 -140

2.36 Compound **5p** (JK-PSMA-15)

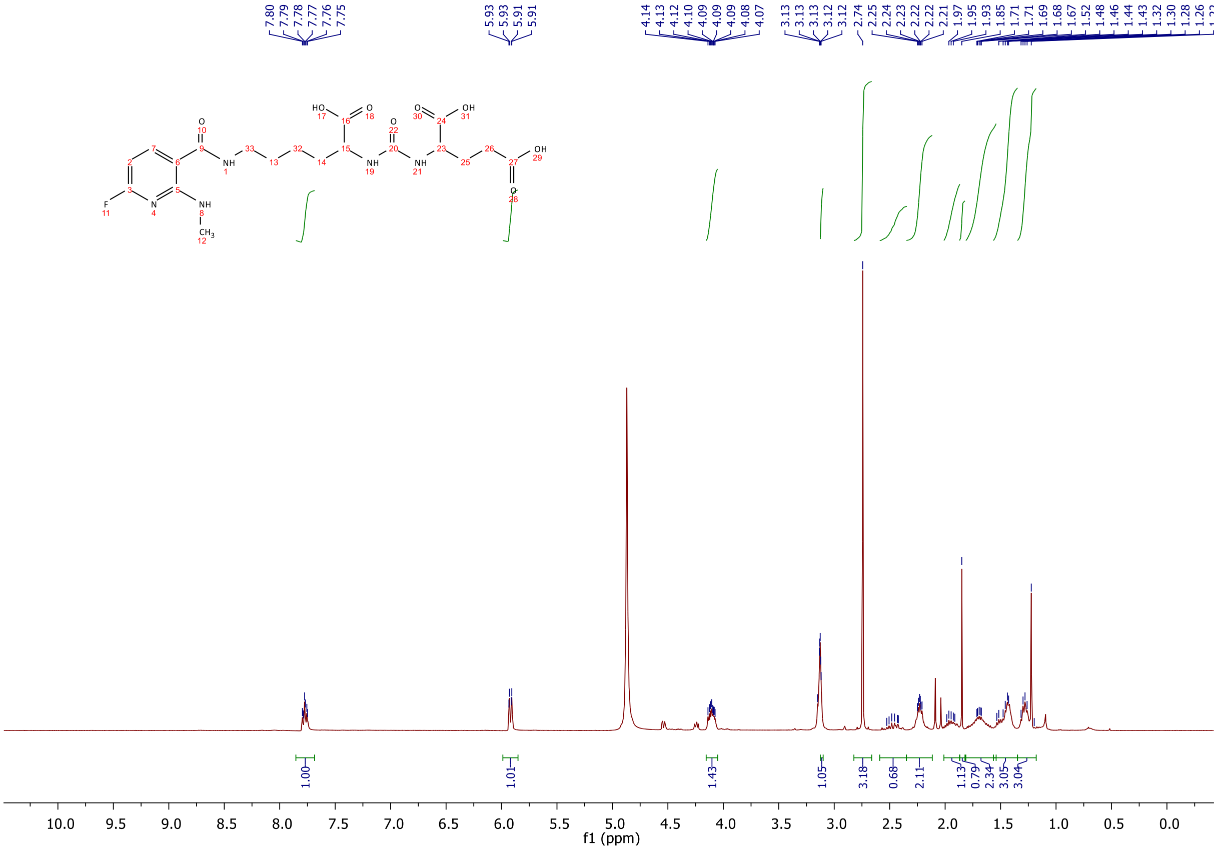

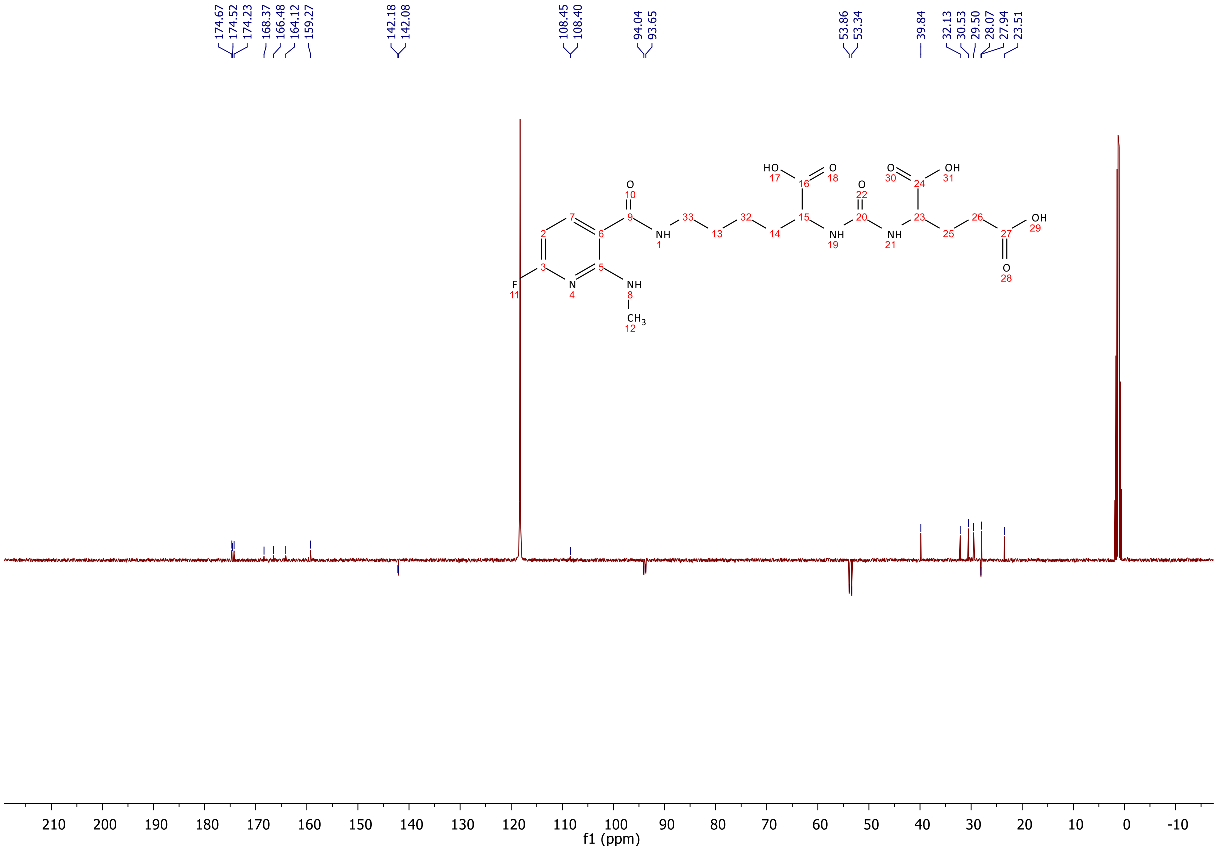

2.37 Compound **6a**

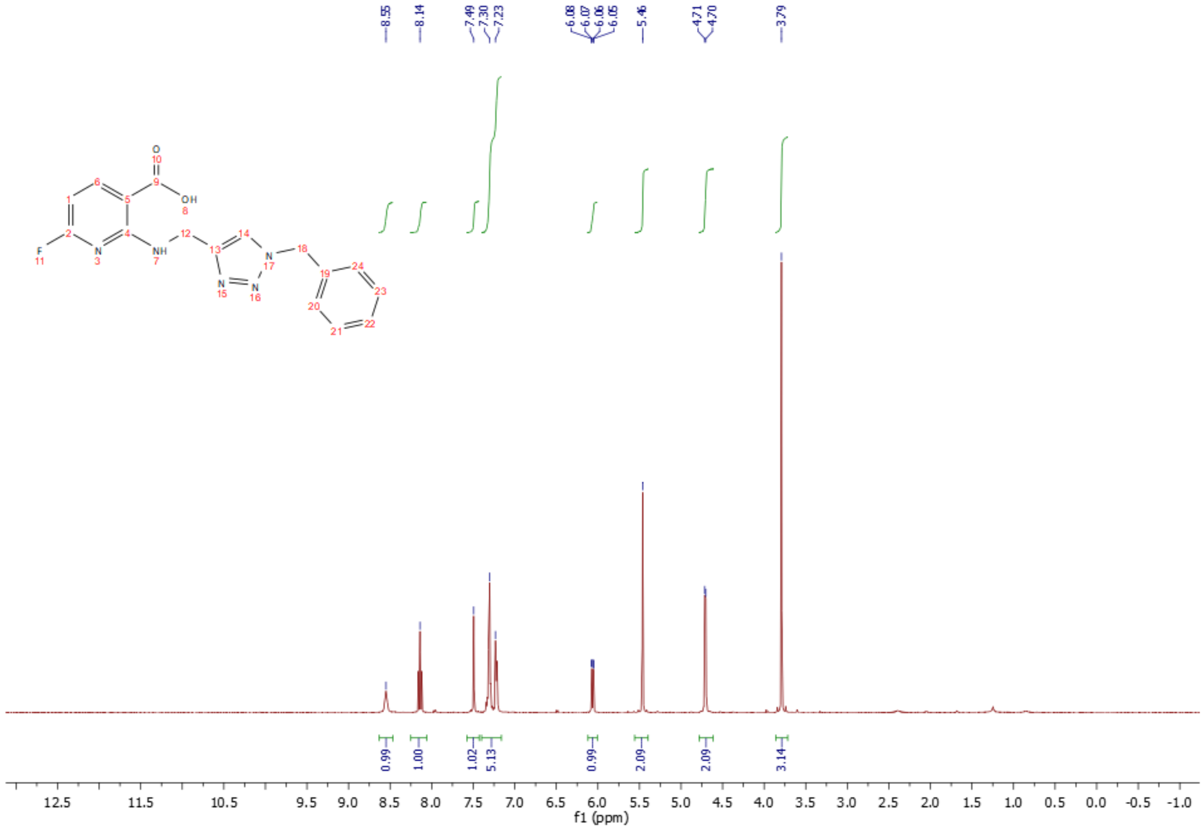

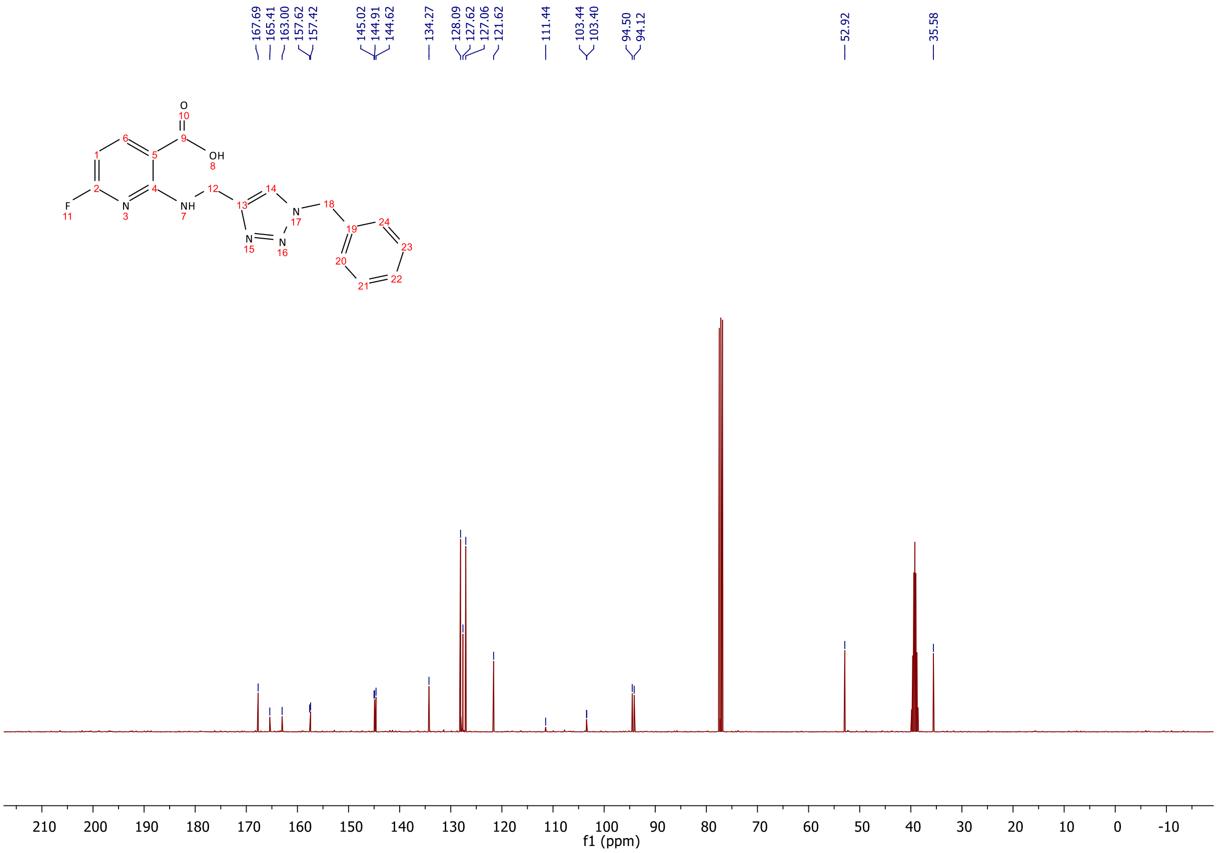

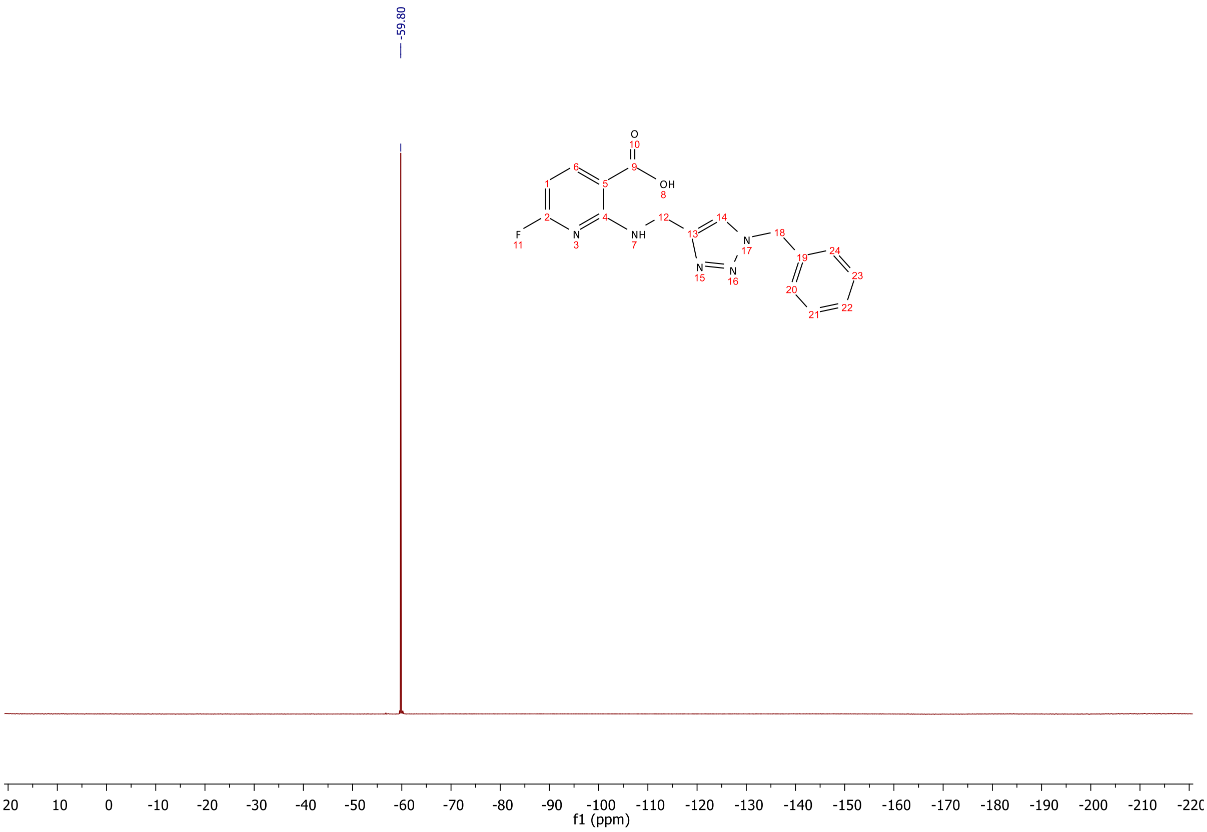
-59.80
O
10
9
OH
8
6
5
1
2
4
F
11
N
3
NH
7
12
13
14
N
17
18
19
24
23
N
15
N
16
20
21
22
20
10
0
-10
-20
-30
-40
-50
-60
-70
-80
-90
-100
-110
-120
-130
-140
-150
-160
-170
-180
-190
-200
-210
-220
f1 (ppm)

2.38 Compound **10**

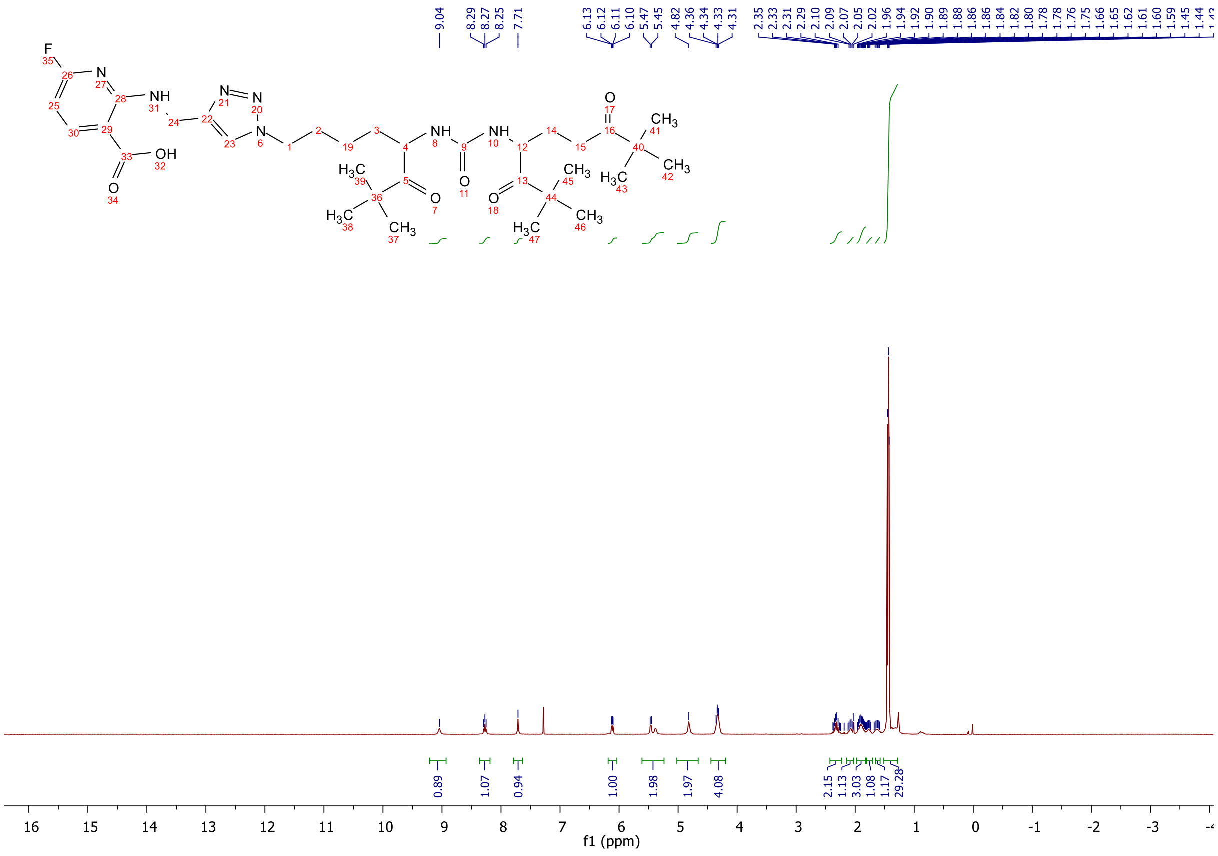

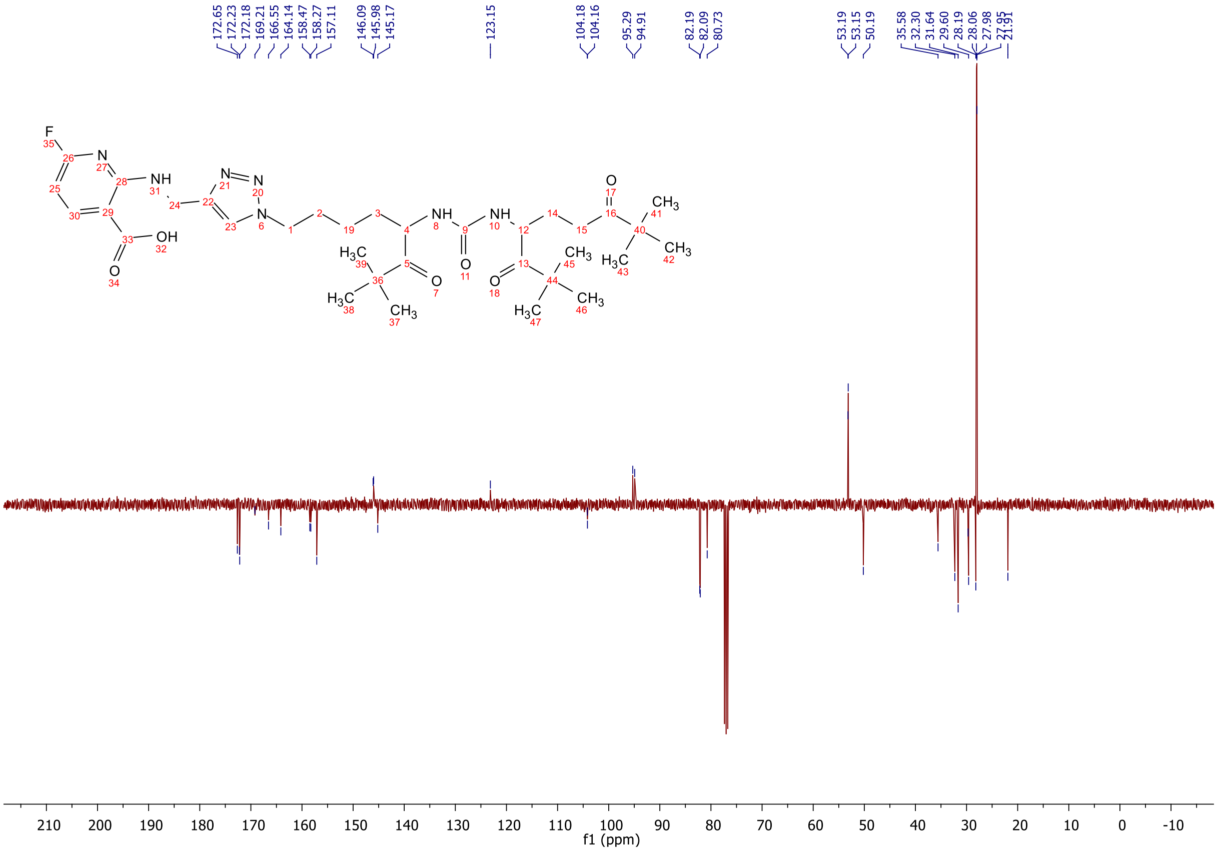

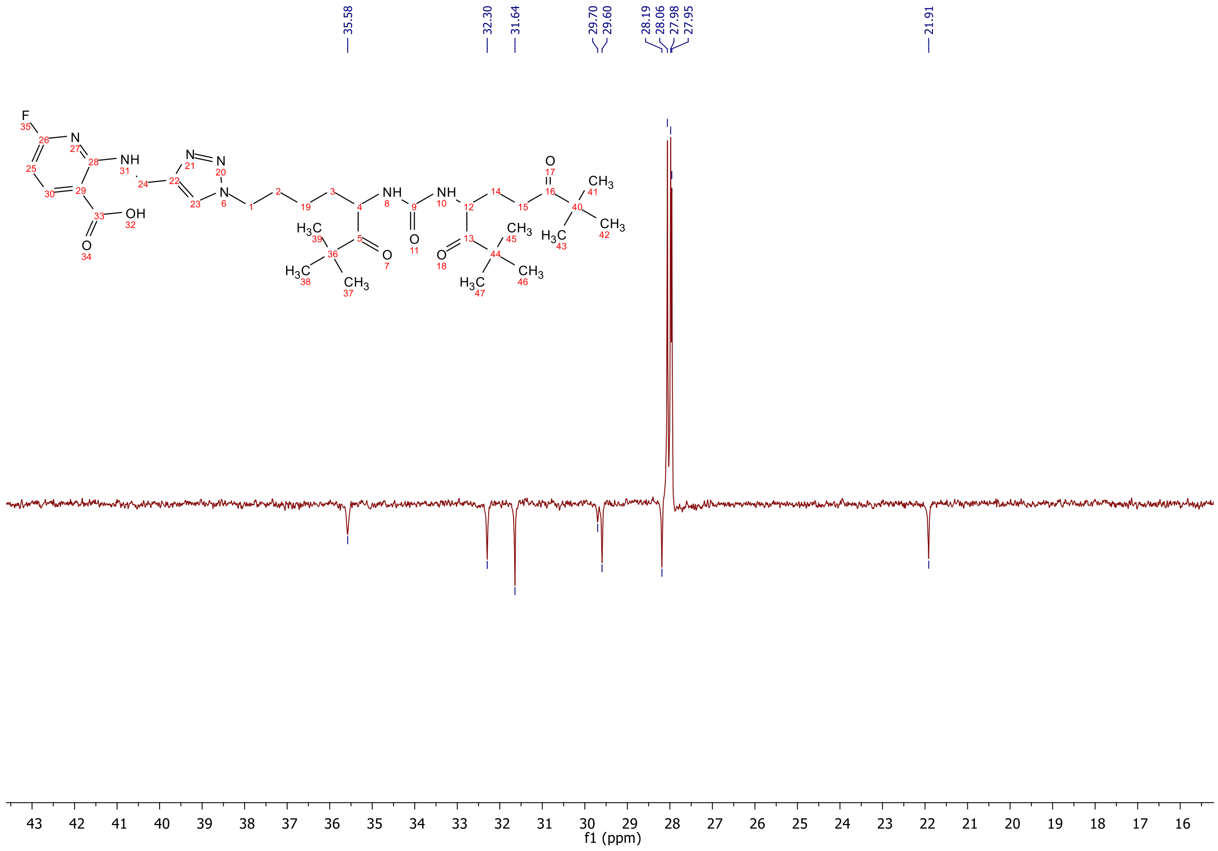
35.58
32.30
31.64
29.70
29.60
28.19
28.06
27.98
27.95
21.91
f1 (ppm)

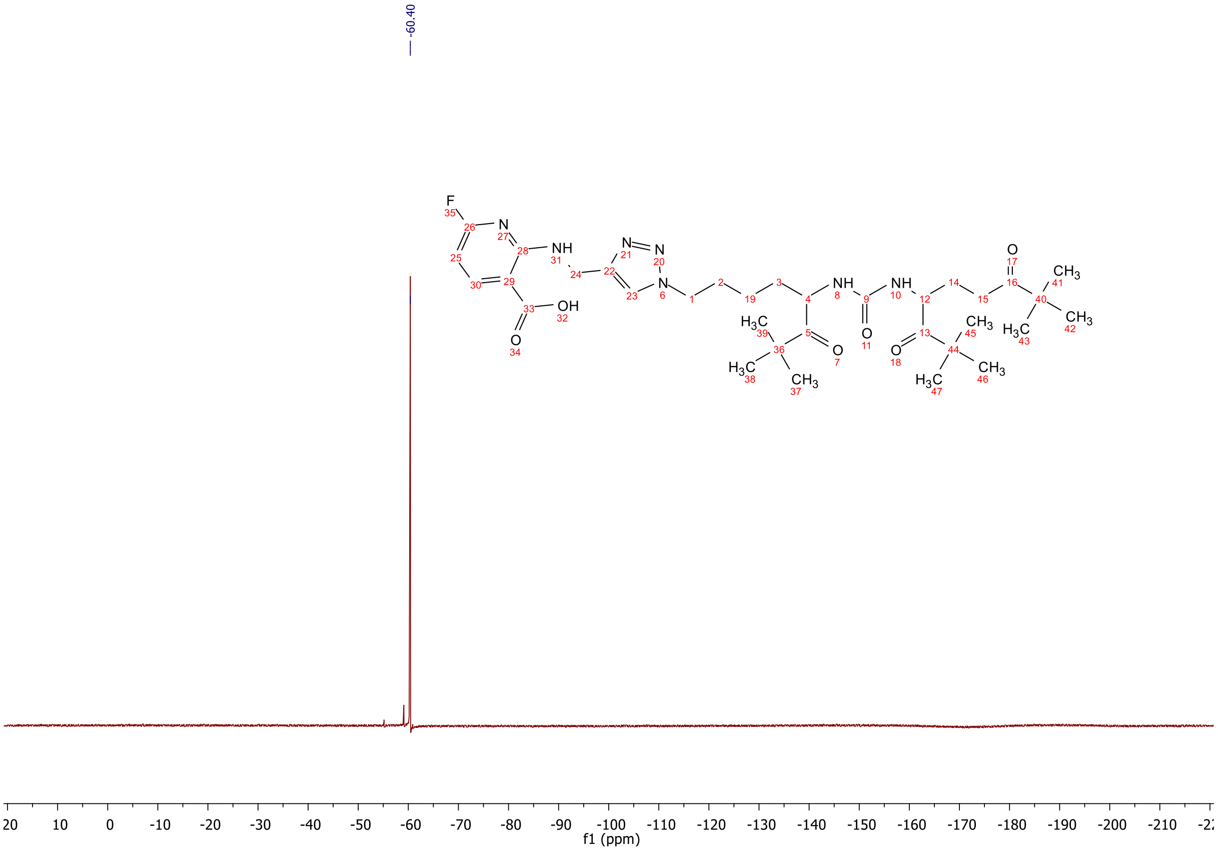
-60.40
f1 (ppm)

2.39 Compound **6b** (JK-PSMA-16)

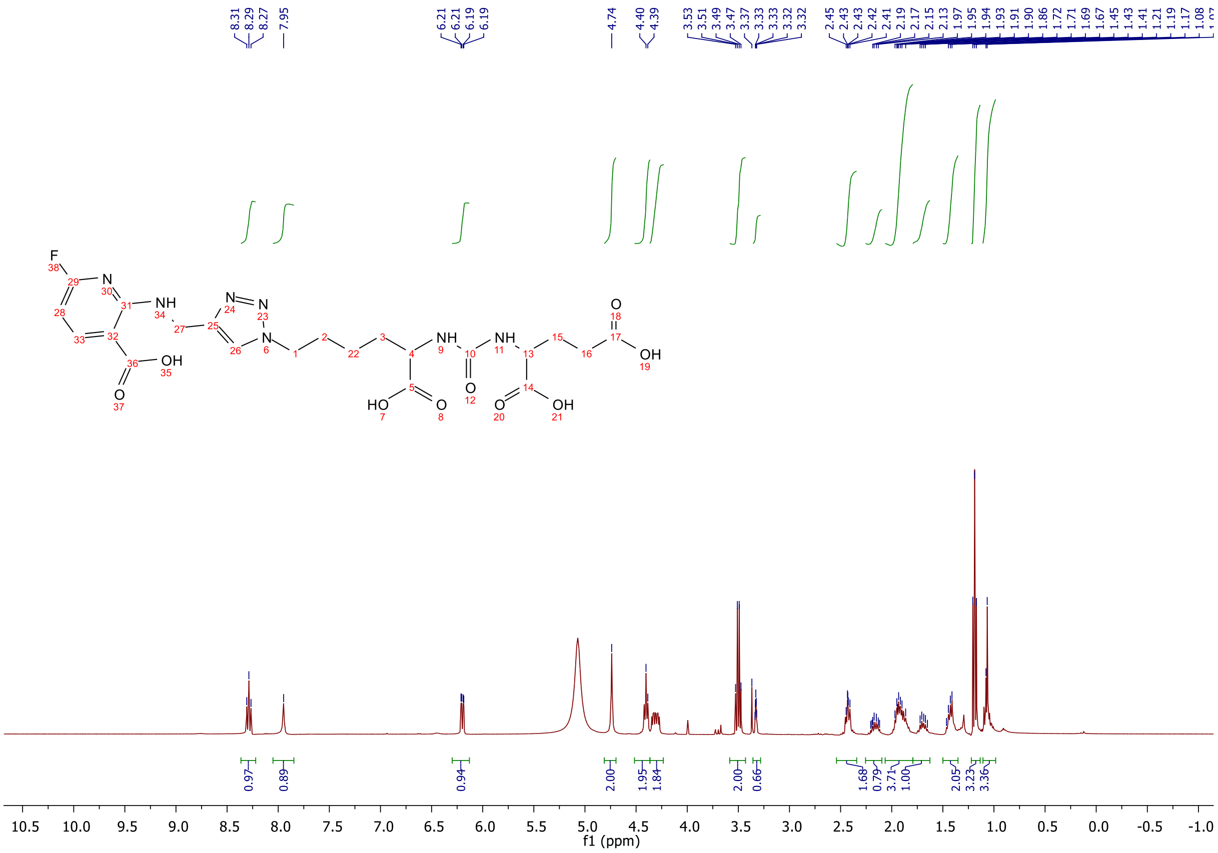

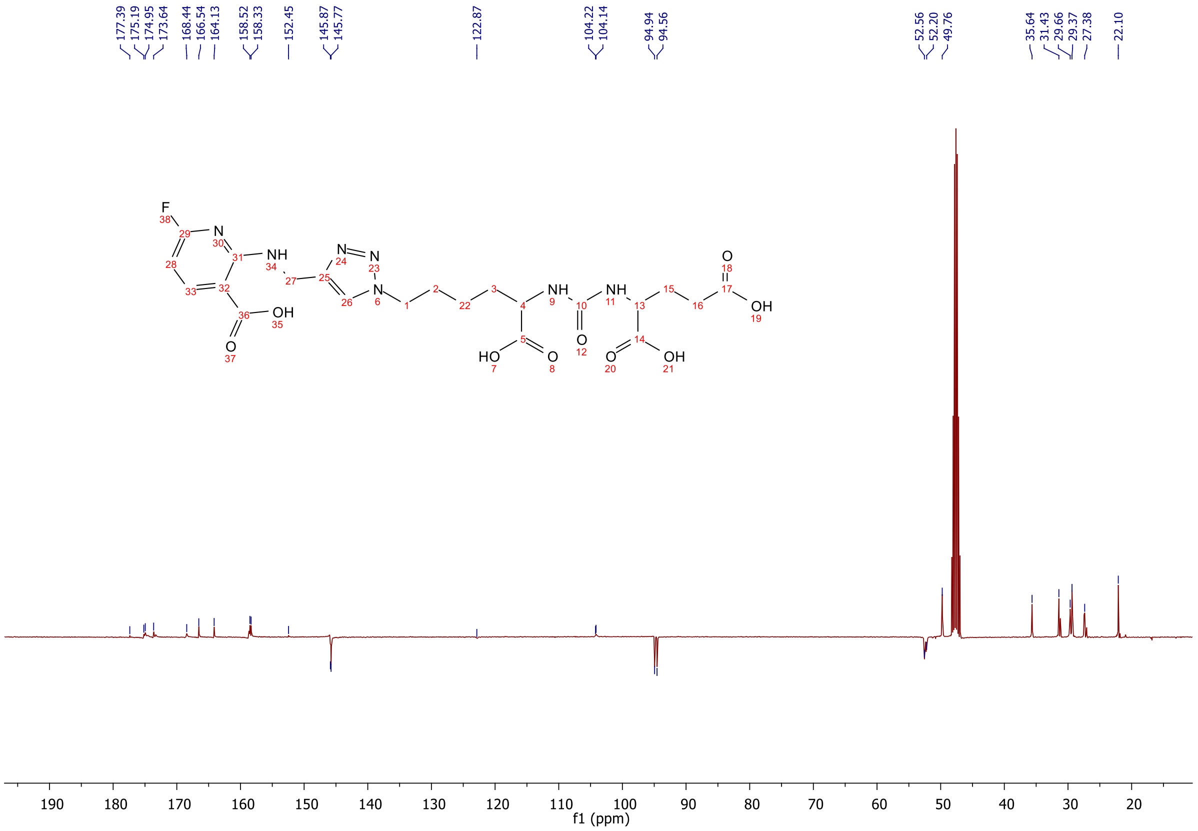

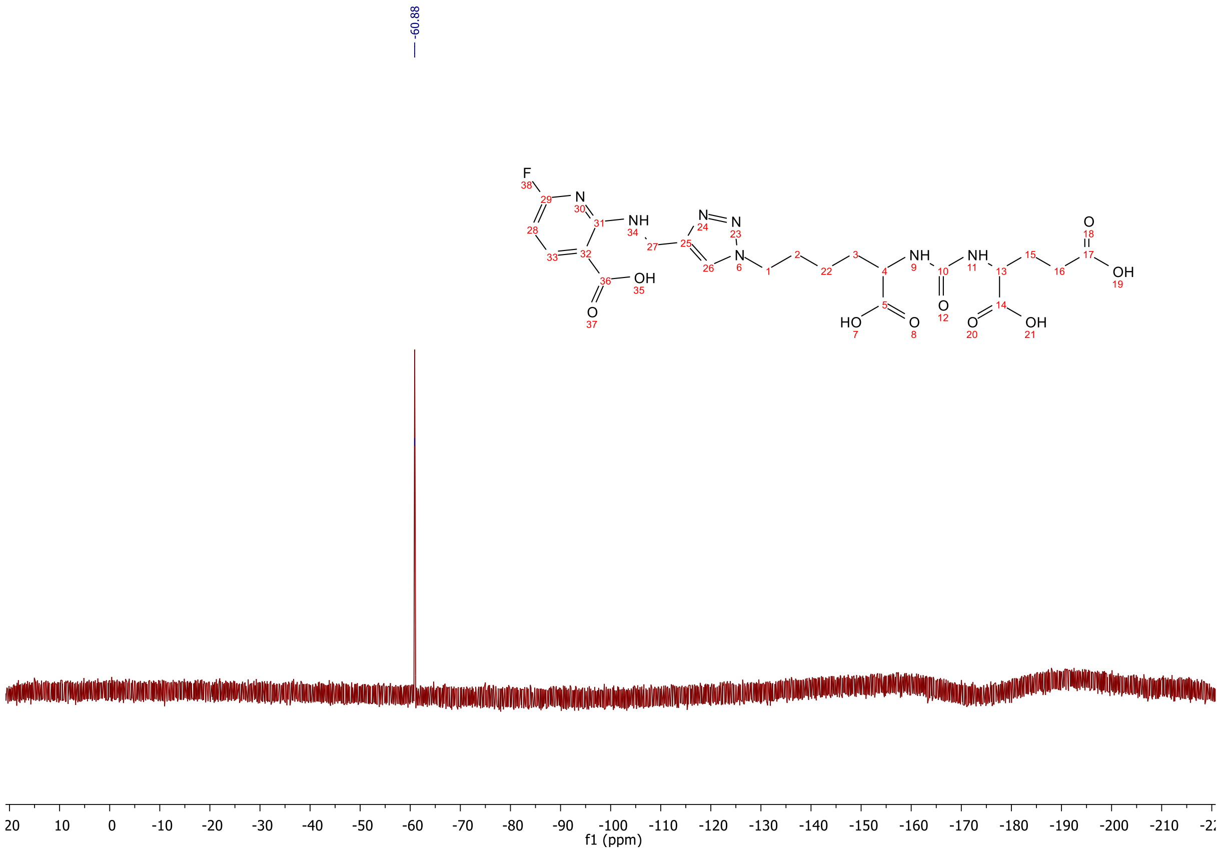
-60.88
20 10 0 -10 -20 -30 -40 -50 -60 -70 -80 -90 -100 -110 -120 -130 -140 -150 -160 -170 -180 -190 -200 -210
f1 (ppm)

2.40 Compound **6c**

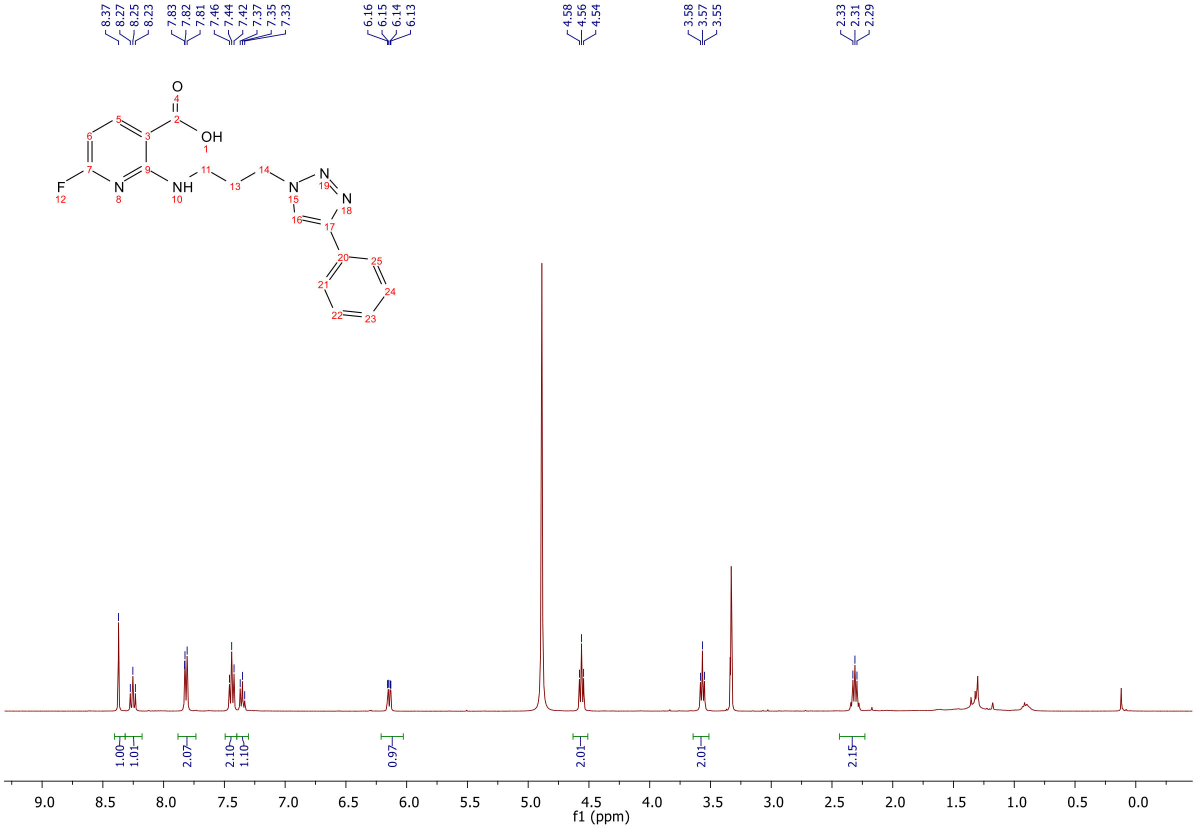

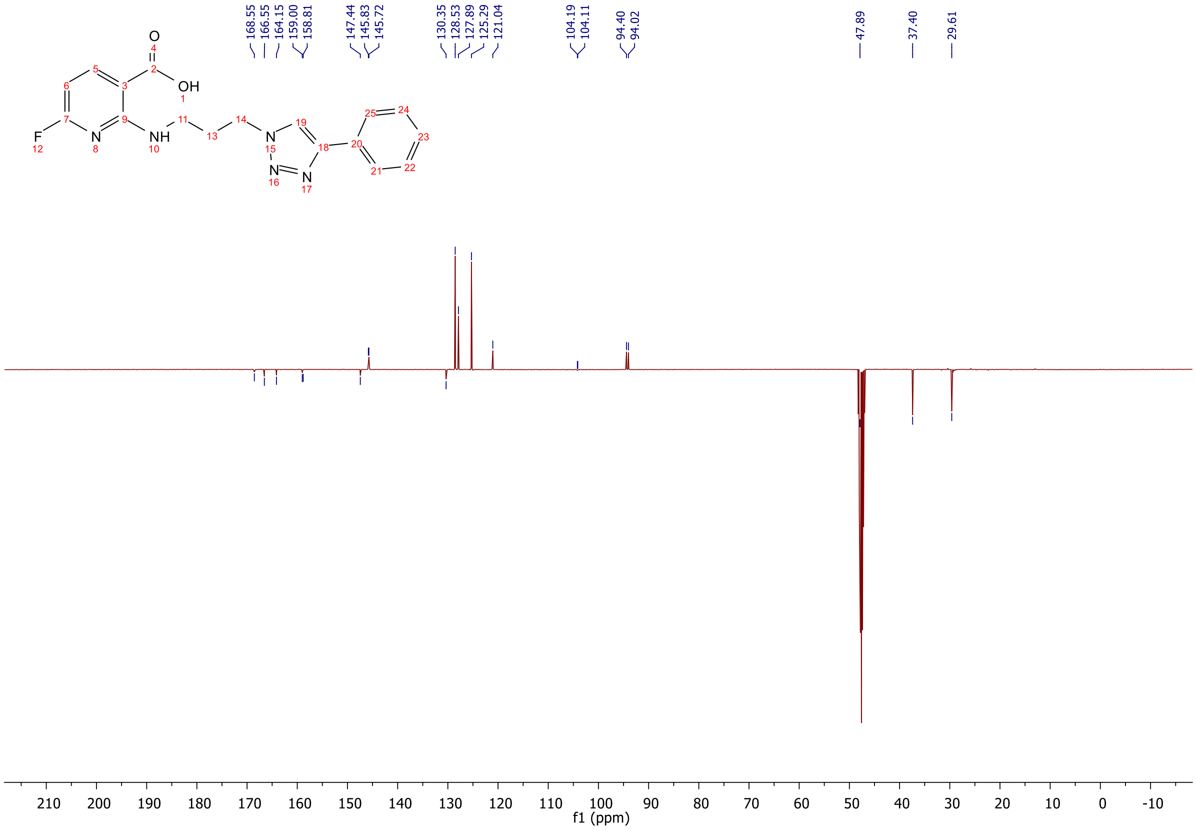

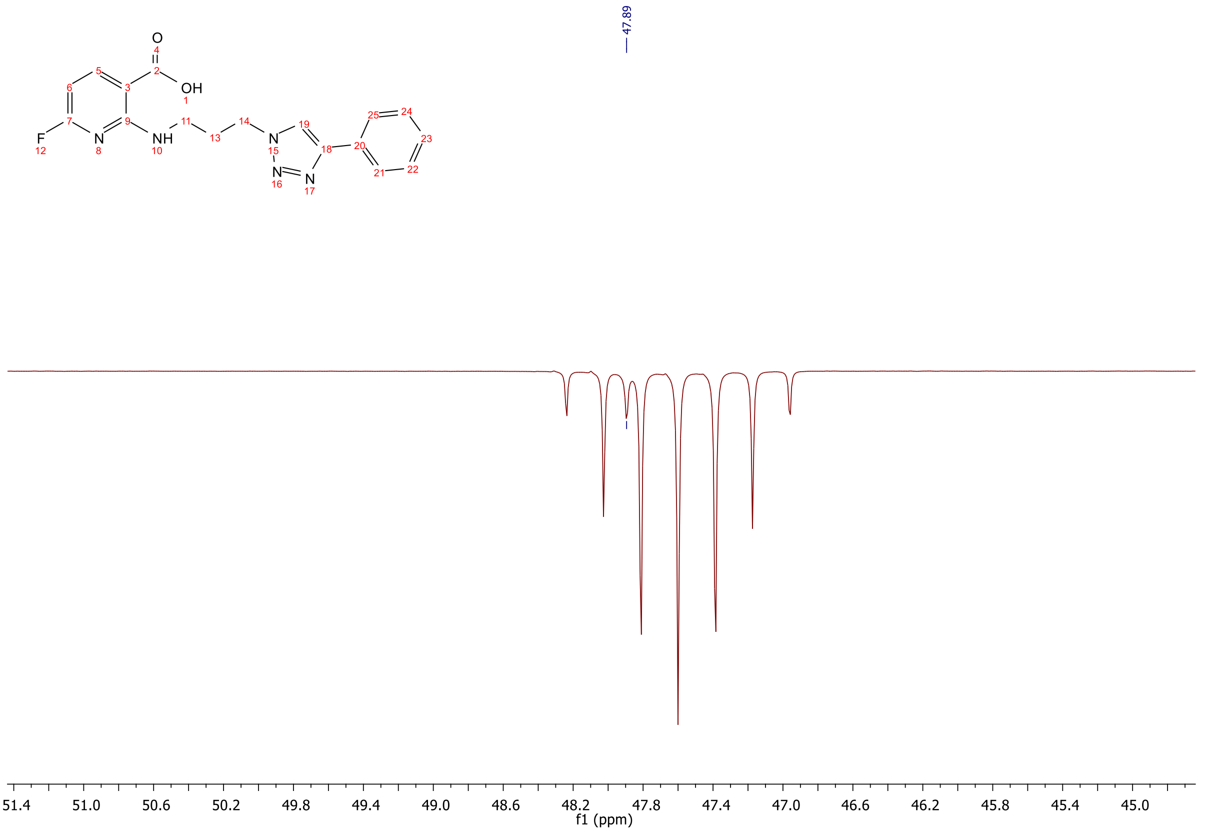
47.89
O
OH
F
N
NH
N
N
N
51.4
51.0
50.6
50.2
49.8
49.4
49.0
48.6
48.2
47.8
47.4
47.0
46.6
46.2
45.8
45.4
45.0
f1 (ppm)

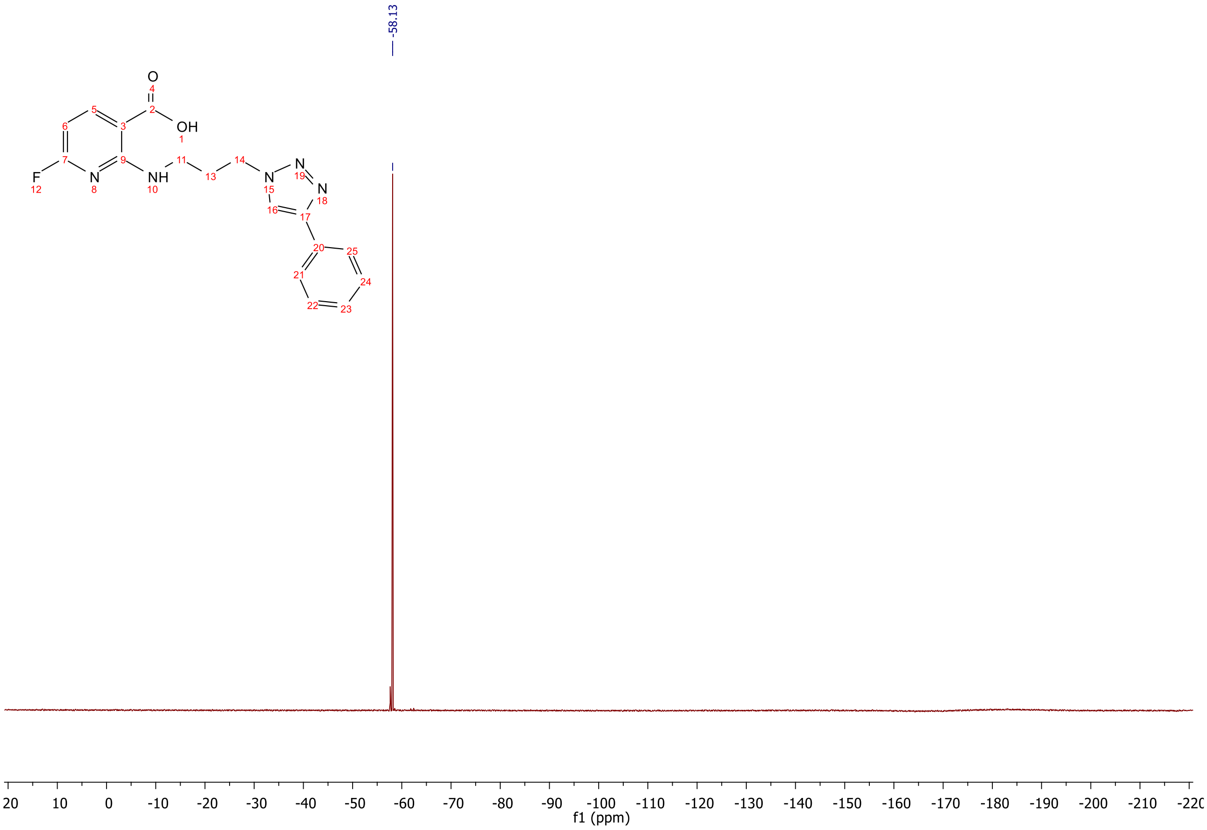
-58.13
O
OH
F
N
NH
N
N
N
20
10
0
-10
-20
-30
-40
-50
-60
-70
-80
-90
-100
-110
-120
-130
-140
-150
-160
-170
-180
-190
-200
-210
-220
f1 (ppm)

2.41 Compound **11**

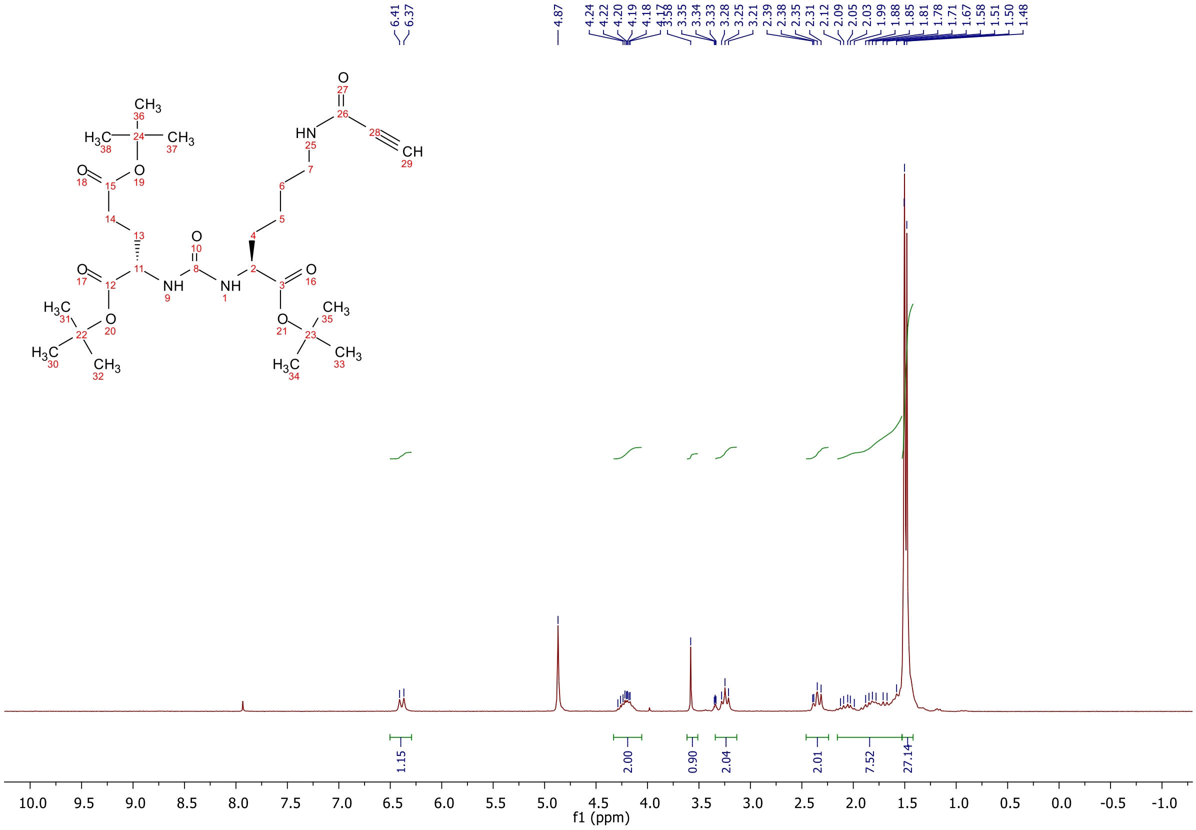

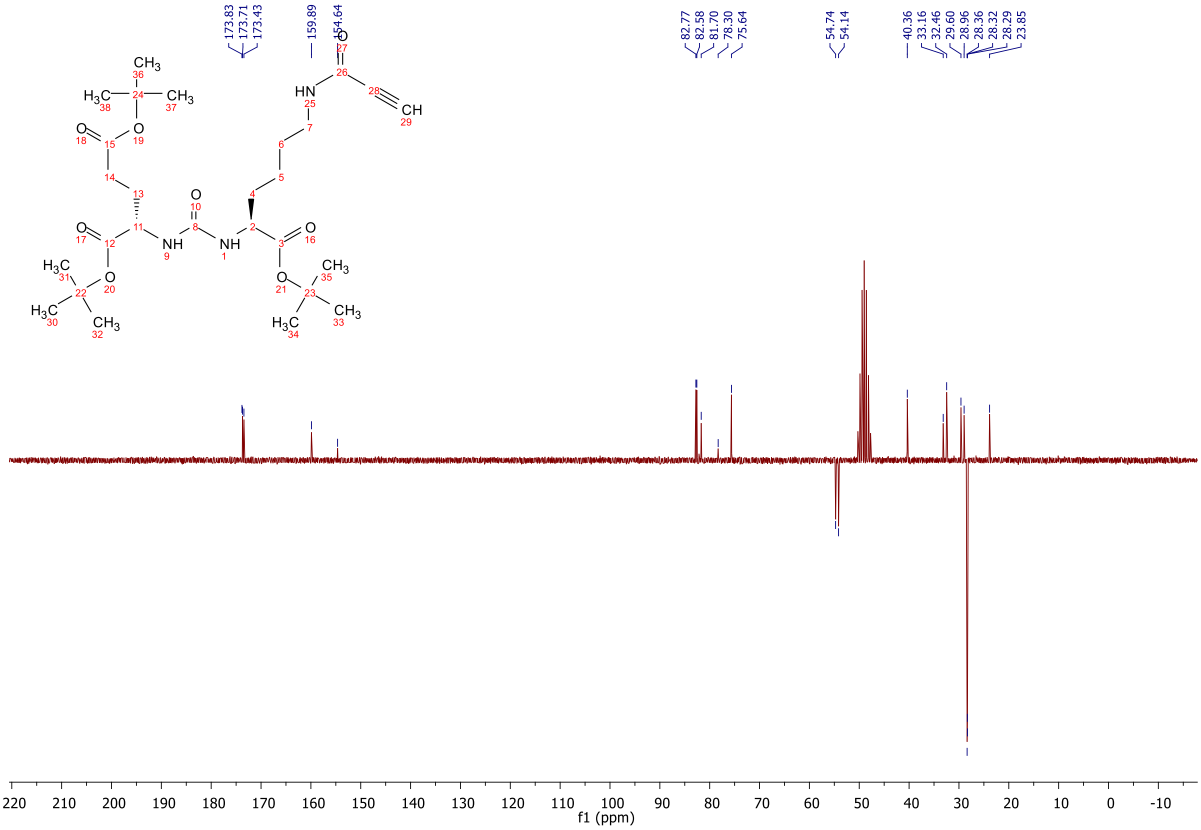

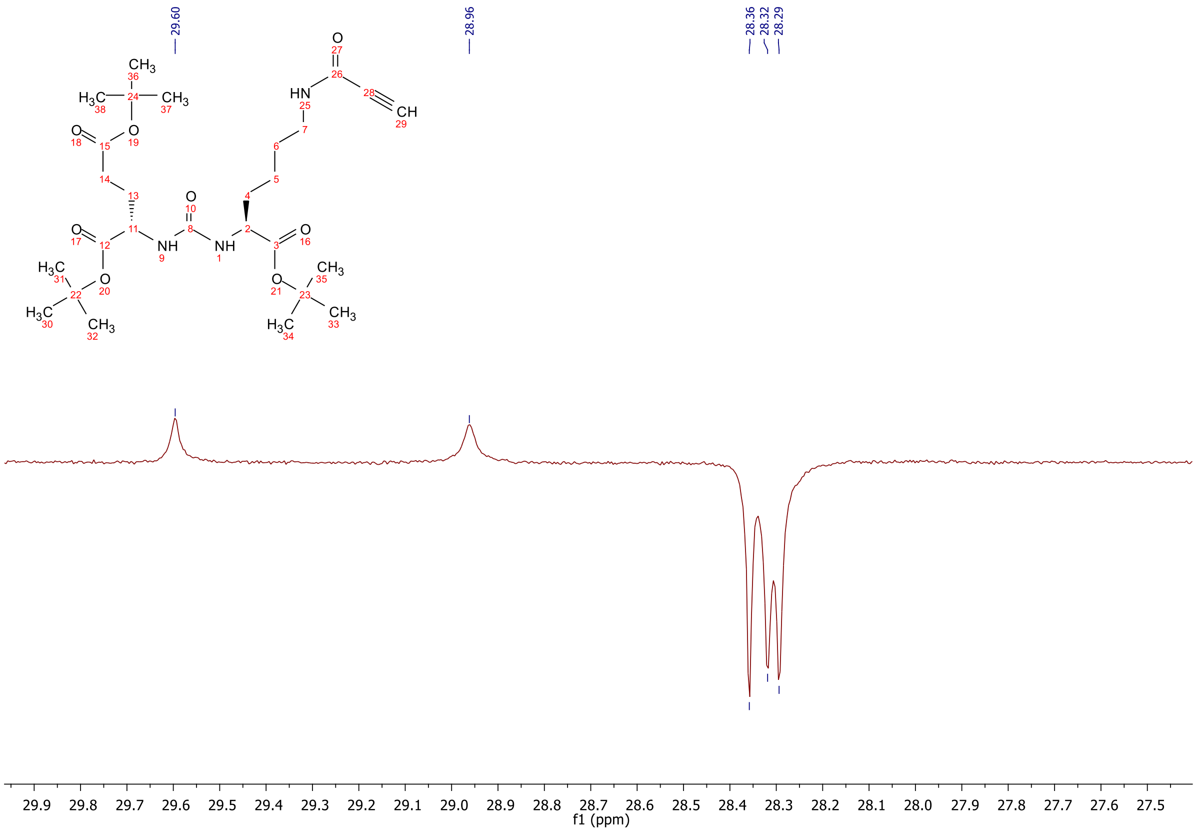
29.60
28.96
28.36
28.32
28.29
29.9 29.8 29.7 29.6 29.5 29.4 29.3 29.2 29.1 29.0 28.9 28.8 28.7 28.6 28.5 28.4 28.3 28.2 28.1 28.0 27.9 27.8 27.7 27.6 27.5
f1 (ppm)

2.42 Compound **8**

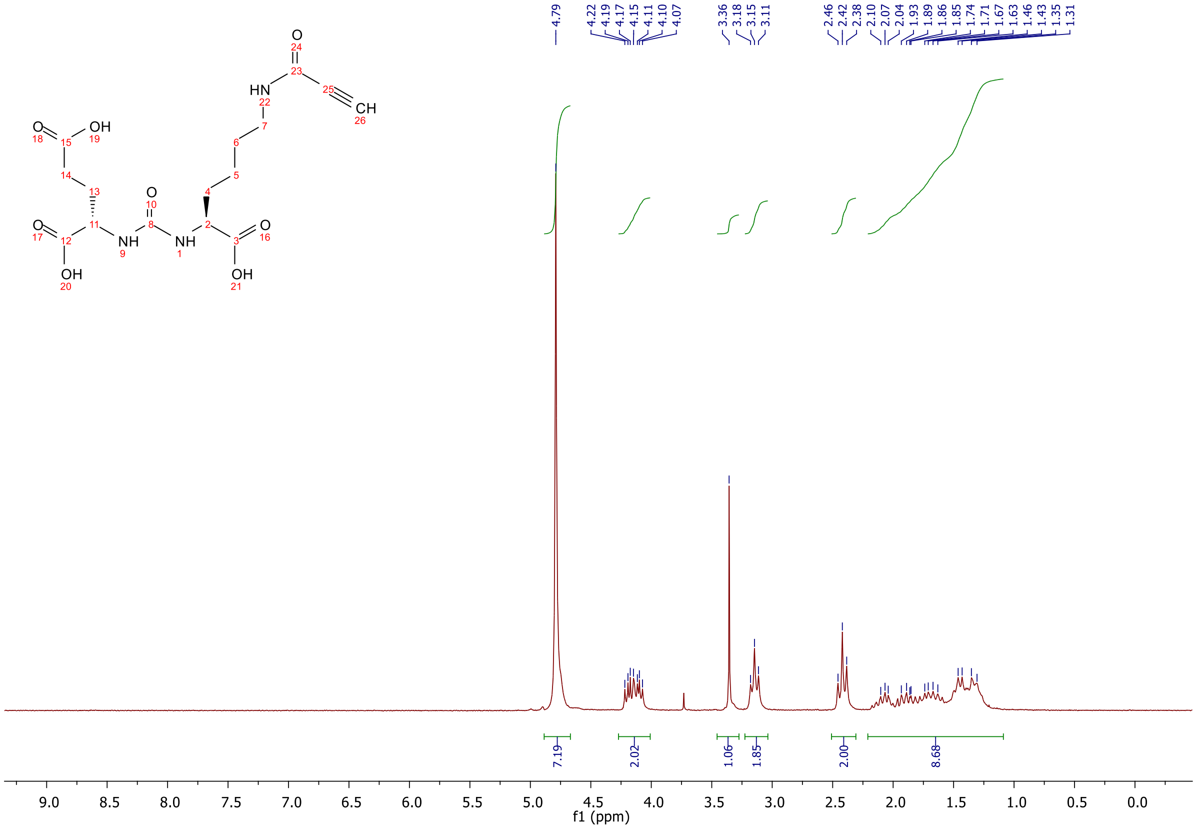

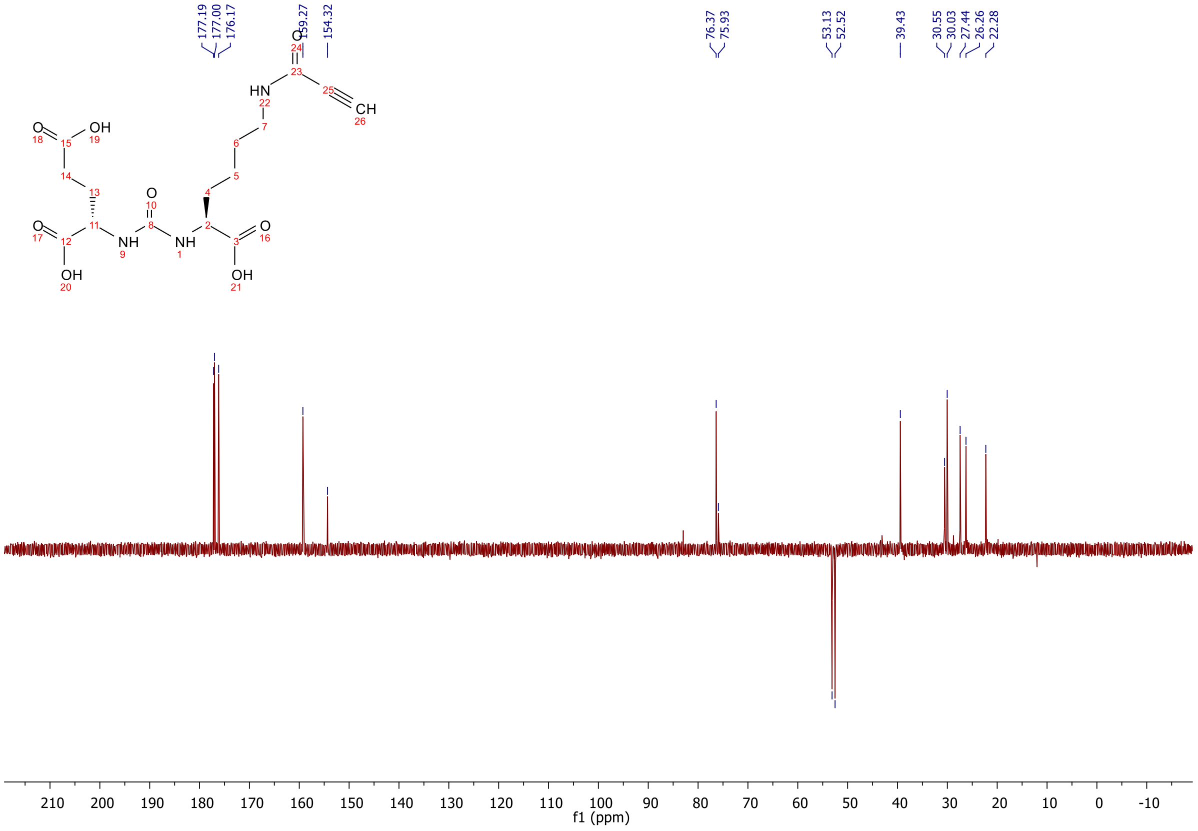

2.43 Compound **6d** (JK-PSMA-18)

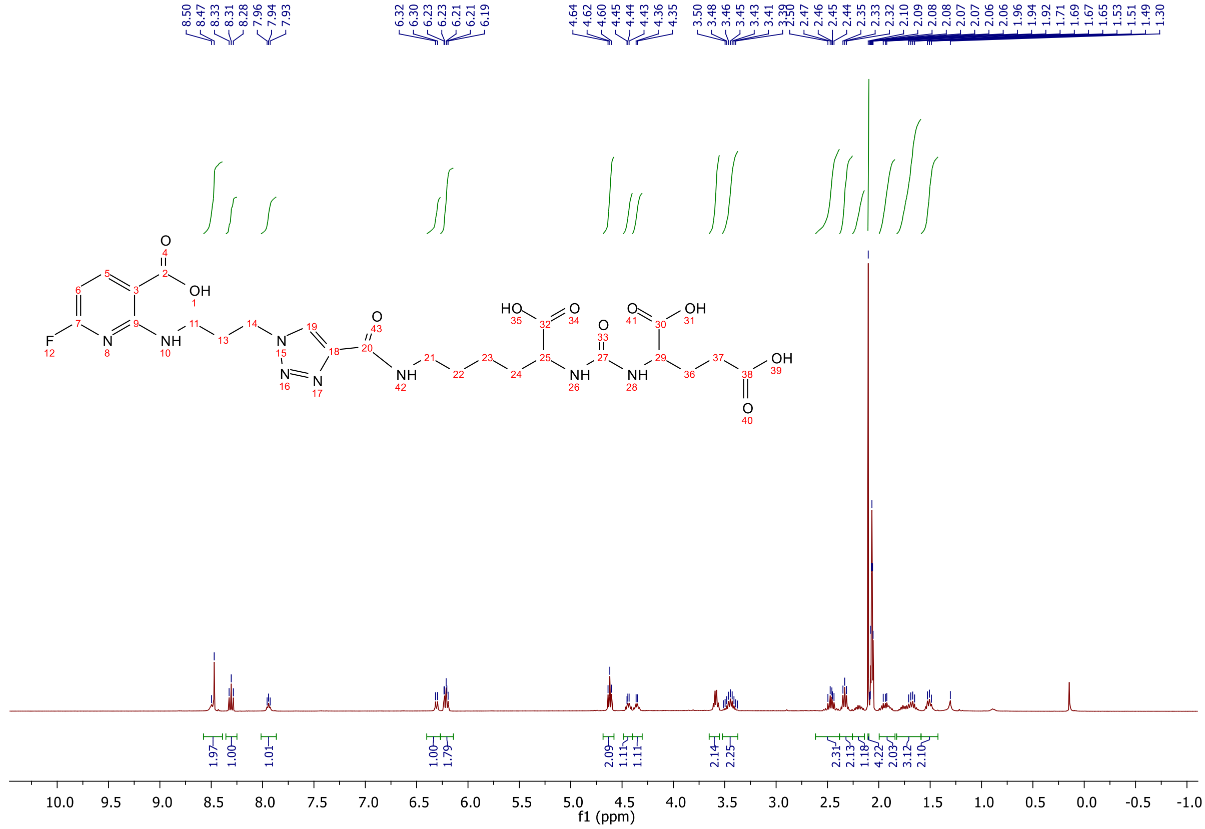

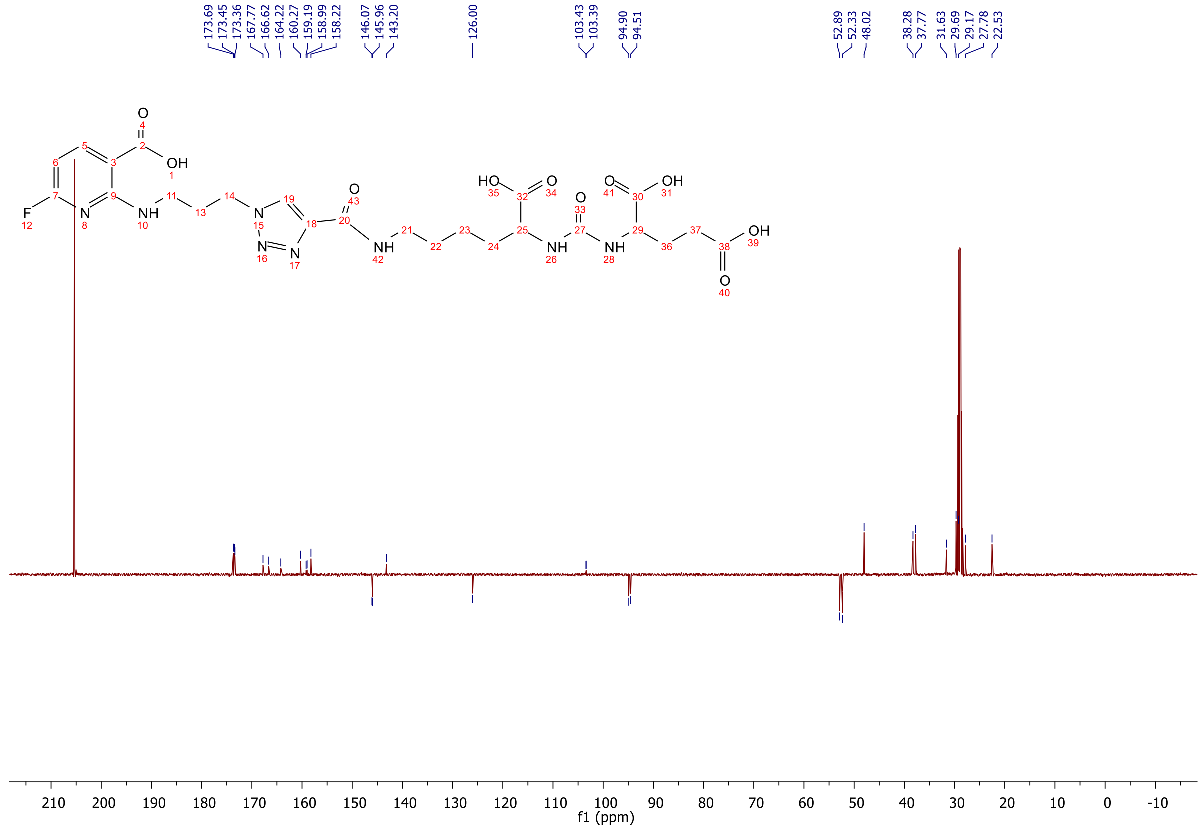

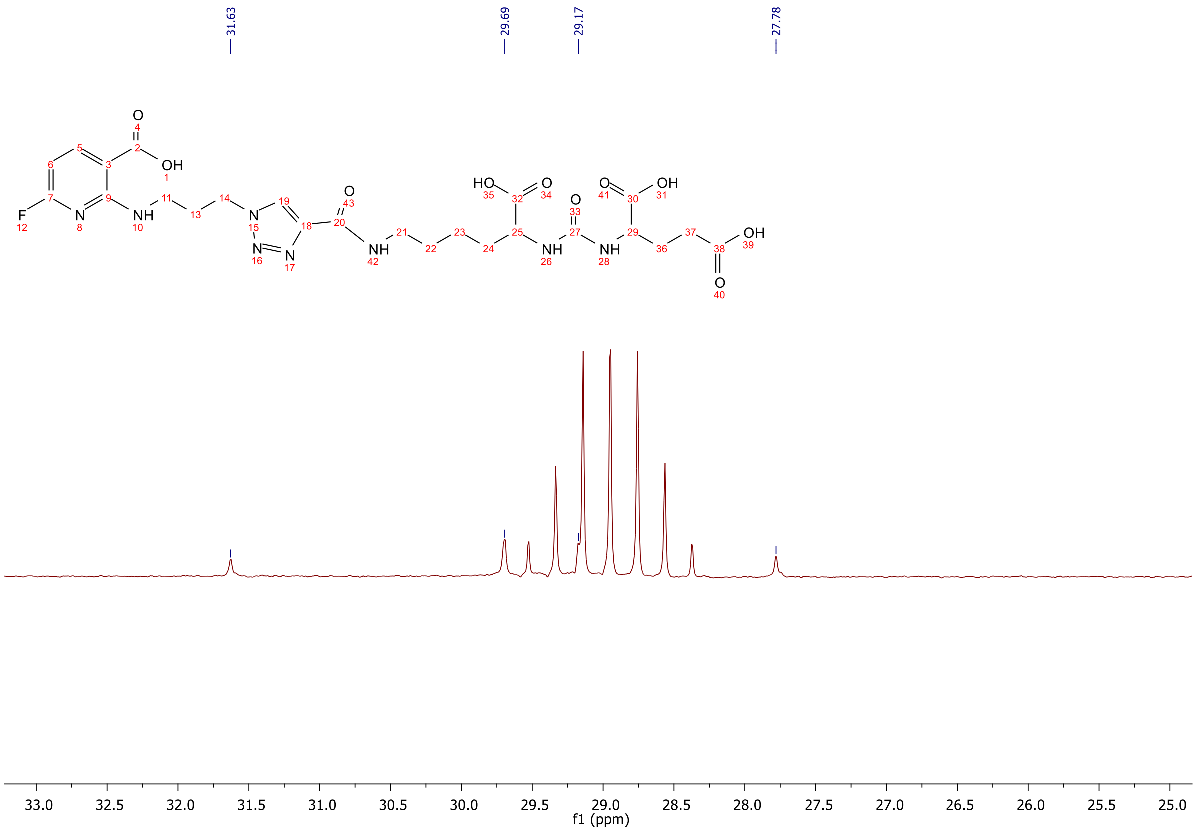
31.63
29.69
29.17
27.78
f1 (ppm)

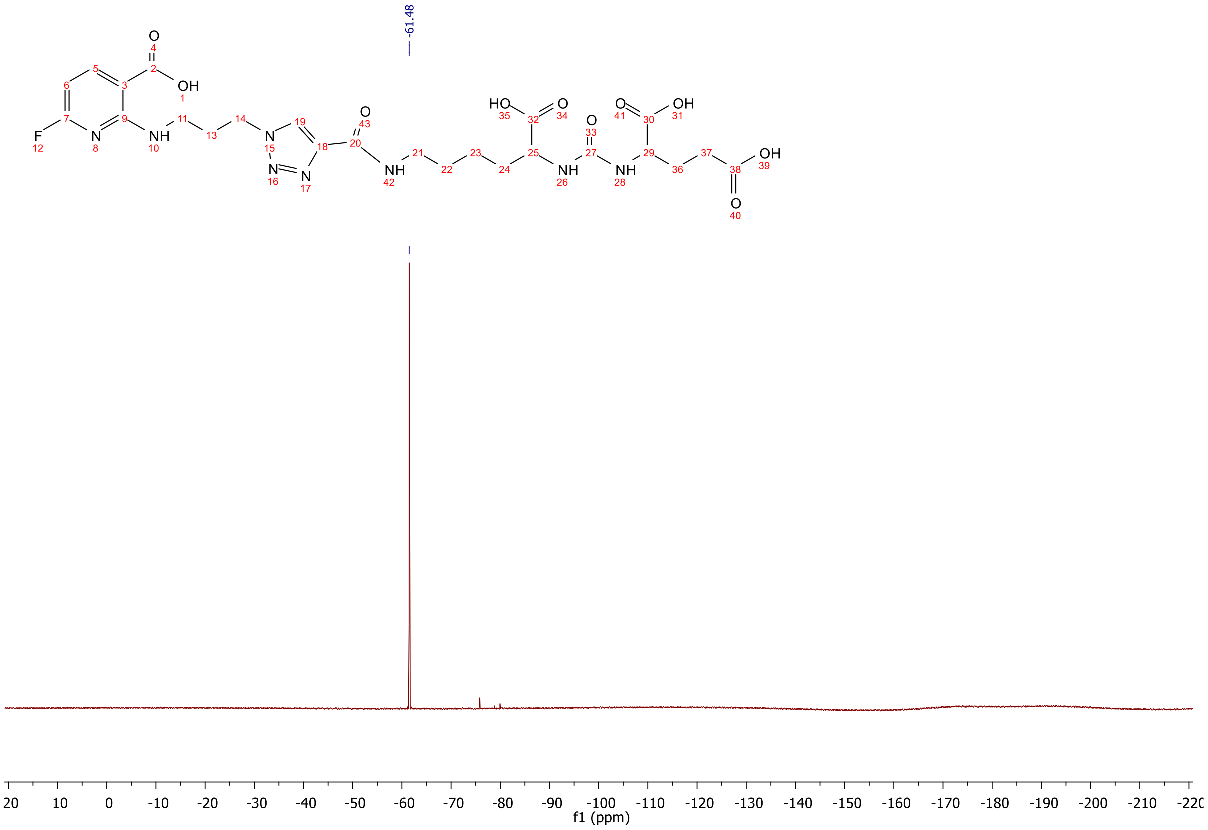
-61.48
f1 (ppm)

3 General Methods for Analytical HPLC:

Column: Multokrom 250×4.6 mm 100-5 C18 AQ column (CS Chromatographie Service GmbH, Langerwehe, Germany)

Method A: 0–10 min 50% MeCN, 11–15 min 90% MeCN, flow rate 1 mL/min.

Method B: 0–20 min 50% MeCN, 21–25 min 90 % MeCN, flow rate 1.5 mL/min.

Method C: 0–20 min 50% MeCN, 21–25 min 90 % MeCN, flow rate 1 mL/min.

Method D: 0–5 min 50% MeCN, 7–12.5 min 70% MeCN, flow rate 1 mL/min.

Method E: 0–15 min 20% to 90% MeCN, 15–20 min 90% MeCN, flow rate 1 mL/min.

Method F: 0–12 min 30% MeCN, 15–20 min 80% MeCN, flow rate 1 mL/min.

4 HPLC chromatograms of ^{18}F-labeled AFAs:

4.1 HPLC traces of [^{18}F]**3e** (**Figs. S1** & **S2**)

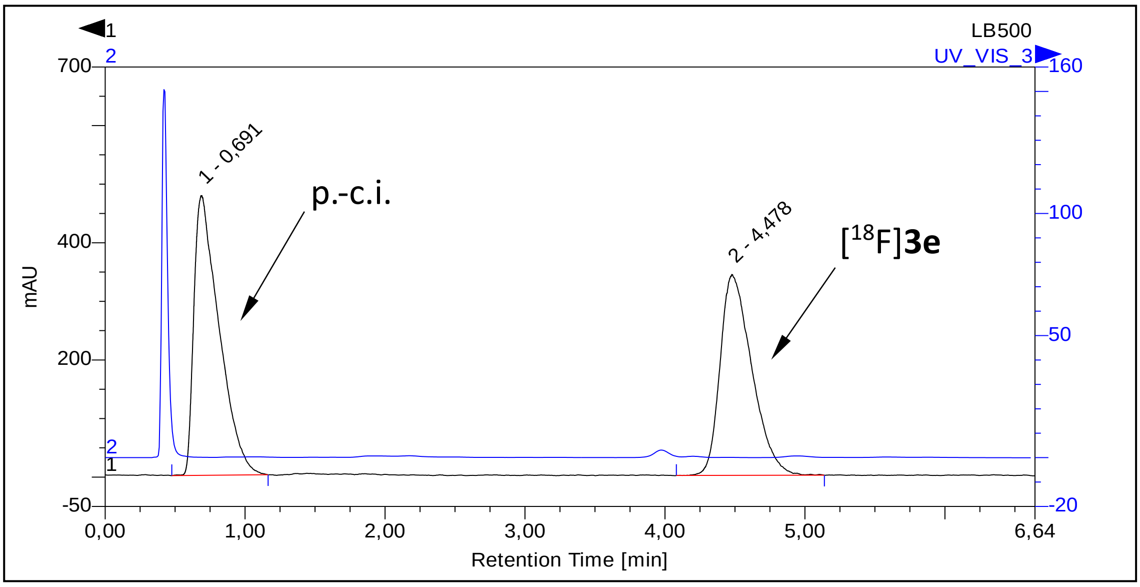

Figure S1: HPLC traces of SPE-purified [^{18}F]**3e**, Method C (0.1% TFA). Blue trace: UV, λ = 254 nm; black trace: radioactivity. Abbreviation: p.-c.i. – post-column injection.

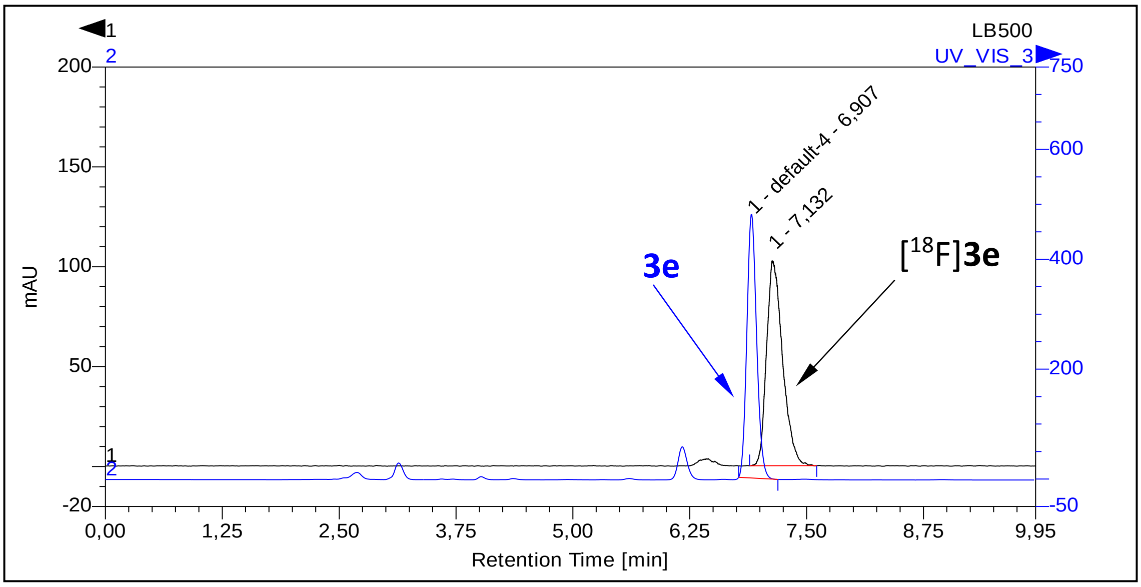

Figure S2: HPLC traces of [^{18}F]**3e** spiked with the non-radioactive reference compound, Method A (0.1% TFA). Blue trace: UV, λ = 254 nm; black trace: radioactivity.

4.2 HPLC traces of [^{18}F]**3f** (**Figs. S3** & **S4**)

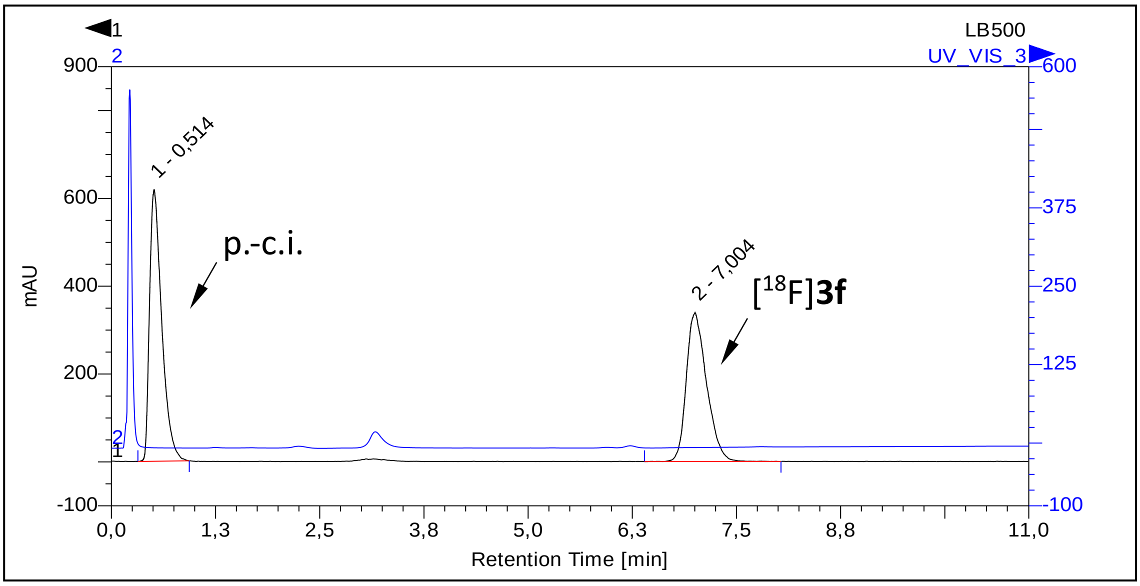

Figure S3: HPLC traces of [^{18}F]**3f**, Method C (0.1% TFA). Blue trace: UV, λ = 254 nm; black trace: radioactivity. Abbreviation: p.-c.i. – post-column injection.

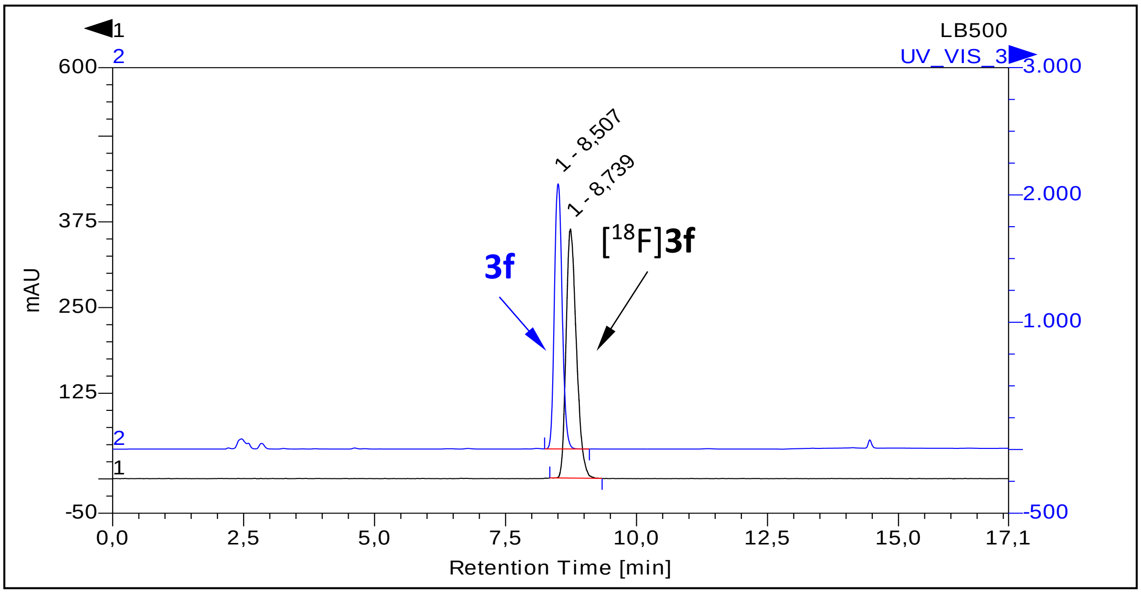

Figure S4: HPLC traces of [^{18}F]**3f** spiked with the non-radioactive reference compound, Method A (0.1% TFA). Blue trace: UV, λ = 254 nm; black trace: radioactivity.

4.3 HPLC traces of [18F]**3g** (**Figs. S5** & **S6**)

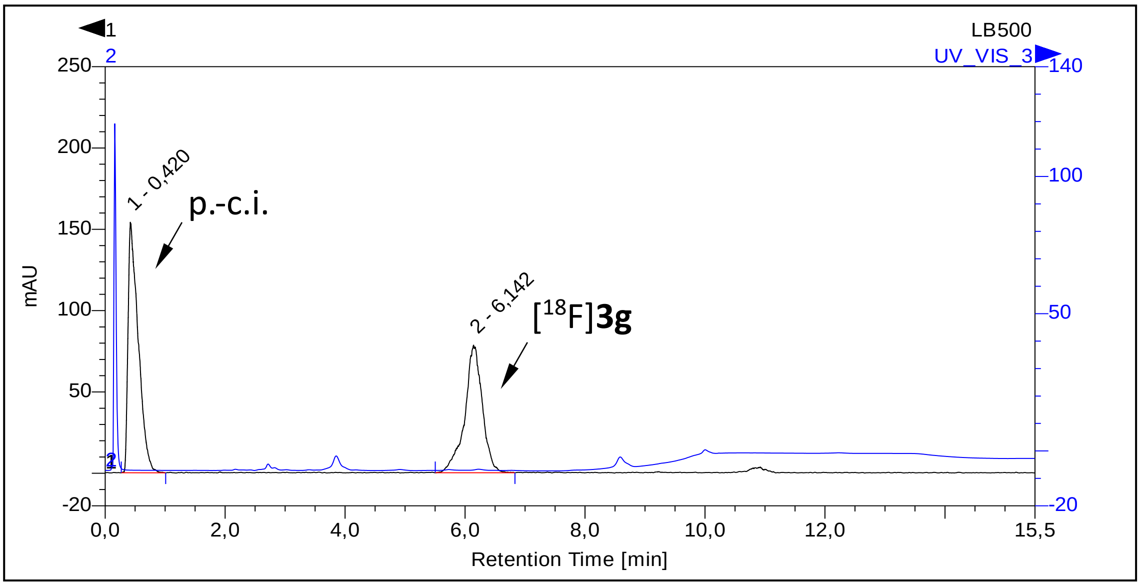

Figure S5: HPLC traces of [18F]**3g**, Method D (0.1% TFA). Blue trace: UV, λ = 254 nm; black trace: radioactivity. Abbreviation: p.-c.i. – post-column injection.

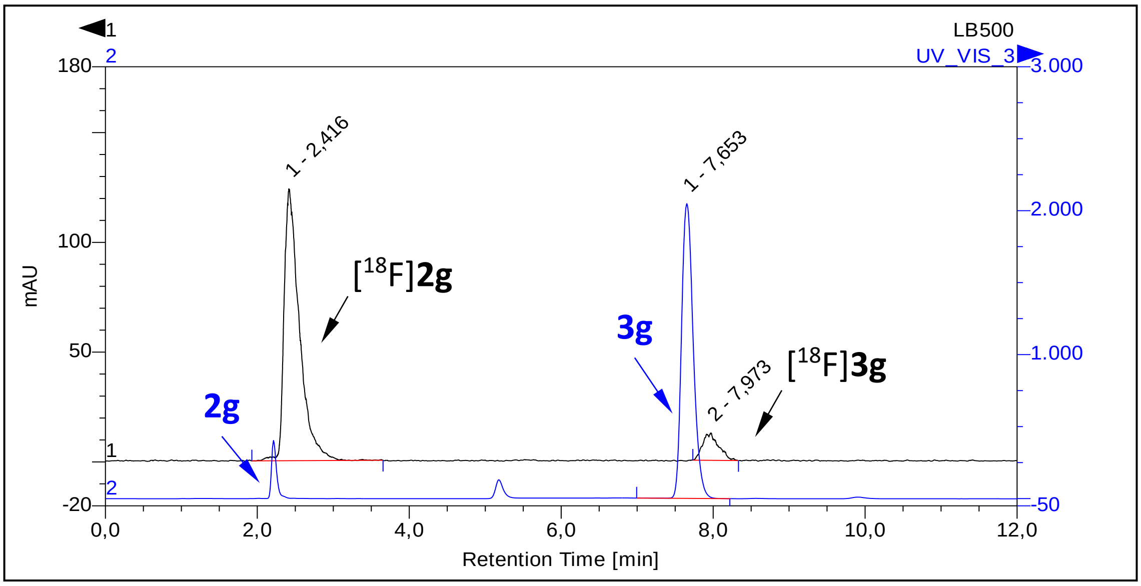

Figure S6: HPLC traces of [18F]**3g** spiked with the non-radioactive reference compounds, Method A (with 300 mg/L NaOAc added to the eluent). Chromatogram shows almost complete (>90%) hydrolysis of the anhydride [18F]**3g** to the acid [18F]**2g** (R_t = 2.4 min). Blue trace: UV, λ = 254 nm; black trace: radioactivity.

4.4 HPLC traces of [^{18}F]**3h** (**Figs. S7** & **S8**)

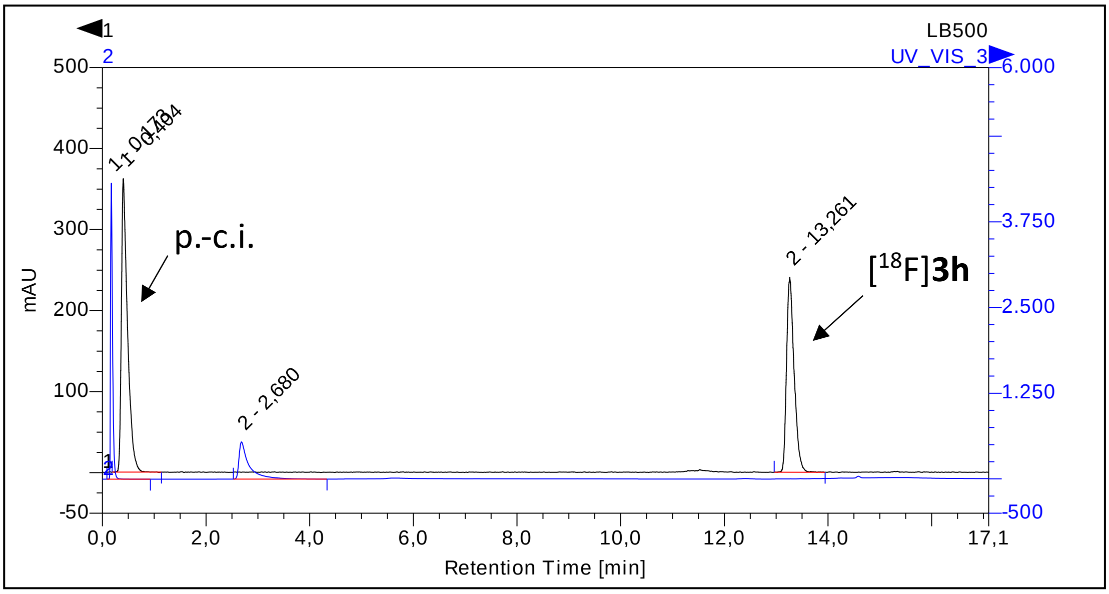

Figure S7: HPLC traces of [^{18}F]**3h**, Method A (0.1% TFA). Blue trace: UV, λ = 254 nm; black trace: radioactivity. Abbreviation: p.-c.i. – post-column injection.

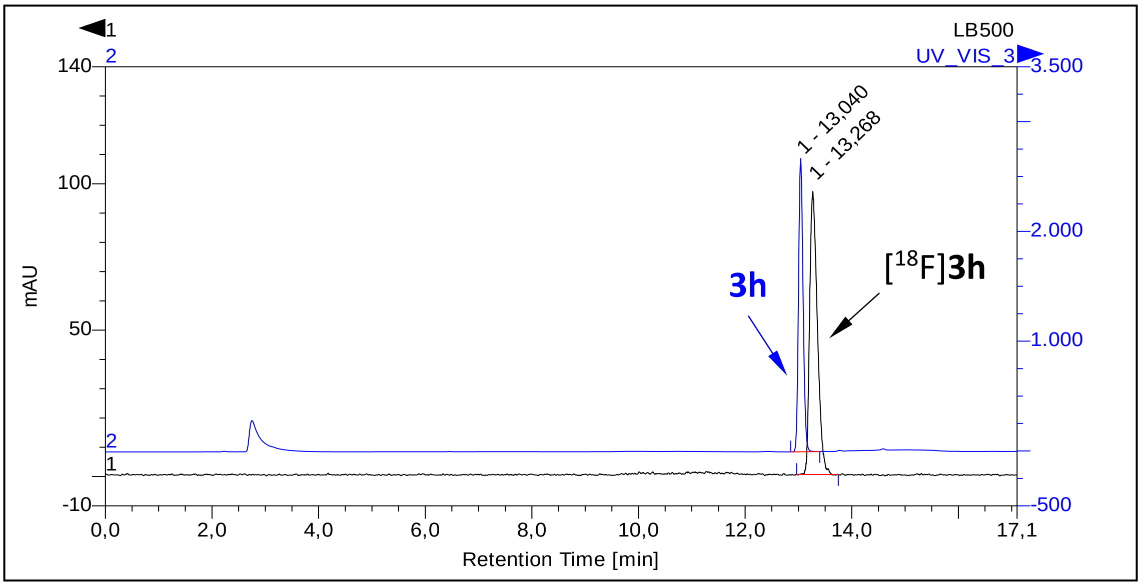

Figure S8: HPLC traces of [^{18}F]**3h** spiked with the non-radioactive reference compound, Method A (with 300 mg/L NaOAc added to the eluent). Blue trace: UV, λ = 254 nm; black trace: radioactivity.

5 HPLC chromatograms of radiolabeled model compounds und PET-tracers:

5.1 HPLC traces of [^{18}F]**5a** (**Figs. S9** & **S10**)

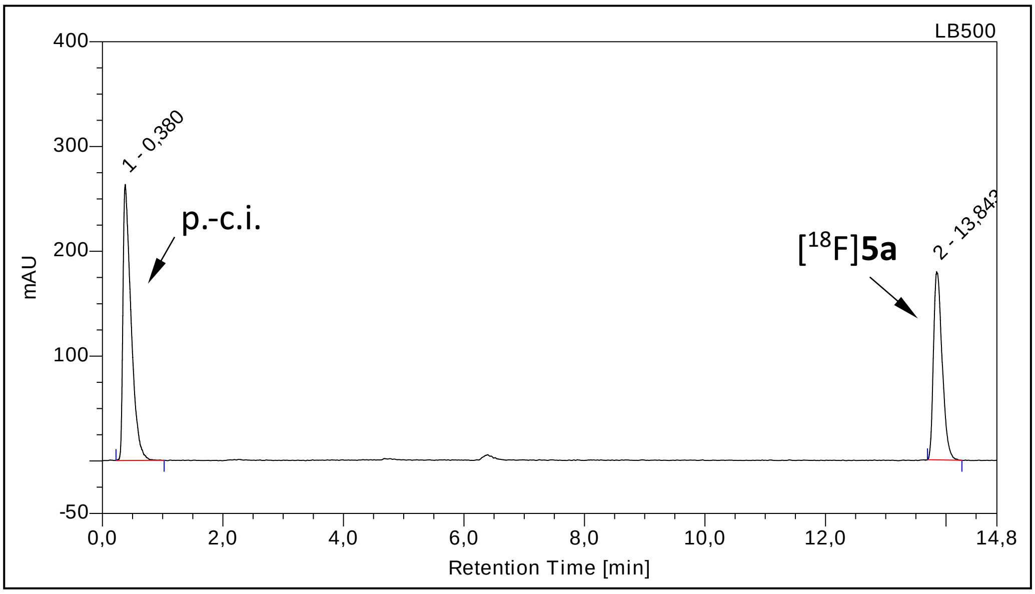

Figure S9: Radio-HPLC trace of crude [^{18}F]**5a**, Method A (0.1% TFA). Abbreviation: p.-c.i. – post-column injection.

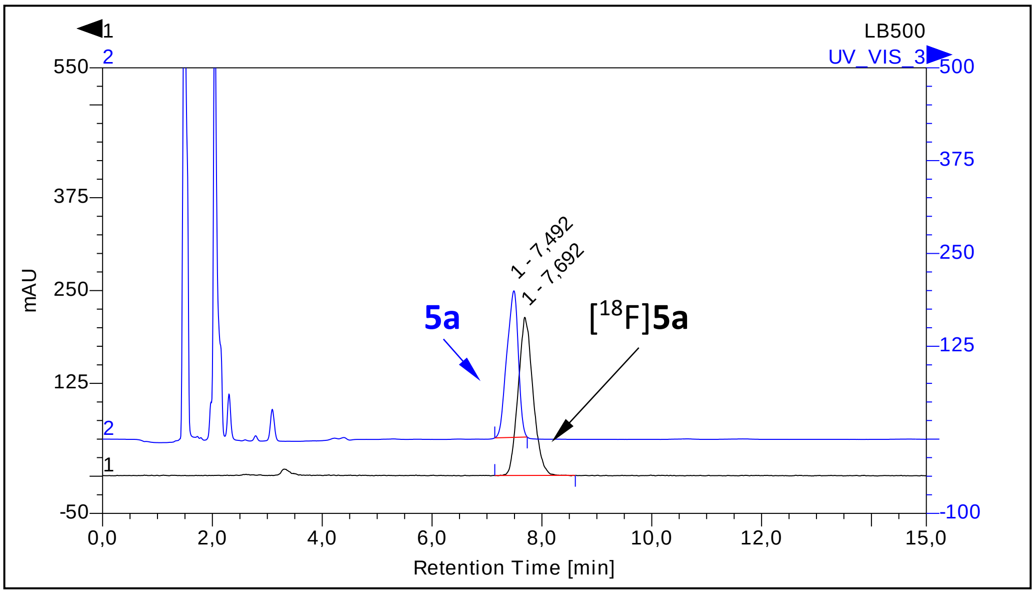

Figure S10: HPLC traces of [^{18}F]**5a** spiked with the non-radioactive reference compound, Method C. Blue trace: UV, λ = 254 nm; black trace: radioactivity.

5.2 HPLC traces of [^{18}F]**5b** (**Figs. S11** & **S12**)

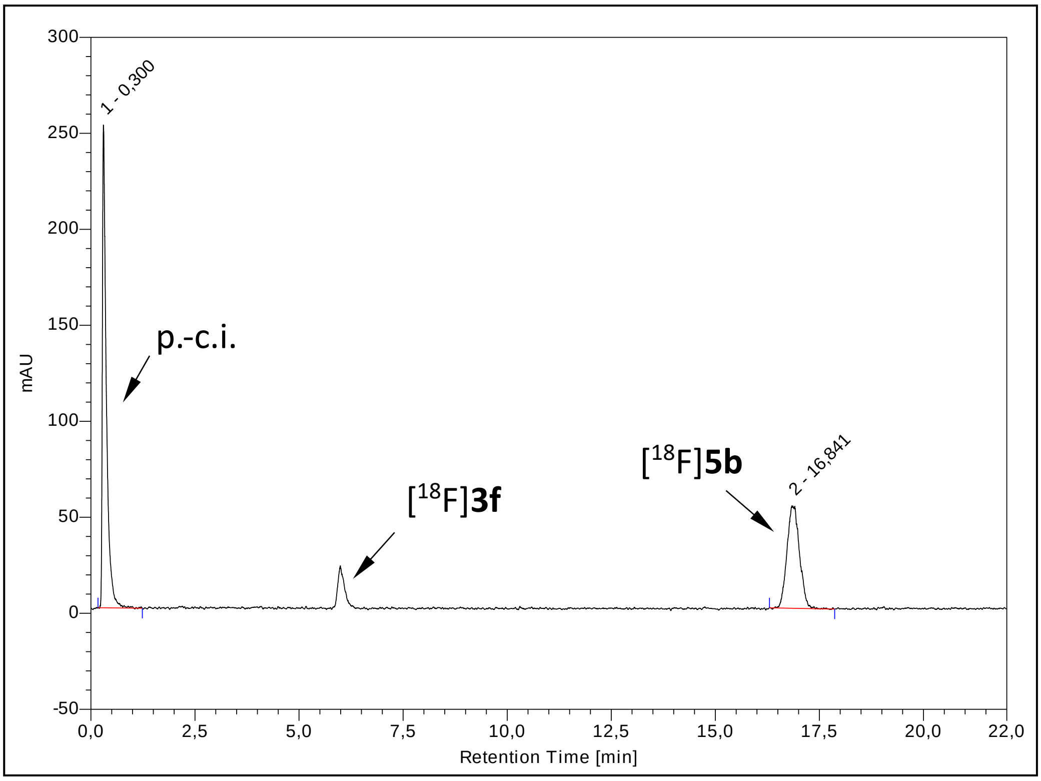

Figure S11: Radio-HPLC trace of crude [^{18}F]**5b**, Method A. Abbreviation: p.-c.i. – post-column injection.

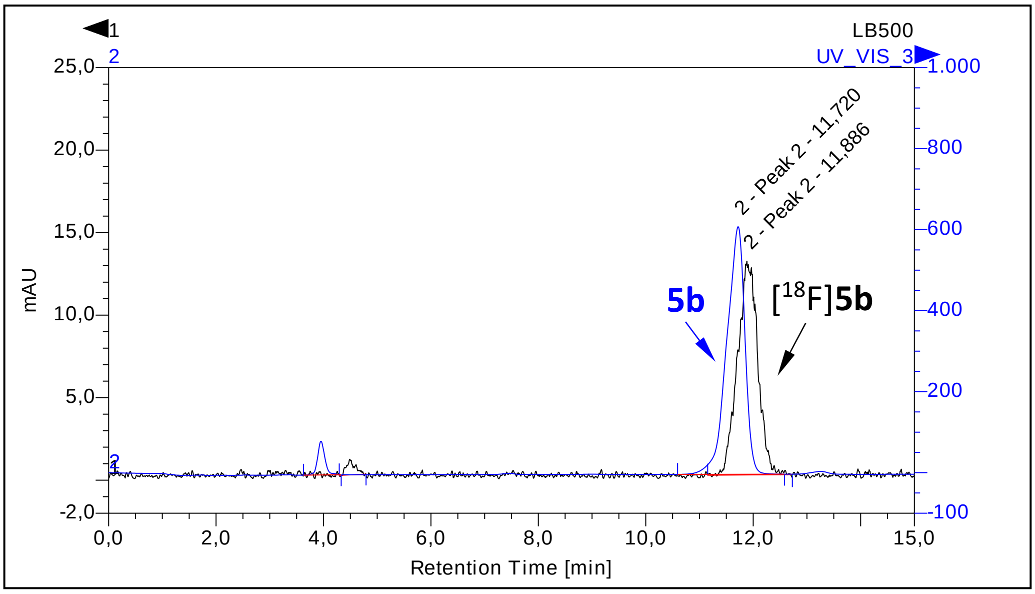

Figure S12: HPLC traces of [^{18}F]**5b** spiked with the non-radioactive reference compound, Method B. Blue trace: UV, λ = 254 nm; black trace: radioactivity.

5.3 HPLC traces of [^{18}F]**5c** (**Figs. S13** & **S14**)

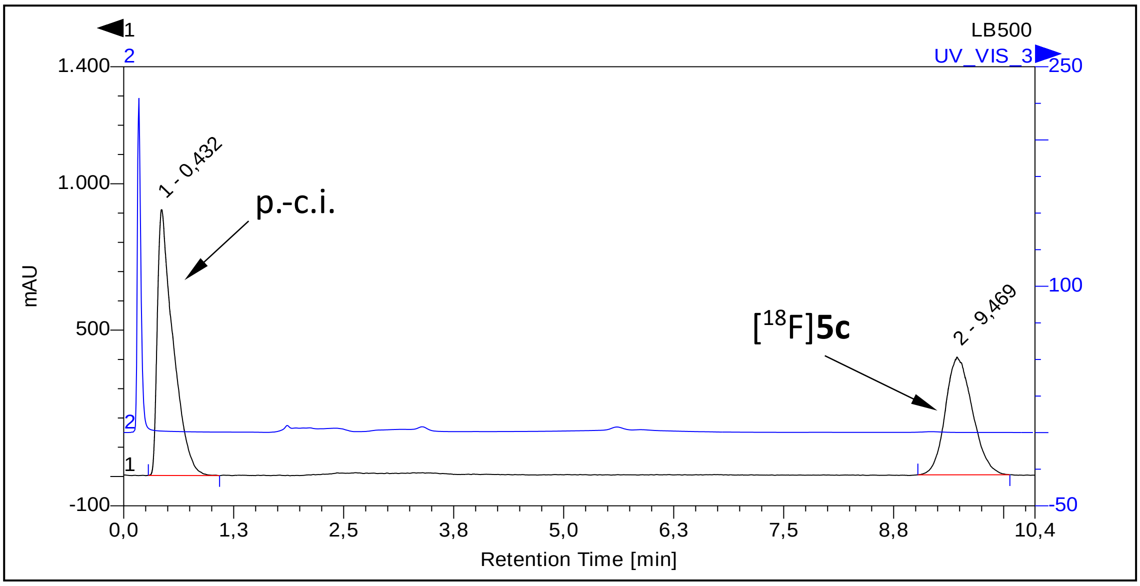

Figure S13: HPLC traces of crude [^{18}F]**5c**, Method C (0.1% TFA). Blue trace: UV, λ = 254 nm; black trace: radioactivity. Abbreviation: p.-c.i. – post-column injection.

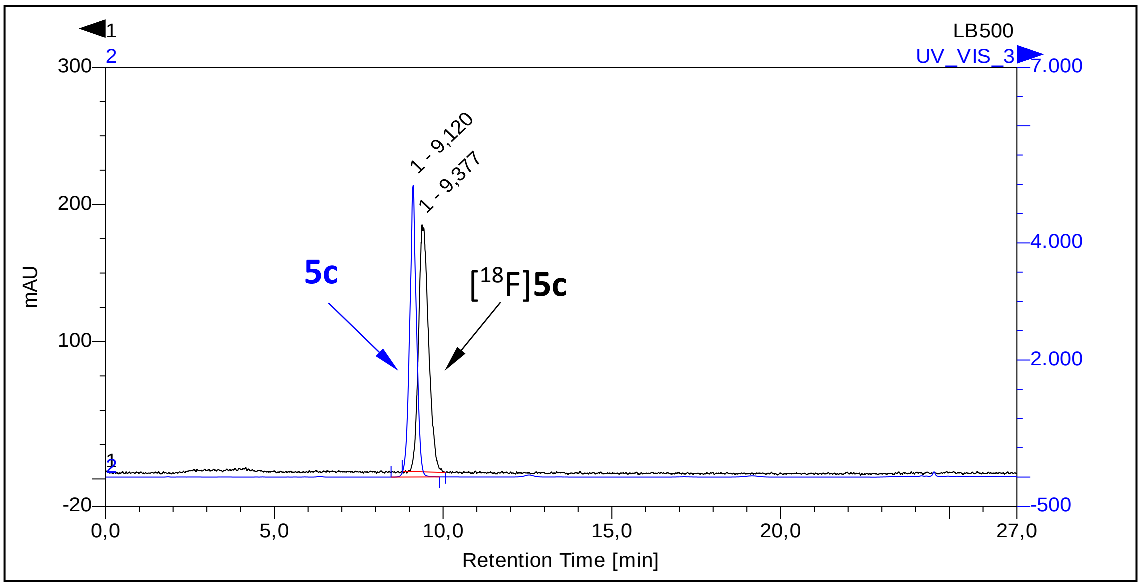

Figure S14: HPLC traces of [^{18}F]**5c** spiked with the non-radioactive reference compound, Method C (0.1% TFA). Blue trace: UV, λ = 254 nm; black trace: radioactivity.

5.4 HPLC traces of [^{18}F]**5d** (**Figs. S15** & **S16**)

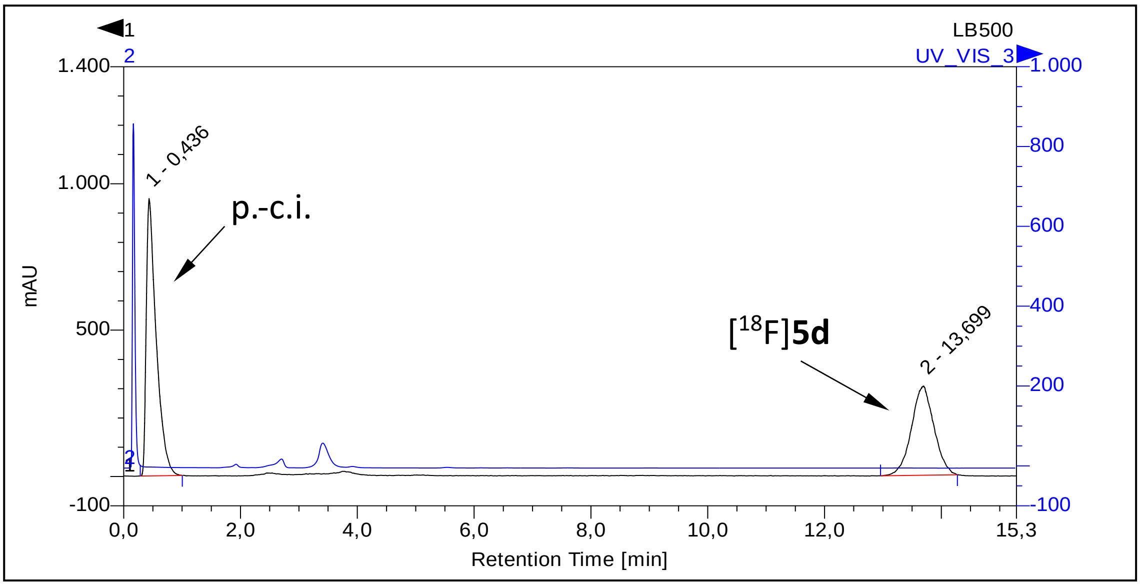

Figure S15: HPLC traces of crude [^{18}F]**5d**, Method C (0.1% TFA). Blue trace: UV, λ = 254 nm; black trace: radioactivity. Abbreviation: p.-c.i. – post-column injection.

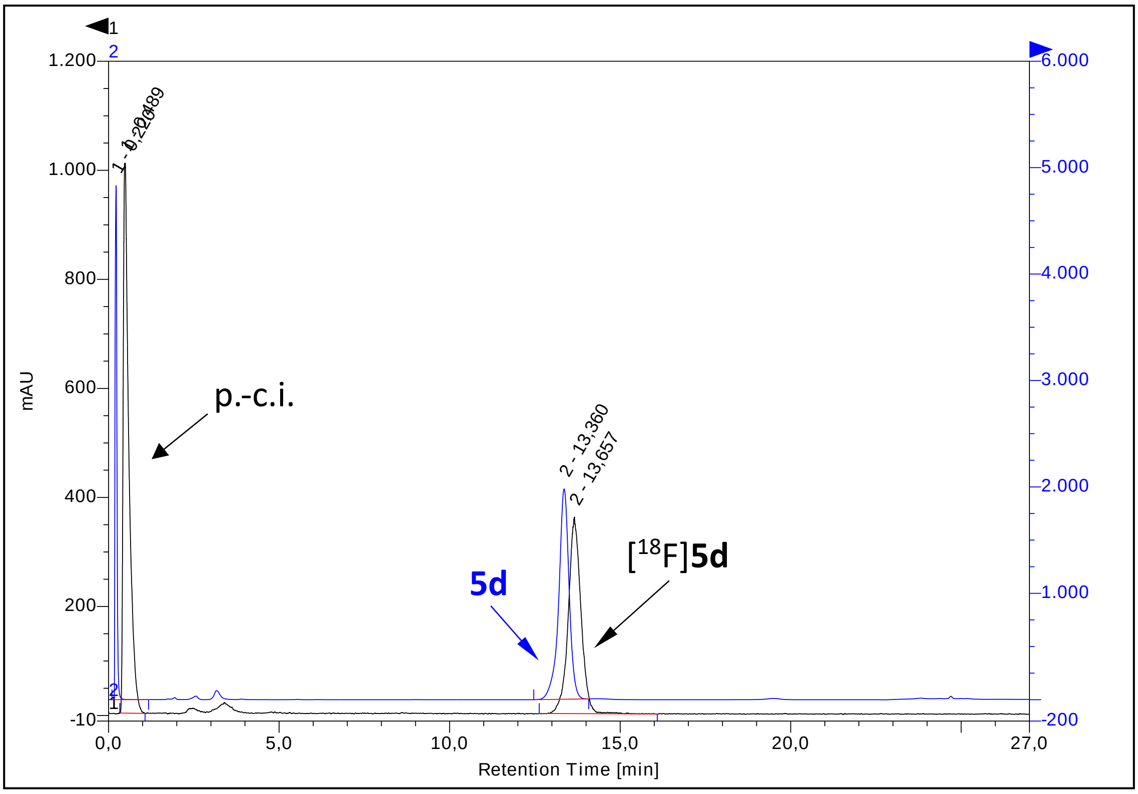

Figure S16: HPLC traces of crude [^{18}F]**5d** spiked with the non-radioactive reference compound, Method C (0.1% TFA). Blue trace: UV, λ = 254 nm; black trace: radioactivity. Abbreviation: p.-c.i. – post-column injection.

5.5 HPLC traces of [18F]**5e** (**Figs. S17** & **S18**)

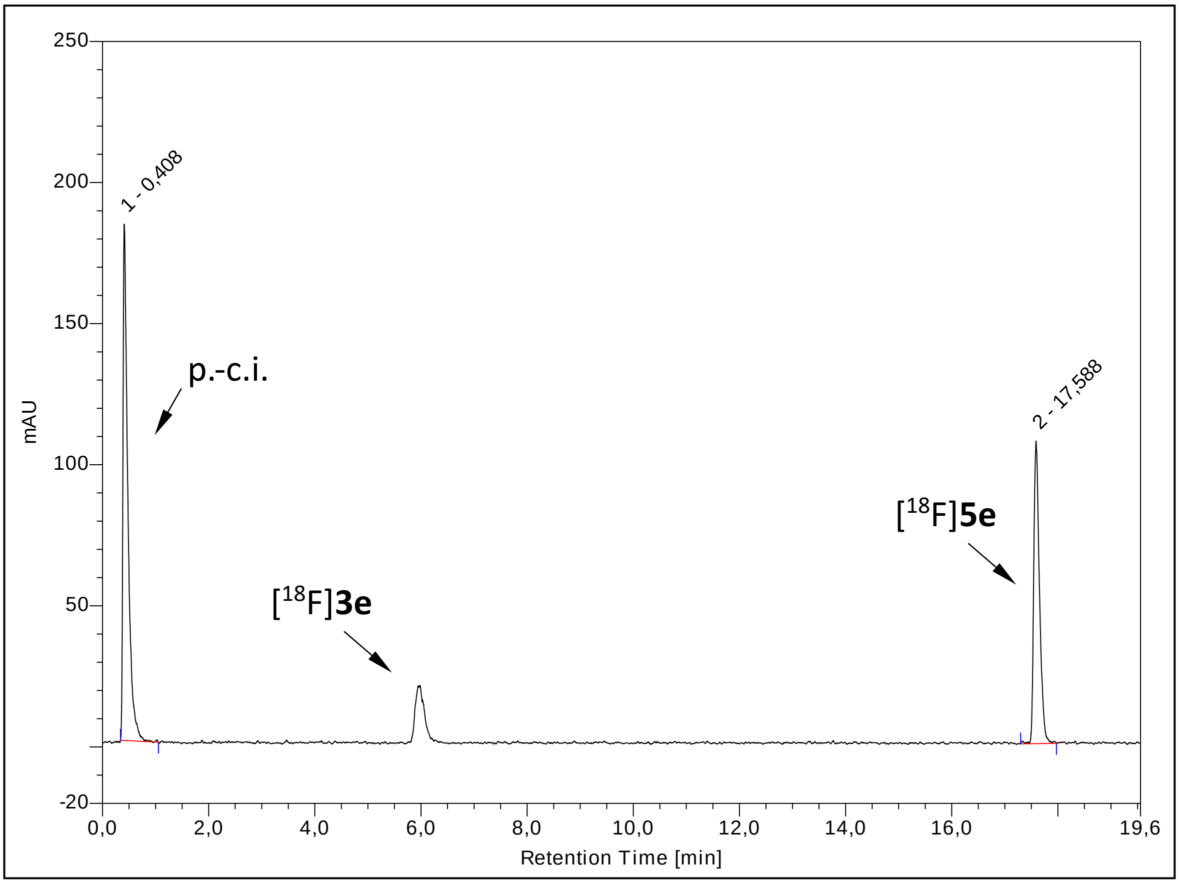

Figure S17: Radio-HPLC trace of [^{18}F]**5e**, Method B. Abbreviation: p.-c.i. – post-column injection.

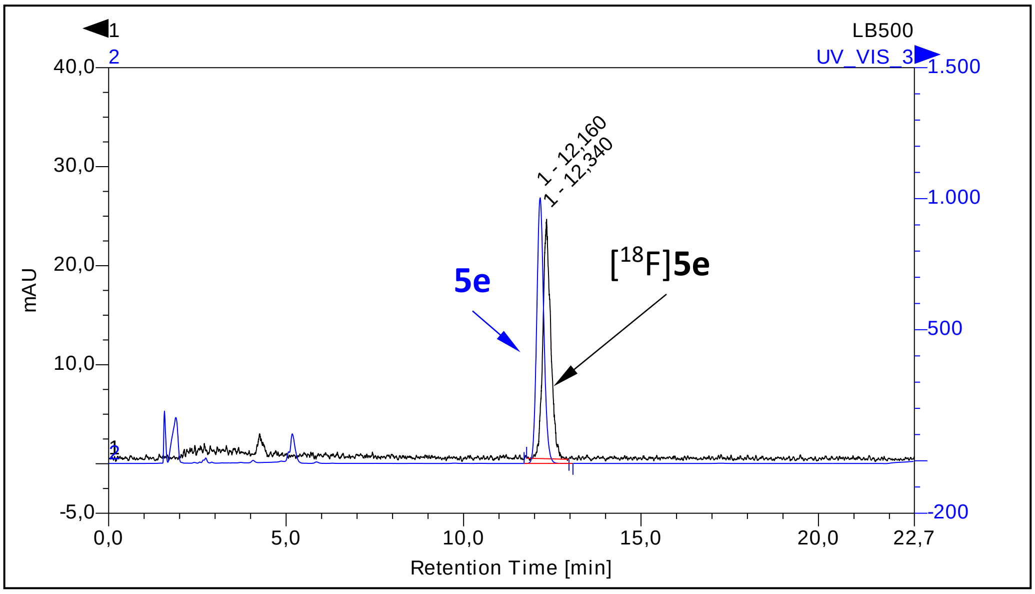

Figure S18: HPLC traces of [^{18}F]**5e** spiked with the non-radioactive reference compound, Method A. Blue trace: UV, λ = 254 nm; black trace: radioactivity.

5.6 HPLC traces of [^{18}F]**5f** (**Figs. S19** & **S20**)

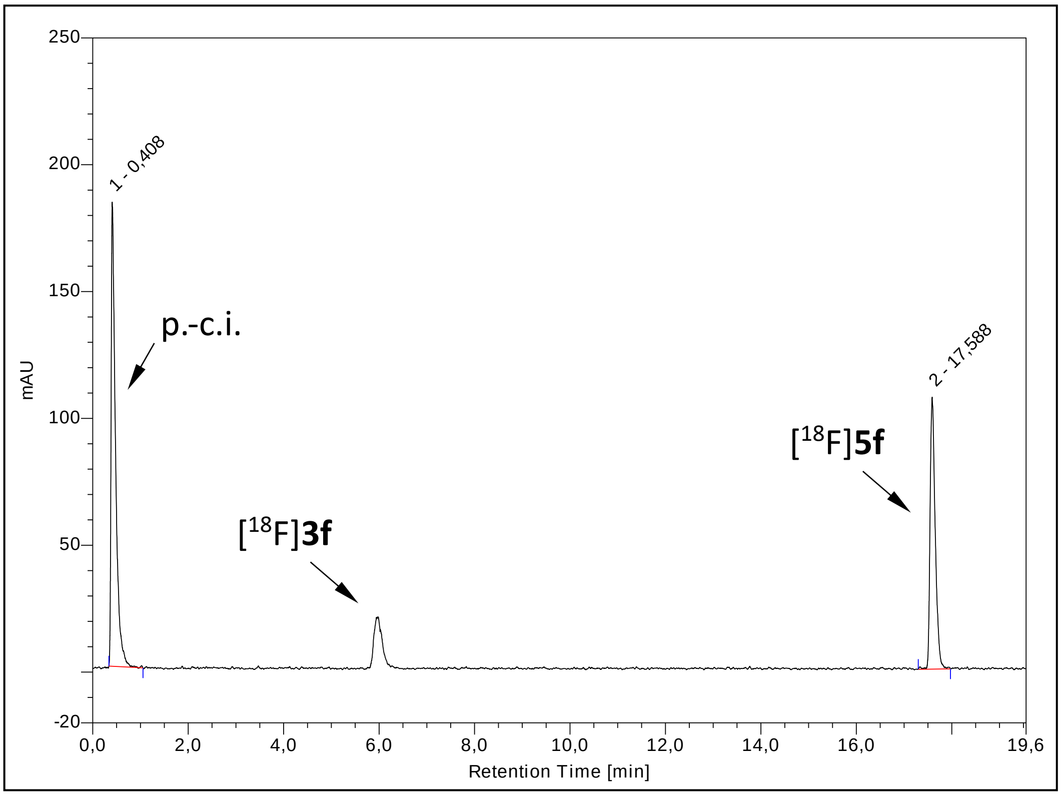

Figure S19: Radio-HPLC trace of crude [^{18}F]**5f**, Method B. Abbreviation: p.-c.i. – post-column injection.

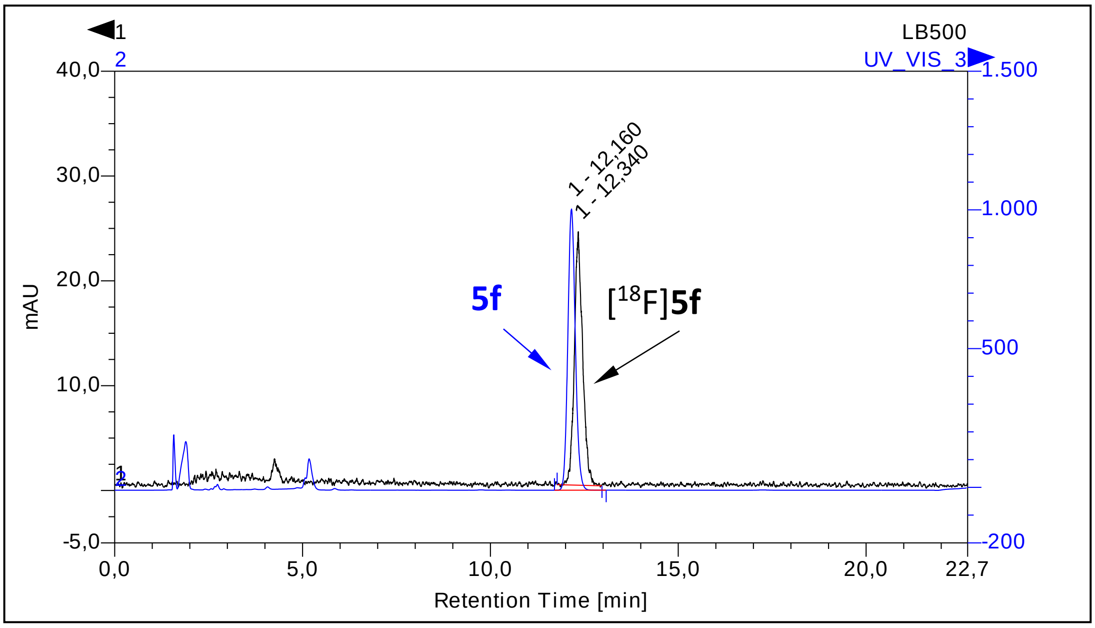

Figure S20: HPLC traces of crude [^{18}F]**5f** spiked with the non-radioactive reference compound, Method A. Blue trace: UV, λ = 254 nm; black trace: radioactivity.

5.7 HPLC traces of [^{18}F]**5g** (**Figs. S21** & **S22**)

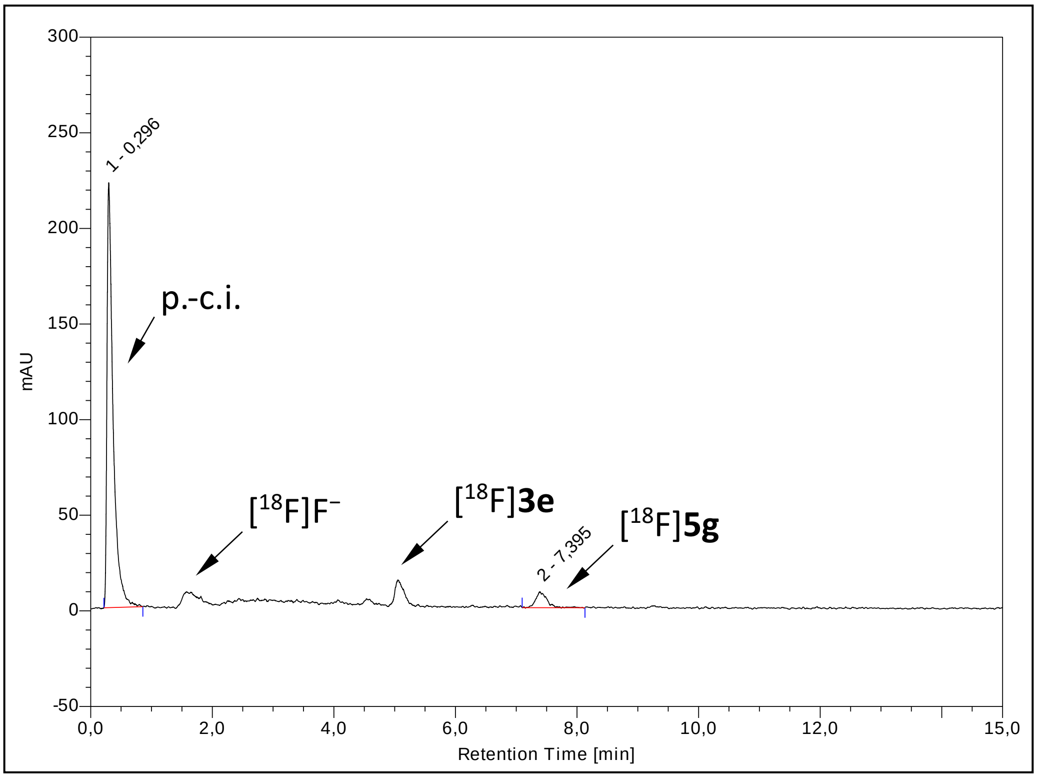

Figure S21: Radio-HPLC trace of crude [^{18}F]**5g**, Method B (0.1% TFA). Abbreviation: p.-c.i. – post-column injection.

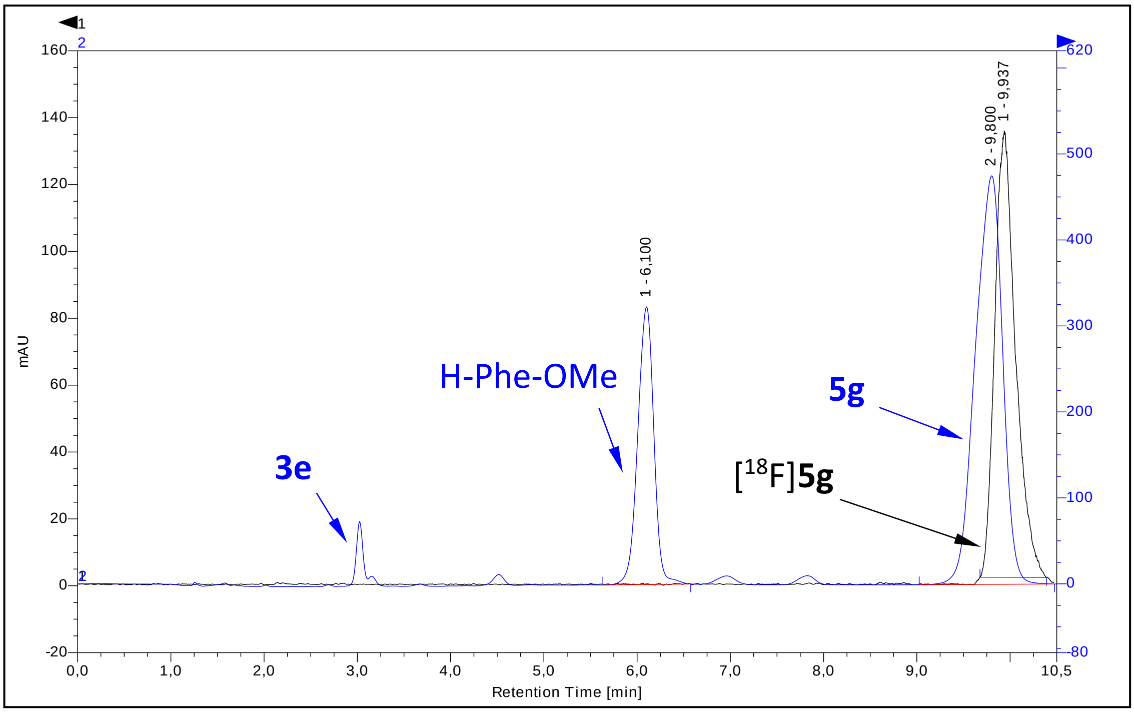

Figure S22: HPLC traces of isolated [^{18}F]**5g** spiked with the non-radioactive reference compounds, Method B. Blue trace: UV, λ = 254 nm; black trace: radioactivity.

5.8 HPLC traces of [^{18}F]**5h** (**Figs. S23** & **S24**)

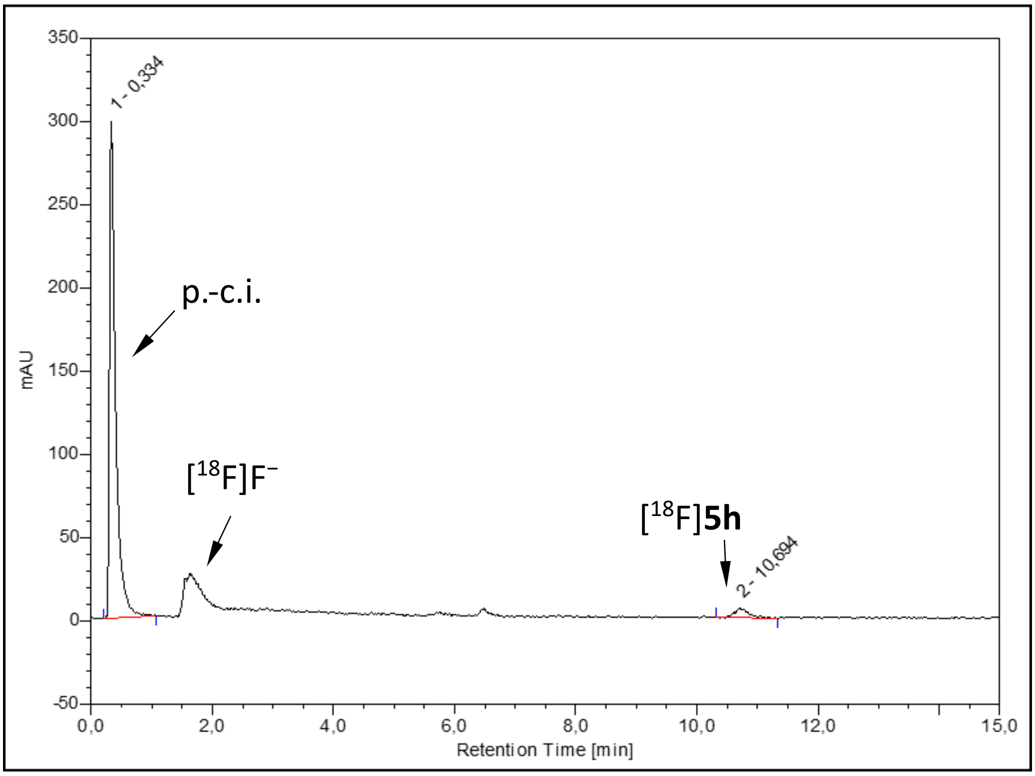

Figure S23: Radio-HPLC trace of crude [^{18}F]**5h**, Method B. Abbreviation: p.-c.i. – post-column injection.

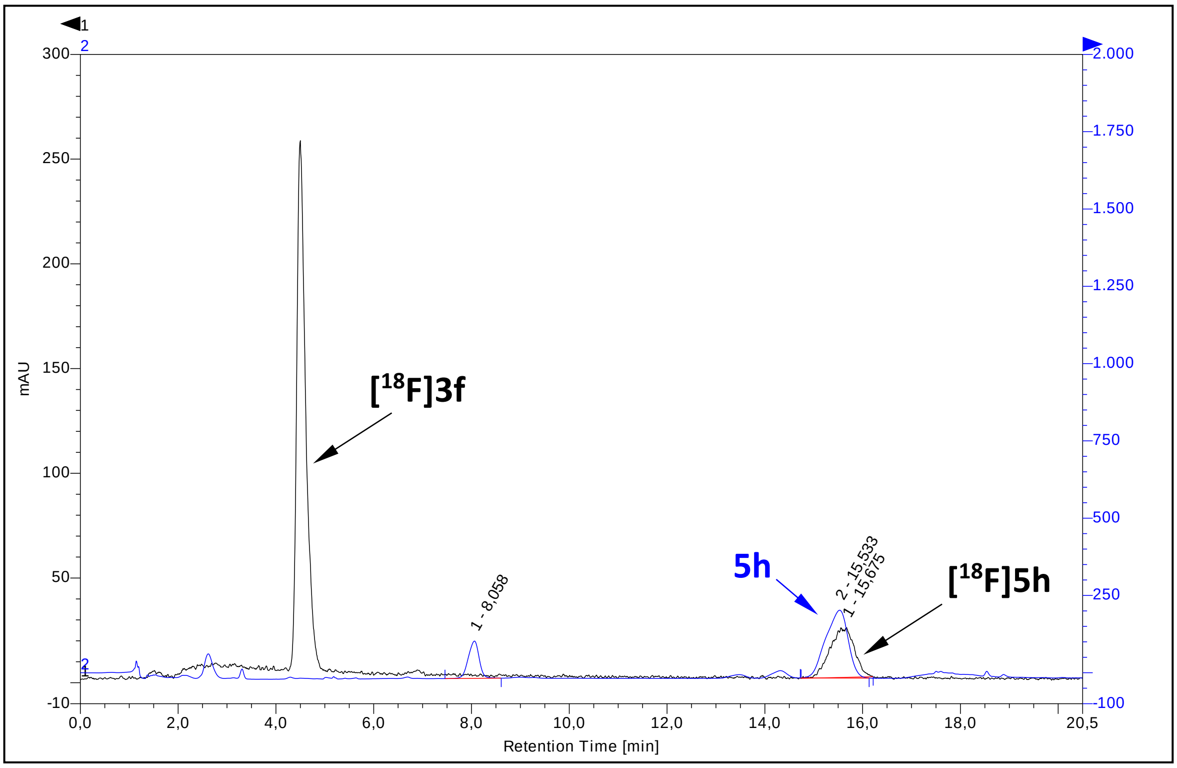

Figure S24: HPLC traces of crude [^{18}F]**5h** spiked with the non-radioactive reference compound, Method B. Blue trace: UV, λ = 254 nm; black trace: radioactivity.

5.9 HPLC traces of [^{18}F]**5i** (**Figs. S25** & **S26**)

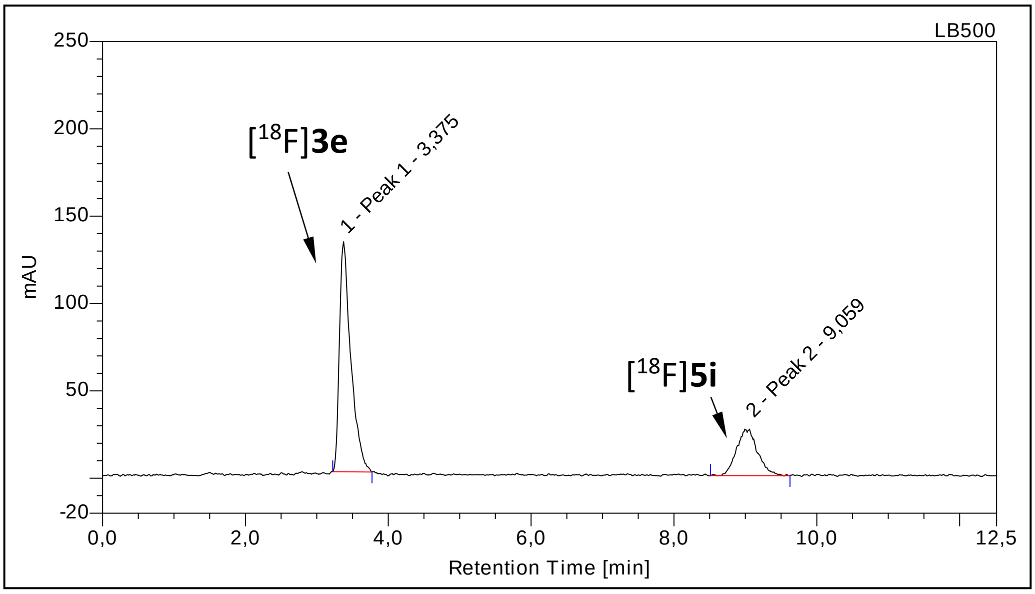

Figure S25: Radio-HPLC trace of crude [^{18}F]**5i**, Method A.

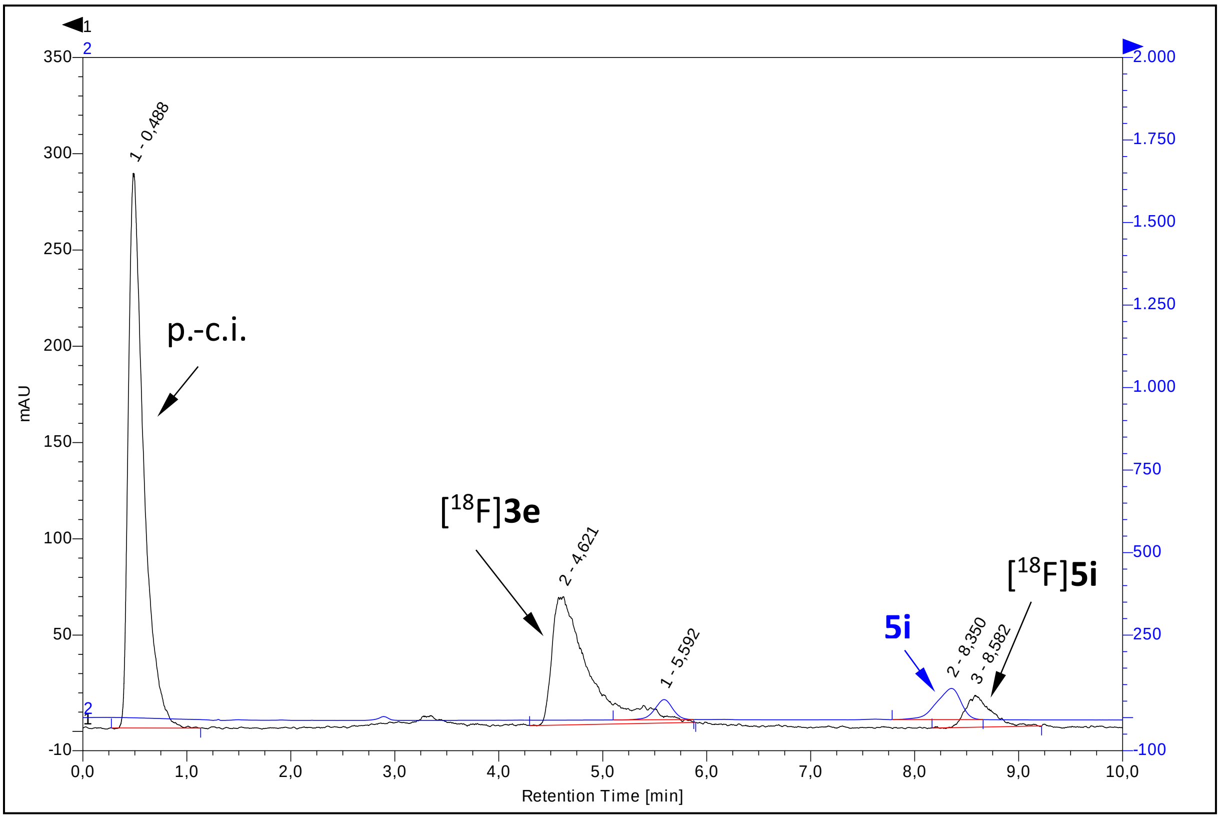

Figure S26: HPLC traces of crude [^{18}F]**5i** spiked with the non-radioactive reference compound, Method B. Blue trace: UV, λ = 254 nm; black trace: radioactivity. Abbreviation: p.-c.i. – post-column injection.

5.10 HPLC traces of [^{18}F]**5j** (**Figs. S27** & **S28**)

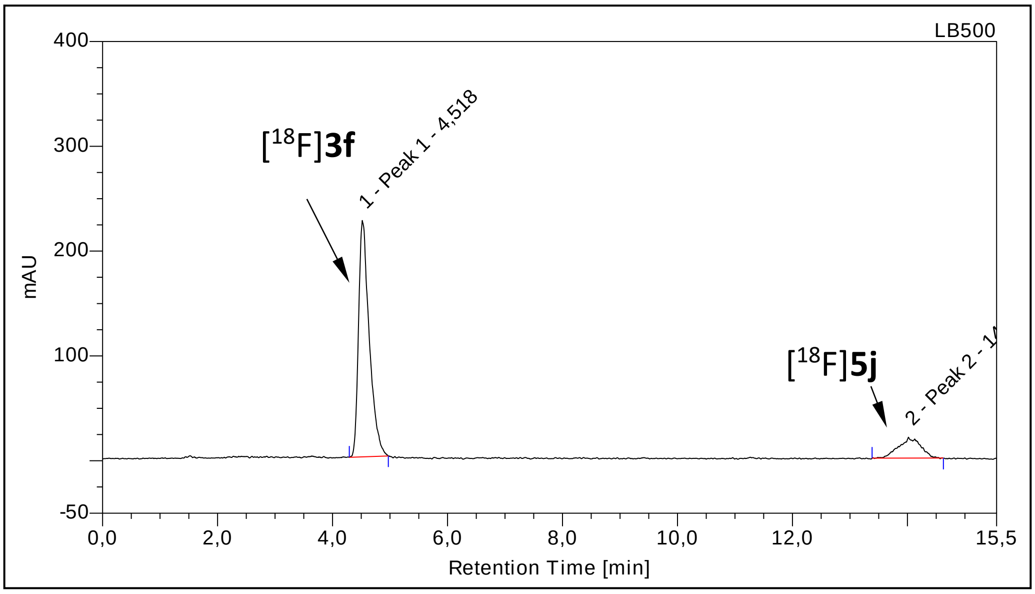

Figure S27: Radio-HPLC trace of crude [^{18}F]**5j**, Method A.

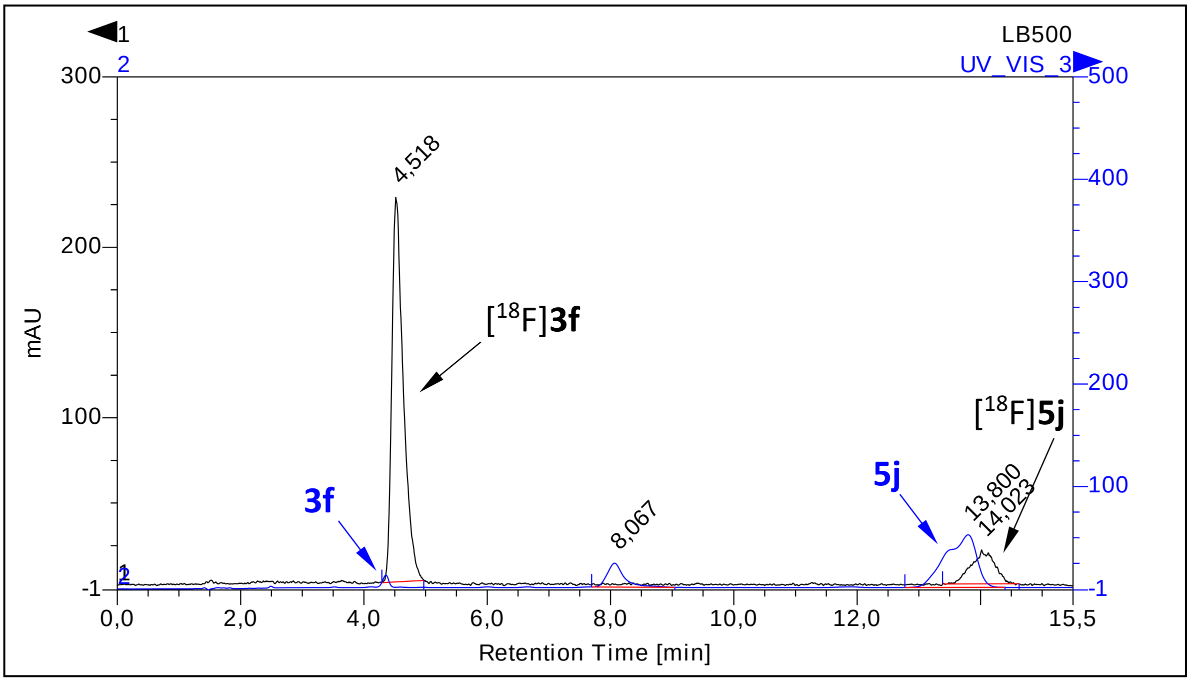

Figure S28: HPLC traces of crude [^{18}F]**5j** spiked with the non-radioactive reference compounds, Method B. Blue trace: UV, λ = 254 nm; black trace: radioactivity.

5.11 HPLC traces of [^{18}F]**5k** (**Figs. S29** & **S30**)

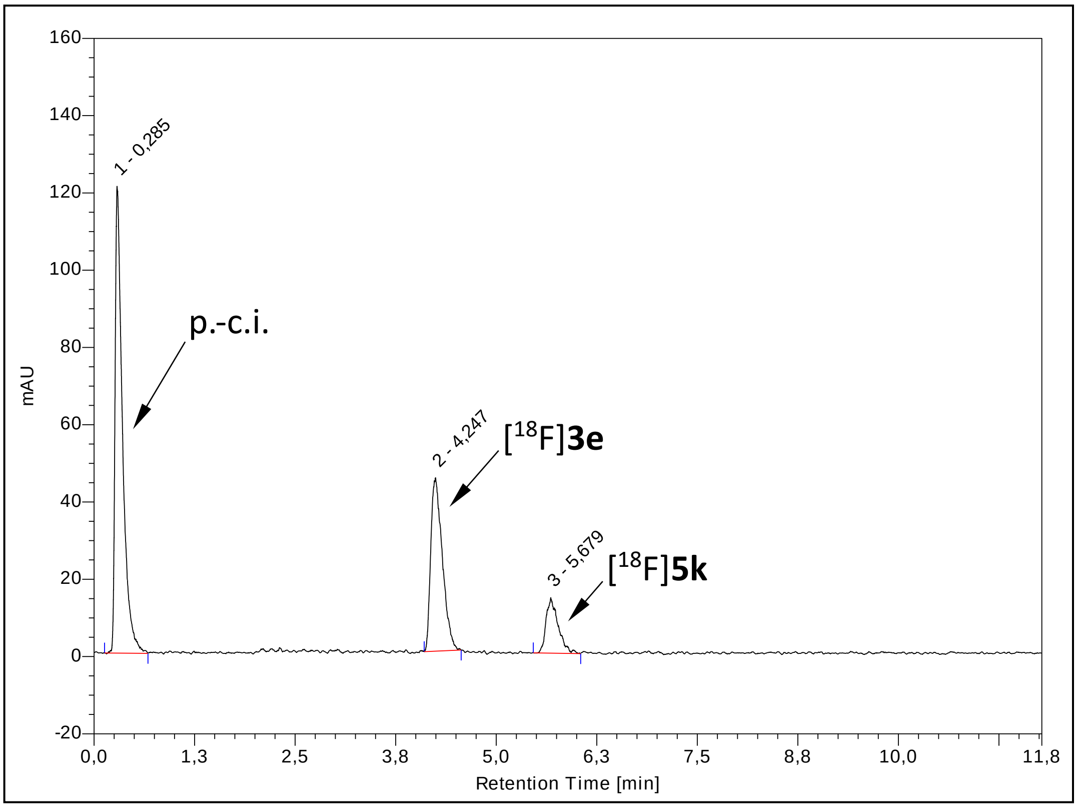

Figure S29: Radio-HPLC trace of [^{18}F]**5k**, Method A. Abbreviation: p.-c.i. – post-column injection.

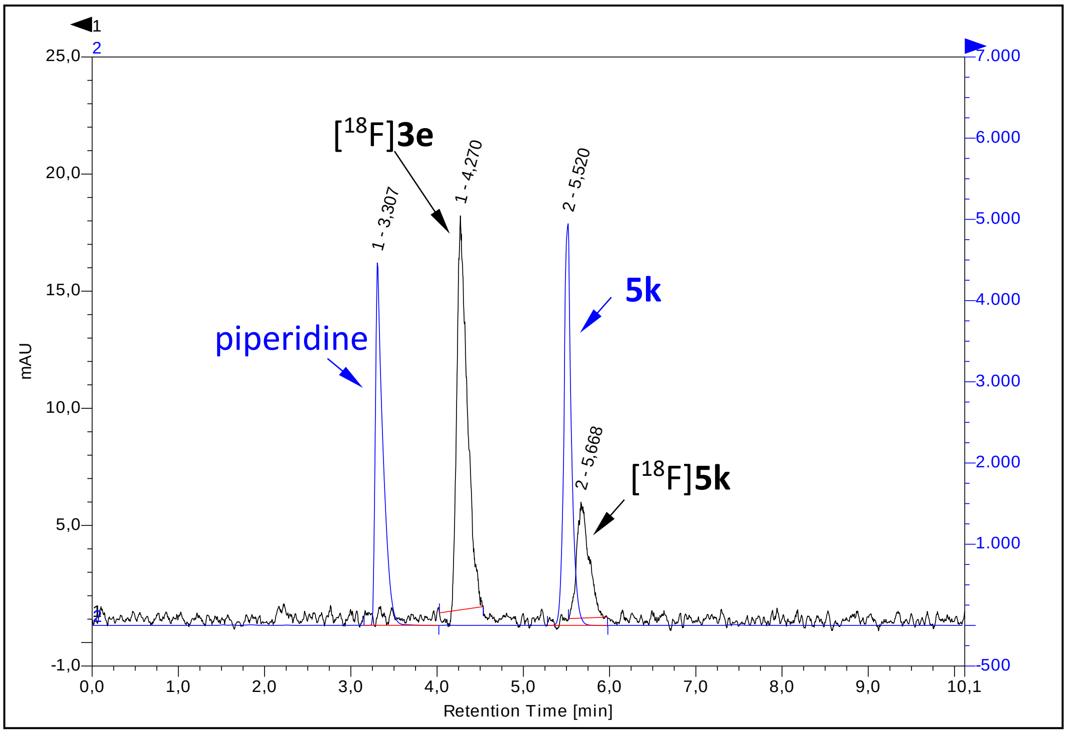

Figure S30: HPLC traces of crude [^{18}F]**5k** spiked with the non-radioactive reference compound, Method B. Blue trace: UV, λ = 254 nm; black trace: radioactivity.

5.12 HPLC trace of [^{18}F]**5l** (**Fig. S31**)

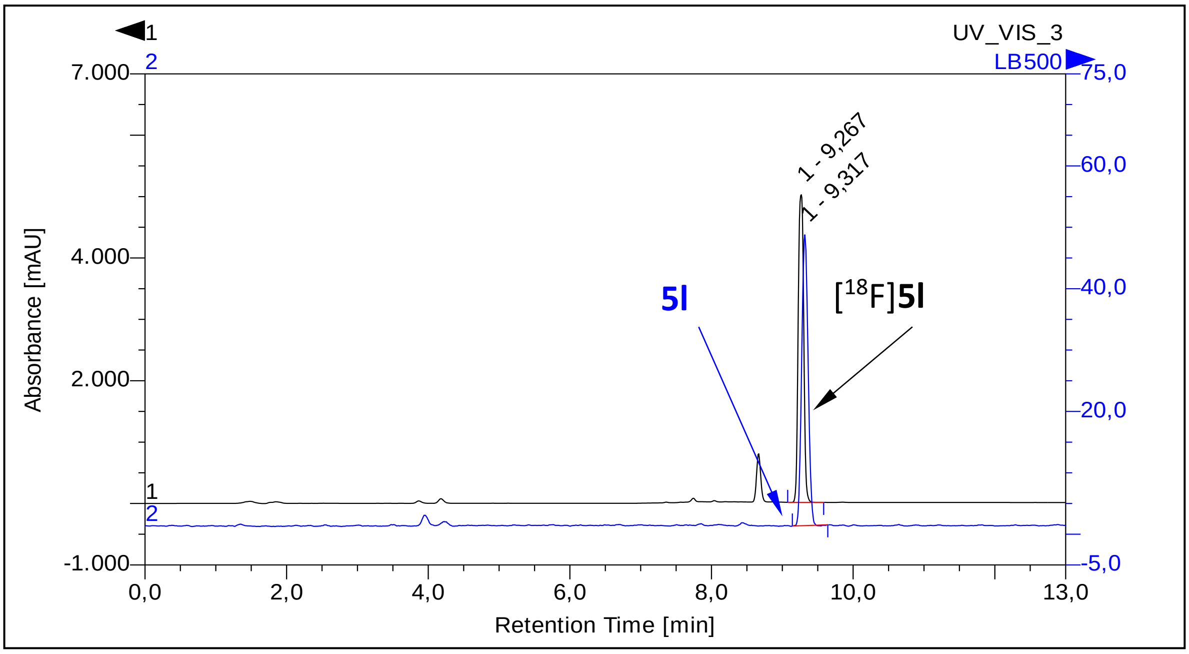

Figure S31: HPLC traces of crude [^{18}F]**5l** spiked with the non-radioactive reference compound, Method A (0.1% TFA). Blue trace: UV, λ = 254 nm; black trace: radioactivity.

5.13 HPLC trace of [18F]**5m** (**Fig. S32**)

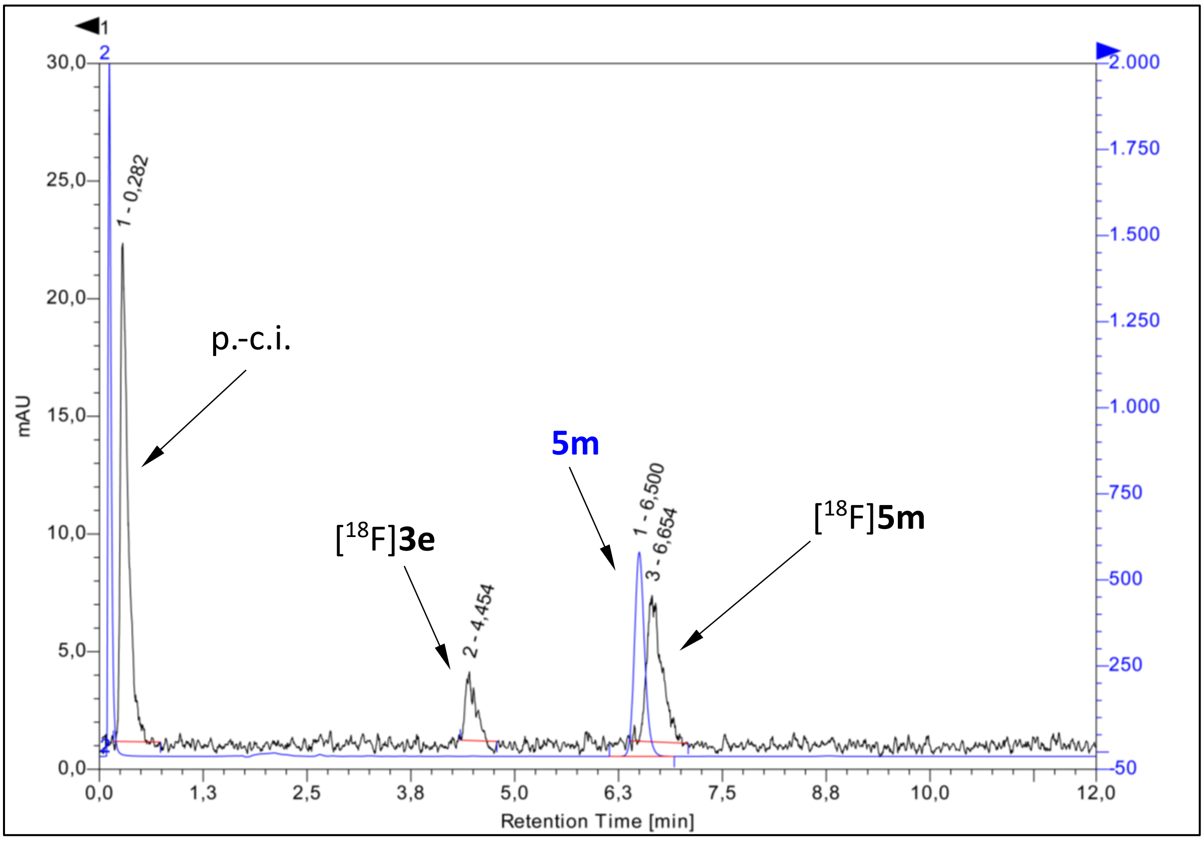

Figure S32: HPLC traces of crude [18F]**5m** spiked with the non-radioactive reference compound, Method B. Blue trace: UV, λ = 254 nm; black trace: radioactivity. Abbreviation: p.-c.i. – post-column injection.

5.14 HPLC trace of [^{18}F]**5o** (**Fig. S33**)

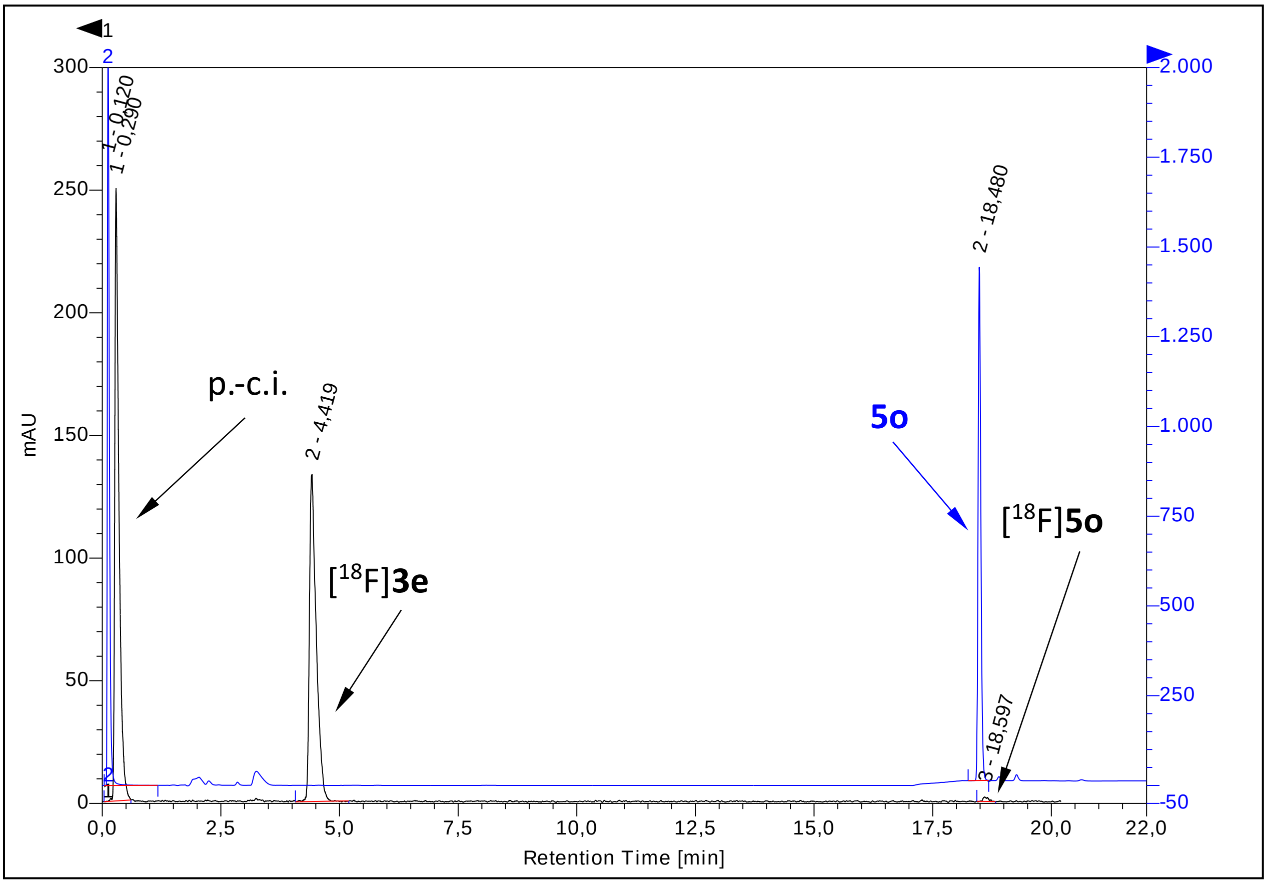

Figure S33: HPLC traces of crude [^{18}F]**5o** spiked with the non-radioactive reference compound, Method B. Blue trace: UV, λ = 254 nm; black trace: radioactivity. Abbreviation: p.-c.i. – post-column injection.

5.15 HPLC trace for competition between Ac-Lys(H)-O*t*Bu and H-Lys(Z)-O*t*Bu (4:1) (**Fig. S34**)

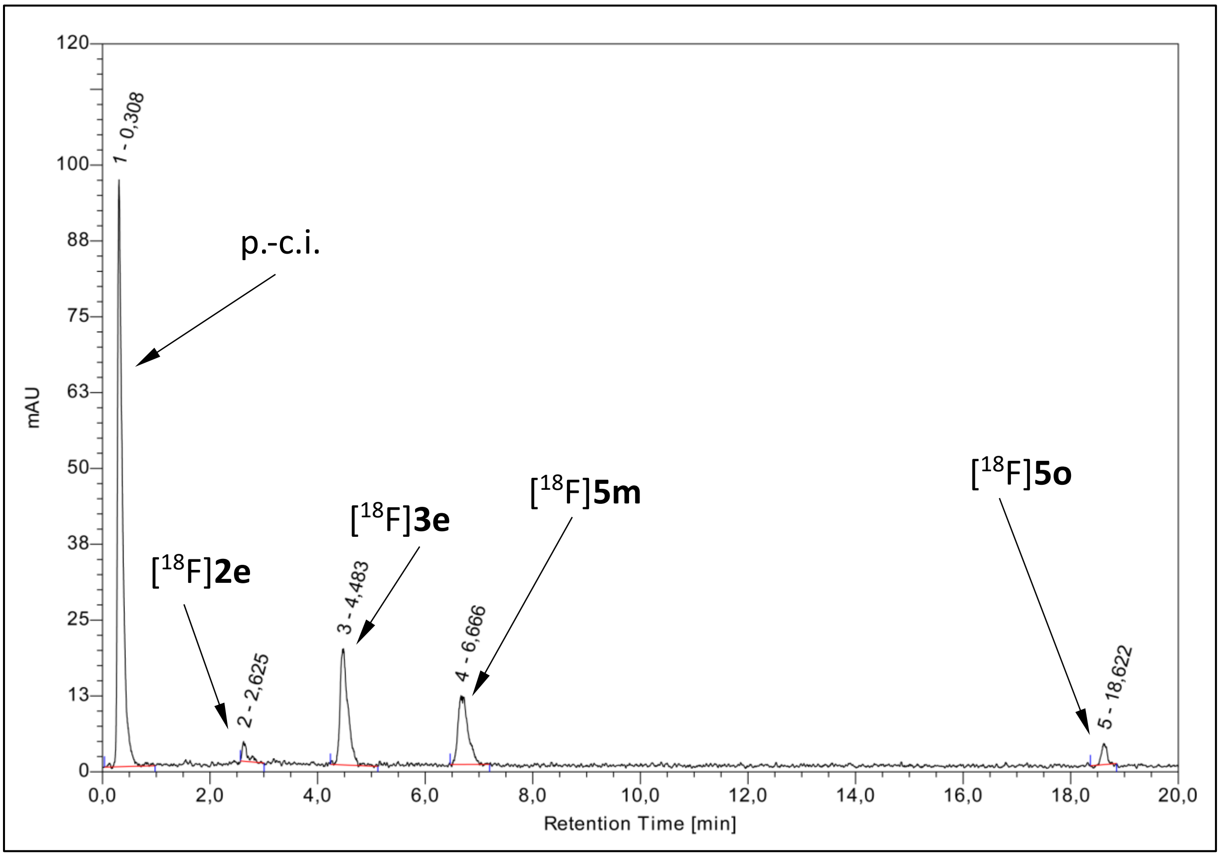

Figure S34: HPLC trace of the competition experiment, Method B. Blue trace: UV, λ = 254 nm; black trace: radioactivity. Abbreviation: p.-c.i. – post-column injection.

5.16 HPLC trace for competition between *n*-butylamine and H-Gly-OMe (**Fig. S35**)

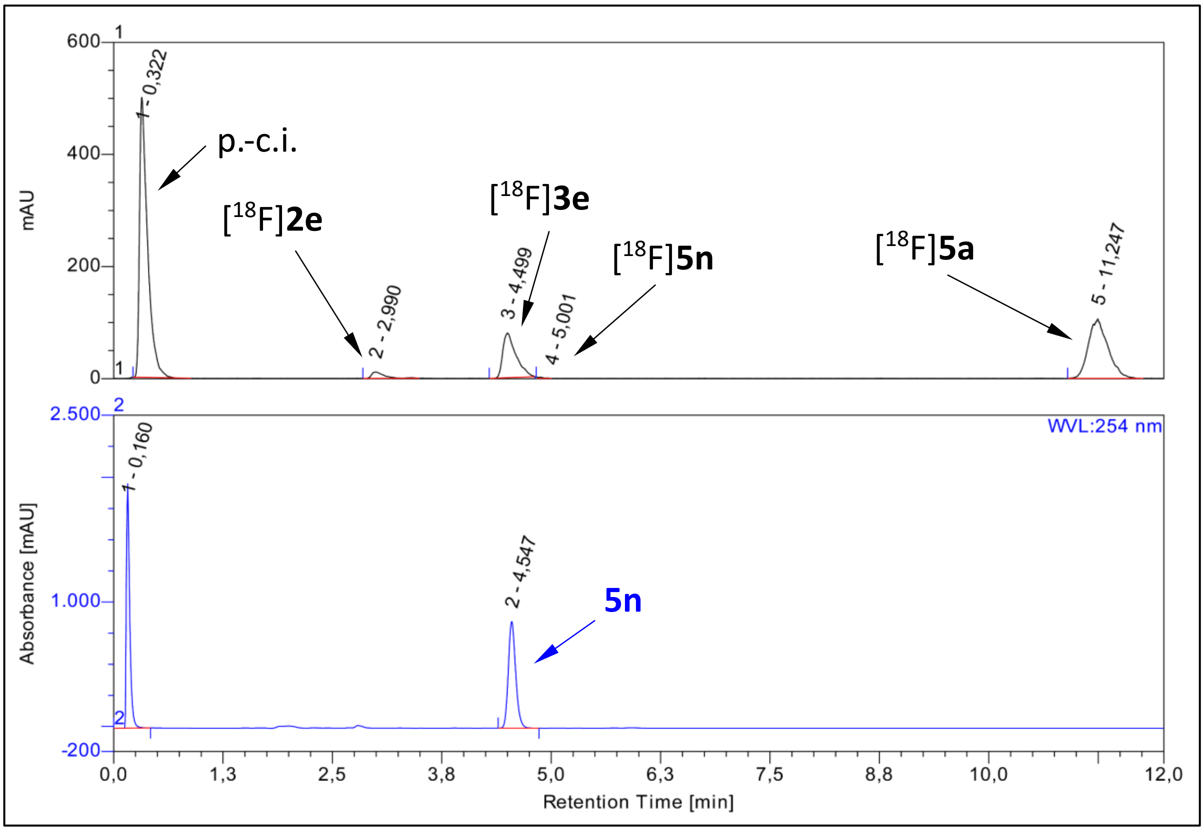

Figure S35: HPLC traces of competition experiment spiked with the non-radioactive reference compound **5n**, Method A (0.1% TFA). Blue trace: UV, λ = 254 nm; black trace: radioactivity. Abbreviation: p.-c.i. – post-column injection.

5.17 HPLC traces of [^{18}F]**5p** ([^{18}F]JK-PSMA-15) (**Figs. S36-S38**)

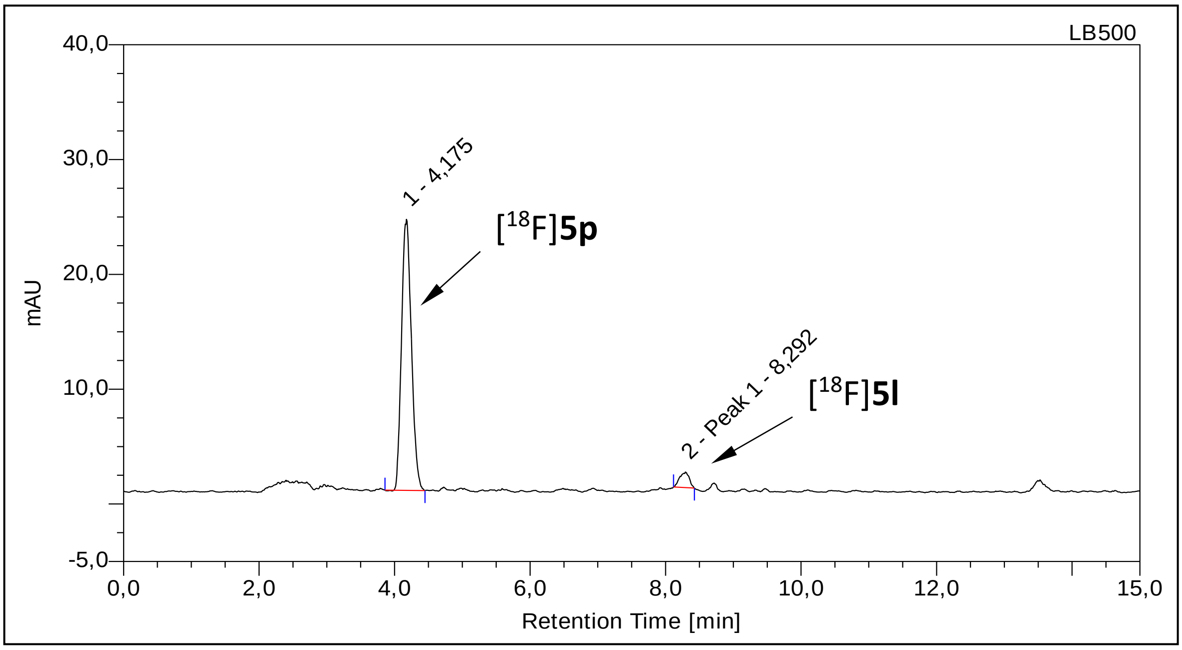

Figure S36: Radio-HPLC trace of crude [^{18}F]**5p** ([^{18}F]JK-PSMA-15), Method E (0.1% TFA).

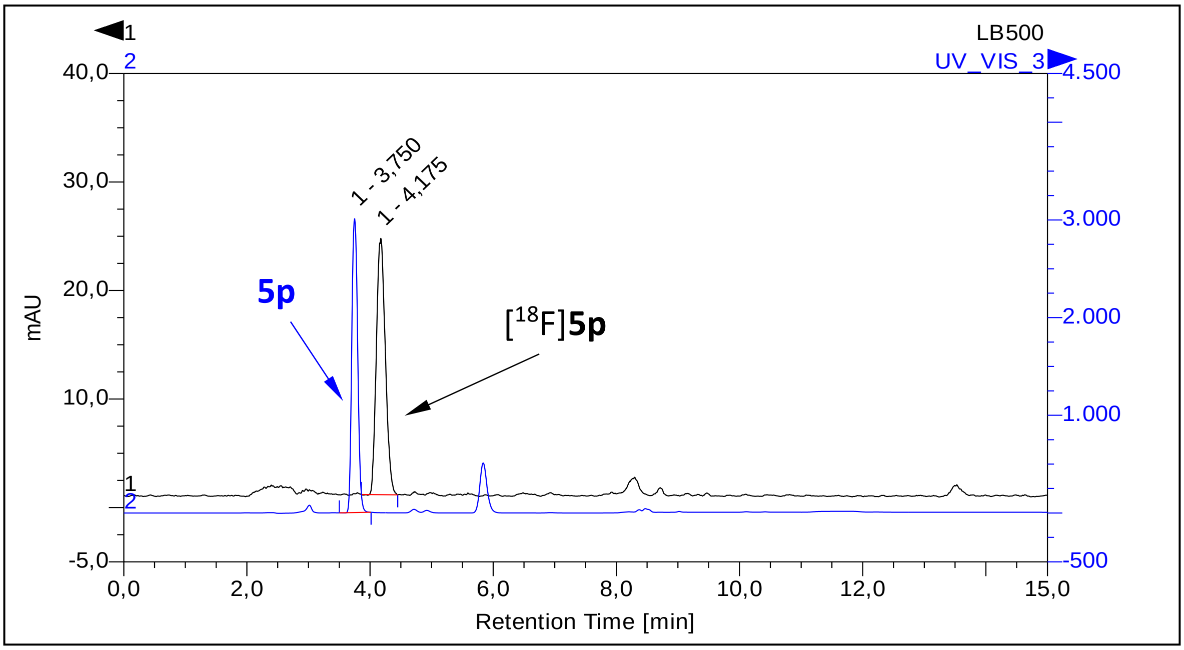

Figure S37: HPLC traces of crude [^{18}F]**5p** ([^{18}F]JK-PSMA-15) spiked with the non-radioactive reference compound, Method E (0.1% TFA). Blue trace: UV, λ = 254 nm; black trace: radioactivity.

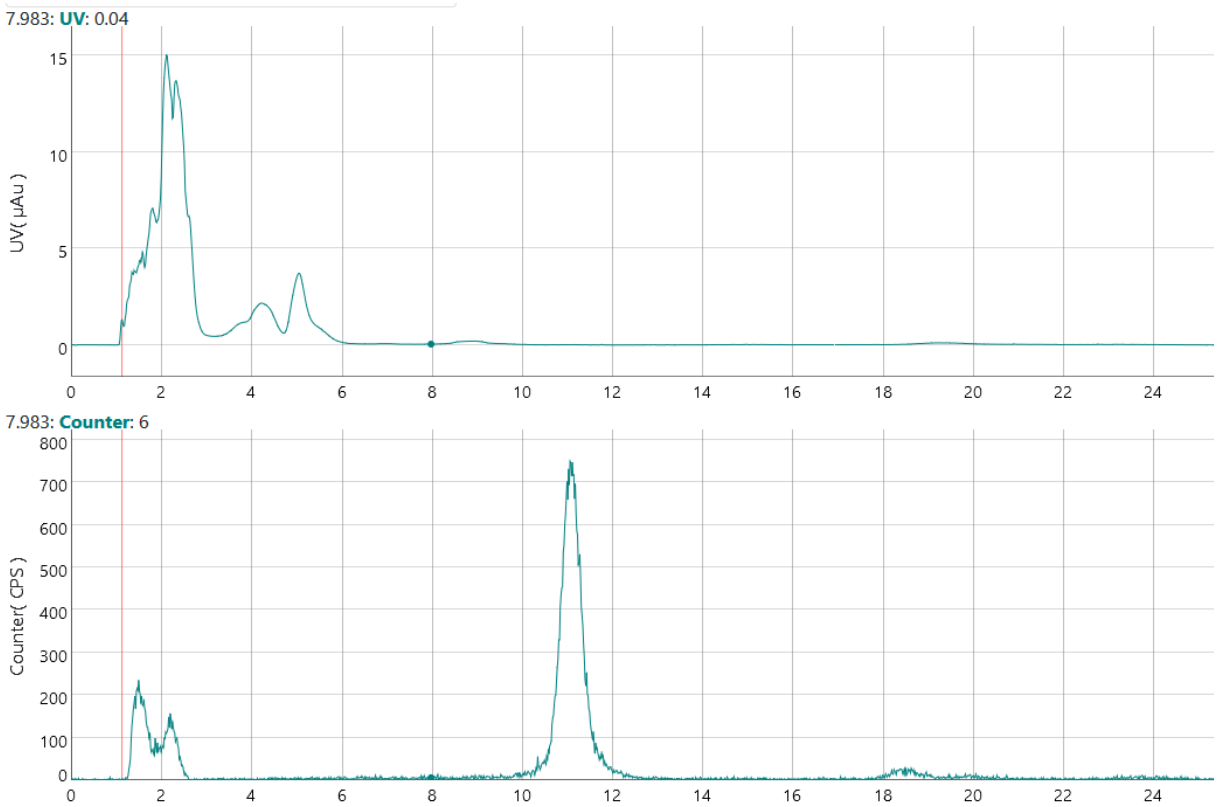

Figure S38: Purification of [^{18}F]**5p** ([^{18}F]JK-PSMA-15) by preparative HPLC (top: UV chromatogram, λ=254 nm; bottom: radio-chromatogram; column: Hydro-RP, 250×10 mm; eluent: 30% MeCN (0.1% TFA); flow rate: 4.7 mL/min; t_R = 11 min).

5.18 HPLC traces of [^{18}F]**6a** (**Figs. S39** & **S40**)

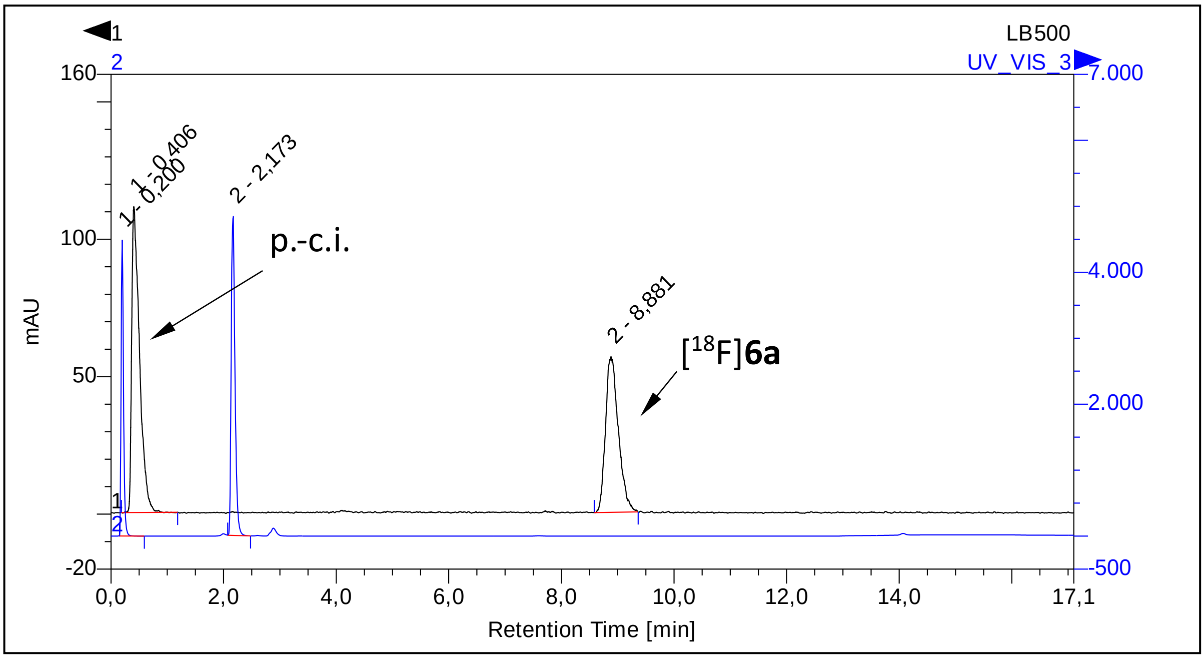

Figure S39: HPLC traces of crude [^{18}F]**6a**, Method A (0.1% TFA). Blue trace: UV, λ = 254 nm; black trace: radioactivity. Abbreviation: p.-c.i. – post-column injection.

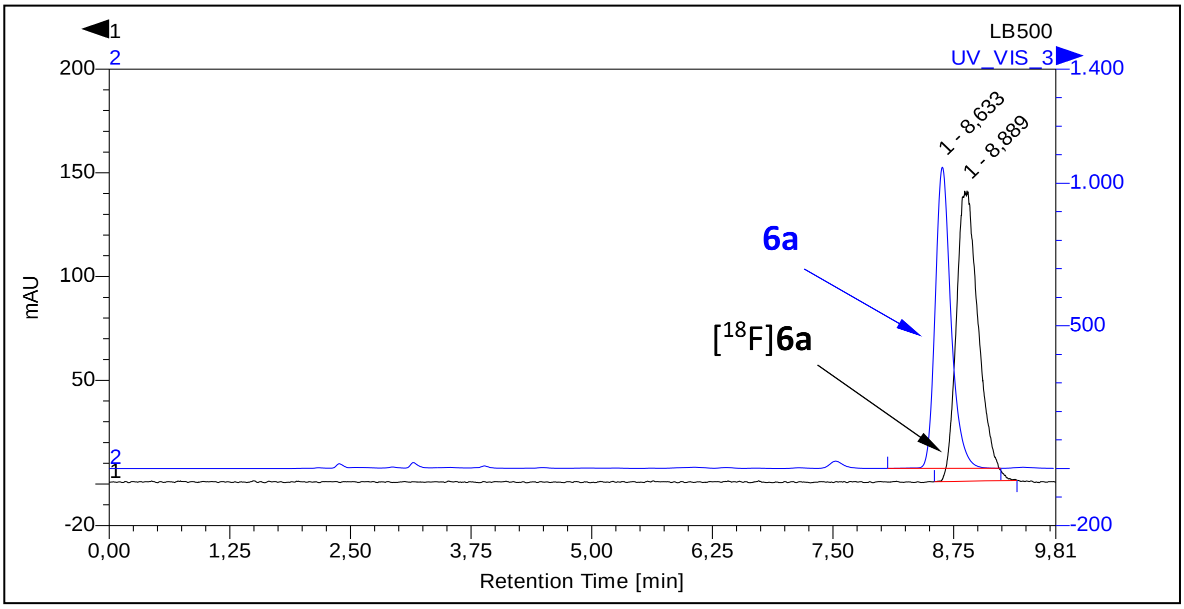

Figure S40: HPLC traces of crude [^{18}F]**6a** spiked with the non-radioactive reference compound, Method A (0.1% TFA). Blue trace: UV, λ = 254 nm; black trace: radioactivity.

5.19 HPLC traces of [^{18}F]**6b** ([^{18}F]JK-PSMA-16) (**Figs. S41-S43**)

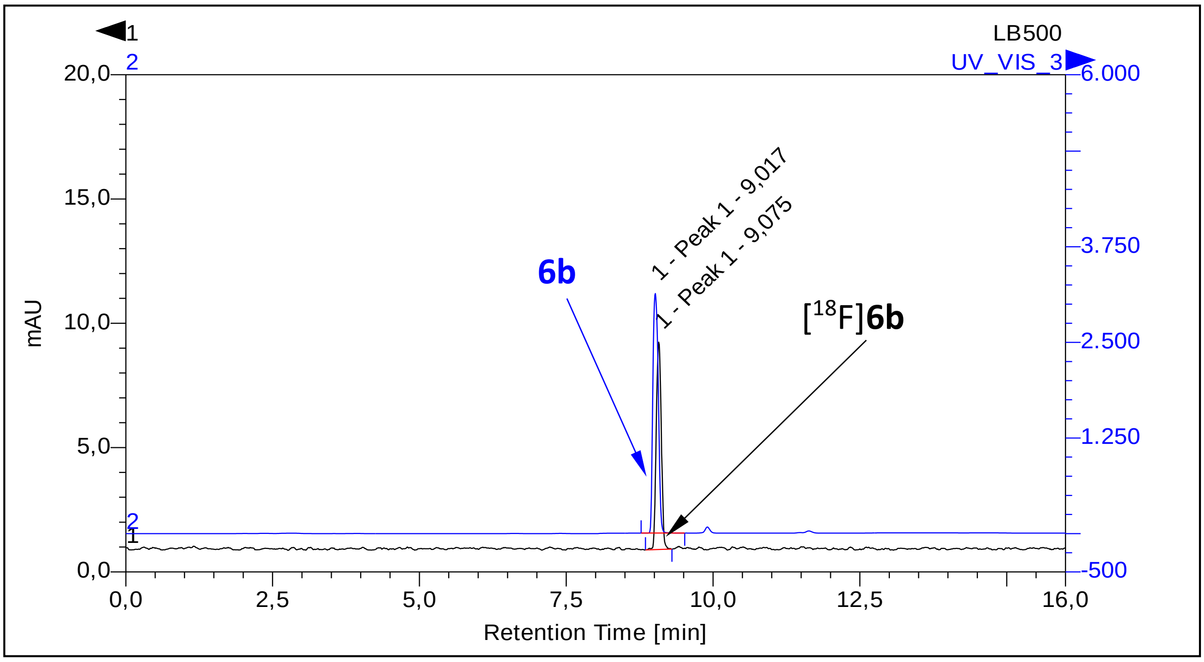

Figure S41: HPLC traces of purified [^{18}F]**6b** ([^{18}F]JK-PSMA-16) spiked with the non-radioactive reference compound, Method E (0.1% TFA). Blue trace: UV, λ = 254 nm; black trace: radioactivity.

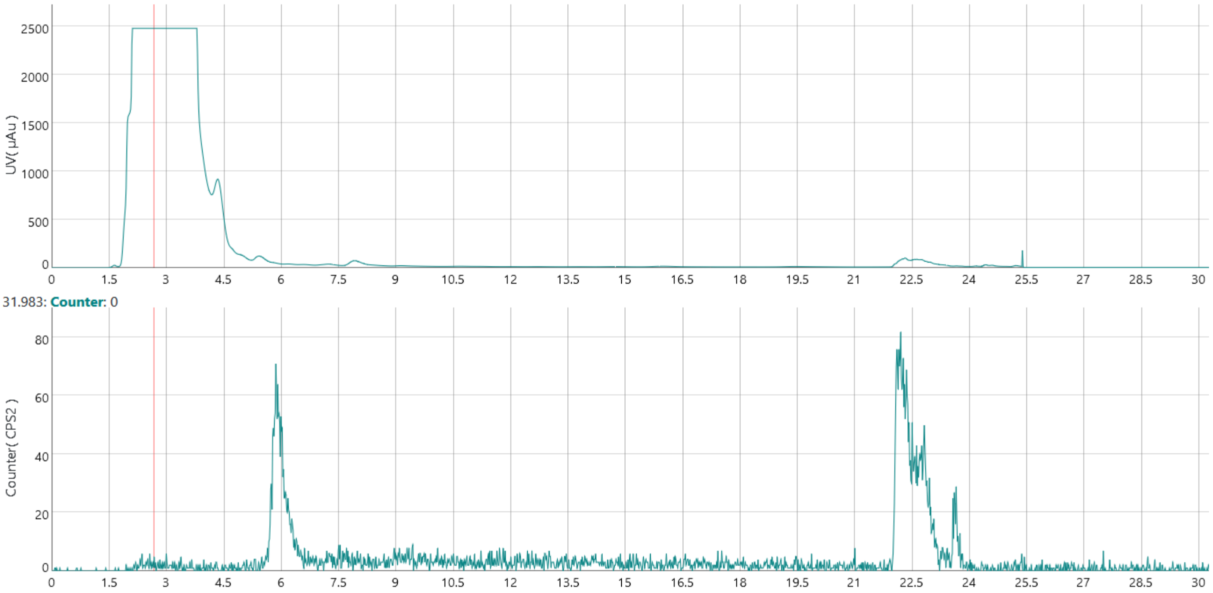

Figure S42: Purification of [^{18}F]**6b** ([^{18}F]JK-PSMA-16) by preparative HPLC (top: UV chromatogram, λ=254 nm; bottom: radio-chromatogram; column: Hydro-RP, 250×10 mm; eluent: 30% MeCN (0.1% TFA); flow rate: 4.7 mL/min; t_R = 22 min).

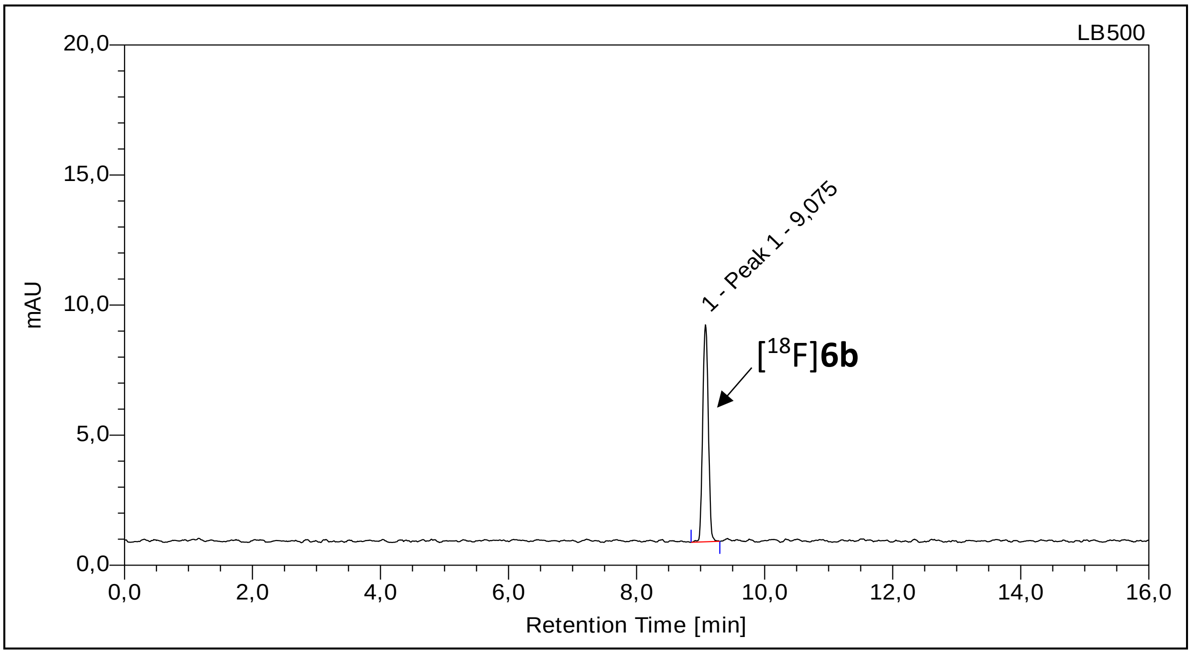

Figure S43: Radio-HPLC of purified [^{18}F]**6b** ([^{18}F]JK-PSMA-16), Method E (0.1% TFA).

5.20 HPLC traces of [^{18}F]**6c** (**Figs. S44** & **S45**)

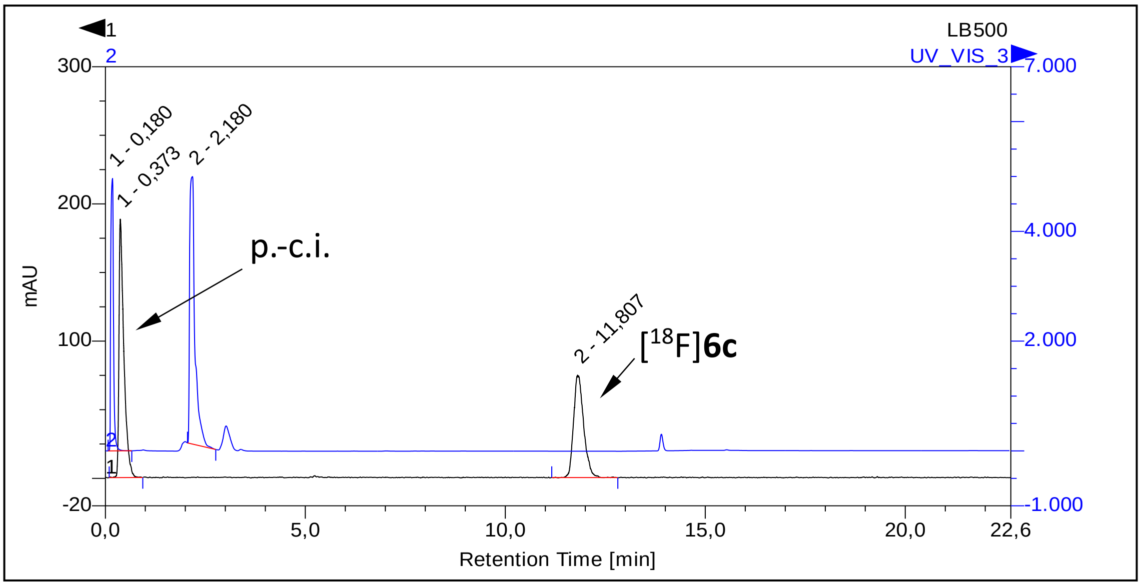

Figure S44: HPLC traces of crude [^{18}F]**6c**, Method A (0.1% TFA). Blue trace: UV, λ = 254 nm; black trace: radioactivity. Abbreviation: p.-c.i. – post-column injection.

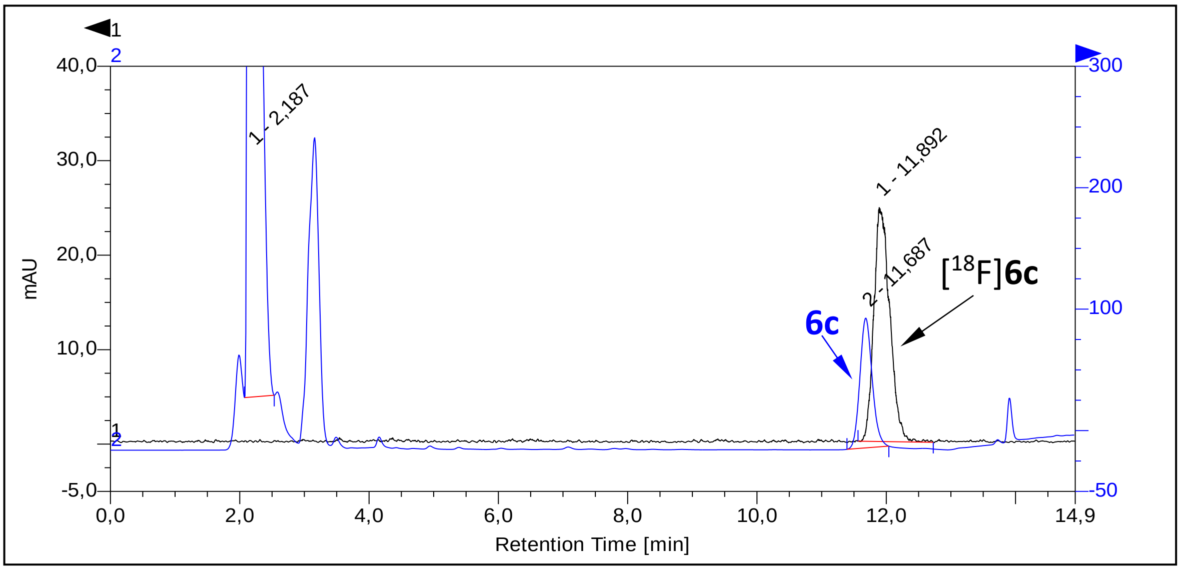

Figure S45: HPLC traces of crude [^{18}F]**6c** spiked with the non-radioactive reference compound, Method A (0.1% TFA). Blue trace: UV, λ = 254 nm; black trace: radioactivity.

5.21 HPLC traces of [^{18}F]**6d** ([^{18}F]JK-PSMA-18) (**Figs. S46**-**S49**)

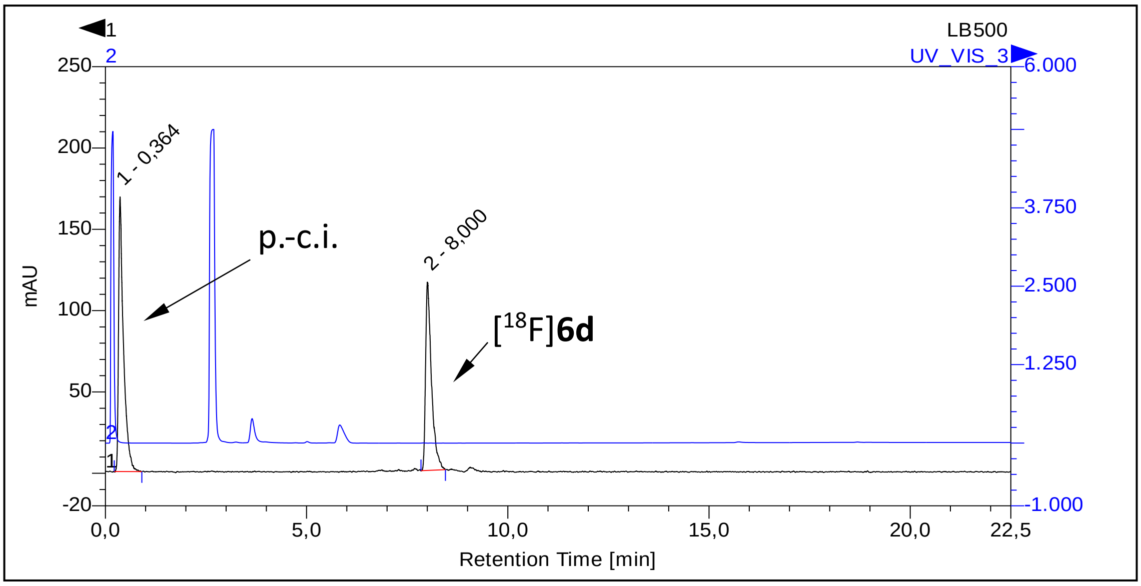

Figure S46: HPLC traces of crude [^{18}F]**6d** ([^{18}F]JK-PSMA-18), Method E (0.1% TFA). Blue trace: UV, λ = 254 nm; black trace: radioactivity. Abbreviation: p.-c.i. – post-column injection.

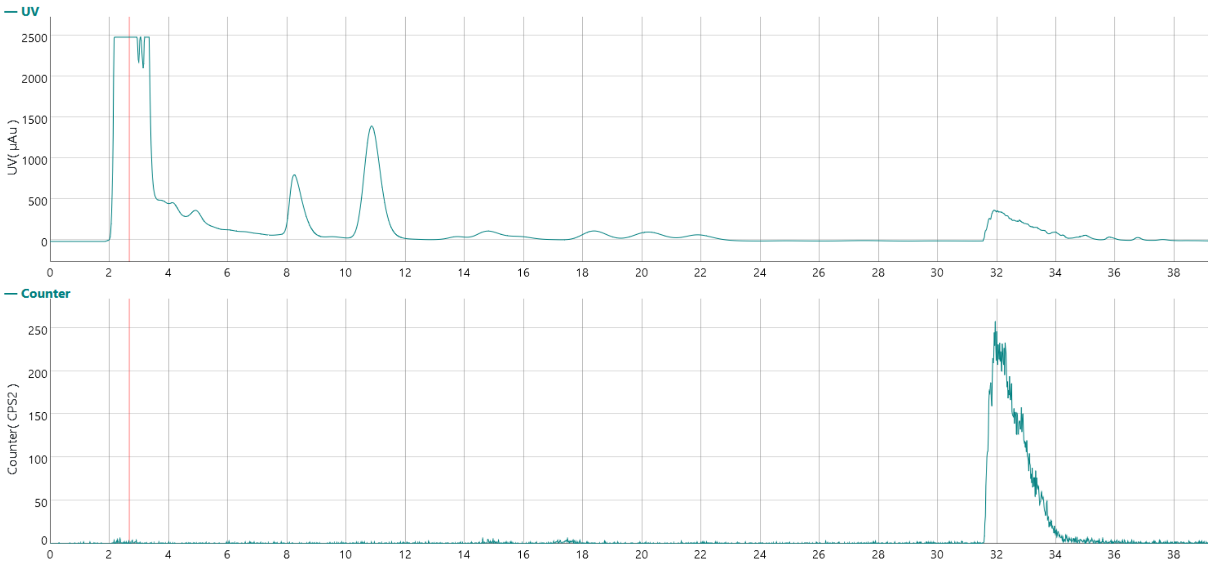

Figure S47: Purification of [^{18}F]**6d** ([^{18}F]JK-PSMA-18) by preparative HPLC (top: UV chromatogram, λ=254 nm; bottom: radio-chromatogram; column: Hydro-RP, 250×10 mm; eluent: 30% MeCN (0.1% TFA); flow rate: 4.7 mL/min; t_R = 32 min).

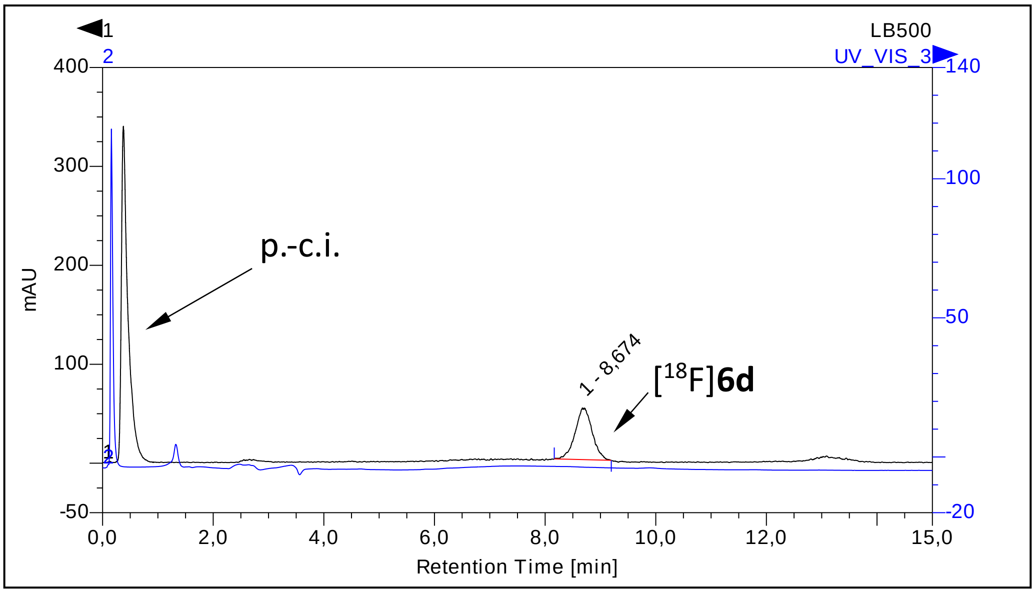

Figure S48: HPLC traces of purified [^{18}F]**6d** ([^{18}F]JK-PSMA-18), Method E (0.1% TFA). Blue trace: UV, λ = 254 nm; black trace: radioactivity. Abbreviation: p.-c.i. – post-column injection.

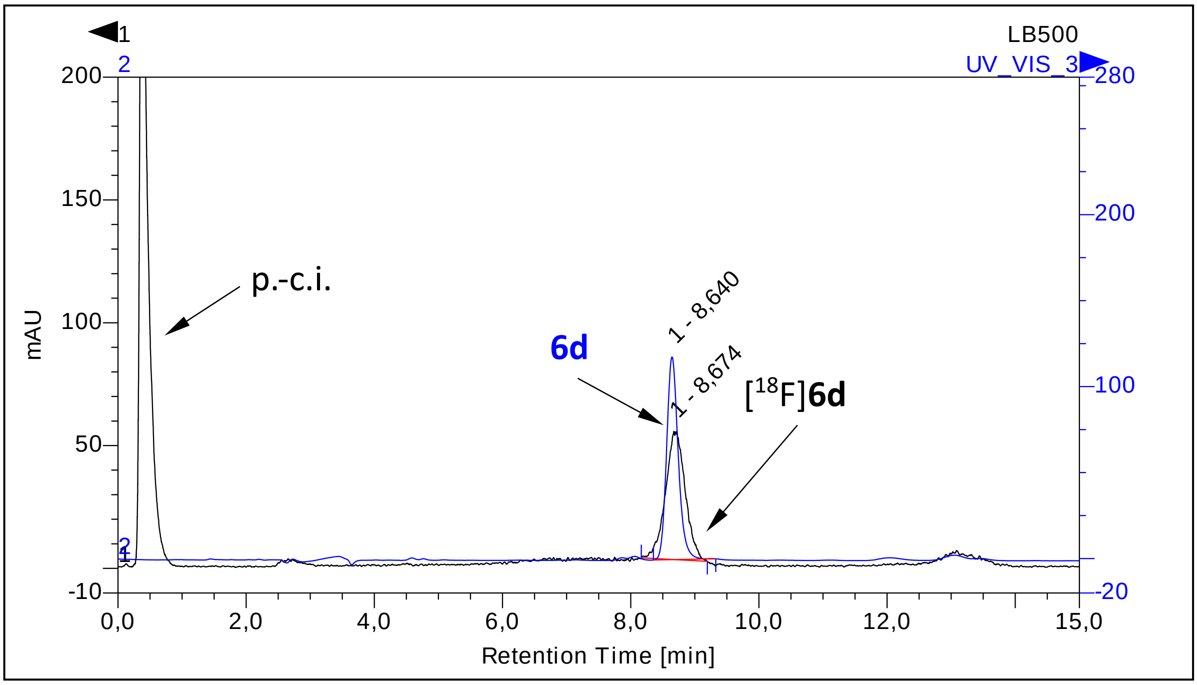

Figure S49: HPLC traces of [^{18}F]**6d** ([^{18}F]JK-PSMA-18) spiked with the non-radioactive reference compound, Method E (0.1% TFA). Blue trace: UV, λ = 254 nm; black trace: radioactivity. Abbreviation: p.-c.i. – post-column injection.

6 Calibration curves

6.1 Calibration curve for JK-PSMA-15

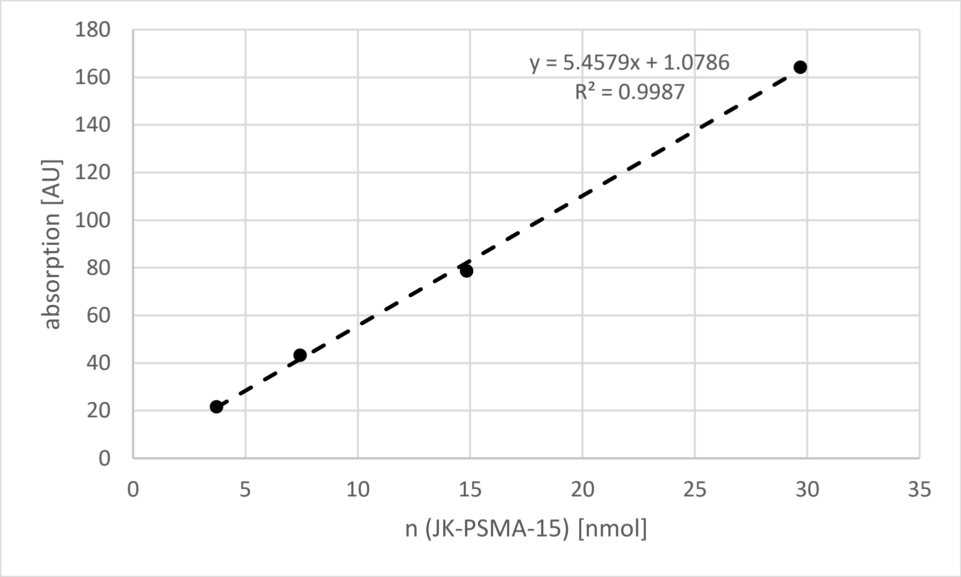

6.2 Calibration curve for JK-PSMA-16

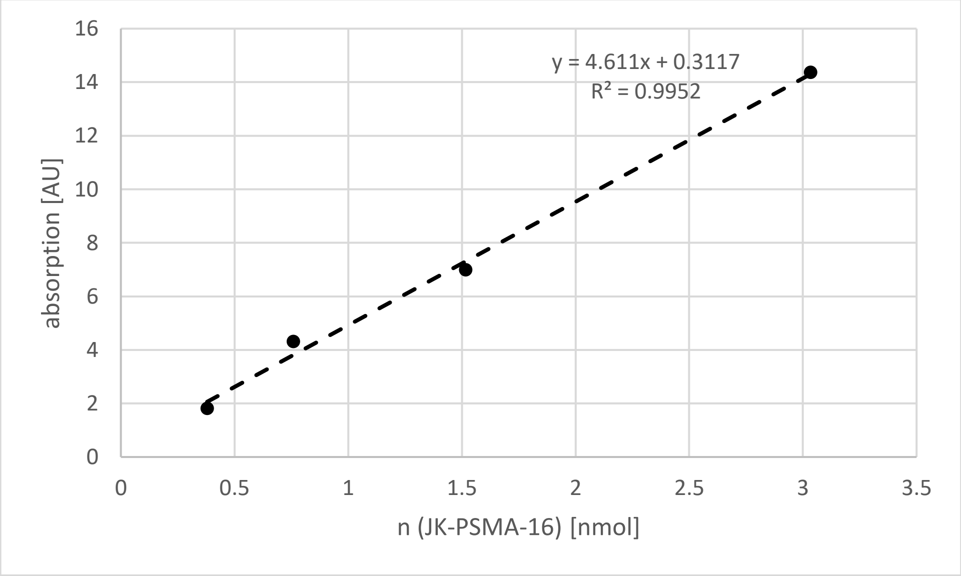

6.3 Calibration curve for JK-PSMA-18

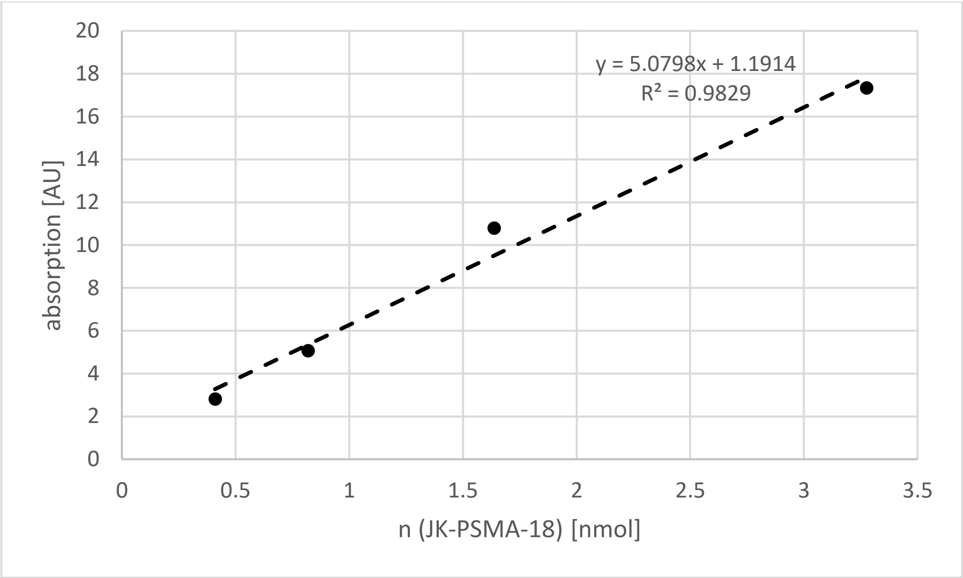

7 In vivo experiments

7.1 Methods

7.1.1 Signal-to-background ratio (SCG/background)

This parameter describes the difference between tracer uptake into the PSMA-positive ganglia and tracer uptake into PSMA-negative background tissues. It was calculated as the ratio of mean SUV_{bw} values determined in elliptical volumes of interest (VOIs) drawn over the PSMA-positive superior cervical ganglion (SCG: 150 voxels) and the PSMA-negative neck region dorsal from the spinal cord (background: 1600 voxels).

7.1.2 Acutance

The image intensity of a PSMA-positive ganglion decreases gradually towards surrounding background tissues, forming a slope which reflects the edge contrast or acutance. Accordingly, the acutance provides a measure for the perceived sharpness of the image and the ability to reliably measure the size of small PSMA-expressing tissues, which are important criteria for, e.g., the delineation of PSMA-positive metastases from surrounding PSMA-negative tissues. For quantification of the acutance in VINCI 5.21 (Max Planck Institute for Metabolism Research, Cologne, Germany), an 8 mm profile (1 pixel width) was placed over the middle of the SCG (diameter approx. 3.5 mm) in the horizontal plane. The slope of the profile plot was determined by dividing the maximum height of the SCG profile (peak minus background) by its full width at half maximum (FWHM). The time frame used for this analysis was 60–120 min p.i.

7.1.3 Resolution

The dorsal root ganglia (DRG) are arranged in pairs along the spinal cord, and the resolution describes how well the two ganglia of a pair can be separated from each other. Accordingly, the resolution provides a measure for the ability to distinguish two adjacent PSMA-expressing tissues, which is an important criterion for, e.g., the delineation of PSMA-positive metastases located close to each other or to other structures with high radioactivity retention. For quantification of the resolution in VINCI 5.21, a 12 mm profile was placed over the first cervical pair of the DRG (ganglion diameter approx. 2.5 mm; distance from center to center approx. 4 mm) in the horizontal plane. The resolution R was then calculated from the profile plot according to:

$$R = \frac{2(P1 - P2)}{1.7(FWHM1 + FWHM2)}$$

where P1-P2 is the distance between the two peaks corresponding to the ganglia and FWHM1 and FWHM2 are their full widths at half maximum.

7.2 Results (**Tab. S1-S5 & Fig. S50**)

Table S1: Values of injected dose and animal weight for the experiments with the newly developed PSMA-tracers.

Tracer	Injected dose [MBq]	Animal weight [g]	n
[^{18}F]JK-PSMA-15	62.8 ± 8.1	334 ± 31	3
[^{18}F]JK-PSMA-15 + 2-PMPA	62.4	302	1
[^{18}F]JK-PSMA-16	56.3 ± 8.1	290 ± 17	3
[^{18}F]JK-PSMA-16 + 2-PMPA	60.2	268	1
[^{18}F]JK-PSMA-18	61.8 ± 1.5	260 ± 25	3
[^{18}F]JK-PSMA-18 + 2-PMPA	58.4	237	1

Table S2: Properties of the newly developed PSMA-tracers in comparison to [^{18}F]JK-PSMA-7 at 90-120 min p.i.

	[^{18}F]JK-PSMA-7[a]	[^{18}F]JK-PSMA-15	[^{18}F]JK-PSMA-16	[^{18}F]JK-PSMA-18
SCG uptake, SUV_{bw}	31.3 ± 10.5	45.2 ± 8.2	19.1 ± 9.4	46.2 ± 9.7
Background uptake, SUV_{bw}	4.03 ± 1.00	4.05 ± 0.79	6.17 ± 3.02	6.78 ± 1.03
Acutance, %ID/g/mm	0.075 ± 0.027	0.079 ± 0.024	0.026 ± 0.015	0.086 ± 0.019
Resolution	1.095 ± 0.042	1.089 ± 0.144	1.032 ± 0.115	0.961 ± 0.157
Signal-to-background ratio	8.15 ± 1.71	11.49 ± 3.29	2.27 ± 0.24	7.04 ± 2.32

[a] Values for [^{18}F]JK-PSMA-7 are from[2].

Table S3: Statistical comparison of the tracer properties at 90-120 min p.i. using one-way ANOVA followed by Dunnett's multiple comparison test.

	ANOVA main effect	[^{18}F]JK-PSMA-15 vs. [^{18}F]JK-PSMA-7	[^{18}F]JK-PSMA-16 vs. [^{18}F]JK-PSMA-7	[^{18}F]JK-PSMA-18 vs. [^{18}F]JK-PSMA-7
SCG uptake, SUV_{bw}	$F(3,8)=5.5$; $p=0.0239$	p=0.2438	p=0.3283	p=0.2040
Background uptake, SUV_{bw}	$F(3,8)=2.2$; $p=0.1608$	not tested	not tested	not tested
Acutance, %ID/g/mm	$F(3,8)=3.5$; $p=0.0698$	not tested	not tested	not tested
Resolution	$F(3,7)=0.8$; $p=0.5516$	not tested	not tested	not tested
Signal-to-background ratio	$F(3,8)=9.1$; $p=0.0058$	p=0.2209	p=0.0273*	p=0.8656

* significant difference compared to [^{18}F]JK-PSMA-7.

Table S4: Signal-to-background ratios (mean±SD) measured during four consecutive 30 min time frames.

Tracer	0-30 min p.i.	30-60 min p.i.	60-90 min p.i.	90-120 min p.i.
[^{18}F]JK-PSMA-7[a]	3.82 ± 0.23	5.44 ± 0.56	7.04 ± 1.31	9.27 ± 2.12
[^{18}F]JK-PSMA-15	4.71 ± 0.54	7.57 ± 1.73	10.28 ± 2.95	14.02 ± 4.37
[^{18}F]JK-PSMA-16	2.54 ± 0.65	2.52 ± 0.65	2.86 ± 0.25	2.86 ± 0.16
[^{18}F]JK-PSMA-18	3.83 ± 0.55	4.71 ± 1.18	6.45 ± 2.11	8.02 ± 3.04

[a] Values for [^{18}F]JK-PSMA-7 are from[2].

Table S5: Comparison of signal-to-background ratios using 2-way ANOVA with factors "tracer" and "uptake period". Main effect for factor "tracer": $F(3,8)=10.08$; $p=0.0043$. Shown are results from Dunnett's multiple comparison test, where each novel tracer is compared with [^{18}F]JK-PSMA-7.

Tracer	0-30 min p.i.	30-60 min p.i.	60-90 min p.i.	90-120 min p.i.
[^{18}F]JK-PSMA-15	p=0.6554	p=0.4187	p=0.1303	p=0.0162*
[^{18}F]JK-PSMA-16	p=0.0708	p=0.1916	p=0.0378*	p=0.0011*
[^{18}F]JK-PSMA-18	p=0.3167	p=0.9428	p=0.9678	p=0.7813

* significant difference compared to [^{18}F]JK-PSMA-7.

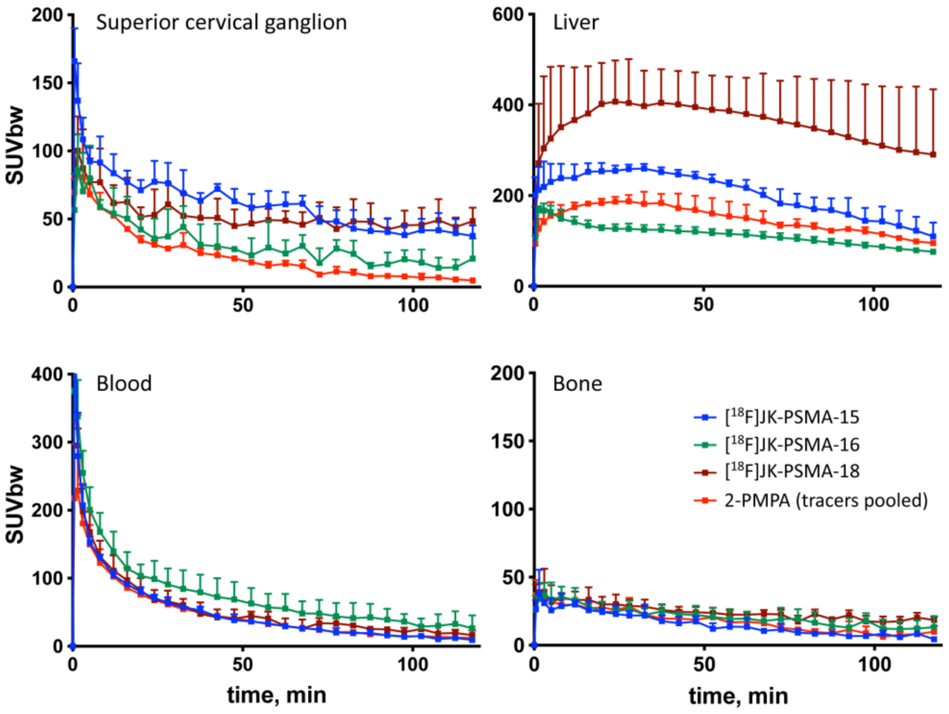

Figure S50: Comparison of time-activity curves (TACs) for the new PSMA-targeting tracers in the indicated volumes of interest (VOIs) measured without (n=3 per tracer) or with (n=1 per tracer, averaged across tracers) co-injection of the PSMA-inhibitor 2-PMPA. Note that one of the animals in the [^{18}F]JK-PSMA-18 group without co-injection of 2-PMPA showed unusually high liver uptake, as reflected in the higher variability of liver uptake for this tracer and the apparent difference between liver uptake with and without 2-PMPA co-injection.

8 References

(1) Krapf, P.; Richarz, R.; Urusova, E. A.; Neumaier, B.; Zlatopolskiy, B. D. Seyferth-Gilbert Homologation as a Route to ^{18}F-Labeled Building Blocks: Preparation of Radiofluor-inated Phenylacetylenes and Their Application in PET Chemistry. *European J. Org. Chem.* **2016**, *2016* (3), 430–433. https://doi.org/10.1002/ejoc.201501377.

(2) Zlatopolskiy, B. D.; Endepols, H.; Krapf, P.; Guliyev, M.; Urusova, E. A.; Richarz, R.; Hohberg, M.; Dietlein, M.; Drzezga, A.; Neumaier, B. Discovery of ^{18}F-JK-PSMA-7, a PET Probe for the Detection of Small PSMA-Positive Lesions. *J. Nucl. Med.* **2019**, *60* (6), 817–823. https://doi.org/10.2967/jnumed.118.218495.
